# Changes in birth certificate-reported prevalence of congenital anomalies at live birth during the COVID-19 pandemic: a nationwide study of 26 million United States live births

**DOI:** 10.3389/fcimb.2026.1914021

**Published:** 2026-09-29

**Authors:** Qinchang Chen, Dongpo Liang, Shushui Wang

**Affiliations:** Department of Pediatric Cardiology, Guangdong Cardiovascular Institute, Guangdong Provincial People’s Hospital (Guangdong Academy of Medical Sciences), Southern Medical University, Guangzhou, China

**Keywords:** birth certificates, congenital abnormalities, congenital heart defects, COVID-19, prevalence

## Abstract

**Background:**

Although the majority of studies have indicated that maternal severe acute respiratory syndrome coronavirus 2 (SARS-CoV-2) infection and coronavirus disease 2019 (COVID-19) vaccination are not associated with neonatal birth defects, it is relevant to examine whether the disease burden changed during the COVID-19 pandemic, as other influencing factors may have also changed. We aimed to compare the birth certificate-reported prevalence of congenital anomalies at live birth before and during the pandemic.

**Methods:**

Using public birth data, all live births from 2016 to 2022 in the United States of America (USA) were retrospectively included. The birth certificate-reported prevalence at live birth of selected congenital anomalies identified from the birth certificate, including anencephaly, meningomyelocele/spina bifida, cyanotic congenital heart disease (CCHD), congenital diaphragmatic hernia, omphalocele, gastroschisis, limb reduction defect, cleft lip, cleft palate, Down syndrome, chromosomal disorder, and hypospadias, was estimated and compared by calendar year of birth using logistic regression.

**Results:**

A total of 26,345,765 live-born infants from 2016 to 2022 were included. The mean gestational age of the infants was 38.4 weeks (SD = 2.1 weeks), 48.9% were female babies, and 96.7% were singleton. The overall reported prevalence of congenital anomalies was highest in 2018 (3.51‰, 95%CI = 3.45‰–3.57‰) and has since shown a decreasing trend [3.38‰ (95%CI = 3.32‰–3.44‰) in 2019 and 3.35‰ (95%CI = 3.29‰–3.41‰) in 2022]. Using 2019 as a reference, it did not differ in 2020 and 2021 [odds ratio (OR) = 1.01 (0.98–1.03) and OR = 1.00 (0.98–1.03), respectively, after adjusting for maternal age, ethnicity/race, and socioeconomic status] and was slightly lower in 2022 [OR = 0.97 (0.95–0.99)]. For the majority of the congenital anomalies, the reported prevalence during the pandemic (2020–2022) remained unchanged or showed a decreasing trend (i.e., gastroschisis and Down syndrome) compared with that in 2019. The reported prevalence of CCHD was higher in 2021 and 2022 than in 2019. However, the increase had begun before the pandemic, and after correction for multiple comparisons, only the 2021 contrast remained significant. Segmented regression confirmed a significant pre-pandemic upward trend in CCHD with a level change dated to infants born in March 2020. When exposure was re-indexed by estimated year of conception, neither pandemic conception cohort differed from the 2019 conception cohort [2020: OR = 1.03 (0.97–1.09); 2021: OR = 1.02 (0.97–1.08)]. The direction of these results was consistent when stratified by gestational age (≥37 or <37 weeks), with wider intervals in the preterm stratum.

**Conclusions:**

The birth certificate-reported prevalence of congenital anomalies at live birth did not increase during the COVID-19 pandemic in the USA and decreased for several specific anomalies. The higher reported prevalence of CCHD continues a trend that began before the pandemic and is null once exposure is aligned with organogenesis.

## Introduction

Concerns about potential vertical transmission or teratogenic effects of severe acute respiratory syndrome coronavirus 2 (SARS-CoV-2) were raised when the coronavirus disease 2019 (COVID-19) pandemic started in 2020 (Vivanti et al., 2020; Wadman, 2020). There was also doubt about the safety of the COVID-19 vaccine in pregnant women, which may explain the COVID-19 vaccine hesitancy in this population (Casubhoy et al., 2024). Assuming that 6%–9% of pregnant women contract COVID-19 during pregnancy (Ellington et al., 2020; Le Ray et al., 2022) and 50% of pregnant women take a COVID-19 vaccine (Norman et al., 2024), the absolute population size concerning this issue (i.e., potential effects of COVID-19 or COVID-19 vaccination during pregnancy) is no doubt substantial.

Fortunately, to date, research suggests that vertical transmission of SARS-CoV-2 is likely to be rare (Allotey et al., 2022). COVID-19 appears not to be associated with pregnancy loss (van Baar et al., 2024) or stillbirth (Smith et al., 2023) (despite the evidence being inconsistent) (DeSisto et al., 2021; Alcover et al., 2023), although it may be associated with more preterm birth (Smith et al., 2023). Moreover, COVID-19 vaccination is safe for pregnant women (Watanabe et al., 2022; Kharbanda et al., 2023). Regarding birth defects, as summarized in the **Supplementary Material**, the majority of studies have reported no increase in the risk of congenital anomalies in infants born to women with COVID-19 or had COVID-19 vaccination during pregnancy (Neelam et al., 2023; Kharbanda et al., 2024; Magnus et al., 2024). However, a few studies have reported an increase during the pandemic in the risk of persistent pulmonary hypertension (Hasan et al., 2023), situs inversus (Wang et al., 2023; Qiu et al., 2024)/fetal mirror-image dextrocardia (Xu et al., 2024), biliary atresia (Nassar et al., 2024), non-syndromic orofacial clefts (Molnárová et al., 2024), and congenital microcephaly (Auger et al., 2024).

Although it is often of the most research interest to explore whether maternal COVID-19 is causally associated with neonatal congenital anomalies, it is challenging to examine causal inference when only observational studies are practical. Apart from the confounding issue, diagnostic practices may have also changed during the pandemic (i.e., either with more or less screening). Therefore, evidence about congenital anomalies should be more carefully interpreted compared with other pregnancy/perinatal outcomes. Nevertheless, regardless of the causes, it is relevant to public health to have knowledge of whether the disease burden of congenital anomalies has changed during the pandemic, as congenital anomalies often require long-term management and an increase may lead to higher healthcare demand in the future. To our knowledge, such evidence is currently unavailable. Therefore, we conducted a nationwide population-based cohort study to describe the birth certificate-reported prevalence of congenital anomalies at live birth before and during the COVID-19 pandemic.

## Methods

### Data source

The study used the public use birth data files (i.e., the natality public use file in 2016–2022) accessed via the Vital Statistics Online Data Portal sourced from the National Vital Statistics System (NVSS), which is administered by the National Center for Health Statistics (NCHS). NCHS is the principal health statistics agency of the USA, which is part of the Centers for Disease Control and Prevention (CDC), Department of Health and Human Services. In the USA, all births (regardless of the length of gestation or birth weight) are required to be registered with a birth certificate according to state laws, and national collection and publication of the data (in addition to other vital statistics data) are mandatory by federal law. NCHS cooperates with the states and provides the public with access to statistical information from birth certificates via the NVSS.

The micro-data files we accessed provide deidentified individual-level data retrieved from the US standard certificate of live birth, the 2003 revision. All live births occurring to US residents and non-residents within the USA between 2016 and 2022 were included. The data had been processed by NCHS according to its data release policy (e.g., geographic identifiers, with the exact day of birth removed). Several data items on the standard birth certificate were also dropped from the data files by the NCHS due to poor data quality. Details about the data source can be found on the website of the NCHS (https://www.cdc.gov/nchs/index.htm). Details about the variables included in the study are presented in **Supplementary Methods**.

The study protocol was reviewed and approved by the Institutional Review Board of Guangdong Provincial People’s Hospital (no. KY2024-622-01). Informed consent was waived due to the data being already deidentified. We followed the Strengthening the Reporting of Observational Studies in Epidemiology (STROBE) statement (von Elm et al., 2007).

### Study population

All live births delivered between 2016 and 2022 in the accessed natality micro-data were included in the study population. No exclusion criterion was applied. However, for each study outcome (stated below), infants with a missing outcome were excluded from the specific analysis.

### Exposure

The calendar year of birth was from 2016 to 2022. We note explicitly that the calendar year of birth is an imperfect index of pandemic exposure. Because gestation lasts approximately 9 months, infants born in the first months of 2020 were conceived around spring 2019 and completed organogenesis before the pandemic began, and only infants born from approximately December 2020 onwards had a first trimester that fell entirely within the pandemic. The consequences of this conception-to-birth offset were addressed by the conception-indexed sensitivity analysis and by the choice of onset points for the segmented regression, both described below.

### Outcome measures

The following 12 specific congenital anomalies were studied as outcomes: anencephaly, meningomyelocele/spina bifida, cyanotic congenital heart disease (CCHD), congenital diaphragmatic hernia, omphalocele, gastroschisis, limb reduction defect, cleft lip with or without cleft palate, cleft palate alone, Down syndrome, suspected chromosomal disorder, and hypospadias. They were selected because they are listed as congenital anomalies on the US standard certificate of live birth, the 2003 revision. In addition, a composite of the 12 congenital anomalies was studied as a study outcome. We also included two further outcomes: transfer within 24 h of delivery and death before birth certificate registration (recorded on the certificate as the infant not being living at the time of the report).

The birth certificate provides a checkbox only: no diagnostic criteria, imaging confirmation, postnatal follow-up, or ICD coding is required, and the only operational case definition that exists for these data is the wording of the NCHS Guide to Completing the Facility Worksheets (https://www.cdc.gov/nchs/nvss/facility-worksheets-guide.htm). **Supplementary Table S1** therefore reproduces, for each of the 12 anomaly items and for the two further outcomes, the definition, the certifier instruction, the prescribed record source, and the keyword list given in that guide. The edition applicable throughout 2016–2022 was used; therefore, the instructions themselves did not change during the study period. For this reason, we describe our outcome throughout as the birth certificate-reported prevalence at live birth rather than as the incidence or the birth prevalence of the conditions. The item recorded as death before birth certificate registration captures only the deaths occurring before the certificate is filed, which is typically within the first days of life: it does not capture late neonatal, post-neonatal, or post-discharge deaths. Moreover, because it is administratively determined, changes in registration practice can alter the fraction captured for reasons unrelated to infant survival.

Missingness of the study outcomes by birth year is presented in **Supplementary Table S2**. Missingness of the anomaly items was 0.13%–0.17% in 2016–2020, but rose to 0.21% in 2021 and 0.20% in 2022, and missingness of the transfer item rose monotonically from 0.06% in 2018 to 0.24% in 2022. The reporting process therefore did change over the study period. For Down syndrome and suspected chromosomal disorder, the data are categorized as “pending” and “confirmed,” reflecting whether karyotype confirmation had been obtained before the certificate was registered. They were taken as one category in the main analyses, and the analyses restricted to each category separately are reported as a sensitivity analysis.

### Covariates

The following covariates were included into the study: 1) maternal age; maternal residence; maternal ethnicity/race; maternal education attainment; principal source of payment for current delivery; and marital status; 2) number of previous live births now dead; number of other (previous) pregnancy outcomes; (timing of the) first prenatal care visit; participation in the Special Supplemental Nutrition Program for Women, Infants, and Children (WIC food) during pregnancy; cigarette smoking during pregnancy; and maternal body mass index (BMI); 3) pre-pregnancy diabetes; gestational diabetes; pre-pregnancy hypertension; gestational hypertension; (current) pregnancy resulted from infertility treatment; fertility enhancing drugs/artificial insemination/intrauterine insemination; and assisted reproductive technology; 4) ruptured uterus; unplanned hysterectomy; and infections present and/or treated during current pregnancy; 5) obstetric estimate of gestation; birth place; and attendant at birth; 6) induction of labor; augmentation of labor; steroids (glucocorticoids) for fetal lung maturation received by the mother prior to delivery; antibiotics received by the mother during labor; clinical chorioamnionitis diagnosed during labor or maternal temperature ≥38°C; and epidural or spinal anesthesia during labor; 7) method of delivery; fetal presentation at birth; final route and method of delivery; and a trial of labor attempted if cesarean; and 8) plurality; sex of the infant; birthweight; Apgar score at 5 min; assisted ventilation required immediately following delivery; assisted ventilation required for more than 6 h; Neonatal Intensive Care Unit (NICU) admission; newborn given surfactant replacement therapy; antibiotics received by the newborn for suspected neonatal sepsis; and seizure or serious neurologic dysfunction.

Details about the variables involved in the study are presented in **Supplementary Methods**. Majority of the covariates were categorical variables directly provided by the original data, and the default categorizations were used in the study. For categorical variables, the category “unknown” was used for missing values. For continuous variables, the missingness was as follows: number of previous live births now dead (0.4%), number of other (previous) pregnancy outcomes (0.5%), obstetric estimate of gestation (0.1%), and birthweight (0.1%). As the nature of this study is descriptive epidemiology, we included these covariates to describe the maternal and perinatal characteristics of the birth cohorts in different years. Of these covariates, 14 were used in the additional adjustment analyses for CCHD described below: the 12 maternal and prenatal covariates (i.e., timing of the first prenatal care visit, WIC participation, cigarette smoking during pregnancy, maternal BMI, pre-pregnancy diabetes, gestational diabetes, pre-pregnancy hypertension, gestational hypertension, pregnancy resulting from infertility treatment, fertility enhancing drugs/artificial insemination/intrauterine insemination, assisted reproductive technology, and infections present and/or treated during the current pregnancy), together with plurality and sex of the infant.

### Statistical analysis

The summary statistics of the maternal and perinatal characteristics were presented by birth year, where the median and 25th–75th percentile [or the mean and standard deviation (SD)] were used for continuous variables and counts and percentages for categorical variables.

For the primary analysis, we first estimated the reported prevalence of each study outcome within each birth cohort by dividing the number of outcome events by the total number of births born in a calendar year, with the 95% confidence interval (CI) estimated as the Clopper–Pearson interval. To further estimate the relative difference, we performed logistic regression to obtain the odds ratio (OR) of each study outcome in each birth year *versus* the birth cohort 2019. As the scope of the study was only to describe the time trend (instead of answering a causal question), we only adjusted for maternal demographics and socioeconomic status, including maternal age, residence, ethnicity/race, education attainment, principal source of payment for current delivery, and marital status. Subgroup analyses were performed among infants delivered at ≥37 or <37 weeks’ gestation.

To address multiplicity, exact *p*-values were recomputed from the fitted adjusted models and were corrected within a prespecified primary family comprising the 12 specific congenital anomalies by the three pandemic year contrasts (2020, 2021, and 2022 *versus* 2019; *m* = 36). An extended family additionally including the three pre-pandemic contrasts (*m* = 72) is reported as a sensitivity analysis. The composite outcome and the two further outcomes were prespecified as separate outcomes and are reported outside the family, while the gestational age-stratified analyses are sensitivity analyses and were not counted towards it. Bonferroni-adjusted confidence intervals and *p*-values and Benjamini–Hochberg *q*-values were estimated.

As an exploratory analysis, if there was any study outcome showing an increasing trend during the pandemic (i.e., in the 2020–2022 birth cohort), we additionally adjusted for each other available covariate individually to determine how the OR (*versus* birth cohort 2019) would change, although such a naive method is subject to non-collapsibility (Whitcomb and Naimi, 2021). Only the maternal or prenatal covariates were considered for this analysis. Because individual adjustment does not account for the joint influence of the covariates, we also fitted a fully adjusted model entering all maternal and prenatal covariates simultaneously, as well as a further model additionally including plurality and sex of the infant, together with generalized variance inflation factors. Several categories of the birth certificate risk factor items are structurally redundant: the “unknown or not stated” categories of the diabetes, hypertension, and infertility treatment items are recorded jointly on the certificate and mark the same records, while the “not applicable” categories of the fertility-enhancing drug and assisted reproductive technology items are determined by construction by the infertility treatment item. These items were therefore reparameterized into a single “unknown” indicator and a single fertility variable built from the observed combinations of the three fertility items. The reparameterization spans the same column space as the fitting routine estimates, thus the birth year ORs being unchanged, but it removes the redundant columns so that variance inflation factors are defined.

Of note is that, in the data we accessed, no variable was available for identifying pairs of the same multiple births; thus, the non-independence of observations within multiple births could not be modeled. As a quantitative check, we repeated the primary analysis restricted to singleton births, in whom observations are independent by construction, and also repeated the analyses of Down syndrome and suspected chromosomal disorder separately among records marked “confirmed” and among records marked “pending.

To align exposure with the period of organogenesis, we repeated the primary comparison indexing each infant by estimated year of conception. Conception was taken as the last menstrual period plus 2 weeks, derived from the obstetric estimate of gestation, with birth placed at the middle of the reported birth month because NCHS does not release the exact day of birth. Within the 2016–2022 birth file, the conception cohorts are complete only for conception years 2016–2021, to which this analysis was restricted. Conception year 2019 is the reference, and conception years 2020 and 2021 are the cohorts whose first trimester fell within the pandemic.

The monthly reported prevalence of each study outcome was estimated by dividing the number of outcome events by the total number of births born in a calendar month within a calendar year. To distinguish a change at the onset of the pandemic from the continuation of a preexisting secular trend, we performed a segmented regression (interrupted time series) of the monthly series. Monthly event counts (84 observations per outcome and subgroup) were modeled with a quasi-Poisson log-linear model with the monthly number of live births as offset, including a linear pre-onset time trend, an immediate level change, a subsequent slope change, and the first and second Fourier harmonics of the annual cycle. The Newey–West standard errors with lag 4 were used to accommodate residual autocorrelation. Two onset points were prespecified: March 2020, the first infants born after the pandemic was declared, and December 2020, the first infants whose first trimester fell entirely within the pandemic.

All analyses were performed using the R programming language (version 4.4.1).

## Results

### Maternal and perinatal characteristics

A total of 26,345,765 live births in 2016–2022 were included, where the number of newborns decreased continuously from 3,956,112 in 2016 to 3,619,826 in 2020, but increased to 3,676,029 in 2022. They were born to mothers of a median age of 29 years (25–33 years). Among the infants, 51.2% were born to mothers of non-Hispanic White ethnicity/race, followed by Hispanic (24.0%) and non-Hispanic Black (14.4%), and 33.2% had an education attainment of bachelor’s degree or higher. Over the years, maternal age slightly increased. The proportions of non-Hispanic White slightly decreased, and both Hispanic and non-Hispanic Black increased; however, the increasing trend in non-Hispanic Black was reversed to a decreasing trend in 2020–2022 (**Table 1**; **Supplementary Table S3**).

**Table 1:** Maternal and perinatal characteristics of the infants by birth year.

Birth year	Overall	2016	2017	2018	2019	2020	2021	2022
*n*	26,345,765	3,956,112	3,864,754	3,801,534	3,757,582	3,619,826	3,669,928	3,676,029
Maternal age (years)	29 (25–33)	29 (24–33)	29 (25–33)	29 (25–33)	29 (25–33)	29 (25–33)	30 (25–34)	30 (25–34)
Maternal ethnicity/race								
Non-Hispanic White (only)	13,498,775 (51.2)	2,057,311 (52.0)	1,993,312 (51.6)	1,957,261 (51.5)	1,917,034 (51.0)	1,844,161 (50.9)	1,888,274 (51.5)	1,841,422 (50.1)
Non-Hispanic Black (only)	3,781,720 (14.4)	559,235 (14.1)	561,297 (14.5)	552,630 (14.5)	548,719 (14.6)	530,036 (14.6)	518,108 (14.1)	511,695 (13.9)
Non-Hispanic AIAN (only)	197,723 (0.8)	31,465 (0.8)	29,967 (0.8)	29,115 (0.8)	28,493 (0.8)	26,822 (0.7)	26,129 (0.7)	25,732 (0.7)
Non-Hispanic Asian (only)	1,637,346 (6.2)	254,870 (6.4)	249,616 (6.5)	241,223 (6.3)	239,189 (6.4)	219,309 (6.1)	213,947 (5.8)	219,192 (6.0)
Non-Hispanic NHOPI (only)	67,339 (0.3)	9,350 (0.2)	9,436 (0.2)	9,481 (0.2)	9,778 (0.3)	9,630 (0.3)	9,536 (0.3)	10,128 (0.3)
Non-Hispanic more than one race	591,343 (2.2)	80,954 (2.0)	82,455 (2.1)	83,889 (2.2)	84,358 (2.2)	84,266 (2.3)	87,013 (2.4)	88,408 (2.4)
Hispanic	6,326,119 (24.0)	926,461 (23.4)	905,998 (23.4)	893,897 (23.5)	893,901 (23.8)	871,385 (24.1)	890,278 (24.3)	944,199 (25.7)
Unknown	245,400 (0.9)	36,466 (0.9)	32,673 (0.8)	34,038 (0.9)	36,110 (1.0)	34,217 (0.9)	36,643 (1.0)	35,253 (1.0)
No. previous live births now dead	0 (0–0)	0 (0–0)	0 (0–0)	0 (0–0)	0 (0–0)	0 (0–0)	0 (0–0)	0 (0–0)
No. other pregnancy outcomes	0 (0–1)	0 (0–1)	0 (0–1)	0 (0–1)	0 (0–1)	0 (0–1)	0 (0–1)	0 (0–1)
First prenatal care visit (months)								
1–3	19,918,931 (75.6)	2,961,603 (74.9)	2,907,955 (75.2)	2,868,985 (75.5)	2,847,057 (75.8)	2,754,025 (76.1)	2,813,011 (76.7)	2,766,295 (75.3)
4–6	4,165,412 (15.8)	641,956 (16.2)	620,205 (16.0)	604,379 (15.9)	589,380 (15.7)	571,173 (15.8)	552,469 (15.1)	585,850 (15.9)
≥7	1,153,561 (4.4)	177,097 (4.5)	171,030 (4.4)	168,033 (4.4)	167,156 (4.4)	151,962 (4.2)	151,863 (4.1)	166,420 (4.5)
No prenatal care	483,315 (1.8)	62,062 (1.6)	66,824 (1.7)	64,465 (1.7)	68,800 (1.8)	68,386 (1.9)	74,900 (2.0)	77,878 (2.1)
Unknown	624,546 (2.4)	113,394 (2.9)	98,740 (2.6)	95,672 (2.5)	85,189 (2.3)	74,280 (2.1)	77,685 (2.1)	79,586 (2.2)
Obstetric estimate of gestation (completed weeks)	38.4 (2.1)	38.5 (2.1)	38.5 (2.1)	38.4 (2.1)	38.4 (2.1)	38.4 (2.1)	38.3 (2.1)	38.3 (2.1)
<37	2,667,175 (10.1)	388,876 (9.8)	383,341 (9.9)	380,452 (10.0)	383,818 (10.2)	365,000 (10.1)	384,457 (10.5)	381,231 (10.4)
≥37	23,658,597 (89.8)	3,563,723 (90.1)	3,478,586 (90.0)	3,418,511 (89.9)	3,371,226 (89.7)	3,252,213 (89.8)	3,282,345 (89.4)	3,291,993 (89.6)
Unknown	19,993 (0.1)	3,513 (0.1)	2,827 (0.1)	2,571 (0.1)	2,538 (0.1)	2,613 (0.1)	3,126 (0.1)	2,805 (0.1)
Birth place								
In hospital	25,852,646 (98.1)	3,892,038 (98.4)	3,801,226 (98.4)	3,737,752 (98.3)	3,693,867 (98.3)	3,546,948 (98.0)	3,588,681 (97.8)	3,592,134 (97.7)
Not in hospital	491,764 (1.9)	63,824 (1.6)	63,326 (1.6)	63,510 (1.7)	63,577 (1.7)	72,680 (2.0)	81,086 (2.2)	83,761 (2.3)
Unknown	1,355 (0.0)	250 (0.0)	202 (0.0)	272 (0.0)	138 (0.0)	198 (0.0)	161 (0.0)	134 (0.0)
Method of delivery								
Vaginal	17,922,435 (68.0)	2,691,523 (68.0)	2,627,140 (68.0)	2,588,534 (68.1)	2,565,529 (68.3)	2,467,001 (68.2)	2,490,507 (67.9)	2,492,201 (67.8)
C-section	8,406,533 (31.9)	1,261,949 (31.9)	1,235,362 (32.0)	1,211,372 (31.9)	1,189,718 (31.7)	1,150,672 (31.8)	1,176,445 (32.1)	1,181,015 (32.1)
Unknown	16,797 (0.1)	2,640 (0.1)	2,252 (0.1)	1,628 (0.0)	2,335 (0.1)	2,153 (0.1)	2,976 (0.1)	2,813 (0.1)
Plurality								
Single	25,475,170 (96.7)	3,820,036 (96.6)	3,732,218 (96.6)	3,674,115 (96.6)	3,633,587 (96.7)	3,504,248 (96.8)	3,552,604 (96.8)	3,558,362 (96.8)
Twin	847,052 (3.2)	132,061 (3.3)	128,608 (3.3)	123,867 (3.3)	120,691 (3.2)	112,691 (3.1)	114,371 (3.1)	114,763 (3.1)
Triplet	22,356 (0.1)	3,767 (0.1)	3,678 (0.1)	3,427 (0.1)	3,154 (0.1)	2,750 (0.1)	2,797 (0.1)	2,783 (0.1)
Quadruplet or higher	1,187 (0.0)	248 (0.0)	250 (0.0)	125 (0.0)	150 (0.0)	137 (0.0)	156 (0.0)	121 (0.0)
Sex of the infant								
Male	13,473,427 (51.1)	2,023,449 (51.1)	1,977,663 (51.2)	1,943,273 (51.1)	1,922,674 (51.2)	1,851,270 (51.1)	1,876,379 (51.1)	1,878,719 (51.1)
Female	12,872,338 (48.9)	1,932,663 (48.9)	1,887,091 (48.8)	1,858,261 (48.9)	1,834,908 (48.8)	1,768,556 (48.9)	1,793,549 (48.9)	1,797,310 (48.9)
Birthweight (g)	3,293 (,2954–3,628)	3,310 (2,970–3,630)	3,300 (2,960–3,629)	3,300 (2,960-3,629)	3,290 (2,953–3,620)	3,290 (2,954–3,620)	3,289 (2,948–3,620)	3,285 (2,945–3,605)
Apgar score at 5 min	9 (9–9)	9 (9–9)	9 (9–9)	9 (9–9)	9 (9–9)	9 (9–9)	9 (9–9)	9 (9–9)
0–3	153,984 (0.6)	23,905 (0.6)	22,981 (0.6)	21,571 (0.6)	21,749 (0.6)	20,970 (0.6)	21,374 (0.6)	21,434 (0.6)
4–6	391,470 (1.5)	55,640 (1.4)	54,474 (1.4)	54,019 (1.4)	55,585 (1.5)	54,744 (1.5)	58,318 (1.6)	58,690 (1.6)
7–8	3,386,031 (12.9)	479,716 (12.1)	462,924 (12.0)	457,065 (12.0)	495,375 (13.2)	478,165 (13.2)	504,286 (13.7)	508,500 (13.8)
9–10	22,305,386 (84.7)	3,380,763 (85.5)	3,309,639 (85.6)	3,253,860 (85.6)	3,170,200 (84.4)	3,050,473 (84.3)	3,069,294 (83.6)	3,071,157 (83.5)
Unknown	108,894 (0.4)	16,088 (0.4)	14,736 (0.4)	15,019 (0.4)	14,673 (0.4)	15,474 (0.4)	16,656 (0.5)	16,248 (0.4)

Details about each variable are presented in **Supplementary Methods**. Other maternal and perinatal characteristics of the infants are presented in **Supplementary Table S3**.

*AIAN*, American Indian or Alaskan Native; *NHOPI*, Native Hawaiian or Other Pacific Islander

Regarding perinatal characteristics, 75.6% of births had the first prenatal care visit in the first 3 months of gestation, increasing from 74.9% in 2016 to a peak of 76.7% in 2021, and approximately 2% had no prenatal care. The proportion of cigarette smoking during pregnancy decreased from 7.1% in 2016 to 3.7% in 2022, while WIC participation declined from 39.0% to 29.8%. The proportions of infants born to mothers with overweight/obesity, diabetes, and hypertension (both chronic and gestational) all increased. The proportion of pregnancies resulting from infertility treatment also increased, mainly driven by the increase in the use of assisted reproductive technology (from 1.0% to 1.7%).

The mean obstetric estimate of gestation of the infants was 38.4 weeks (SD = 2.1 weeks), with a median birthweight of 3,293 g (2,954–3,628 g). Among them, 48.9% were female infants, and 96.7% were singleton. These statistics were similar over the years, but the proportion of preterm birth was slightly higher in 2021–2022 than in previous years (i.e., approximately 10.5% *versus* 10.0%). The vast majority (98.1%) of infants were born in hospitals; however, in 2020–2022, the proportion of not being born in hospitals was slightly higher than that before the pandemic. Furthermore, 31.9% of infants were delivered by cesarean section, consistent with data from previous years. From 2016 to 2022, the proportion of infants receiving assisted ventilation immediately after delivery and for more than 6 h increased, as did the proportion of NICU admissions. Details about the investigated maternal and perinatal characteristics are presented in **Table 1** and **Supplementary Table S3**.

### Reported prevalence of congenital anomalies between birth years

The overall reported prevalence of congenital anomalies at live birth was 3.49‰ (95%CI = 3.43‰–3.55‰) in the birth cohort 2016 and was highest in 2018 (3.51‰, 95%CI = 3.45‰–3.57‰). It has since shown a decreasing trend: 3.38‰ (95%CI = 3.32‰–3.44‰) in 2019 and 3.35‰ (95%CI = 3.29‰–3.41‰) in 2022. Using the data from 2019 as a reference, it did not differ significantly in 2020 and 2021 after adjusting for maternal age, ethnicity/race, and socioeconomic status [OR = 1.01 (0.98–1.03) and OR = 1.00 (0.98–1.03), respectively], but was significantly lower in 2022 [OR = 0.97 (0.95–0.99)].

Among the investigated congenital anomalies, CCHD (61 per 100,000 births), hypospadias (57 per 100,000 births), Down syndrome (53 per 100,000 births), cleft lip with or without cleft palate (52 per 100,000 births), and suspected chromosomal disorder (45 per 100,000 births) showed the highest reported prevalence. As shown in **Figure 1** and **Supplementary Table S4**, for the majority of congenital anomalies, the reported prevalence during the pandemic remained constant, while that of gastroschisis and Down syndrome showed a decreasing trend in 2020–2022 compared with that in 2019. Conversely, the reported prevalence of CCHD was higher than the 2019 estimate of 59.19 (56.76–61.71) per 100,000 in both 2021 [67.99 (65.34–70.71) per 100,000, OR = 1.12 (1.05–1.18)] and 2022 [66.13 (63.53–68.81) per 100,000, OR = 1.08 (1.02–1.14)], but with the increase having already started before the pandemic [55.71 (53.40–58.08) per 100,000 in 2016, OR = 0.89 (0.84–0.95) *versus* 2019].

**Figure 1:**
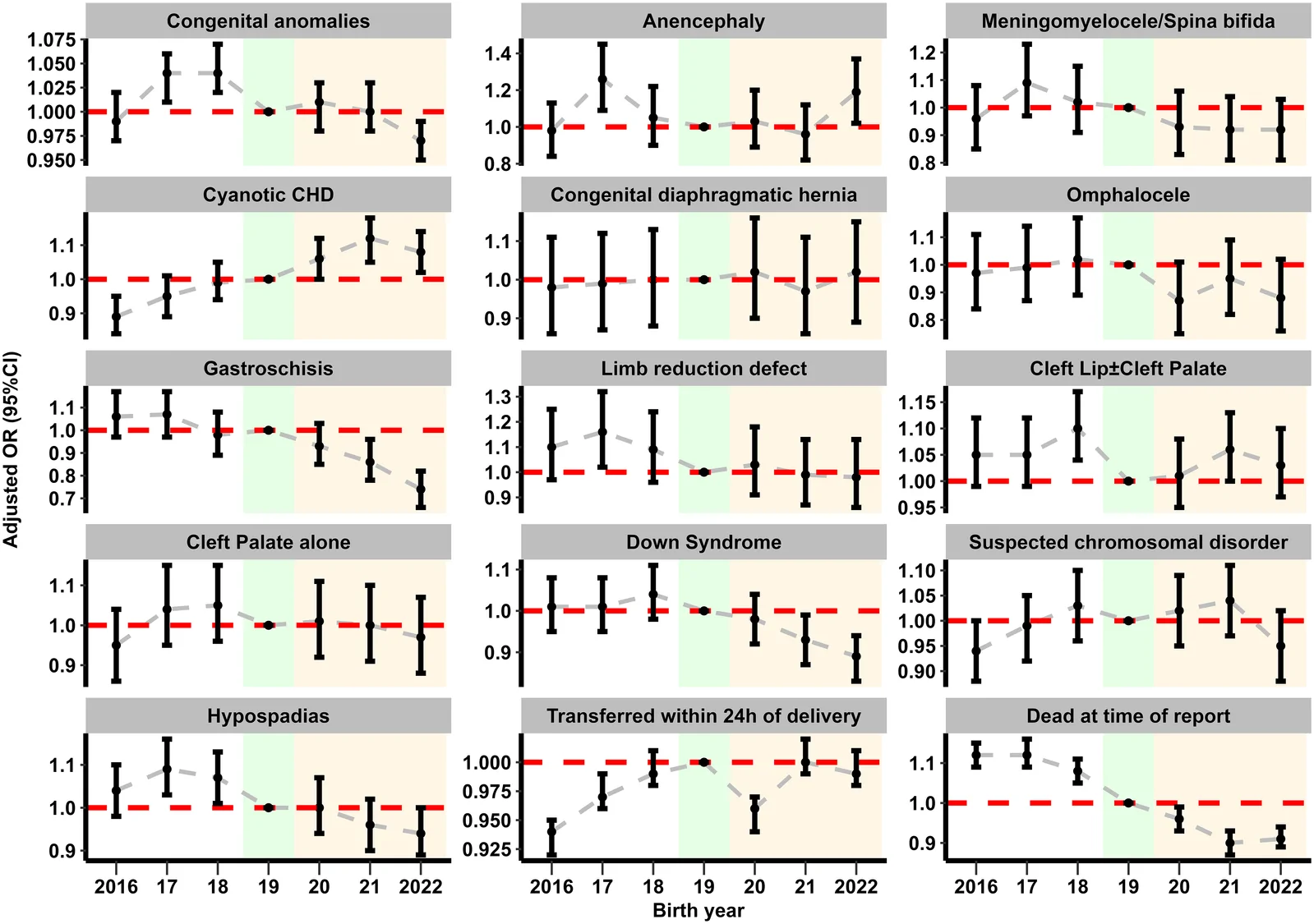
Adjusted odds ratios of the study outcomes by birth year. Details about each variable are presented in **Supplementary Methods**. Infants with missing outcomes were excluded from the analyses of the specific outcomes. The presented odds ratios were adjusted for maternal age, maternal residence, maternal ethnicity/race, maternal education attainment, principal source of payment for current delivery, and marital status. *OR*, odds ratio; *CI*, confidence interval; *CHD*, congenital heart disease.

These results were similar when stratifying by gestational age (≥37 or <37 weeks) (**Supplementary Figures S1** and **Supplementary Figures S2**), except that infants delivered at <37 weeks’ gestation had much higher reported prevalence of the study outcomes than those delivered at ≥37 weeks’ gestation (**Supplementary Tables S5** and **Supplementary Tables S6**). Gastroschisis replaced cleft lip with or without cleft palate as one of the five most frequently reported congenital anomalies in those delivered at <37 weeks’ gestation, and there was no difference in death before birth certificate registration from 2019 to 2022 in those delivered at ≥37 weeks’ gestation.

After correction for multiplicity within the prespecified family of 36 contrasts (**Supplementary Table S7**), seven contrasts were nominally significant, four survived Benjamini–Hochberg correction (CCHD in 2021, gastroschisis in 2021 and 2022, and Down syndrome in 2022), and only three survived Bonferroni correction: CCHD in 2021 (OR = 1.12, Bonferroni-adjusted CI = 1.02–1.23), gastroschisis in 2022 (OR = 0.74, CI = 0.62–0.88), and Down syndrome in 2022 (OR = 0.89, CI = 0.80–0.98). Notably, the CCHD estimate for 2022 did not survive correction (*q* = 0.061, Bonferroni-adjusted CI = 0.98–1.19). The results were materially unchanged in the extended family additionally including the pre-pandemic contrasts.

Regarding the other two study outcomes, the reported prevalence of being transferred within 24 h of delivery steadily increased since 2016, but reversed course and significantly decreased in 2020 [OR = 0.96 (0.94–0.97)], while those in 2021 and 2022 returned to the level in 2019. Death before birth certificate registration decreased continuously from 2016 [2.75 (2.70–2.80) per 1,000] to 2021 [2.16 (2.12–2.21) per 1,000] and was marginally higher in 2022 [2.22 (2.17–2.27) per 1,000] (**Supplementary Table S4**).

### Reported prevalence of CCHD between birth years after additional adjustment

When additionally adjusting for each covariate individually, including the first prenatal care visit, WIC participation, cigarette smoking during pregnancy, maternal BMI, pre-pregnancy diabetes, gestational diabetes, pre-pregnancy hypertension, gestational hypertension, pregnancy resulted from infertility treatment, fertility-enhancing drugs/artificial insemination/intrauterine insemination, assisted reproductive technology, infections present and/or treated during this pregnancy, plurality, and sex of the infant, the ORs of CCHD in 2020–2022 compared with that in 2019 remained essentially the same (**Supplementary Table S8**).

In the fully adjusted model, which entered all maternal and prenatal covariates simultaneously among the 26,302,211 infants with a non-missing CCHD item (15,971 events), the estimates were attenuated: OR = 1.05 (0.99–1.12, *p* = 0.078) in 2020, OR = 1.11 (1.04–1.17, *p* < 0.001) in 2021, and OR = 1.06 (1.00–1.12, *p* = 0.060) in 2022, so that only the 2021 contrast remained significant. Adding plurality and sex of the infant made no material difference. Collinearity did not threaten these contrasts: birth year was essentially orthogonal to the covariate set (adjusted generalized variance inflation factor, 1.00), and no term exceeded 1.16 (**Supplementary Table S9**).

### Sensitivity analyses restricted to singleton births and by confirmation status

Restricting the primary analysis to singleton births, in whom observations are independent by construction, left the estimates essentially unchanged [CCHD *versus* 2019: OR = 1.05 (0.99–1.12) in 2020, OR = 1.10 (1.04–1.17) in 2021, and OR = 1.08 (1.01–1.14) in 2022, with the composite outcome unchanged]. The decline in Down syndrome was present among records marked “confirmed” [2022 *versus* 2019: OR = 0.85 (0.77–0.93)] and, more weakly, among records marked “pending” [OR = 0.92 (0.84–1.00)] and is therefore not solely an artifact of the timing of karyotype confirmation (**Supplementary Table S10**).

### Sensitivity analysis re-indexed by estimated year of conception

When exposure was re-indexed by estimated year of conception (22,387,865 infants, 85.1% of live births with a non-missing composite outcome) (**Supplementary Table S11**), neither pandemic conception cohort differed in the reported prevalence of CCHD from the 2019 conception cohort [2020: 66.6 (63.9–69.3) per 100,000, OR = 1.03 (0.97–1.09); 2021: 67.0 (64.4–69.7) per 100,000, OR = 1.02 (0.97–1.08)], whereas all three pre-pandemic conception cohorts were significantly lower than that in 2019 [2016: OR = 0.85 (0.81–0.91); 2017: OR = 0.91 (0.86–0.97); 2018: OR = 0.92 (0.87–0.98)]. The composite outcome was likewise unchanged [2020: OR = 1.00 (0.97–1.02); 2021: OR = 0.97 (0.95–1.00)].

### Monthly reported prevalence and segmented regression

When the overall reported prevalence of congenital anomalies is further presented by calendar month within each birth year (**Figure 2**), similar monthly distributions were observed between the years: the earlier the birth month in a calendar year, the higher the overall reported prevalence of congenital anomalies at the population level.

**Figure 2:**
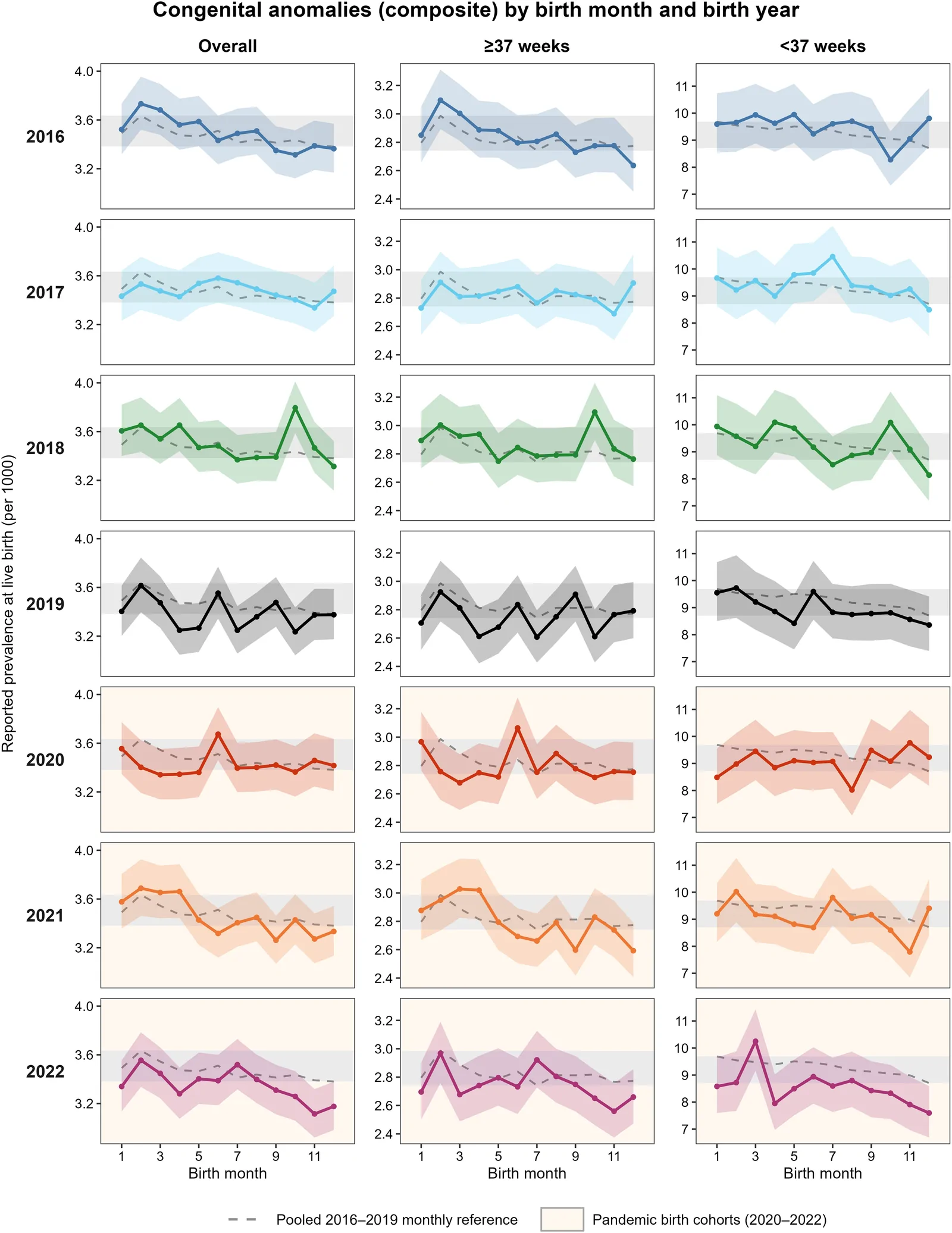
Reported prevalence of congenital anomalies by birth month and birth year. Each panel shows one birth year. Points and lines are the observed monthly birth certificate-reported prevalence at live birth (per 1,000 live births) with 95% Clopper–Pearson bands. The dashed gray line repeated in every panel is the pooled 2016–2019 monthly series, shown as a common reference, and the gray horizontal band spans its range. Panels for the pandemic birth cohorts (2020–2022) are shaded. Columns are the overall population and the subgroups delivered at ≥37 and <37 weeks’ gestation. The vertical scale is common within a column and independent between columns. Details about each variable are presented in **Supplementary Methods**. Infants with missing obstetric estimate of gestation (in the subgroups by obstetric estimate of gestation) or missing outcome were excluded from the analyses of specific outcomes.

For CCHD, the monthly reported prevalence was generally higher during the pandemic (especially in 2021 and 2022) than in infants born in the same calendar months of earlier calendar years (**Figure 3**). The interval of the highest single month [March 2021: 82 (72–93) per 100,000] overlapped those of 26 of the other 35 months of the 2020–2022 birth cohorts. The monthly distributions of the other study outcomes are presented in **Supplementary Figures S3**– **Supplementary Tables S15** and **Supplementary Tables S12**– **Supplementary Tables S14**.

**Figure 3:**
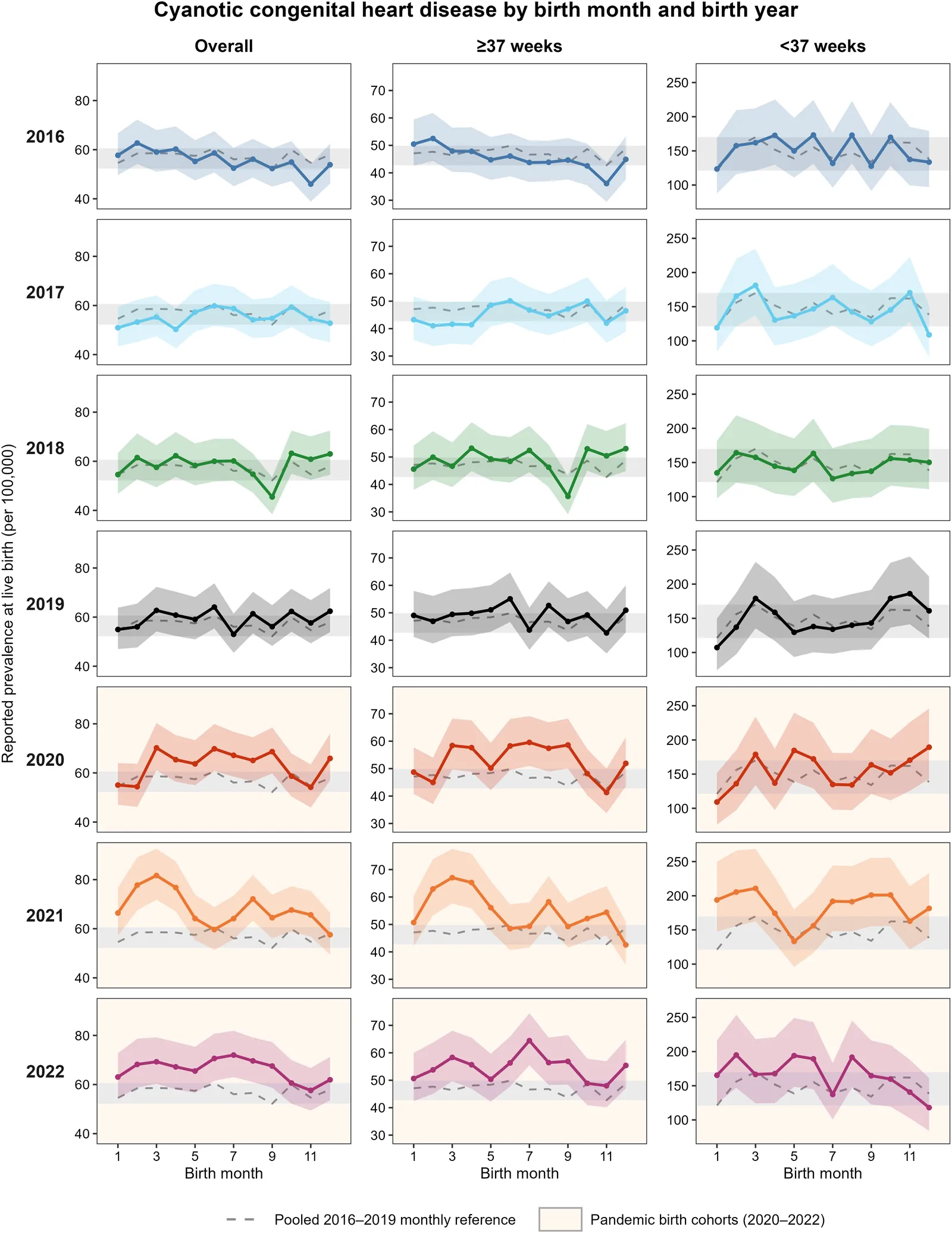
Reported prevalence of cyanotic congenital heart disease by birth month and birth year. Each panel shows one birth year. Points and lines are the observed monthly birth certificate-reported prevalence at live birth (per 100,000 live births) with 95% Clopper–Pearson bands. The dashed gray line repeated in every panel is the pooled 2016–2019 monthly series, shown as a common reference, and the gray horizontal band spans its range. Panels for the pandemic birth cohorts (2020–2022) are shaded. Columns are the overall population and the subgroups delivered at ≥37 and <37 weeks’ gestation. The vertical scale is common within a column and independent between columns. Details about each variable are presented in **Supplementary Methods**. Infants with missing obstetric estimate of gestation (in the subgroups by obstetric estimate of gestation) or missing outcome were excluded from the analysis of the specific outcome.

Segmented regression of the monthly series (**Supplementary Table S15** and **Supplementary Figure S16**) showed, for CCHD, a statistically significant upward trend already present before the pandemic (rate ratio of 1.0018 per month, 1.0004–1.0032, *p* = 0.014, which is equivalent to approximately 2.2% per year). There was, in addition, an abrupt level change at the March 2020 onset (1.11, 1.06–1.17, *p* < 0.001), with no subsequent slope change (0.9982, 0.9958–1.0007, *p* = 0.168). Among the other outcomes, gastroschisis and hypospadias showed significant pre-pandemic downward trends with no level change at either onset point, indicating continuation—and, for gastroschisis, acceleration—of a preexisting secular decline. Furthermore, the composite outcome showed no level change at March 2020 (1.0227, 0.9892–1.0574, *p* = 0.186). In the gestational age strata, the CCHD level change at March 2020 was present in both (≥37 weeks: 1.0981, 1.0397–1.1597, *p* < 0.001; <37 weeks: 1.1827, 1.0366–1.3495, *p* = 0.013), but with the pre-pandemic upward trend being confined to infants delivered at ≥37 weeks’ gestation (1.0023, 1.0006–1.0041, *p* = 0.009) and absent among those delivered at <37 weeks (0.9994, 0.9972–1.0016, *p* = 0.598).

## Discussion

In this cohort study, we used public birth data in the USA between 2016 and 2022 to examine trends in the birth certificate-reported prevalence of congenital anomalies at live birth. The main findings of the study are as follows:

1) The reported prevalence of congenital anomalies, both overall and for the majority of the specific types, did not increase during the pandemic, and several types showed a decreasing trend, of which the decreases in gastroschisis and Down syndrome survived correction for multiplicity.

2) The one outcome for which the reported prevalence was higher in the pandemic birth cohorts was CCHD, and the same direction was observed in infants delivered at both ≥37 and <37 weeks of gestation. However, the increase was already under way before the pandemic, and the abrupt level change was dated to infants born in March 2020, who had completed cardiac organogenesis during 2019.

3) When exposure was re-indexed by estimated year of conception, so that the first trimester rather than the birth was aligned with the pandemic, the contrast between the pandemic and the immediately preceding cohorts was null, and after correction for multiplicity and joint adjustment for the measured covariates, only the 2021 birth year contrast remained significant.

These findings, for the first time, describe the potential changes in the reported prevalence of congenital anomalies at live birth during the COVID-19 pandemic at the population level. Although the COVID-19 pandemic has resolved, considering the long-term consequences of congenital anomalies, it is still relevant to confirm whether the disease burden changed during the pandemic. Our principal finding is that, for the 12 items recorded on the US birth certificate, they did not. In addition to this implication, the trends in some of the maternal/perinatal characteristics may also attract attention. For example, we found the rates of overweight/obesity, diabetes, and hypertension (both chronic and gestational) to increase. Although these may be global issues (Alwash et al., 2023; Dzakpasu et al., 2024; Kent et al., 2024), they remain paths to further improving maternal/child health.

As summarized in the **Supplementary Material**, existing studies have mainly focused on a causal research question: whether maternal COVID-19 (or SARS-CoV-2 infection) or COVID-19 vaccination during pregnancy is causally associated with neonatal congenital anomalies. This research question is important, but is very challenging to answer with an observational study design. Conversely, we took one step back and tried to answer whether, at the population level, the reported prevalence of congenital anomalies at live birth had changed during the pandemic. This study design, which underscores descriptive epidemiology, is not suitable for determining causal inference. However, it may provide some new insights. If COVID-19 or COVID-19 vaccination did pose an additional risk of congenital anomalies, we should be able to observe an increase in the disease burden at the population level. Our null findings (i.e., no change, or even a decreasing trend) are consistent with several large-scale studies (Neelam et al., 2023; Kharbanda et al., 2024; Magnus et al., 2024). Although a few studies reported an increase in several types of birth defects (Hasan et al., 2023; Wang et al., 2023; Auger et al., 2024; Molnárová et al., 2024; Nassar et al., 2024; Qiu et al., 2024; Xu et al., 2024), we could not examine the same conditions for comparison because situs inversus, biliary atresia, and microcephaly are not among the items recorded on the US birth certificate.

The higher reported prevalence of CCHD in the pandemic birth cohorts should not be interpreted as evidence of a teratogenic effect, and three non-causal explanations are more parsimonious. The first is ascertainment. The anomaly items on the birth certificate are checkboxes with no diagnostic criteria, no imaging confirmation, and no ICD coding, and their sensitivity has repeatedly been shown to be low: validated against an active birth defects registry, the overall sensitivity of the anomaly indicators on the 2003 revision certificate was 19% (Salemi et al., 2017), and the medical and health items of the same certificate show similarly low sensitivity when compared with abstracted hospital records (Martin et al., 2013). Our own estimate is consistent with that: the pooled birth prevalence of the 12 critical congenital heart defects reported by the National Birth Defects Prevention Network from active case-finding programs is 19.93 per 10,000, i.e., 199 per 100,000 live births (Mai et al., 2019), whereas we report 61 per 100,000, approximately one third of it. The case definitions are not identical, but the gap is far larger than any plausible definitional difference, and with ascertainment this incomplete, a temporal signal is highly vulnerable to drift in the reporting process. Such drift is directly visible in our own data: missingness of the anomaly items rose from 0.13%–0.17% in 2016–2020 to 0.21% in 2021, and missingness of the transfer item rose monotonically from 0.06% in 2018 to 0.24% in 2022.

The second explanation is the maturation of detection. Newborn pulse oximetry screening for critical CHD was added to the recommended uniform screening panel in 2011 and had been adopted by every state by 2018 (Glidewell et al., 2019). Increasing detection, together with more frequent echocardiographic confirmation before the certificate is completed, would raise the apparent prevalence without any change in the true occurrence of disease and would generate precisely the pre-pandemic upward trend that the segmented regression identifies.

The third explanation runs in the opposite direction to teratogenesis and applies specifically to the pandemic years. Because only live births are included, anomalies ending in termination or stillbirth are excluded from our denominator and numerator alike. Prenatal detection of critical CHD fell during the pandemic (Maenchen et al., 2023; Gupta et al., 2024); fewer prenatally diagnosed cases means fewer terminations, and therefore more affected infants surviving to live birth. This mechanism would raise the reported prevalence of CCHD at live birth without any change in the underlying occurrence of the malformation, and it would equally explain a partial reversal as prenatal services recovered. We searched for a data source that would allow us to examine this directly and could not identify one: the NCHS fetal death file does not record prenatally diagnosed cardiac anomalies in terminations, CDC abortion surveillance does not record the indication, and no national birth defects registry provides pregnancy outcome ascertainment for 2016–2022. Triangulation with fetal diagnosis or termination data is therefore identified as a target for future work rather than something the present data can settle. A similar reasoning applies, in the opposite direction, to Down syndrome. The uptake of cell-free DNA (noninvasive prenatal) screening rose steeply in the USA over the study period (Palomaki et al., 2024), and a substantial share of prenatally diagnosed cases does not proceed to live birth (de Graaf et al., 2022). A secular decline in the live birth prevalence of Down syndrome is therefore expected independently of the pandemic. Our confirmation status analysis indicates that the decline we observed is not merely an artifact of the timing of karyotype confirmation, but does not distinguish a pandemic effect from this secular trend.

Finally, the timing of the observations argues against a teratogenic interpretation on purely biological grounds. Cardiac morphogenesis occurs at approximately 3–8 weeks post-conception, roughly 7–8 months before a term birth, and the recognized teratogenic window closes at approximately 14 weeks’ gestation (Ruderman et al., 2022). A level change dated to infants born in March 2020 therefore falls entirely outside any window in which maternal infection could have acted, and the conception-indexed analysis, the analysis that does align exposure with organogenesis, is null. We nevertheless note that there could have been other changes during the pandemic, including changes in maternal mental health (Ng et al., 2020; Cao et al., 2021; Sabbagh et al., 2023; Sabbagh et al., 2024), the epidemiology of other communicable diseases (Teutsch et al., 2021), medication treatment for COVID-19 (Giampreti et al., 2020; Louchet et al., 2020; Rizzi et al., 2020; Howley et al., 2021; Huybrechts et al., 2021), and social distancing (Nomden et al., 2022; Arshad et al., 2023; Nomden et al., 2023; Zhou et al., 2023), which themselves may have influenced the occurrence of congenital anomalies, and that potential measurement errors (Carmona et al., 2022; Zhang et al., 2024) may also have contributed to the observed trends. The CCHD observation should accordingly be regarded as hypothesis-generating and as a target for surveillance in data sources with an independent case definition, not as evidence of harm.

## Limitations

Several limitations should be noted. First, and most importantly, all data on congenital anomalies were collected from birth certificates without an independent case definition. The certificate requires only a checkbox, completed from the delivery record by the certifier, with no diagnostic criteria, no imaging or karyotype confirmation, and no ICD coding. Moreover, the reported prevalence we estimated is approximately one third of the pooled estimate from active case-finding surveillance for critical congenital heart defects (Mai et al., 2019). Our outcome is therefore the birth certificate-reported prevalence at live birth, and it should not be read as the birth prevalence of the conditions themselves. Because ascertainment is this incomplete, modest changes in the reporting behavior can produce temporal signals of the size we observed, and the rising missingness of the anomaly and transfer items over 2021–2022 showed that the reporting process did change during the study period.

Second, the data covered live births only. Anomalies ending in spontaneous abortion, stillbirth, or termination after prenatal diagnosis were not captured. This limits generalizability to all pregnancy outcomes and, as discussed above, is also a source of directional bias: a pandemic-era reduction in prenatal detection and termination would raise the reported prevalence of severe anomalies at live birth in the absence of any change in their occurrence.

Third, the outcome set is neither exhaustive nor a random sample of congenital anomalies. It is restricted to the 12 items listed on the US standard certificate of live birth, and several conditions reported by others to have increased during the pandemic—including situs inversus, biliary atresia, and microcephaly—cannot be examined in these data at all. Our conclusions cannot be generalized beyond the 12 items among live births.

Fourth, exposure in the primary analysis was indexed by calendar year of birth, which was offset from the period of organogenesis by approximately 9 months. We addressed this with the conception-indexed analysis. However, that analysis has limitations of its own: the obstetric estimate of gestation is imprecise, NCHS does not release the exact day of birth, thus births being placed at the middle of the reported month, and the conception cohorts are complete only for 2016–2021, and therefore 14.9% of the infants could not be assigned to a complete conception cohort.

Fifth, no variable identifies infants belonging to the same multiple pregnancy, and thus the non-independence of observations within multiple births could not be modeled. The resulting standard errors were, if anything, slightly too narrow. Restricting the analysis to singleton births, in whom the observations were independent by construction, left the estimates essentially unchanged; we therefore do not think that this materially affects the conclusions.

Sixth, the mortality item we analyzed captured only deaths recorded before the birth certificate was filed and therefore missed late neonatal, post-neonatal, and post-discharge deaths. It was also administratively determined; hence, registration timing disruptions during the pandemic could change the fraction captured for non-biological reasons. The NCHS-linked birth–infant death files, with follow-up of up to 1 year, would be the appropriate mortality anchor. Extending the study in that way is beyond the scope of this analysis and is identified as future work. Similarly, no follow-up data were used, and the time trends we observed may differ from those in studies using follow-up data, simply because not all congenital anomalies can be recognized at delivery.

Lastly, the study is descriptive. No individual-level data on maternal SARS-CoV-2 infection or COVID-19 vaccination are available in these files, and the design cannot support causal inference about either.

In conclusion, the birth certificate-reported prevalence of congenital anomalies at live birth in the USA did not increase during the COVID-19 pandemic and decreased for several specific anomalies. The higher reported prevalence of CCHD in the pandemic birth cohorts continues a trend that began before the pandemic and is null once exposure is aligned with the period of organogenesis. It is more parsimoniously explained by incomplete and changing ascertainment, by the maturation of newborn screening, and by reduced prenatal detection and termination than by any effect of the pandemic on fetal development. It warrants monitoring in data sources with an independent case definition rather than a causal interpretation.

## Data Availability

The original contributions presented in the study are included in the article/**Supplementary Material**. Further inquiries can be directed to the corresponding authors.
