# Supplementary material for: Changes in birth certificate-reported prevalence of congenital anomalies at live birth during the COVID-19 pandemic: a nationwide study of 26 million United States live births
Source: Front Cell Infect Microbiol. 2026 Sep 29;16:1914021. doi: 10.3389/fcimb.2026.1914021 (PMC13658458; doi:10.3389/fcimb.2026.1914021)
Supplement: Supplementary file 1 [file DataSheet1.pdf]

Supplementary material

Supplementary information: Search strategy (PubMed) & Summary of the evidence		2
Supplementary methods: Variables used in the study		10
Supplemental Table S1	Operational definitions of the birth-certificate items used as study outcomes	13
Supplemental Table S2	Missingness of the study outcomes by birth year	15
Supplemental Table S3	Other maternal and perinatal characteristics of the included infants by birth year	17
Supplemental Table S4	Reported prevalence of the study outcomes by birth year	23
Supplemental Table S5	Reported prevalence of the study outcomes by birth year (subgroup analysis, only infants delivered at ≥37 weeks' gestation)	26
Supplemental Table S6	Reported prevalence of the study outcomes by birth year (subgroup analysis, only infants delivered at <37 weeks' gestation)	29
Supplemental Table S7	Multiplicity-corrected results for the pre-specified family of contrasts	32
Supplemental Table S8	Reported prevalence of cyanotic congenital heart disease by birth year with additional adjustment	35
Supplemental Table S9	Fully adjusted models for cyanotic congenital heart disease, and collinearity diagnostics	37
Supplemental Table S10	Singleton-restricted and confirmation-status sensitivity analyses	39
Supplemental Table S11	Sensitivity analysis re-indexed by estimated year of conception	41
Supplemental Table S12	Reported prevalence of the study outcomes by birth month	44
Supplemental Table S13	Reported prevalence of the study outcomes by birth month (subgroup analysis, only infants delivered at ≥37 weeks' gestation)	62
Supplemental Table S14	Reported prevalence of the study outcomes by birth month (subgroup analysis, only infants delivered at <37 weeks' gestation)	80
Supplemental Table S15	Segmented regression (interrupted time-series) of the monthly series, 2016-2022	98
Supplemental Figure S1	Reported prevalence of the study outcomes by birth year (subgroup analysis, only infants delivered at ≥37 weeks' gestation)	101
Supplemental Figure S2	Reported prevalence of the study outcomes by birth year (subgroup analysis, only infants delivered at <37 weeks' gestation)	102
Supplemental Figure S3	Reported prevalence of anencephaly by birth month	103
Supplemental Figure S4	Reported prevalence of meningomyelocele/spina bifida by birth month	104
Supplemental Figure S5	Reported prevalence of congenital diaphragmatic hernia by birth month	105
Supplemental Figure S6	Reported prevalence of omphalocele by birth month	106
Supplemental Figure S7	Reported prevalence of gastroschisis by birth month	107
Supplemental Figure S8	Reported prevalence of limb reduction defect by birth month	108
Supplemental Figure S9	Reported prevalence of cleft lip with or without cleft palate by birth month	109
Supplemental Figure S10	Reported prevalence of cleft palate alone by birth month	110
Supplemental Figure S11	Reported prevalence of Down syndrome by birth month	111
Supplemental Figure S12	Reported prevalence of suspected chromosomal disorder by birth month	112
Supplemental Figure S13	Reported prevalence of hypospadias by birth month	113
Supplemental Figure S14	Reported prevalence of transfer within 24 hours of delivery by birth month	114
Supplemental Figure S15	Reported prevalence of death before birth-certificate registration by birth month	115
Supplemental Figure S16	Segmented regression of the monthly reported prevalence of cyanotic congenital heart disease, 2016-2022	116

Supplementary information
The supplementary information presents available evidence about potential effect of COVID-19 infection/vaccination on risk of congenital anomalies in newborns.

1.1 Search strategy (PubMed)

#1 Congenital abnormalities

"Congenital Abnormalities"[Mesh] OR "Congenital Abnormalities"[tiab] OR "Abnormality, Congenital"[tiab] OR "Congenital Abnormality"[tiab] OR "Abnormalities, Congenital"[tiab] OR "Birth Defects"[tiab] OR "Birth Defect"[tiab] OR "Defect, Birth"[tiab] OR "Congenital Defects"[tiab] OR "Congenital Defect"[tiab] OR "Defect, Congenital"[tiab] OR "Defects, Congenital"[tiab] OR "Deformities"[tiab] OR "Deformity"[tiab] OR "Fetal Anomalies"[tiab] OR "Anomaly, Fetal"[tiab] OR "Fetal Anomaly"[tiab] OR "Fetal Malformations"[tiab] OR "Fetal Malformation"[tiab] OR "Malformation, Fetal"[tiab] OR "Abnormalities, Drug-Induced"[Mesh] OR "Abnormalities, Multiple"[Mesh] OR "22q11 Deletion Syndrome"[Mesh] OR "Alagille Syndrome"[Mesh] OR "Angelman Syndrome"[Mesh] OR "Barth Syndrome"[Mesh] OR "Basal Cell Nevus Syndrome"[Mesh] OR "Beckwith-Wiedemann Syndrome"[Mesh] OR "Bloom Syndrome"[Mesh] OR "Branchio-Oto-Renal Syndrome"[Mesh] OR "Carney Complex"[Mesh] OR "Ciliopathies"[Mesh] OR "Cockayne Syndrome"[Mesh] OR "Costello Syndrome"[Mesh] OR "Cri-du-Chat Syndrome"[Mesh] OR "De Lange Syndrome"[Mesh] OR "Deaf-Blind Disorders"[Mesh] OR "Donohue Syndrome"[Mesh] OR "Down syndrome"[Mesh] OR "Ectodermal Dysplasia"[Mesh] OR "Fraser Syndrome"[Mesh] OR "Gardner Syndrome"[Mesh] OR "Heterotaxy Syndrome"[Mesh] OR "Holoprosencephaly"[Mesh] OR "Incontinentia Pigmenti"[Mesh] OR "Isolated Noncompaction of the Ventricular Myocardium"[Mesh] OR "Laurence-Moon Syndrome"[Mesh] OR "LEOPARD Syndrome"[Mesh] OR "Loeys-Dietz Syndrome"[Mesh] OR "Marfan Syndrome"[Mesh] OR "Mobius Syndrome"[Mesh] OR "Monilethrix"[Mesh] OR "Nail-Patella Syndrome"[Mesh] OR "Netherton Syndrome"[Mesh] OR "Nevus, Sebaceous of Jadassohn"[Mesh] OR "Oculocerebrorenal Syndrome"[Mesh] OR "Orofaciodigital Syndromes"[Mesh] OR "Pallister-Hall Syndrome"[Mesh] OR "Pentalogy of Cantrell"[Mesh] OR "POEMS Syndrome"[Mesh] OR "Polycystic Kidney Diseases"[Mesh] OR "Prader-Willi Syndrome"[Mesh] OR "Prolidase Deficiency"[Mesh] OR "Proteus Syndrome"[Mesh] OR "Prune Belly Syndrome"[Mesh] OR "Rubella Syndrome, Congenital"[Mesh] OR "Rubinstein-Taybi Syndrome"[Mesh] OR "Short Rib-Polydactyly Syndrome"[Mesh] OR "Silver-Russell Syndrome"[Mesh] OR "Smith-Lemli-Opitz Syndrome"[Mesh] OR "Smith-Magenis Syndrome"[Mesh] OR "Sotos Syndrome"[Mesh] OR "Trichothiodystrophy Syndromes"[Mesh] OR "Trisomy 13 Syndrome"[Mesh] OR "Trisomy 18 Syndrome"[Mesh] OR "Waardenburg Syndrome"[Mesh] OR "Weill-Marchesani Syndrome"[Mesh] OR "Wolf-Hirschhorn Syndrome"[Mesh] OR "Zellweger Syndrome"[Mesh] OR "Abnormalities, Radiation-Induced"[Mesh] OR "Abnormalities, Severe Teratoid"[Mesh] OR "Anencephaly"[Mesh] OR "Twins, Conjoined"[Mesh] OR "Aicardi Syndrome"[Mesh] OR "Cardiovascular Abnormalities"[Mesh] OR "Heart Defects, Congenital"[Mesh] OR "Vascular Malformations"[Mesh] OR "Chromosome Disorders"[Mesh] OR "22q11 Deletion Syndrome"[Mesh] OR "Angelman Syndrome"[Mesh] OR "Beckwith-Wiedemann Syndrome"[Mesh] OR "Branchio-Oto-Renal Syndrome"[Mesh] OR "Cri-du-Chat Syndrome"[Mesh] OR "De Lange Syndrome"[Mesh] OR "Down syndrome"[Mesh] OR "Holoprosencephaly"[Mesh] OR "Jacobsen Distal 11q Deletion Syndrome"[Mesh] OR "Prader-Willi Syndrome"[Mesh] OR "Rubinstein-Taybi Syndrome"[Mesh] OR "Sex Chromosome Disorders"[Mesh] OR "Silver-Russell Syndrome"[Mesh] OR "Smith-Magenis Syndrome"[Mesh] OR "Sotos Syndrome"[Mesh] OR "Trisomy 13 Syndrome"[Mesh] OR "Trisomy 18 Syndrome"[Mesh] OR "WAGR Syndrome"[Mesh] OR "Williams Syndrome"[Mesh] OR "Wolf-Hirschhorn Syndrome"[Mesh] OR "Congenital Microtia"[Mesh] OR "Digestive System Abnormalities"[Mesh] OR "Anorectal Malformations"[Mesh] OR "Anus, Imperforate"[Mesh] OR "Biliary Atresia"[Mesh] OR "Choledochal Cyst"[Mesh] OR "Diaphragmatic Eventration"[Mesh] OR "Esophageal Atresia"[Mesh] OR "Hirschsprung Disease"[Mesh] OR "Intestinal Atresia"[Mesh] OR "Meckel Diverticulum"[Mesh] OR "Pancreas Divisum"[Mesh] OR "Pancreaticobiliary Maljunction"[Mesh] OR "Eye Abnormalities"[Mesh] OR "Aniridia"[Mesh] OR "Anophthalmos"[Mesh] OR "Blepharophimosis"[Mesh] OR "Coloboma"[Mesh] OR "Ectopia Lentis"[Mesh] OR "Familial Exudative Vitreoretinopathies"[Mesh] OR "Fraser Syndrome"[Mesh] OR "Hydrophthalmos"[Mesh] OR "Microphthalmos"[Mesh] OR "Persistent Hyperplastic Primary Vitreous"[Mesh] OR "Retinal Dysplasia"[Mesh] OR "Hernias, Diaphragmatic, Congenital"[Mesh] OR "Lymphatic Abnormalities"[Mesh] OR "22q11 Deletion Syndrome"[Mesh] OR "Lymphangiectasis, Intestinal"[Mesh] OR "Musculoskeletal Abnormalities"[Mesh] OR "Arthrogryposis"[Mesh] OR "Campomelic Dysplasia"[Mesh] OR "Cervical Rib Syndrome"[Mesh] OR "Craniofacial Abnormalities"[Mesh] OR "Developmental Dysplasia of the Hip"[Mesh] OR "Funnel Chest"[Mesh] OR "Gastroschisis"[Mesh] OR "Hajdu-Cheney Syndrome"[Mesh] OR "Hip Dislocation, Congenital"[Mesh] OR "Klippel-Feil Syndrome"[Mesh] OR "Laryngomalacia"[Mesh] OR "Limb Deformities, Congenital"[Mesh] OR "Pectus Carinatum"[Mesh] OR "Synostosis"[Mesh] OR "Tracheobronchomalacia"[Mesh] OR "Nervous System Malformations"[Mesh] OR "Agenesis of Corpus Callosum"[Mesh] OR "Central Nervous System Cysts"[Mesh] OR "Central Nervous System Vascular Malformations"[Mesh] OR "Congenital Cranial Dysinnervation Disorders"[Mesh] OR "Dandy-Walker Syndrome"[Mesh] OR "Hereditary Sensory and Autonomic Neuropathies"[Mesh] OR "Hereditary Sensory and Motor Neuropathy"[Mesh] OR "Hydranencephaly"[Mesh] OR "Malformations of Cortical Development"[Mesh] OR "Neural Tube Defects"[Mesh] OR "Optic Nerve Hypoplasia"[Mesh] OR "Respiratory System Abnormalities"[Mesh] OR "Bronchogenic Cyst"[Mesh] OR "Bronchopulmonary Sequestration"[Mesh] OR "Choanal Atresia"[Mesh] OR "Cystic Adenomatoid Malformation of Lung, Congenital"[Mesh] OR "Kartagener Syndrome"[Mesh] OR "Laryngocele"[Mesh] OR "Laryngostenosis"[Mesh] OR "Scimitar Syndrome"[Mesh] OR "Tracheobronchomegaly"[Mesh] OR "Situs Inversus"[Mesh] OR "Dextrocardia"[Mesh] OR "Levocardia"[Mesh] OR "Skin Abnormalities"[Mesh] OR "Acrodermatitis"[Mesh] OR "Carney Complex"[Mesh] OR "Dyskeratosis Congenita"[Mesh] OR "Ectodermal Dysplasia"[Mesh] OR "Ehlers-Danlos Syndrome"[Mesh] OR "Epidermolysis Bullosa"[Mesh] OR "Ichthyosis"[Mesh] OR "Incontinentia Pigmenti"[Mesh] OR "Port-Wine Stain"[Mesh] OR "Prolidase Deficiency"[Mesh] OR "Pseudoxanthoma Elasticum"[Mesh] OR "Rothmund-Thomson Syndrome"[Mesh] OR "Sclerema Neonatorum"[Mesh] OR "Trichothiodystrophy Syndromes"[Mesh] OR "Xeroderma Pigmentosum"[Mesh] OR "Stomatognathic System Abnormalities"[Mesh] OR "Maxillofacial Abnormalities"[Mesh] OR "Mouth Abnormalities"[Mesh] OR "Tooth Abnormalities"[Mesh] OR "Thyroid Dysgenesis"[Mesh] OR "Lingual Thyroid"[Mesh] OR "Urogenital Abnormalities"[Mesh] OR "Bladder Exstrophy"[Mesh] OR "Cryptorchidism"[Mesh] OR "Disorders of Sex Development"[Mesh] OR "Epispadias"[Mesh] OR "Fraser Syndrome"[Mesh] OR "Fused Kidney"[Mesh] OR "Hypospadias"[Mesh] OR "Multicystic Dysplastic Kidney"[Mesh] OR "Nephritis, Hereditary"[Mesh] OR "Pyelectasis"[Mesh] OR "Retrocaval Ureter"[Mesh] OR "Uterine Duplication Anomalies"[Mesh] OR "Abnormalities, Drug-Induced"[tiab] OR "Abnormalities, Multiple"[tiab] OR "22q11 Deletion Syndrome"[tiab] OR "Alagille Syndrome"[tiab] OR "Angelman Syndrome"[tiab] OR "Barth Syndrome"[tiab] OR "Basal Cell Nevus Syndrome"[tiab] OR "Beckwith-Wiedemann Syndrome"[tiab] OR "Bloom Syndrome"[tiab] OR "Branchio-Oto-Renal Syndrome"[tiab] OR "Carney Complex"[tiab] OR "Ciliopathies"[tiab] OR "Cockayne Syndrome"[tiab] OR "Costello Syndrome"[tiab] OR "Cri-du-Chat Syndrome"[tiab] OR "De Lange Syndrome"[tiab] OR "Deaf-Blind Disorders"[tiab] OR "Donohue Syndrome"[tiab] OR "Down syndrome"[tiab] OR "Ectodermal Dysplasia"[tiab] OR "Fraser Syndrome"[tiab] OR "Gardner Syndrome"[tiab] OR "Heterotaxy Syndrome"[tiab] OR "Holoprosencephaly"[tiab] OR "Incontinentia Pigmenti"[tiab] OR "Isolated Noncompaction of the Ventricular Myocardium"[tiab] OR "Laurence-Moon Syndrome"[tiab] OR "LEOPARD Syndrome"[tiab] OR "Loeys-Dietz Syndrome"[tiab] OR "Marfan Syndrome"[tiab] OR "Mobius Syndrome"[tiab] OR "Monilethrix"[tiab] OR "Nail-Patella Syndrome"[tiab] OR "Netherton Syndrome"[tiab] OR "Nevus, Sebaceous of Jadassohn"[tiab] OR "Oculocerebrorenal Syndrome"[tiab] OR

"Orofaciodigital Syndromes"[tiab] OR "Pallister-Hall Syndrome"[tiab] OR "Pentalogy of Cantrell"[tiab] OR "POEMS Syndrome"[tiab] OR "Polycystic Kidney Diseases"[tiab] OR "Prader-Willi Syndrome"[tiab] OR "Prolidase Deficiency"[tiab] OR "Proteus Syndrome"[tiab] OR "Prune Belly Syndrome"[tiab] OR "Rubella Syndrome, Congenital"[tiab] OR "Rubinstein-Taybi Syndrome"[tiab] OR "Short Rib-Polydactyly Syndrome"[tiab] OR "Silver-Russell Syndrome"[tiab] OR "Smith-Lemli-Opitz Syndrome"[tiab] OR "Smith-Magenis Syndrome"[tiab] OR "Sotos Syndrome"[tiab] OR "Trichothiodystrophy Syndromes"[tiab] OR "Trisomy 13 Syndrome"[tiab] OR "Trisomy 18 Syndrome"[tiab] OR "Waardenburg Syndrome"[tiab] OR "Weill-Marchesani Syndrome"[tiab] OR "Wolf-Hirschhorn Syndrome"[tiab] OR "Zellweger Syndrome"[tiab] OR "Abnormalities, Radiation-Induced"[tiab] OR "Abnormalities, Severe Teratoid"[tiab] OR "Anencephaly"[tiab] OR "Twins, Conjoined"[tiab] OR "Aicardi Syndrome"[tiab] OR "Cardiovascular Abnormalities"[tiab] OR "Heart Defects, Congenital"[tiab] OR "Vascular Malformations"[tiab] OR "Chromosome Disorders"[tiab] OR "22q11 Deletion Syndrome"[tiab] OR "Angelman Syndrome"[tiab] OR "Beckwith-Wiedemann Syndrome"[tiab] OR "Branchio-Oto-Renal Syndrome"[tiab] OR "Cri-du-Chat Syndrome"[tiab] OR "De Lange Syndrome"[tiab] OR "Down syndrome"[tiab] OR "Holoprosencephaly"[tiab] OR "Jacobsen Distal 11q Deletion Syndrome"[tiab] OR "Prader-Willi Syndrome"[tiab] OR "Rubinstein-Taybi Syndrome"[tiab] OR "Sex Chromosome Disorders"[tiab] OR "Silver-Russell Syndrome"[tiab] OR "Smith-Magenis Syndrome"[tiab] OR "Sotos Syndrome"[tiab] OR "Trisomy 13 Syndrome"[tiab] OR "Trisomy 18 Syndrome"[tiab] OR "WAGR Syndrome"[tiab] OR "Williams Syndrome"[tiab] OR "Wolf-Hirschhorn Syndrome"[tiab] OR "Congenital Microtia"[tiab] OR "Digestive System Abnormalities"[tiab] OR "Anorectal Malformations"[tiab] OR "Anus, Imperforate"[tiab] OR "Biliary Atresia"[tiab] OR "Choledochal Cyst"[tiab] OR "Diaphragmatic Eventration"[tiab] OR "Esophageal Atresia"[tiab] OR "Hirschsprung Disease"[tiab] OR "Intestinal Atresia"[tiab] OR "Meckel Diverticulum"[tiab] OR "Pancreas Divisum"[tiab] OR "Pancreaticobiliary Maljunction"[tiab] OR "Eye Abnormalities"[tiab] OR "Aniridia"[tiab] OR "Anophthalmos"[tiab] OR "Blepharophimosis"[tiab] OR "Coloboma"[tiab] OR "Ectopia Lentis"[tiab] OR "Familial Exudative Vitreoretinopathies"[tiab] OR "Fraser Syndrome"[tiab] OR "Hydrophthalmos"[tiab] OR "Microphthalmos"[tiab] OR "Persistent Hyperplastic Primary Vitreous"[tiab] OR "Retinal Dysplasia"[tiab] OR "Hernias, Diaphragmatic, Congenital"[tiab] OR "Lymphatic Abnormalities"[tiab] OR "22q11 Deletion Syndrome"[tiab] OR "Lymphangiectasis, Intestinal"[tiab] OR "Musculoskeletal Abnormalities"[tiab] OR "Arthrogryposis"[tiab] OR "Campomelic Dysplasia"[tiab] OR "Cervical Rib Syndrome"[tiab] OR "Craniofacial Abnormalities"[tiab] OR "Developmental Dysplasia of the Hip"[tiab] OR "Funnel Chest"[tiab] OR "Gastroschisis"[tiab] OR "Hajdu-Cheney Syndrome"[tiab] OR "Hip Dislocation, Congenital"[tiab] OR "Klippel-Feil Syndrome"[tiab] OR "Laryngomalacia"[tiab] OR "Limb Deformities, Congenital"[tiab] OR "Pectus Carinatum"[tiab] OR "Synostosis"[tiab] OR "Tracheobronchomalacia"[tiab] OR "Nervous System Malformations"[tiab] OR "Agenesis of Corpus Callosum"[tiab] OR "Central Nervous System Cysts"[tiab] OR "Central Nervous System Vascular Malformations"[tiab] OR "Congenital Cranial Dysinnervation Disorders"[tiab] OR "Dandy-Walker Syndrome"[tiab] OR "Hereditary Sensory and Autonomic Neuropathies"[tiab] OR "Hereditary Sensory and Motor Neuropathy"[tiab] OR "Hydranencephaly"[tiab] OR "Malformations of Cortical Development"[tiab] OR "Neural Tube Defects"[tiab] OR "Optic Nerve Hypoplasia"[tiab] OR "Respiratory System Abnormalities"[tiab] OR "Bronchogenic Cyst"[tiab] OR "Bronchopulmonary Sequestration"[tiab] OR "Choanal Atresia"[tiab] OR "Cystic Adenomatoid Malformation of Lung, Congenital"[tiab] OR "Kartagener Syndrome"[tiab] OR "Laryngocele"[tiab] OR "Laryngostenosis"[tiab] OR "Scimitar Syndrome"[tiab] OR "Tracheobronchomegaly"[tiab] OR "Situs Inversus"[tiab] OR "Dextrocardia"[tiab] OR "Levocardia"[tiab] OR "Skin Abnormalities"[tiab] OR "Acrodermatitis"[tiab] OR "Carney Complex"[tiab] OR "Dyskeratosis Congenita"[tiab] OR "Ectodermal Dysplasia"[tiab] OR "Ehlers-Danlos Syndrome"[tiab] OR "Epidermolysis Bullosa"[tiab] OR "Ichthyosis"[tiab] OR "Incontinentia Pigmenti"[tiab] OR "Port-Wine Stain"[tiab] OR "Prolidase Deficiency"[tiab] OR "Pseudoxanthoma Elasticum"[tiab] OR "Rothmund-Thomson Syndrome"[tiab] OR "Sclerema Neonatorum"[tiab] OR "Trichothiodystrophy Syndromes"[tiab] OR "Xeroderma Pigmentosum"[tiab] OR "Stomatognathic System Abnormalities"[tiab] OR "Maxillofacial Abnormalities"[tiab] OR "Mouth Abnormalities"[tiab] OR "Tooth Abnormalities"[tiab] OR "Thyroid Dysgenesis"[tiab] OR "Lingual Thyroid"[tiab] OR "Urogenital Abnormalities"[tiab] OR "Bladder Exstrophy"[tiab] OR "Cryptorchidism"[tiab] OR "Disorders of Sex Development"[tiab] OR "Epispadias"[tiab] OR "Fraser Syndrome"[tiab] OR "Fused Kidney"[tiab] OR "Hypospadias"[tiab] OR "Multicystic Dysplastic Kidney"[tiab] OR "Nephritis, Hereditary"[tiab] OR "Pyelectasis"[tiab] OR "Retrocaval Ureter"[tiab] OR "Uterine Duplication Anomalies"[tiab]

#2 COVID-19

("COVID-19"[Mesh] OR "SARS-CoV-2"[Mesh] OR "COVID-19 Testing"[Mesh] OR 2019-nCoV[All Fields] OR 2019nCoV[All Fields] OR COVID-19[All Fields] OR COVID-2019[All Fields] OR COVID-19s[All Fields] OR COVID[TiAb] OR COVID*[Ti] OR COVID19[All Fields] OR CoV-19[All Fields] OR SARS-CoV-2[All Fields] OR SARS-COV2[All Fields] OR "severe acute respiratory syndrome coronavirus 2"[All Fields] OR "COVID-19 Vaccines"[Mesh] OR "Coronavirus Disease 2019"[All Fields] OR (Coronavirus*[All Fields] AND pandemic[All Fields]) OR "COVID-19 Serological Testing"[Mesh] OR "COVID-19 Nucleic Acid Testing"[Mesh] OR "SARS-CoV-2 variants" [Supplementary Concept] OR "spike protein, SARS-CoV-2" [Supplementary Concept] OR "COVID-19 serotherapy" [Supplementary Concept] OR "COVID-19 breakthrough infections" [Supplementary Concept] OR "COVID-19 drug treatment" [Supplementary Concept] OR "SARS-CoV-2 inactivated vaccines" [Supplementary Concept] OR "COVID-19 stress syndrome" [Supplementary Concept] OR "COVID-19 post-intensive care syndrome" [Supplementary Concept] OR "post-acute COVID-19 syndrome" [Supplementary Concept]) AND 2019:3000[pdat]

#3 Animal, not Human

"Animals"[Mesh] NOT ("Animals"[Mesh] AND "Humans"[Mesh])

#4

(#1 AND #2) NOT #3

The search conducted on 2024-12-02 returned 1481 results, which were used for further screening.

1.2 Summary of the evidence

Reference	First author	Publication year	Study period	Study region	Study design	Sample size	Main findings
**Potential effects of SARS-CoV-2 infection/COVID-19**							
1	Morhart	2022	2021	Germany	Case series	27	Newborns whose mothers were pregnant when exposed to the SARS-CoV-2: a severe eye malformation (unilateral microphthalmia, optic nerve hypoplasia, and congenital retinopathy) associated with maternal SARS-CoV-2 infection in weeks 5 and 6 of embryonic development
2	Dosedla	2023	?	Slovakia?	Case report	1	A pregnant woman who had COVID-19 infection in the 7th month of pregnancy gave birth to an infant with epidermolysis.
3	Batinović	2023	?	Croatia	Case report	1	A pregnant woman who had SARS-CoV-2 infection during the early stage of pregnancy gave birth to an infant with double aneuploidy [Klinefelter and Edwards Syndrome (48, XXY, +18)]
4	Abdelkader	2022	?	Egypt	Case report	1	A pregnant woman who had COVID-19 infection in the first month of pregnancy gave birth to an infant with cyanotic congenital heart disease.
5	Jiang	2022	?	China	Case series	24	Among neonates born to SARS-CoV-2 infected pregnant women: one infant had ulcerous lesions on the scalp vertex at birth.
6	Goldshtrom	2020	2020	US	Case series	7	Neonates with prenatal diagnosis of complex congenital malformations of the heart or lung born to mothers who were diagnosed with COVID-19:<br>• Clinical course was as expected for the congenital lesion.<br>• Neither vertical nor horizontal transmission of SARS-CoV-2 was noted.
7	Dalla	2021	?	US	Case report	1	An infant was diagnosed with tetralogy of Fallot despite normal prenatal sonograms from 12 to 38 weeks' gestation. The mother had COVID-19 history at 18 weeks (i.e., second-trimester).
8	Okido	2023	?	Brazil	Case report	1	An infant was diagnosed with a severe phenotype of Adams-Oliver Syndrome. The infant's mother had SARS-CoV-2 infection in the first trimester. Prenatal ultrasound indicates a probable worsening of the disease after the first trimester.
9	Chimenea	2023	2021	Spain	Case report	1	A pregnant woman with early SARS-CoV-2 infection was carrying a fetus prenatally diagnosed with VACTERL association through a mid-trimester ultrasound.
10	Erciyestepe	2024	?	Turkey	Case report	1	Ultrasonography in the first trimester revealed right forearm aplasia and deformities of both feet and legs in a fetus whose mother was hospitalized with COVID-19 two days before the onset of her last menstrual period.
11	Cosma	2021	2020	Italy	Cohort study	147 vs 17	Pregnant women admitted for 12-week noninvasive prenatal testing without vs with SARS-CoV-2 infection: No difference in fetal incidence of structural anomalies at week 20
12	Cosma	2022	2020	Italy	Cohort study	16+105	Neonates born to mothers who were diagnosed with COVID-19 in the first trimester vs COVID-19 negative: Similar congenital birth defect (6.3% vs 6.7%)
13	Ludvigsson	2024	2018-2022	Norway, Denmark, Sweden	Cohort study	Not reported	There was no major increase in the incidence of foetal situs inversus during the COVID-19 pandemic.
14	Hofbauer	2024	2020-2023	Germany	Cohort study	7142	The overall prevalence of congenital malformations (1.96%) was comparable to that of pre-pandemic data (2.39%).
15	Molnárová	2024	2021-2023	Slovakia	Case-control study	2729	Maternal COVID-19 is associated with non-syndromic orofacial clefts.
16	Çıplak	2024	2020-2021	Turkey	Cohort study	127	Maternal COVID-19 was not associated with bronchopulmonary dysplasia among preterm infants.

17	Eltemamy	2021	2020-2021	Egypt	Cohort study	60 vs 30	Newborns born to women without vs with a history of SARS-CoV-2 infection at week 6-12: No difference in Doppler scan, and gross anomaly scan throughout all following visits
18	Arena	2023	2019-2020	Congo	Cohort study	14300	Prevalence of congenital microcephaly was similar between pre-COVID-19 and intra-COVID-19 period.
19	Khalid	2022	2013-2021	Iraq	Cohort study	?	There was a sudden increase in incidence of neural tube defects during 2021.
20	Heidarzadeh	2022	2019-2021	Iran	Cohort study	?	The incidence of congenital birth anomalies was significantly increased during COVID-19 pandemic compared with before COVID-19, especially birth defect of central nervous system and genitourinary.
21	Hasan	2023	2019-2021	Jordan	Cohort study	1497	Among neonates who had an abnormal echocardiogram, there was a higher rate of persistent pulmonary hypertension during the COVID-19 pandemic than before.
22	Nassar	2024	2012-2023	Israel	Cohort study	Not reported	There was an increase in risk of neonatal biliary atresia during the pandemic.
23	Phadke	2022	2020-2021	India	Cohort study	?	The prevalence of neural tube defects in 2021 was four times that of 2020.
24	Wang	2023	2014-2023	China	Cohort study	462148	After a surge in SARS-CoV-2 infections, there was an increase in risk of situs inversus among pregnant women who underwent ultrasound screening.
25	Qiu	2024	2022-2023	China	Cohort study	8381	Among pregnant women who underwent fetal ultrasound examinations, maternal COVID-19 was associated with fetal situs inversus.
26	Xu	2024	2019-2023	China	Cohort study	31699	There was increased risk of fetal mirror-image dextrocardia after a surge in COVID-19 infection. Among the cases, the timing of COVID-19 infection was about week 5.
27	Guo	2024	2023	China	Case-control study	52 vs 208	Fetal situs inversus was significantly associated with SARS-CoV-2 infection specifically in gestational weeks 4-6.
28	Ren	2024	2023	China	Cohort study	531	The prevalence of cardiac ultrasound abnormalities was significantly higher in the pregnant women with documented COVID-19 compared to those without COVID-19.<br>The annual incidence of congenital heart disease was increasing from 2020 to 2023.
29	Hu	2024	2018-2020	China	Cohort study	19170	Risk of congenital anomalies increased from 4.42% in 2018 to 6.55% in 2020.
30	Adhikari	2021	2020	US	Cohort study	3374	Risk of major malformation was similar between pregnant women with and without SARS-CoV-2 infection during pregnancy (2% vs 2%).
31	Neelam	2023	2020	US	Cohort study	22372	No difference in prevalence of birth defects by trimester of SARS-CoV-2 infection
32	Reppucci	2023	2019-2021	US	Cohort study	194	Rates of intestinal atresia did not differ prior to or after the onset of the pandemic, nor did rates of limb deficiency disorders.
33	Auger	2024	2017-2022	Canada	Cohort study	420222	First trimester SARS-CoV-2 infections were not associated with a greater risk of birth defects compared with no infection.<br>Births during the late pandemic period were more likely to be diagnosed with congenital microcephaly compared with pre-pandemic births.
34	Cardona-Pérez	2021	2020	Mexico	Cohort study	70 vs 170	There were 3 neonates born to pregnant women with SARS-CoV-2 infection during pregnancy diagnosed with malformations, versus zero from the uninfected pregnant women.
35	Pérez-López	2022	-June 2021	Four studies[30, 34, 36, 37]	Meta-analysis	4483 vs 522	Pregnant women without vs with SARS-CoV-2 infection:<br>No significant difference in fetal malformations

38	Sabbagh	2024	2020-2023	5 Arab countries	Cross-sectional study	273 (all with CL/P)	Mothers infected with COVID-19 before and during pregnancy had more than twofold higher odds of having an infant with a more severe CL/P phenotype.
**Potential effects of COVID-19 vaccination**							
39	Trostle	2021	2021	US	Case series	85	Prevalence of congenital anomalies was 1.2% among infants born to pregnant women who received mRNA vaccination.
40	Moro	2022	2020-2021	US	Case series	3462	Among the pregnant women who received a booster dose of mRNA COVID-19 vaccine with a report of adverse event, there were 33 reports of birth defects.
41	Moro	2022	2021-2022	US	Case series	323	Among the pregnant women who received a booster dose of mRNA COVID-19 vaccine with a report of adverse event, there were 2 reports of birth defects (i.e., spina bifida, and an unspecified condition).
42	Woestenberg	2023	2021-2022	Netherlands	Cohort study	795 vs 2926	Risk of major non-genetic congenital anomaly was comparable between pregnant women vaccinated in the first trimester (1.1%) and those unvaccinated (1.2%).
43	Norman	2024	2021-2023	Sweden, Norway	Cohort study	196470	Vaccination of pregnant individuals with mRNA COVID-19 vaccines was not associated with increased risks of neonatal adverse events in their infants.<br>Note: the study outcomes included persistent ductus arteriosus, and persistent pulmonary hypertension of the neonate.
44	Blakeway	2022	2020-2021	UK	Cohort study	133	Risk of fetal abnormalities was similar between women who received at least one dose of COVID-19 vaccine in pregnancy and those unvaccinated (2.2% vs 2.5%).
45	Goldshtein	2022	2021	Israel	Cohort study	24288	No substantial difference in congenital anomalies between newborns exposed and unexposed to maternal BNT162b2 mRNA vaccination in utero.
46	Amer	2024	2022	Saudi Arabia	Cross-sectional study	438	The prevalence of congenital anomalies showed no significant difference between pregnant women who received the COVID-19 vaccine and those who did not.
47	Arulappen	2022	2021?	Malaysia	Cohort study	121	Among the pregnant women who received mRNA COVID-19 vaccine, three (2.5%) neonates were reported to have anomalies.
48	Du	2023	2021	China	Cohort study	326 vs 2032	Risk of major structural birth defects was comparable between pregnant women vaccinated in the first trimester (2.1%) and those unvaccinated (1.5%).
49	Li	2023	2021-2022	China	Cohort study	93 vs 160	Risk of congenital abnormalities was similar (2.15% vs 1.25%) between vaccinated and unvaccinated pregnant women.
50	Ma	2023	2021-2022	China	Cohort study	232 vs 735	Risk of birth defect in pregnant women vaccinated in the first trimester was similar to those unvaccinated (0.83% vs 1.0%).
51	Yang	2023	2022	China	Cohort study	899 vs 481	Pregnant women who received preconception vaccination showed similar risk of congenital defects to those unvaccinated (1.5% vs 1.8%), regardless of natural conception or assisted reproduction treatment.
52	Shimabukuro	2021	2020-2021	US	Cohort study	724	Risk of congenital anomalies among live-born infants born to pregnant women who received mRNA COVID-19 vaccines was similar (2.2%) to published incidence (3%).
53	Ruderman	2022	2021	US	Cohort study	3156	No difference in risk of congenital fetal anomalies (identified with ultrasonography) between vaccinated and unvaccinated pregnant women, and between those vaccinated within and outside teratogenic window.
54	Santos	2024	2020-2021	US	Cohort study	5096	COVID-19 vaccination status, including vaccination during the first trimester of pregnancy, was not associated with an increased risk of composite congenital anomalies.<br>The vaccinated group showed significantly lower risk of eye, ear, face, neck anomalies (2.3% vs 3.3%).
55	Kharbanda	2024	2020-	US	Cohort	42156	Among live-born infants, first-trimester mRNA COVID-19 vaccine exposure was

			2022		study		not associated with an increased risk for selected major structural birth defects.
56	Hui	2023	2021-2022	Australia	Cohort study	32536	Vaccinated women were significantly less likely to have an infant with a major congenital anomaly compared with the unvaccinated group (2.4% vs 3.0%).
57	Alamoudi	2024	2020-2021	5 middle east countries	Case-control	348 vs 629	COVID-19 vaccination during pregnancy is not related to non-syndromic orofacial cleft.
**Potential effects of SARS-CoV-2 infection/COVID-19 & COVID-19 vaccination**							
58	Magnus	2024	2020-2022	Sweden, Denmark, Norway	Cohort study	343066	Covid-19 infection and vaccination during the first trimester of pregnancy were not associated with risk of congenital anomalies.
59	Calvert	2023	2020-2022	Scotland, UK	Cohort study	6731	There was no association between any vaccination (or SARS-CoV-2 infection) and any anomaly or non-genetic anomalies.
60	Sabbagh	2023	2020-2021	Saudi Arabia	Cross-sectional	140380	Incidence of non-syndromic oro-facial clefts (NSOFC): 1.26 per 1000 live-births. There were 21(12.1) or 23(13.2%) of NSOFC's mothers exposed or vaccinated with Covid-19, respectively.
61	Hantoushzadeh	2023	2021	Iran	Cohort study	7052	Among the study groups (i.e., Infected only, Infected + vaccinated, Vaccinated only, and Controls):<br>• Risk of open spina bifida were not affected.<br>• Risk of trisomy 18 was lower in the "Infected" and "Vaccinated" groups compared with controls.<br>AstraZeneca and Sinopharm vaccines were associated with elevation of the calculated risk values of trisomy 21 and trisomy 18.

References

1. Morhart P*, et al.* Maternal SARS-CoV-2 infection during pregnancy: possible impact on the infant. *Eur J Pediatr* **181**, 413-418 (2022).
2. Dosedla E, Gašparová P, Ballová Z, Radaljová K, Calda P. Epidermolysis in a newborn of a mother affected by covid-19 in the 3rd trimester of pregnancy. *Ceska Gynekol* **88**, 13-16 (2023).
3. Batinović E*, et al.* First Croatian Case of Double Aneuploidy: A Child With Klinefelter and Edwards Syndrome (48,XXY,+18) - Possible Causes and Contributing Factors. *Acta Medica (Hradec Kralove)* **66**, 32-36 (2023).
4. Abdelkader MA, Abbas HM, Aboelkhair IM, Alafify ASA, Elgazzar BA, Koura MSE. Congenital heart disease in a patient with COVID-19 infection during early pregnancy: a case report. *Egypt Heart J* **74**, 61 (2022).
5. Jiang Y*, et al.* Maternal SARS-CoV-2 infection and aplasia cutis congenita in a newborn. *J Eur Acad Dermatol Venereol* **36**, e868-e870 (2022).
6. Goldshtrom N*, et al.* Neonates With Complex Cardiac Malformation and Congenital Diaphragmatic Hernia Born to SARS-CoV-2 Positive Women-A Single Center Experience. *World J Pediatr Congenit Heart Surg* **11**, 697-703 (2020).
7. Dalla S, Rausch M, Kellerman R. Case Report: Second-Trimester Maternal COVID-19 Infection and Tetralogy of Fallot. *Am Fam Physician* **104**, 331 (2021).
8. Okido MM, Ragazini CS, Duarte G, Coutinho CM, Marcolin AC. Severe Adams-Oliver Syndrome after Maternal COVID-19 Infection Could Be Another Effect of the SARS-CoV-2 Inflammatory Storm? Case Report. *Fetal Pediatr Pathol* **42**, 131-136 (2023).
9. Chimenea A, García-Díaz L, Calderón AM, Antiñolo G. Prenatal diagnosis of VACTERL association after early-first trimester SARS-COV-2 infection. *Congenit Anom (Kyoto)* **63**, 44-46 (2023).
10. Erciyestepe SG, Pata Ö. COVID-19 positive woman presented with major fetal congenital anomalies: A case report with literature review. *Medicine (Baltimore)* **103**, e39504 (2024).
11. Cosma S*, et al.* Prenatal Biochemical and Ultrasound Markers in COVID-19 Pregnant Patients: A Prospective Case-Control Study. *Diagnostics (Basel)* **11**, (2021).
12. Cosma S*, et al.* Obstetric and neonatal outcomes after SARS-CoV-2 infection in the first trimester of pregnancy: A prospective comparative study. *J Obstet Gynaecol Res* **48**, 393-401 (2022).
13. Ludvigsson JF, Håberg SE, Juliusson PB, Andersen AN, Urhoj SK, Stephansson O. Three Scandinavian countries did not see the same increase in foetal situs inversus observed in China during the COVID-19 pandemic. *Acta Paediatr* **113**, 751-752 (2024).
14. Hofbauer A*, et al.* [SARS-CoV-2 Infection in Pregnancy and Incidence of Congenital Malformations - is there a Correlation? Analysis of 8032 Pregnancies from the CRONOS Registry]. *Z Geburtshilfe Neonatol* **228**, 65-73 (2024).
15. Molnárová A*, et al.* Relationship between COVID-19 and orofacial clefts. *Gen Physiol Biophys* **43**, 313-319 (2024).
16. Çıplak G, Becerir C, Sarı FN, Alyamaç Dizdar E. Effect of Maternal Coronavirus Disease on Preterm Morbidities. *Am J Perinatol* **41**, e1835-e1840 (2024).
17. Eltemamy E*, et al.* Assessment of fetal growth and anomalies in the era of COVID-19 pandemic: an Egyptian pilot study. *Middle East Fertil Soc J* **26**, 32 (2021).
18. Arena PJ*, et al.* Comparison of adverse pregnancy and birth outcomes using archival medical records before and during the first wave of the COVID-19 pandemic in Kinshasa, Democratic Republic of Congo: a facility-based, retrospective cohort study. *BMC Pregnancy Childbirth* **23**, 31 (2023).
19. Fatima k K. Neural Tube Defects Trend, is COVID19 Responsible for Increased Rates? *Pakistan Journal of Medical & Health Sciences* **16**, 822 (2022).
20. Heidarzadeh M, Taheri M, Mazaheripour Z, Abbasi-Khameneh F. The incidence of congenital anomalies in newborns before and during the Covid-19 pandemic. *Ital J Pediatr* **48**, 174 (2022).

21. Hasan AA*, et al.* Incidence and types of congenital heart disease at a referral hospital in Jordan: retrospective study from a tertiary center. *Front Pediatr* **11**, 1261130 (2023).
22. Nassar R, Mozer-Glassberg Y, Rosenfeld Bar-Lev M, Shamir R, Gurevich M, Waisbourd-Zinman O. The Impact of the COVID-19 Pandemic on Biliary Atresia Incidence in a Tertiary Referral Center in Israel. *Pediatr Infect Dis J* **43**, e260 (2024).
23. Phadke M, Nair R, Menon P, Jotkar R, Saunik S. COVID-19 Women's Health, Occurrence of Neural Tube Defects and Severe acute Malnutrition in Children. *Journal of Clinical Nutrition and Dietetics* **8**, (2022).
24. Wang Y*, et al.* Association of SARS-CoV-2 Infection during Early Weeks of Gestation with Situs Inversus. *N Engl J Med* **389**, 1722-1724 (2023).
25. Qiu S, Wu S, Yin R, Wang B, Wu H. Correlation between COVID-19 infection and fetal situs inversus. *Birth Defects Res* **116**, e2324 (2024).
26. Xu W, Wei Y, Huo H, Zhao E, Liu B. Is there an association between COVID-19 infection and fetuses with mirror-image dextrocardia? *Arch Gynecol Obstet* **310**, 1279-1281 (2024).
27. Guo Z*, et al.* Association between situs inversus and maternal SARS-CoV-2 infection at gestational age 4-6 weeks. *Med* **5**, 1433-1441.e1433 (2024).
28. Ren H, Zhang X, Zhang S, Pan J, Wang W. The association of increased incidence of congenital heart disease in newborns with maternal COVID-19 infection during pregnancy. *Sci Rep* **14**, 24866 (2024).
29. Hu J*, et al.* Increased adverse pregnancy outcomes among decreased assisted reproductions during the COVID-19 pandemic: insights from a birth cohort study in Southwest China. *BMC Pregnancy Childbirth* **24**, 766 (2024).
30. Adhikari EH*, et al.* Pregnancy Outcomes Among Women With and Without Severe Acute Respiratory Syndrome Coronavirus 2 Infection. *JAMA Netw Open* **3**, e2029256 (2020).
31. Neelam V*, et al.* Pregnancy and infant outcomes by trimester of SARS-CoV-2 infection in pregnancy-SET-NET, 22 jurisdictions, January 25, 2020-December 31, 2020. *Birth Defects Res* **115**, 145-159 (2023).
32. Reppucci ML*, et al.* Incidence of congenital complications related to COVID-19 infection during pregnancy. *J Neonatal Perinatal Med* **16**, 227-234 (2023).
33. Auger N, Arbour L, Lewin A, Brousseau É, Healy-Profitós J, Luu TM. Congenital anomalies during Covid-19: artifact of surveillance or a real TORCH? *Eur J Epidemiol* **39**, 613-621 (2024).
34. Cardona-Pérez JA*, et al.* Prevalence, clinical features, and outcomes of SARS-CoV-2 infection in pregnant women with or without mild/moderate symptoms: Results from universal screening in a tertiary care center in Mexico City, Mexico. *PLoS One* **16**, e0249584 (2021).
35. Pérez-López FR*, et al.* Obstetric and perinatal outcomes of pregnancies with COVID 19: a systematic review and meta-analysis. *J Matern Fetal Neonatal Med* **35**, 9742-9758 (2022).
36. Flaherman VJ*, et al.* Infant Outcomes Following Maternal Infection With Severe Acute Respiratory Syndrome Coronavirus 2 (SARS-CoV-2): First Report From the Pregnancy Coronavirus Outcomes Registry (PRIORITY) Study. *Clin Infect Dis* **73**, e2810-e2813 (2021).
37. Savirón-Cornudella R*, et al.* Screening of severe acute respiratory syndrome coronavirus-2 infection during labor and delivery using polymerase chain reaction and immunoglobulin testing. *Life Sci* **271**, 119200 (2021).
38. Sabbagh HJ*, et al.* Cleft lip and palate and periconception COVID-19 infection in five arab countries. *Clin Oral Investig* **28**, 586 (2024).
39. Trostle ME, Limaye MA, Avtushka V, Lighter JL, Penfield CA, Roman AS. COVID-19 vaccination in pregnancy: early experience from a single institution. *Am J Obstet Gynecol MFM* **3**, 100464 (2021).
40. Moro PL*, et al.* Post-authorization surveillance of adverse events following COVID-19 vaccines in pregnant persons in the vaccine adverse event reporting system (VAERS), December 2020 - October 2021. *Vaccine* **40**, 3389-3394 (2022).
41. Moro PL, Olson CK, Zhang B, Marquez P, Strid P. Safety of Booster Doses of Coronavirus Disease 2019 (COVID-19) Vaccine in Pregnancy in the Vaccine Adverse Event Reporting System. *Obstet Gynecol* **140**, 421-427 (2022).
42. Woestenberg PJ, de Feijter M, Bergman JEH, Lutke LR, Passier A, Kant AC. Maternal first trimester COVID-19 vaccination and risk of major non-genetic congenital anomalies. *Birth Defects Res* **115**, 1746-1757 (2023).
43. Norman M*, et al.* Neonatal Outcomes After COVID-19 Vaccination in Pregnancy. *Jama* **331**, 396-407 (2024).
44. Blakeway H*, et al.* COVID-19 vaccination during pregnancy: coverage and safety. *Am J Obstet Gynecol* **226**, 236.e231-236.e214 (2022).
45. Goldshtein I*, et al.* Association of BNT162b2 COVID-19 Vaccination During Pregnancy With Neonatal and Early Infant Outcomes. *JAMA Pediatr* **176**, 470-477 (2022).
46. Amer AA*, et al.* Exploring COVID-19 vaccine adverse events among pregnant women: a cross-sectional study, 2022. *Ther Adv Vaccines Immunother* **12**, 25151355241285594 (2024).
47. Arulappen AL, Danial M, Shanmugam G, Cheng JT, Dulasi M, Chow TS. A Multicenter Cohort Study on the Adverse Effects Evaluation After Messenger RNA COVID-19 Vaccination Among Pregnant Healthcare Employees in Penang General Hospitals. *Front Public Health* **10**, 876966 (2022).
48. Du T, Qu Q, Zhang Y, Huang Q. No observable influence of COVID-19 inactivated vaccines on pregnancy and birth outcomes in the first trimester of gestation. *Expert Rev Vaccines* **22**, 900-905 (2023).
49. Li M*, et al.* Maternal and neonatal safety of COVID-19 vaccination during the peri-pregnancy period: A prospective study. *J Med Virol* **95**, e28378 (2023).
50. Ma Y, Shan Z, Gu Y, Huang Y. Safety and efficacy of inactivated COVID-19 vaccines in women vaccinated during the first trimester of pregnancy. *Int J Infect Dis* **130**, 196-202 (2023).
51. Yang Y*, et al.* Pregnancy outcomes following natural conception and assisted reproduction treatment in women who received COVID-19 vaccination prior to conception: a population-based cohort study in China. *Front Med (Lausanne)* **10**, 1250165 (2023).
52. Shimabukuro TT*, et al.* Preliminary Findings of mRNA Covid-19 Vaccine Safety in Pregnant Persons. *N Engl J Med* **384**, 2273-2282 (2021).
53. Ruderman RS*, et al.* Association of COVID-19 Vaccination During Early Pregnancy With Risk of Congenital Fetal Anomalies. *JAMA Pediatr* **176**, 717-719 (2022).
54. Santos J, Miller M, Branda ME, Mehta RA, Theiler RN. Maternal COVID-19 vaccination status and association with neonatal congenital anomalies. *Front Pediatr* **12**, 1355502 (2024).
55. Kharbanda EO*, et al.* COVID-19 Vaccination in the First Trimester and Major Structural Birth Defects Among Live Births. *JAMA Pediatr*, (2024).
56. Hui L*, et al.* Reductions in stillbirths and preterm birth in COVID-19-vaccinated women: a multicenter cohort study of vaccination uptake and perinatal outcomes. *Am J Obstet Gynecol* **228**, 585 e581-585 e516 (2023).
57. Alamoudi RA*, et al.* COVID-19 vaccine and non-syndromic orofacial clefts in five arab countries. A case-control study. *Clin Oral Investig* **28**, 357 (2024).
58. Magnus MC*, et al.* Covid-19 infection and vaccination during first trimester and risk of congenital anomalies: Nordic registry based study. *Bmj* **386**, e079364 (2024).

59. Calvert C*, et al.* A population-based matched cohort study of major congenital anomalies following COVID-19 vaccination and SARS-CoV-2 infection. *Nat Commun* **14**, 107 (2023).
60. Sabbagh HJ*, et al.* Incidence of non-syndromic orofacial cleft during the COVID-19 pandemic in Saudi Arabia. *BMC Public Health* **23**, 2538 (2023).
61. Hantoushzadeh S*, et al.* Impact of COVID-19 and vaccination on first and second trimester screening results. *Cytokine* **168**, 156228 (2023).

Supplementary methods
1. Variables used in the study

Variable name in the study	Original variable name†	Note
**Maternal/perinatal characteristics**		
Birth year	DOB_YY	
Birth month	DOB_MM	
Maternal age (continuous)	MAGER	Age of mother is imputed for ages 8 years or under and 65 years and over. Births to mothers 9-11 years are collapsed into the categories "12 years or under;" births to mothers 50-64 years into the category "50-54 years."
Maternal age (categorical)	MAGER9	<15, 15-19, 20-24, 25-29, 30-34, 35-39, 40-44, 45-49, 50-54
Maternal residence	RESTATUS	Resident, Intrastate nonresident, Interstate nonresident, Foreign resident
Maternal ethnicity/race	MRACEHISP	Non-Hispanic White (only), Non-Hispanic Black (only), Non-Hispanic American Indian or Alaskan Native (AIAN) (only), Non-Hispanic Asian (only), Non-Hispanic Native Hawaiian or Other Pacific Islander (NHOPI) (only), Non-Hispanic more than one race, Hispanic, Unknown
Maternal education attainment	MEDUC	8th grade or less, 9th-12th grade, no diploma, High school graduate or GED completed, Some college credit but no degree, Associate degree, Bachelor's degree, Master's degree, Doctorate or Professional degree, Unknown<br><br>An Associate degree refers to AA (Associate of Arts) or AS (Associate of Science); A Bachelor's degree refers to BA (Bachelor of Arts), AB ("Artium Baccalaureus", Bachelor of Arts in Latin), or BS (Bachelor of Science); A Master's degree refers to MA (Master of Arts), MS (Master of Science), MEng (Master of Engineering), MEd (Master of Education), MSW (Master of Social Work), or MBA (Master of Business Administration); A Doctorate degree refers to PhD (Doctor of Philosophy) or EdD (Doctor of Education); A Professional Degree refers to MD (Doctor of Medicine), DDS (Doctor of Dental Surgery), DVM (Doctor of Veterinary Medicine), LLB (Bachelor of Laws), or JD (Juris Doctor).
Principal source of payment for current delivery	PAY_REC	Medicaid, Private Insurance, Self-pay, Other, Unknown
Marital status	DMAR	Married, Unmarried, Unknown
No. previous live births now dead	PRIORDEAD	0.4% were missing
No. other pregnancy outcomes	PRIORTERM	0.5% were missing; including spontaneous or induced losses or ectopic pregnancies
First prenatal care visit	PRECARE5	1-3, 4-6, ≥7, No prenatal care, Unknown
WIC food during pregnancy	WIC	No, Yes, Unknown<br>The Special Supplemental Nutrition Program for Women, Infants, and Children (which is administered by the U.S. Department of Agriculture and intended to help low-income pregnant women, infants, and children through age 5 receive proper nutrition by providing vouchers for food, nutrition counseling, health care screenings and referrals
Cigarette smoking during pregnancy	CIG_REC	No, Yes, Unknown
Maternal body mass index (BMI)	BMI_R	<18.5 (Underweight), 18.5-24.9 (Normal), 25.0-29.9 (Overweight), 30.0-34.9 (Obesity class I), 35.0-39.9 (Obesity class II), ≥40.0 (Obesity class III), Unknown; kg/m^2
Prepregnancy diabetes	RF_PDIAB	No, Yes, Unknown
Gestational diabetes	RF_GDIAB	No, Yes, Unknown
Prepregnancy hypertension	RF_PHYPE	No, Yes, Unknown
Gestational hypertension	RF_GHYPE	No, Yes, Unknown
Pregnancy resulted from infertility treatment	RF_INFTR	No, Yes, Unknown
Fertility enhancing drugs/artificial insemination/intrauterine insemination	RF_FEDRG	No, Yes, Not applicable, Unknown
Assisted reproductive technology	RF_ARTEC	No, Yes, Not applicable, Unknown<br>Including in vitro fertilization (IVF), gamete intrafallopian transfer (GIFT), or zygote intrafallopian transfer (ZIFT)
Ruptured uterus	MM_RUPT	No, Yes, Unknown
Unplanned hysterectomy	MM_UHYST	No, Yes, Unknown

Infections present and/or treated during current pregnancy	NO_INFEC	No, Yes, Unknown<br>Including Gonorrhea, Syphilis, Chlamydia, Hepatitis B, and/or Hepatitis C
Obstetric estimate of gestation (continuous)	OEGest_Comb	0.1% were missing<br>According to the NCHS standard for obstetric estimate of gestation at delivery (in weeks)<br>Reference: Martin JA, Osterman, MJK, Kirmeyer SE, Gregory, ECW. Measuring Gestational Age in Vital Statistics: Transitioning to the Obstetric Estimate. National Vital Statistics Reports; vol. 64, no. 5. Hyattsville, MD: National Center for Health Statistics. 2015.
Obstetric estimate of gestation (categorical, 2-category)	OEGest_R3	<37, ≥37 weeks, Unknown
Obstetric estimate of gestation (categorical, 10-category)	OEGest_R10	<20, 20-27, 28-31, 32-33, 34-36, 37-38, 39, 40, 41, ≥42 weeks, Unknown
Birth place	BFACIL3	In Hospital, Not in hospital, Unknown
Attendant at birth	ATTEND	Doctor of Medicine, Doctor of Osteopathy, Certified Nurse Midwife/Certified Midwife, Other Midwife, Other, Unknown
Induction of labor	LD_INDL	No, Yes, Unknown
Augmentation of labor	LD_AUGM	No, Yes, Unknown
Steroids (glucocorticoids) for fetal lung maturation received by the mother prior to delivery	LD_STER	No, Yes, Unknown
Antibiotics received by the mother during labor	LD_ANTB	No, Yes, Unknown
Clinical chorioamnionitis diagnosed during labor or maternal temperature ≥38°C	LD_CHOR	No, Yes, Unknown
Epidural or spinal anesthesia during labor	LD_ANES	No, Yes, Unknown
Method of delivery (3-category)	DMETH_REC	Vaginal, C-section, Unknown
Method of delivery (6-category)	RDMETH_REC	Vaginal (excludes vaginal after previous C-section), Vaginal after previous C-section, Primary C-section, Repeat C-section, Vaginal (unknown if previous C-section), C-section (unknown if previous C-section), Unknown
Fetal presentation at birth	ME_PRES	Cephalic, Breech, Other, Unknown
Final route and method of delivery	ME_ROUT	Vaginal/Spontaneous, Vaginal/Forceps, Vaginal/Vacuum, Cesarean, Unknown
A trial of labor attempted if cesarean	ME_TRIAL	No, Yes, Not applicable, Unknown
Plurality	DPLURAL	Single, Twin, Triplet, Quadruplet or higher;<br>Records for which plurality is unknown are imputed as singletons. This occurred for 0.007% (253) of all records for 2022.
Sex of the infant	SEX	Male, Female
Birthweight (continuous)	DBWT	0.1% were missing
Birthweight (categorical, 3-category)	BWTR4	227-1499, 1500–2499, 2500-8165, Unknown
Birthweight (categorical, 11-category)	BWTR12	227-499, 500–999, 1000-1499, 1500–1999, 2000–2499, 2500–2999, 3000–3499, 3500–3999, 4000–4499, 4500–4999, 5000–8165, Unknown
Apgar score at five minutes	APGAR5R	0-3, 4-6, 7-8, 9-10, Unknown
Assisted ventilation required immediately following delivery	AB_AVEN1	No, Yes, Unknown
Assisted ventilation required for more than six hours	AB_AVEN6	No, Yes, Unknown
Neonatal intensive care unit (NICU) admission	AB_NICU	No, Yes, Unknown
Newborn given surfactant replacement therapy	AB_SURF	No, Yes, Unknown
Antibiotics received by the newborn for suspected neonatal sepsis	AB_ANTI	No, Yes, Unknown
Seizure or serious neurologic dysfunction	AB_SEIZ	No, Yes, Unknown

**Study outcomes**		
Congenital anomalies	NO_CONGEN	No, Yes, Missing<br>Including Anencephaly, Meningomyelocele/Spina bifida, Cyanotic congenital heart disease, Congenital diaphragmatic hernia, Omphalocele, Gastroschisis, Limb reduction defect, Cleft Lip with or without Cleft Palate, Cleft Palate alone, Down syndrome, Suspected chromosomal disorder, or Hypospadias
Anencephaly	CA_ANEN	No, Yes, Missing
Meningomyelocele/Spina bifida	CA_MNSB	No, Yes, Missing
Cyanotic congenital heart disease	CA_CCHD	No, Yes, Missing
Congenital diaphragmatic hernia	CA_CDH	No, Yes, Missing
Omphalocele	CA_OMPH	No, Yes, Missing
Gastroschisis	CA_GAST	No, Yes, Missing
Limb reduction defect	CA_LIMB	No, Yes, Missing<br>Excluding congenital amputation and dwarfing syndromes
Cleft Lip with or without Cleft Palate	CA_CLEFT	No, Yes, Missing
Cleft Palate alone	CA_CLPAL	No, Yes, Missing
Down syndrome	CA_DOWN	No, Pending, Confirmed, Missing
Suspected chromosomal disorder	CA_DISOR	No, Pending, Confirmed, Missing
Hypospadias	CA_HYPO	No, Yes, Missing
Transferred within 24 hours of delivery	ITRAN	No, Yes, Missing
Death before birth-certificate registration	ILIVE	Infant living at time of report: Yes, No, Missing (analysed as death before birth-certificate registration, i.e. ‘No’)

† The Original variable names are based on the User Guide to the 2022 Natality Public Use File. The equivalent variables in datasets of other birth years were used for the study, and the default categories might be reset to keep consistent.

Supplementary Tables

Supplemental Table S1 Operational definitions of the birth-certificate items used as study outcomes

Item on the birth certificate	Variable in the natality public-use file	Definition and certifier instruction (NCHS Guide to Completing the Facility Worksheets)	Keywords and abbreviations listed in the Guide
Anencephaly	Newborn_congenital_anomalies_Anencephaly (CA_ANEN)	Partial or complete absence of the brain and skull. Also called anencephalus, acrania, or absent brain. Also includes infants with craniorachischisis (anencephaly with a contiguous spine defect).	Anencephalus; acrania; absent brain; craniorachischisis; exencephaly; hydraencephaly
Meningomyelocele / spina bifida	..._Meningomyelocele_Spina_Bifida (CA_MNSB)	Spina bifida is herniation of the meninges or spinal cord tissue through a bony defect of spine closure. Meningomyelocele is herniation of meninges and spinal cord tissue. Meningocele (herniation of meninges without spinal cord tissue) should also be included in this category. Both open and closed (covered with skin) lesions should be included. Do not include spina bifida occulta.	Meningocele; rachischisis
Cyanotic congenital heart disease	..._Cyanotic_Congenital_Heart_Disease (CA_CCHD)	Congenital heart defects that cause cyanosis. Source given to the certifier: physician progress notes under Circulation/Cardiovascular.	TGA (transposition of the great arteries); TOF (tetralogy of Fallot); pulmonary or pulmonic valvular atresia; tricuspid atresia; truncus arteriosus; TAPVR; COA (coarctation of the aorta); HLHS; IAA (interrupted aortic arch)
Congenital diaphragmatic hernia	..._Congenital_Diaphragmatic_Hernia (CA_CDH)	Defect in the formation of the diaphragm allowing herniation of abdominal organs into the thoracic cavity.	
Omphalocele	..._Omphalocele (CA_OMPH)	A defect in the anterior abdominal wall in which the umbilical ring is widened, allowing herniation of abdominal organs into the umbilical cord. The herniating organs are covered by a nearly transparent membranous sac, although this sac may rupture. Do not include umbilical hernia.	Exomphalos
Gastroschisis	..._Gastroschisis (CA_GAST)	An abnormality of the anterior abdominal wall, lateral to the umbilicus, resulting in herniation of the abdominal contents directly into the amniotic cavity. Differentiated from omphalocele by the location of the defect and the absence of a protective membrane.	
Limb reduction defect	..._Limb_Reduction_Defect (CA_LIMB)	Limb reduction defect excluding congenital amputation and dwarfing syndromes. Complete or partial absence of a portion of an extremity, secondary to failure to develop.	Amniotic bands; amniotic band syndrome
Cleft lip with or without cleft palate	..._Cleft_Lip_with_with_out_Cleft_Palate (CA_CLEFT)	Incomplete closure of the lip. May be unilateral, bilateral, or median.	Cleft lip (unilateral, bilateral, or median)
Cleft palate alone	..._Cleft_Palate_alone (CA_CLPAL)	Incomplete fusion of the palatal shelves. May be limited to the soft palate or may extend into the hard palate. Cleft palate in the presence of cleft lip is included in the category above.	
Down syndrome	..._Down_Syndrome (CA_DOWN)	Trisomy 21 - a chromosomal abnormality caused by the presence of all or part of a third copy of	Trisomy 21; positive (confirmed); possible Down (pending); rule out Down (pending); trisomy 21

		chromosome 21. Recorded as karyotype confirmed or karyotype pending. Certifier instruction: check if a diagnosis of Down syndrome / trisomy 21 is confirmed or pending.	mosaicism
Suspected chromosomal disorder	..._Suspected_Chromosomal_Disorder (CA_DISOR)	Includes any constellation of congenital malformations resulting from or compatible with known syndromes caused by detectable defects in chromosome structure. Recorded as karyotype confirmed or karyotype pending. Certifier instruction: check if a diagnosis of a suspected chromosomal disorder is confirmed or pending (may include trisomy 21).	Trisomy 13 (Patau syndrome); trisomy 18 (Edwards syndrome); positive (confirmed); possible trisomy (pending); rule out (pending)
Hypospadias	..._Hypospadias (CA_HYPO)	Incomplete closure of the male urethra resulting in the urethral meatus opening on the ventral surface of the penis. Includes first degree (on the glans ventral to the tip), second degree (in the coronal sulcus) and third degree (on the penile shaft).	
Transferred within 24 hours of delivery	Infant_Transferred (ITRAN)	Transfer status of the infant from this facility to another within 24 hours after delivery. Certifier instruction: check 'yes' if the infant was transferred from this facility to another within 24 hours of delivery; if the infant was transferred more than once, enter the name of the first facility.	Disposition
Infant living at time of report (analysed as death before birth-certificate registration)	Infant_Living_at_Time_of_Report (ILIVE)	Information on the infant's survival. Certifier instruction: check 'yes' if the infant is living; check 'yes' if the infant has already been discharged to home care; check 'no' if it is known that the infant has died. Because certificates are typically filed within the first days of life, this item does not capture late neonatal, postneonatal or post-discharge death.	

Note: Definitions, certifier instructions and keyword lists are reproduced verbatim from the NCHS Guide to Completing the Facility Worksheets for the Certificate of Live Birth and Report of Fetal Death, item 37 (Congenital anomalies of the newborn; birth-certificate item 54), item 38 (Was the infant transferred within 24 hours of delivery?) and item 39 (Is the infant living at the time of the report?; birth-certificate item 57), available at https://www.cdc.gov/nchs/nvss/facility-worksheets-guide.htm (last reviewed 6 August 2019). This is the edition in force at the end of the study period, and the wording of these items and of the accompanying instructions was unchanged throughout 2016-2022. The certificate provides a checkbox only: no diagnostic criteria, imaging, karyotype or other postnatal confirmation is required, no ICD coding is applied, and the certifier is directed to the delivery record rather than to a diagnostic report. A blank cell in the final column indicates that the Guide lists no keywords or abbreviations for that item. These definitions are the reason the outcome is described throughout as the birth-certificate–reported prevalence at live birth rather than as the incidence or the birth prevalence of the conditions.

Supplemental Table S2 Missingness of the study outcomes by birth year

Birth year	Overall	2016	2017	2018	2019	2020	2021	2022
n	26345765	3956112	3864754	3801534	3757582	3619826	3669928	3676029
**Congenital anomalies**								
No	26211702 (99.5)	3935493 (99.5)	3846412 (99.5)	3782113 (99.5)	3739424 (99.5)	3602271 (99.5)	3649744 (99.5)	3656245 (99.5)
Yes	90509 (0.3)	13791 (0.3)	13405 (0.3)	13314 (0.4)	12690 (0.3)	12390 (0.3)	12636 (0.3)	12283 (0.3)
Missing	43554 (0.2)	6828 (0.2)	4937 (0.1)	6107 (0.2)	5468 (0.1)	5165 (0.1)	7548 (0.2)	7501 (0.2)
**Anencephaly**								
No	26299697 (99.8)	3948918 (99.8)	3859385 (99.9)	3795073 (99.8)	3751781 (99.8)	3614329 (99.8)	3662070 (99.8)	3668141 (99.8)
Yes	2514 (0.0)	366 (0.0)	432 (0.0)	354 (0.0)	333 (0.0)	332 (0.0)	310 (0.0)	387 (0.0)
Missing	43554 (0.2)	6828 (0.2)	4937 (0.1)	6107 (0.2)	5468 (0.1)	5165 (0.1)	7548 (0.2)	7501 (0.2)
**Meningomyelocele/Spina bifida**								
No	26298486 (99.8)	3948736 (99.8)	3859221 (99.9)	3794872 (99.8)	3751575 (99.8)	3614171 (99.8)	3661882 (99.8)	3668029 (99.8)
Yes	3725 (0.0)	548 (0.0)	596 (0.0)	555 (0.0)	539 (0.0)	490 (0.0)	498 (0.0)	499 (0.0)
Missing	43554 (0.2)	6828 (0.2)	4937 (0.1)	6107 (0.2)	5468 (0.1)	5165 (0.1)	7548 (0.2)	7501 (0.2)
**Cyanotic congenital heart disease**								
No	26286240 (99.8)	3947084 (99.8)	3857687 (99.8)	3793211 (99.8)	3749893 (99.8)	3612373 (99.8)	3659890 (99.7)	3666102 (99.7)
Yes	15971 (0.1)	2200 (0.1)	2130 (0.1)	2216 (0.1)	2221 (0.1)	2288 (0.1)	2490 (0.1)	2426 (0.1)
Missing	43554 (0.2)	6828 (0.2)	4937 (0.1)	6107 (0.2)	5468 (0.1)	5165 (0.1)	7548 (0.2)	7501 (0.2)
**Congenital diaphragmatic hernia**								
No	26298878 (99.8)	3948783 (99.8)	3859346 (99.9)	3794955 (99.8)	3751642 (99.8)	3614192 (99.8)	3661918 (99.8)	3668042 (99.8)
Yes	3333 (0.0)	501 (0.0)	471 (0.0)	472 (0.0)	472 (0.0)	469 (0.0)	462 (0.0)	486 (0.0)
Missing	43554 (0.2)	6828 (0.2)	4937 (0.1)	6107 (0.2)	5468 (0.1)	5165 (0.1)	7548 (0.2)	7501 (0.2)
**Omphalocele**								
No	26299540 (99.8)	3948880 (99.8)	3859417 (99.9)	3795019 (99.8)	3751717 (99.8)	3614325 (99.8)	3662004 (99.8)	3668178 (99.8)
Yes	2671 (0.0)	404 (0.0)	400 (0.0)	408 (0.0)	397 (0.0)	336 (0.0)	376 (0.0)	350 (0.0)
Missing	43554 (0.2)	6828 (0.2)	4937 (0.1)	6107 (0.2)	5468 (0.1)	5165 (0.1)	7548 (0.2)	7501 (0.2)
**Gastroschisis**								
No	26296646 (99.8)	3948325 (99.8)	3858891 (99.8)	3794597 (99.8)	3751285 (99.8)	3613915 (99.8)	3661691 (99.8)	3667942 (99.8)
Yes	5565 (0.0)	959 (0.0)	926 (0.0)	830 (0.0)	829 (0.0)	746 (0.0)	689 (0.0)	586 (0.0)
Missing	43554 (0.2)	6828 (0.2)	4937 (0.1)	6107 (0.2)	5468 (0.1)	5165 (0.1)	7548 (0.2)	7501 (0.2)
**Limb reduction defect**								
No	26298917 (99.8)	3948735 (99.8)	3859291 (99.9)	3794940 (99.8)	3751673 (99.8)	3614221 (99.8)	3661953 (99.8)	3668104 (99.8)
Yes	3294 (0.0)	549 (0.0)	526 (0.0)	487 (0.0)	441 (0.0)	440 (0.0)	427 (0.0)	424 (0.0)
Missing	43554 (0.2)	6828 (0.2)	4937 (0.1)	6107 (0.2)	5468 (0.1)	5165 (0.1)	7548 (0.2)	7501 (0.2)
**Cleft Lip with or without Cleft Palate**								
No	26288642 (99.8)	3947173 (99.8)	3857824 (99.8)	3793373 (99.8)	3750274 (99.8)	3612869 (99.8)	3660463 (99.7)	3666666 (99.7)
Yes	13569 (0.1)	2111 (0.1)	1993 (0.1)	2054 (0.1)	1840 (0.0)	1792 (0.0)	1917 (0.1)	1862 (0.1)
Missing	43554 (0.2)	6828 (0.2)	4937 (0.1)	6107 (0.2)	5468 (0.1)	5165 (0.1)	7548 (0.2)	7501 (0.2)
**Cleft Palate alone**								
No	26296069 (99.8)	3948369 (99.8)	3858889 (99.8)	3794507 (99.8)	3751249 (99.8)	3613815 (99.8)	3661533 (99.8)	3667707 (99.8)
Yes	6142 (0.0)	915 (0.0)	928 (0.0)	920 (0.0)	865 (0.0)	846 (0.0)	847 (0.0)	821 (0.0)
Missing	43554 (0.2)	6828 (0.2)	4937 (0.1)	6107 (0.2)	5468 (0.1)	5165 (0.1)	7548 (0.2)	7501 (0.2)
**Down syndrome**								
No	26288143 (99.8)	3947116 (99.8)	3857773 (99.8)	3793319 (99.8)	3750084 (99.8)	3612707 (99.8)	3660477 (99.7)	3666667 (99.7)
Pending	7815 (0.0)	1229 (0.0)	1143 (0.0)	1187 (0.0)	1107 (0.0)	1060 (0.0)	1046 (0.0)	1043 (0.0)

Confirmed	6253 (0.0)	939 (0.0)	901 (0.0)	921 (0.0)	923 (0.0)	894 (0.0)	857 (0.0)	818 (0.0)
Missing	43554 (0.2)	6828 (0.2)	4937 (0.1)	6107 (0.2)	5468 (0.1)	5165 (0.1)	7548 (0.2)	7501 (0.2)
**Suspected chromosomal disorder**								
No	26290482 (99.8)	3947584 (99.8)	3858159 (99.8)	3793701 (99.8)	3750447 (99.8)	3613009 (99.8)	3660649 (99.7)	3666933 (99.8)
Pending	7800 (0.0)	1117 (0.0)	1121 (0.0)	1144 (0.0)	1099 (0.0)	1126 (0.0)	1150 (0.0)	1043 (0.0)
Confirmed	3929 (0.0)	583 (0.0)	537 (0.0)	582 (0.0)	568 (0.0)	526 (0.0)	581 (0.0)	552 (0.0)
Missing	43554 (0.2)	6828 (0.2)	4937 (0.1)	6107 (0.2)	5468 (0.1)	5165 (0.1)	7548 (0.2)	7501 (0.2)
**Hypospadias**								
No	26287256 (99.8)	3946828 (99.8)	3857500 (99.8)	3793189 (99.8)	3750044 (99.8)	3612653 (99.8)	3660424 (99.7)	3666618 (99.7)
Yes	14955 (0.1)	2456 (0.1)	2317 (0.1)	2238 (0.1)	2070 (0.1)	2008 (0.1)	1956 (0.1)	1910 (0.1)
Missing	43554 (0.2)	6828 (0.2)	4937 (0.1)	6107 (0.2)	5468 (0.1)	5165 (0.1)	7548 (0.2)	7501 (0.2)
**Transferred within 24 hours of delivery**								
No	26010514 (98.7)	3908642 (98.8)	3817765 (98.8)	3754764 (98.8)	3709597 (98.7)	3574716 (98.8)	3619928 (98.6)	3625102 (98.6)
Yes	303671 (1.2)	44602 (1.1)	44502 (1.2)	44523 (1.2)	44212 (1.2)	40640 (1.1)	42909 (1.2)	42283 (1.2)
Missing	31580 (0.1)	2868 (0.1)	2487 (0.1)	2247 (0.1)	3773 (0.1)	4470 (0.1)	7091 (0.2)	8644 (0.2)
**Death before birth-certificate registration**								
Yes	64137 (0.2)	10860 (0.3)	10218 (0.3)	9683 (0.3)	8983 (0.2)	8323 (0.2)	7925 (0.2)	8145 (0.2)
No	26238554 (99.6)	3939033 (99.6)	3848096 (99.6)	3784682 (99.6)	3742320 (99.6)	3605495 (99.6)	3656273 (99.6)	3662655 (99.6)
Missing	43074 (0.2)	6219 (0.2)	6440 (0.2)	7169 (0.2)	6279 (0.2)	6008 (0.2)	5730 (0.2)	5229 (0.1)

Note: Details about each variable are presented in Supplementary methods.

Supplemental Table S3 Other maternal and perinatal characteristics of the included infants by birth year

Birth year	Overall	2016	2017	2018	2019	2020	2021	2022
n	26345765	3956112	3864754	3801534	3757582	3619826	3669928	3676029
**Maternal age (years)**								
<15	13184 (0.1)	2256 (0.1)	1921 (0.0)	1740 (0.0)	1790 (0.0)	1768 (0.0)	1881 (0.1)	1828 (0.0)
15-19	1206618 (4.6)	210240 (5.3)	194761 (5.0)	180219 (4.7)	172041 (4.6)	158243 (4.4)	147123 (4.0)	143991 (3.9)
20-24	4960125 (18.8)	805610 (20.4)	766112 (19.8)	727625 (19.1)	705717 (18.8)	666314 (18.4)	649101 (17.7)	639646 (17.4)
25-29	7529512 (28.6)	1152194 (29.1)	1126355 (29.1)	1102334 (29.0)	1081033 (28.8)	1026153 (28.3)	1025631 (27.9)	1015812 (27.6)
30-34	7705541 (29.2)	1114184 (28.2)	1094790 (28.3)	1093761 (28.8)	1092397 (29.1)	1072000 (29.6)	1116917 (30.4)	1121492 (30.5)
35-39	4014487 (15.2)	549016 (13.9)	556241 (14.4)	568410 (15.0)	574269 (15.3)	565162 (15.6)	593239 (16.2)	608150 (16.5)
40-44	848976 (3.2)	113501 (2.9)	115162 (3.0)	117780 (3.1)	120606 (3.2)	120859 (3.3)	126586 (3.4)	134482 (3.7)
45-49	60238 (0.2)	8306 (0.2)	8551 (0.2)	8681 (0.2)	8625 (0.2)	8303 (0.2)	8396 (0.2)	9376 (0.3)
50-54	7084 (0.0)	805 (0.0)	861 (0.0)	984 (0.0)	1104 (0.0)	1024 (0.0)	1054 (0.0)	1252 (0.0)
**Maternal residence**								
Resident	18207008 (69.1)	2803125 (70.9)	2719506 (70.4)	2650118 (69.7)	2601910 (69.2)	2486335 (68.7)	2479685 (67.6)	2466329 (67.1)
Intrastate nonresident	7432827 (28.2)	1051713 (26.6)	1045868 (27.1)	1050665 (27.6)	1054510 (28.1)	1037269 (28.7)	1088058 (29.6)	1104744 (30.1)
Interstate nonresident	646489 (2.5)	91037 (2.3)	90126 (2.3)	90929 (2.4)	91120 (2.4)	90043 (2.5)	96549 (2.6)	96685 (2.6)
Foreign resident	59441 (0.2)	10237 (0.3)	9254 (0.2)	9822 (0.3)	10042 (0.3)	6179 (0.2)	5636 (0.2)	8271 (0.2)
**Maternal education attainment**								
8th grade or less	829108 (3.1)	132478 (3.3)	124941 (3.2)	118177 (3.1)	116141 (3.1)	110594 (3.1)	108350 (3.0)	118427 (3.2)
9th-12th grade, no diploma	2376992 (9.0)	407598 (10.3)	381896 (9.9)	358686 (9.4)	338337 (9.0)	312781 (8.6)	291669 (7.9)	286025 (7.8)
High school graduate or GED completed	6742803 (25.6)	981410 (24.8)	974429 (25.2)	968380 (25.5)	967286 (25.7)	945784 (26.1)	946517 (25.8)	958997 (26.1)
Some college credit but no degree	5082658 (19.3)	809083 (20.5)	781473 (20.2)	752253 (19.8)	730270 (19.4)	686298 (19.0)	672353 (18.3)	650928 (17.7)
Associate degree	2179940 (8.3)	322703 (8.2)	316816 (8.2)	314455 (8.3)	310241 (8.3)	299808 (8.3)	308199 (8.4)	307718 (8.4)
Bachelor's degree	5468223 (20.8)	788562 (19.9)	776965 (20.1)	776969 (20.4)	775088 (20.6)	755455 (20.9)	796371 (21.7)	798813 (21.7)
Master's degree	2547938 (9.7)	359830 (9.1)	355719 (9.2)	358924 (9.4)	360980 (9.6)	353469 (9.8)	378004 (10.3)	381012 (10.4)
Doctorate or Professional degree	742045 (2.8)	102727 (2.6)	102728 (2.7)	104468 (2.7)	104733 (2.8)	103500 (2.9)	111469 (3.0)	112420 (3.1)
Unknown	376058 (1.4)	51721 (1.3)	49787 (1.3)	49222 (1.3)	54506 (1.5)	52137 (1.4)	56996 (1.6)	61689 (1.7)
**Principal source of payment for current delivery**								
Medicaid	10983986 (41.7)	1670806 (42.2)	1648323 (42.7)	1596254 (42.0)	1568296 (41.7)	1507268 (41.6)	1490442 (40.6)	1502597 (40.9)
Private Insurance	13119476 (49.8)	1938067 (49.0)	1882268 (48.7)	1871419 (49.2)	1868726 (49.7)	1816441 (50.2)	1880449 (51.2)	1862106 (50.7)
Self-pay	1104783 (4.2)	168723 (4.3)	163242 (4.2)	164540 (4.3)	162933 (4.3)	145426 (4.0)	145188 (4.0)	154731 (4.2)
Other	953354 (3.6)	153876 (3.9)	147445 (3.8)	146575 (3.9)	131633 (3.5)	124644 (3.4)	125562 (3.4)	123619 (3.4)
Unknown	184166 (0.7)	24640 (0.6)	23476 (0.6)	22746 (0.6)	25994 (0.7)	26047 (0.7)	28287 (0.8)	32976 (0.9)
**Marital status**								
Married	14193232 (53.9)	2383288 (60.2)	2032203 (52.6)	2008022 (52.8)	1974916 (52.6)	1894880 (52.3)	1945690 (53.0)	1954233 (53.2)
Unmarried	9513733 (36.1)	1572824 (39.8)	1359731 (35.2)	1337429 (35.2)	1335013 (35.5)	1304046 (36.0)	1303150 (35.5)	1301540 (35.4)
Unknown	2638800 (10.0)	0 (0.0)	472820 (12.2)	456083 (12.0)	447653 (11.9)	420900 (11.6)	421088 (11.5)	420256 (11.4)
**WIC food during pregnancy**								
No	17112737 (65.0)	2361577 (59.7)	2366493 (61.2)	2407680 (63.3)	2457297 (65.4)	2435741 (67.3)	2546343 (69.4)	2537606 (69.0)
Yes	8930278 (33.9)	1544804 (39.0)	1453053 (37.6)	1351578 (35.6)	1256694 (33.4)	1146246 (31.7)	1082290 (29.5)	1095613 (29.8)
Unknown	302750 (1.1)	49731 (1.3)	45208 (1.2)	42276 (1.1)	43591 (1.2)	37839 (1.0)	41295 (1.1)	42810 (1.2)
**Cigarette smoking during pregnancy**								
No	24708356 (93.8)	3653855 (92.4)	3580938 (92.7)	3539052 (93.1)	3516194 (93.6)	3405905 (94.1)	3486014 (95.0)	3526398 (95.9)

Yes	1517239 (5.8)	282788 (7.1)	264975 (6.9)	245360 (6.5)	222146 (5.9)	199631 (5.5)	168140 (4.6)	134199 (3.7)
Unknown	120170 (0.5)	19469 (0.5)	18841 (0.5)	17122 (0.5)	19242 (0.5)	14290 (0.4)	15774 (0.4)	15432 (0.4)
**Maternal BMI (kg/m^2)**								
<18.5 (Underweight)	785511 (3.0)	134920 (3.4)	127206 (3.3)	118273 (3.1)	111767 (3.0)	100065 (2.8)	96279 (2.6)	97001 (2.6)
18.5-24.9 (Normal)	10609697 (40.3)	1706002 (43.1)	1632309 (42.2)	1566055 (41.2)	1507362 (40.1)	1418785 (39.2)	1404921 (38.3)	1374263 (37.4)
25.0-29.9 (Overweight)	6913607 (26.2)	1001694 (25.3)	989181 (25.6)	988593 (26.0)	988259 (26.3)	964707 (26.7)	988245 (26.9)	992928 (27.0)
30.0-34.9 (Obesity class I)	4004612 (15.2)	549652 (13.9)	553981 (14.3)	562531 (14.8)	572310 (15.2)	569836 (15.7)	591289 (16.1)	605013 (16.5)
35.0-39.9 (Obesity class II)	1994033 (7.6)	266680 (6.7)	271361 (7.0)	278664 (7.3)	285300 (7.6)	286905 (7.9)	298034 (8.1)	307089 (8.4)
≥40.0 (Obesity class III)	1438099 (5.5)	187948 (4.8)	193332 (5.0)	201218 (5.3)	205841 (5.5)	208692 (5.8)	217423 (5.9)	223645 (6.1)
Unknown	600206 (2.3)	109216 (2.8)	97384 (2.5)	86200 (2.3)	86743 (2.3)	70836 (2.0)	73737 (2.0)	76090 (2.1)
**Prepregnancy diabetes**								
No	26053034 (98.9)	3918402 (99.0)	3825603 (99.0)	3762791 (99.0)	3717529 (98.9)	3577494 (98.8)	3624809 (98.8)	3626406 (98.7)
Yes	262287 (1.0)	33856 (0.9)	35365 (0.9)	35770 (0.9)	36603 (1.0)	38105 (1.1)	39752 (1.1)	42836 (1.2)
Unknown	30444 (0.1)	3854 (0.1)	3786 (0.1)	2973 (0.1)	3450 (0.1)	4227 (0.1)	5367 (0.1)	6787 (0.2)
**Gestational diabetes**								
No	24440434 (92.8)	3717103 (94.0)	3615923 (93.6)	3545698 (93.3)	3495054 (93.0)	3333560 (92.1)	3360701 (91.6)	3372395 (91.7)
Yes	1874887 (7.1)	235155 (5.9)	245045 (6.3)	252863 (6.7)	259078 (6.9)	282039 (7.8)	303860 (8.3)	296847 (8.1)
Unknown	30444 (0.1)	3854 (0.1)	3786 (0.1)	2973 (0.1)	3450 (0.1)	4227 (0.1)	5367 (0.1)	6787 (0.2)
**Prepregnancy hypertension**								
No	25714198 (97.6)	3883807 (98.2)	3787896 (98.0)	3720188 (97.9)	3670933 (97.7)	3524031 (97.4)	3564952 (97.1)	3562391 (96.9)
Yes	601123 (2.3)	68451 (1.7)	73072 (1.9)	78373 (2.1)	83199 (2.2)	91568 (2.5)	99609 (2.7)	106851 (2.9)
Unknown	30444 (0.1)	3854 (0.1)	3786 (0.1)	2973 (0.1)	3450 (0.1)	4227 (0.1)	5367 (0.1)	6787 (0.2)
**Gestational hypertension**								
No	24279181 (92.2)	3716148 (93.9)	3612346 (93.5)	3526564 (92.8)	3462061 (92.1)	3311047 (91.5)	3330636 (90.8)	3320379 (90.3)
Yes	2036140 (7.7)	236110 (6.0)	248622 (6.4)	271997 (7.2)	292071 (7.8)	304552 (8.4)	333925 (9.1)	348863 (9.5)
Unknown	30444 (0.1)	3854 (0.1)	3786 (0.1)	2973 (0.1)	3450 (0.1)	4227 (0.1)	5367 (0.1)	6787 (0.2)
**Pregnancy resulted from infertility treatment**								
No	25780418 (97.9)	3883314 (98.2)	3788637 (98.0)	3724584 (98.0)	3677981 (97.9)	3542091 (97.9)	3583856 (97.7)	3579955 (97.4)
Yes	534903 (2.0)	68944 (1.7)	72331 (1.9)	73977 (1.9)	76151 (2.0)	73508 (2.0)	80705 (2.2)	89287 (2.4)
Unknown	30444 (0.1)	3854 (0.1)	3786 (0.1)	2973 (0.1)	3450 (0.1)	4227 (0.1)	5367 (0.1)	6787 (0.2)
**Fertility enhancing drugs/artificial insemination/intrauterine insemination**								
No	300077 (1.1)	34085 (0.9)	36994 (1.0)	39003 (1.0)	43102 (1.1)	42471 (1.2)	48638 (1.3)	55784 (1.5)
Yes	207493 (0.8)	29713 (0.8)	30560 (0.8)	30388 (0.8)	29041 (0.8)	27756 (0.8)	29012 (0.8)	31023 (0.8)
Not applicable	25780418 (97.9)	3883314 (98.2)	3788637 (98.0)	3724584 (98.0)	3677981 (97.9)	3542091 (97.9)	3583856 (97.7)	3579955 (97.4)
Unknown	57777 (0.2)	9000 (0.2)	8563 (0.2)	7559 (0.2)	7458 (0.2)	7508 (0.2)	8422 (0.2)	9267 (0.3)
**Assisted reproductive technology**								
No	164068 (0.6)	24274 (0.6)	24708 (0.6)	24499 (0.6)	23186 (0.6)	22330 (0.6)	22548 (0.6)	22523 (0.6)
Yes	343502 (1.3)	39524 (1.0)	42846 (1.1)	44892 (1.2)	48957 (1.3)	47897 (1.3)	55102 (1.5)	64284 (1.7)
Not applicable	25780418 (97.9)	3883314 (98.2)	3788637 (98.0)	3724584 (98.0)	3677981 (97.9)	3542091 (97.9)	3583856 (97.7)	3579955 (97.4)
Unknown	57777 (0.2)	9000 (0.2)	8563 (0.2)	7559 (0.2)	7458 (0.2)	7508 (0.2)	8422 (0.2)	9267 (0.3)
**Ruptured uterus**								
No	26300495 (99.8)	3949426 (99.8)	3859206 (99.9)	3796665 (99.9)	3752192 (99.9)	3613709 (99.8)	3661519 (99.8)	3667778 (99.8)
Yes	9173 (0.0)	1246 (0.0)	1209 (0.0)	1317 (0.0)	1277 (0.0)	1318 (0.0)	1397 (0.0)	1409 (0.0)

Unknown	36097 (0.1)	5440 (0.1)	4339 (0.1)	3552 (0.1)	4113 (0.1)	4799 (0.1)	7012 (0.2)	6842 (0.2)
**Unplanned hysterectomy**								
No	26297229 (99.8)	3948933 (99.8)	3858598 (99.8)	3796200 (99.9)	3751606 (99.8)	3613340 (99.8)	3661146 (99.8)	3667406 (99.8)
Yes	12439 (0.0)	1739 (0.0)	1817 (0.0)	1782 (0.0)	1863 (0.0)	1687 (0.0)	1770 (0.0)	1781 (0.0)
Unknown	36097 (0.1)	5440 (0.1)	4339 (0.1)	3552 (0.1)	4113 (0.1)	4799 (0.1)	7012 (0.2)	6842 (0.2)
**Infections present and/or treated during current pregnancy**								
No	25544453 (97.0)	3842784 (97.1)	3749957 (97.0)	3688228 (97.0)	3642772 (96.9)	3506342 (96.9)	3553538 (96.8)	3560832 (96.9)
Yes	718064 (2.7)	103587 (2.6)	104698 (2.7)	104732 (2.8)	105110 (2.8)	101838 (2.8)	101352 (2.8)	96747 (2.6)
Unknown	83248 (0.3)	9741 (0.2)	10099 (0.3)	8574 (0.2)	9700 (0.3)	11646 (0.3)	15038 (0.4)	18450 (0.5)
**Obstetric estimate of gestation (completed weeks)**								
10-category								
<20	9131 (0.0)	1368 (0.0)	1390 (0.0)	1316 (0.0)	1360 (0.0)	1317 (0.0)	1162 (0.0)	1218 (0.0)
20-27	163540 (0.6)	25288 (0.6)	24555 (0.6)	23662 (0.6)	23451 (0.6)	21994 (0.6)	22391 (0.6)	22199 (0.6)
28-31	242050 (0.9)	36290 (0.9)	35508 (0.9)	34436 (0.9)	34829 (0.9)	32506 (0.9)	34772 (0.9)	33709 (0.9)
32-33	310950 (1.2)	46056 (1.2)	45083 (1.2)	44753 (1.2)	44327 (1.2)	41720 (1.2)	44805 (1.2)	44206 (1.2)
34-36	1941504 (7.4)	279874 (7.1)	276805 (7.2)	276285 (7.3)	279851 (7.4)	267463 (7.4)	281327 (7.7)	279899 (7.6)
37-38	7180252 (27.3)	1006868 (25.5)	1004020 (26.0)	1007908 (26.5)	1025504 (27.3)	1004922 (27.8)	1054541 (28.7)	1076489 (29.3)
39	9963475 (37.8)	1473390 (37.2)	1436729 (37.2)	1423881 (37.5)	1436342 (38.2)	1403153 (38.8)	1407401 (38.3)	1382579 (37.6)
40	5007958 (19.0)	817084 (20.7)	783674 (20.3)	750993 (19.8)	705082 (18.8)	658797 (18.2)	642533 (17.5)	649795 (17.7)
41	1431990 (5.4)	252370 (6.4)	241521 (6.2)	224336 (5.9)	194811 (5.2)	176447 (4.9)	168868 (4.6)	173637 (4.7)
≥42	74922 (0.3)	14011 (0.4)	12642 (0.3)	11393 (0.3)	9487 (0.3)	8894 (0.2)	9002 (0.2)	9493 (0.3)
Unknown	19993 (0.1)	3513 (0.1)	2827 (0.1)	2571 (0.1)	2538 (0.1)	2613 (0.1)	3126 (0.1)	2805 (0.1)
**Attendant at birth**								
Doctor of Medicine	21027391 (79.8)	3248843 (82.1)	3149540 (81.5)	3074334 (80.9)	3005549 (80.0)	2858534 (79.0)	2853148 (77.7)	2837443 (77.2)
Doctor of Osteopathy	2202073 (8.4)	291083 (7.4)	296405 (7.7)	303441 (8.0)	315275 (8.4)	318281 (8.8)	332203 (9.1)	345385 (9.4)
Certified Nurse Midwife/Certified Midwife	2590425 (9.8)	348745 (8.8)	352405 (9.1)	357626 (9.4)	368745 (9.8)	373244 (10.3)	390429 (10.6)	399231 (10.9)
Other Midwife	254825 (1.0)	31653 (0.8)	31038 (0.8)	31317 (0.8)	33391 (0.9)	38012 (1.1)	44316 (1.2)	45098 (1.2)
Other	253753 (1.0)	33375 (0.8)	32514 (0.8)	32308 (0.8)	32239 (0.9)	29833 (0.8)	47449 (1.3)	46035 (1.3)
Unknown	17298 (0.1)	2413 (0.1)	2852 (0.1)	2508 (0.1)	2383 (0.1)	1922 (0.1)	2383 (0.1)	2837 (0.1)
**Induction of labor**								
No	18753375 (71.2)	2984308 (75.4)	2868959 (74.2)	2769934 (72.9)	2654493 (70.6)	2482742 (68.6)	2490462 (67.9)	2502477 (68.1)
Yes	7572416 (28.7)	969102 (24.5)	993349 (25.7)	1027392 (27.0)	1101127 (29.3)	1134816 (31.4)	1175974 (32.0)	1170656 (31.8)
Unknown	19974 (0.1)	2702 (0.1)	2446 (0.1)	4208 (0.1)	1962 (0.1)	2268 (0.1)	3492 (0.1)	2896 (0.1)
**Augmentation of labor**								
No	20699589 (78.6)	3125018 (79.0)	3040925 (78.7)	2981699 (78.4)	2940549 (78.3)	2831291 (78.2)	2871704 (78.2)	2908403 (79.1)
Yes	5628986 (21.4)	828392 (20.9)	821383 (21.3)	818411 (21.5)	815071 (21.7)	786267 (21.7)	794732 (21.7)	764730 (20.8)
Unknown	17190 (0.1)	2702 (0.1)	2446 (0.1)	1424 (0.0)	1962 (0.1)	2268 (0.1)	3492 (0.1)	2896 (0.1)
**Steroids (glucocorticoids) for fetal lung maturation received by the mother prior to delivery**								
No	25410946 (96.5)	3859351 (97.6)	3742005 (96.8)	3668061 (96.5)	3612253 (96.1)	3482391 (96.2)	3520145 (95.9)	3526740 (95.9)
Yes	917629 (3.5)	94059 (2.4)	120303 (3.1)	132049 (3.5)	143367 (3.8)	135167 (3.7)	146291 (4.0)	146393 (4.0)
Unknown	17190 (0.1)	2702 (0.1)	2446 (0.1)	1424 (0.0)	1962 (0.1)	2268 (0.1)	3492 (0.1)	2896 (0.1)

**Antibiotics received by the mother during labor**								
No	19684798 (74.7)	2975182 (75.2)	2897479 (75.0)	2841760 (74.8)	2811913 (74.8)	2691563 (74.4)	2724363 (74.2)	2742538 (74.6)
Yes	6643777 (25.2)	978228 (24.7)	964829 (25.0)	958350 (25.2)	943707 (25.1)	925995 (25.6)	942073 (25.7)	930595 (25.3)
Unknown	17190 (0.1)	2702 (0.1)	2446 (0.1)	1424 (0.0)	1962 (0.1)	2268 (0.1)	3492 (0.1)	2896 (0.1)
**Clinical chorioamnionitis diagnosed during labor or maternal temperature ≥38°C**								
No	25910446 (98.3)	3892741 (98.4)	3801144 (98.4)	3739146 (98.4)	3695747 (98.4)	3560540 (98.4)	3608280 (98.3)	3612848 (98.3)
Yes	418129 (1.6)	60669 (1.5)	61164 (1.6)	60964 (1.6)	59873 (1.6)	57018 (1.6)	58156 (1.6)	60285 (1.6)
Unknown	17190 (0.1)	2702 (0.1)	2446 (0.1)	1424 (0.0)	1962 (0.1)	2268 (0.1)	3492 (0.1)	2896 (0.1)
**Epidural or spinal anesthesia during labor**								
No	6371155 (24.2)	1045639 (26.4)	997723 (25.8)	946260 (24.9)	900106 (24.0)	825261 (22.8)	820904 (22.4)	835262 (22.7)
Yes	19957420 (75.8)	2907771 (73.5)	2864585 (74.1)	2853850 (75.1)	2855514 (76.0)	2792297 (77.1)	2845532 (77.5)	2837871 (77.2)
Unknown	17190 (0.1)	2702 (0.1)	2446 (0.1)	1424 (0.0)	1962 (0.1)	2268 (0.1)	3492 (0.1)	2896 (0.1)
**Method of delivery**								
6-category								
Vaginal (excludes vaginal after previous C-section)	17352111 (65.9)	2613713 (66.1)	2548266 (65.9)	2507707 (66.0)	2482944 (66.1)	2386516 (65.9)	2407506 (65.6)	2405459 (65.4)
Vaginal after previous C-section	551105 (2.1)	75407 (1.9)	76467 (2.0)	79017 (2.1)	80289 (2.1)	77866 (2.2)	79644 (2.2)	82415 (2.2)
Primary C-section	4882617 (18.5)	730480 (18.5)	712699 (18.4)	695090 (18.3)	685861 (18.3)	667646 (18.4)	692655 (18.9)	698186 (19.0)
Repeat C-section	3518455 (13.4)	530775 (13.4)	521982 (13.5)	515780 (13.6)	503358 (13.4)	482321 (13.3)	482820 (13.2)	481419 (13.1)
Vaginal (unknown if previous C-section)	19219 (0.1)	2403 (0.1)	2407 (0.1)	1810 (0.0)	2296 (0.1)	2619 (0.1)	3357 (0.1)	4327 (0.1)
C-section (unknown if previous C-section)	5461 (0.0)	694 (0.0)	681 (0.0)	502 (0.0)	499 (0.0)	705 (0.0)	970 (0.0)	1410 (0.0)
Unknown	16797 (0.1)	2640 (0.1)	2252 (0.1)	1628 (0.0)	2335 (0.1)	2153 (0.1)	2976 (0.1)	2813 (0.1)
**Fetal presentation at birth**								
Cephalic	24944298 (94.7)	3728594 (94.2)	3659937 (94.7)	3605803 (94.9)	3566399 (94.9)	3435565 (94.9)	3474107 (94.7)	3473893 (94.5)
Breech	1064712 (4.0)	152846 (3.9)	151554 (3.9)	150385 (4.0)	150678 (4.0)	146907 (4.1)	153927 (4.2)	158415 (4.3)
Other	246309 (0.9)	51709 (1.3)	37373 (1.0)	32606 (0.9)	30135 (0.8)	30064 (0.8)	31084 (0.8)	33338 (0.9)
Unknown	90446 (0.3)	22963 (0.6)	15890 (0.4)	12740 (0.3)	10370 (0.3)	7290 (0.2)	10810 (0.3)	10383 (0.3)
**Final route and method of delivery**								
Vaginal/Spontaneous	17126915 (65.0)	2568828 (64.9)	2507646 (64.9)	2472414 (65.0)	2451304 (65.2)	2356621 (65.1)	2381810 (64.9)	2388292 (65.0)
Vaginal/Forceps	133911 (0.5)	21706 (0.5)	20324 (0.5)	19478 (0.5)	19034 (0.5)	18325 (0.5)	18160 (0.5)	16884 (0.5)
Vaginal/Vacuum	661609 (2.5)	100989 (2.6)	99170 (2.6)	96642 (2.5)	95191 (2.5)	92055 (2.5)	90537 (2.5)	87025 (2.4)
Cesarean	8406533 (31.9)	1261949 (31.9)	1235362 (32.0)	1211372 (31.9)	1189718 (31.7)	1150672 (31.8)	1176445 (32.1)	1181015 (32.1)
Unknown	16797 (0.1)	2640 (0.1)	2252 (0.1)	1628 (0.0)	2335 (0.1)	2153 (0.1)	2976 (0.1)	2813 (0.1)
**A trial of labor attempted if cesarean**								
No	6088980 (23.1)	928062 (23.5)	906060 (23.4)	886652 (23.3)	858314 (22.8)	822556 (22.7)	841507 (22.9)	845829 (23.0)
Yes	2237415 (8.5)	319418 (8.1)	318946 (8.3)	318477 (8.4)	318293 (8.5)	316100 (8.7)	321747 (8.8)	324434 (8.8)
Not applicable	17922435 (68.0)	2691523 (68.0)	2627140 (68.0)	2588534 (68.1)	2565529 (68.3)	2467001 (68.2)	2490507 (67.9)	2492201 (67.8)
Unknown	96935 (0.4)	17109 (0.4)	12608 (0.3)	7871 (0.2)	15446 (0.4)	14169 (0.4)	16167 (0.4)	13565 (0.4)
**Birthweight (g)**								
3-category								

227-1499	362420 (1.4)	55189 (1.4)	54191 (1.4)	52244 (1.4)	51857 (1.4)	48374 (1.3)	50611 (1.4)	49954 (1.4)
1500–2499	1831984 (7.0)	267244 (6.8)	265236 (6.9)	262104 (6.9)	260065 (6.9)	249632 (6.9)	261753 (7.1)	265950 (7.2)
2500-8165	24127474 (91.6)	3629136 (91.7)	3542294 (91.7)	3484226 (91.7)	3441893 (91.6)	3318743 (91.7)	3354141 (91.4)	3357041 (91.3)
Unknown	23887 (0.1)	4543 (0.1)	3033 (0.1)	2960 (0.1)	3767 (0.1)	3077 (0.1)	3423 (0.1)	3084 (0.1)
11-category								
227-499	36027 (0.1)	5722 (0.1)	5478 (0.1)	5313 (0.1)	5198 (0.1)	4796 (0.1)	4632 (0.1)	4888 (0.1)
500–999	132695 (0.5)	20349 (0.5)	20006 (0.5)	18994 (0.5)	18874 (0.5)	17826 (0.5)	18529 (0.5)	18117 (0.5)
1000-1499	193698 (0.7)	29118 (0.7)	28707 (0.7)	27937 (0.7)	27785 (0.7)	25752 (0.7)	27450 (0.7)	26949 (0.7)
1500–1999	429806 (1.6)	62970 (1.6)	62700 (1.6)	61615 (1.6)	61318 (1.6)	58243 (1.6)	61676 (1.7)	61284 (1.7)
2000–2499	1402178 (5.3)	204274 (5.2)	202536 (5.2)	200489 (5.3)	198747 (5.3)	191389 (5.3)	200077 (5.5)	204666 (5.6)
2500–2999	5005666 (19.0)	732657 (18.5)	723001 (18.7)	712329 (18.7)	715844 (19.1)	692833 (19.1)	705948 (19.2)	723054 (19.7)
3000–3499	10234775 (38.8)	1532159 (38.7)	1497093 (38.7)	1472895 (38.7)	1466872 (39.0)	1408485 (38.9)	1424643 (38.8)	1432628 (39.0)
3500–3999	6892377 (26.2)	1051062 (26.6)	1020909 (26.4)	1004311 (26.4)	979132 (26.1)	946792 (26.2)	950676 (25.9)	939495 (25.6)
4000–4499	1730816 (6.6)	270431 (6.8)	260497 (6.7)	255184 (6.7)	243620 (6.5)	235317 (6.5)	237254 (6.5)	228513 (6.2)
4500–4999	236622 (0.9)	38322 (1.0)	36554 (0.9)	35492 (0.9)	32764 (0.9)	31528 (0.9)	31995 (0.9)	29967 (0.8)
5000–8165	27218 (0.1)	4505 (0.1)	4240 (0.1)	4015 (0.1)	3661 (0.1)	3788 (0.1)	3625 (0.1)	3384 (0.1)
Unknown	23887 (0.1)	4543 (0.1)	3033 (0.1)	2960 (0.1)	3767 (0.1)	3077 (0.1)	3423 (0.1)	3084 (0.1)
**Assisted ventilation required immediately following delivery**								
No	25033017 (95.0)	3796628 (96.0)	3700854 (95.8)	3627095 (95.4)	3570466 (95.0)	3427248 (94.7)	3457283 (94.2)	3453443 (93.9)
Yes	1281064 (4.9)	155052 (3.9)	160428 (4.2)	170986 (4.5)	183622 (4.9)	188673 (5.2)	206826 (5.6)	215477 (5.9)
Unknown	31684 (0.1)	4432 (0.1)	3472 (0.1)	3453 (0.1)	3494 (0.1)	3905 (0.1)	5819 (0.2)	7109 (0.2)
**Assisted ventilation required for more than six hours**								
No	25876580 (98.2)	3897593 (98.5)	3804789 (98.4)	3739507 (98.4)	3692075 (98.3)	3551565 (98.1)	3593103 (97.9)	3597948 (97.9)
Yes	437501 (1.7)	54087 (1.4)	56493 (1.5)	58574 (1.5)	62013 (1.7)	64356 (1.8)	71006 (1.9)	70972 (1.9)
Unknown	31684 (0.1)	4432 (0.1)	3472 (0.1)	3453 (0.1)	3494 (0.1)	3905 (0.1)	5819 (0.2)	7109 (0.2)
**NICU admission**								
No	23887230 (90.7)	3606610 (91.2)	3514742 (90.9)	3450677 (90.8)	3404120 (90.6)	3278378 (90.6)	3311935 (90.2)	3320768 (90.3)
Yes	2426851 (9.2)	345070 (8.7)	346540 (9.0)	347404 (9.1)	349968 (9.3)	337543 (9.3)	352174 (9.6)	348152 (9.5)
Unknown	31684 (0.1)	4432 (0.1)	3472 (0.1)	3453 (0.1)	3494 (0.1)	3905 (0.1)	5819 (0.2)	7109 (0.2)
**Newborn given surfactant replacement therapy**								
No	26186480 (99.4)	3932583 (99.4)	3842897 (99.4)	3780358 (99.4)	3735985 (99.4)	3598472 (99.4)	3645332 (99.3)	3650853 (99.3)
Yes	127601 (0.5)	19097 (0.5)	18385 (0.5)	17723 (0.5)	18103 (0.5)	17449 (0.5)	18777 (0.5)	18067 (0.5)
Unknown	31684 (0.1)	4432 (0.1)	3472 (0.1)	3453 (0.1)	3494 (0.1)	3905 (0.1)	5819 (0.2)	7109 (0.2)
**Antibiotics received by the newborn for suspected neonatal sepsis**								
No	25765256 (97.8)	3859363 (97.6)	3772328 (97.6)	3716947 (97.8)	3677769 (97.9)	3545037 (97.9)	3593146 (97.9)	3600666 (97.9)
Yes	548825 (2.1)	92317 (2.3)	88954 (2.3)	81134 (2.1)	76319 (2.0)	70884 (2.0)	70963 (1.9)	68254 (1.9)
Unknown	31684 (0.1)	4432 (0.1)	3472 (0.1)	3453 (0.1)	3494 (0.1)	3905 (0.1)	5819 (0.2)	7109 (0.2)
**Seizure or serious neurologic dysfunction**								
No	26304797 (99.8)	3950285 (99.9)	3859934 (99.9)	3796755 (99.9)	3752824 (99.9)	3614674 (99.9)	3662742 (99.8)	3667583 (99.8)
Yes	9284 (0.0)	1395 (0.0)	1348 (0.0)	1326 (0.0)	1264 (0.0)	1247 (0.0)	1367 (0.0)	1337 (0.0)
Unknown	31684 (0.1)	4432 (0.1)	3472 (0.1)	3453 (0.1)	3494 (0.1)	3905 (0.1)	5819 (0.2)	7109 (0.2)

Note: Details about each variable are presented in Supplementary methods.
Abbreviations: GED, General Educational Development; WIC, the Special Supplemental Nutrition Program for Women, Infants, and Children (which is administered by the U.S. Department of Agriculture and intended to help low-income pregnant women, infants, and children through age 5 receive proper nutrition by providing vouchers for food, nutrition counseling, health care screenings and referrals); BMI, body mass index; NICU, neonatal intensive care unit.

Supplemental Table S4 Reported prevalence of the study outcomes by birth year

Clinical outcome	Birth year	No. at risk	No. events	Reported prevalence (95% confidence interval)	Odds ratio (95% confidence interval)	
					Crude	Adjusted
Congenital anomalies	2016	3949284	13791	3.49 (3.43-3.55) [/1000]	1.03 (1.01-1.06)	0.99 (0.97-1.02)
Congenital anomalies	2017	3859817	13405	3.47 (3.41-3.53) [/1000]	1.03 (1.00-1.05)	1.04 (1.01-1.06)
Congenital anomalies	2018	3795427	13314	3.51 (3.45-3.57) [/1000]	1.04 (1.01-1.06)	1.04 (1.02-1.07)
Congenital anomalies	2019	3752114	12690	3.38 (3.32-3.44) [/1000]	1 (Reference)	1 (Reference)
Congenital anomalies	2020	3614661	12390	3.43 (3.37-3.49) [/1000]	1.01 (0.99-1.04)	1.01 (0.98-1.03)
Congenital anomalies	2021	3662380	12636	3.45 (3.39-3.51) [/1000]	1.02 (1.00-1.05)	1.00 (0.98-1.03)
Congenital anomalies	2022	3668528	12283	3.35 (3.29-3.41) [/1000]	0.99 (0.97-1.01)	0.97 (0.95-0.99)
Anencephaly	2016	3949284	366	9.27 (8.34-10.27) [/100,000]	1.04 (0.90-1.21)	0.98 (0.84-1.13)
Anencephaly	2017	3859817	432	11.19 (10.16-12.30) [/100,000]	1.26 (1.09-1.45)	1.26 (1.09-1.45)
Anencephaly	2018	3795427	354	9.33 (8.38-10.35) [/100,000]	1.05 (0.90-1.22)	1.05 (0.90-1.22)
Anencephaly	2019	3752114	333	8.87 (7.95-9.88) [/100,000]	1 (Reference)	1 (Reference)
Anencephaly	2020	3614661	332	9.18 (8.22-10.23) [/100,000]	1.03 (0.89-1.20)	1.03 (0.89-1.20)
Anencephaly	2021	3662380	310	8.46 (7.55-9.46) [/100,000]	0.95 (0.82-1.11)	0.96 (0.82-1.12)
Anencephaly	2022	3668528	387	10.55 (9.52-11.65) [/100,000]	1.19 (1.03-1.38)	1.19 (1.02-1.37)
Meningomyelocele/Spina bifida	2016	3949284	548	13.88 (12.74-15.09) [/100,000]	0.97 (0.86-1.09)	0.96 (0.85-1.08)
Meningomyelocele/Spina bifida	2017	3859817	596	15.44 (14.23-16.73) [/100,000]	1.07 (0.96-1.21)	1.09 (0.97-1.23)
Meningomyelocele/Spina bifida	2018	3795427	555	14.62 (13.43-15.89) [/100,000]	1.02 (0.90-1.15)	1.02 (0.91-1.15)
Meningomyelocele/Spina bifida	2019	3752114	539	14.37 (13.18-15.63) [/100,000]	1 (Reference)	1 (Reference)
Meningomyelocele/Spina bifida	2020	3614661	490	13.56 (12.38-14.81) [/100,000]	0.94 (0.83-1.07)	0.93 (0.83-1.06)
Meningomyelocele/Spina bifida	2021	3662380	498	13.60 (12.43-14.85) [/100,000]	0.95 (0.84-1.07)	0.92 (0.81-1.04)
Meningomyelocele/Spina bifida	2022	3668528	499	13.60 (12.43-14.85) [/100,000]	0.95 (0.84-1.07)	0.92 (0.81-1.03)
Cyanotic congenital heart disease	2016	3949284	2200	55.71 (53.40-58.08) [/100,000]	0.94 (0.89-1.00)	0.89 (0.84-0.95)
Cyanotic congenital heart disease	2017	3859817	2130	55.18 (52.87-57.58) [/100,000]	0.93 (0.88-0.99)	0.95 (0.89-1.01)
Cyanotic congenital heart disease	2018	3795427	2216	58.39 (55.98-60.87) [/100,000]	0.99 (0.93-1.05)	0.99 (0.94-1.05)
Cyanotic congenital heart disease	2019	3752114	2221	59.19 (56.76-61.71) [/100,000]	1 (Reference)	1 (Reference)
Cyanotic congenital heart disease	2020	3614661	2288	63.30 (60.73-65.94) [/100,000]	1.07 (1.01-1.13)	1.06 (1.00-1.12)
Cyanotic congenital heart disease	2021	3662380	2490	67.99 (65.34-70.71) [/100,000]	1.15 (1.08-1.22)	1.12 (1.05-1.18)
Cyanotic congenital heart disease	2022	3668528	2426	66.13 (63.53-68.81) [/100,000]	1.12 (1.05-1.18)	1.08 (1.02-1.14)
Congenital diaphragmatic hernia	2016	3949284	501	12.69 (11.60-13.85) [/100,000]	1.01 (0.89-1.14)	0.98 (0.86-1.11)
Congenital diaphragmatic hernia	2017	3859817	471	12.20 (11.13-13.36) [/100,000]	0.97 (0.85-1.10)	0.99 (0.87-1.12)
Congenital diaphragmatic hernia	2018	3795427	472	12.44 (11.34-13.61) [/100,000]	0.99 (0.87-1.12)	1.00 (0.88-1.13)
Congenital diaphragmatic hernia	2019	3752114	472	12.58 (11.47-13.77) [/100,000]	1 (Reference)	1 (Reference)
Congenital diaphragmatic hernia	2020	3614661	469	12.97 (11.83-14.20) [/100,000]	1.03 (0.91-1.17)	1.02 (0.90-1.16)
Congenital diaphragmatic hernia	2021	3662380	462	12.61 (11.49-13.82) [/100,000]	1.00 (0.88-1.14)	0.97 (0.86-1.11)
Congenital diaphragmatic hernia	2022	3668528	486	13.25 (12.10-14.48) [/100,000]	1.05 (0.93-1.20)	1.02 (0.89-1.15)
Omphalocele	2016	3949284	404	10.23 (9.26-11.28) [/100,000]	0.97 (0.84-1.11)	0.97 (0.84-1.11)
Omphalocele	2017	3859817	400	10.36 (9.37-11.43) [/100,000]	0.98 (0.85-1.13)	0.99 (0.87-1.14)
Omphalocele	2018	3795427	408	10.75 (9.73-11.85) [/100,000]	1.02 (0.88-1.17)	1.02 (0.89-1.17)
Omphalocele	2019	3752114	397	10.58 (9.57-11.67) [/100,000]	1 (Reference)	1 (Reference)
Omphalocele	2020	3614661	336	9.30 (8.33-10.34) [/100,000]	0.88 (0.76-1.02)	0.87 (0.75-1.01)
Omphalocele	2021	3662380	376	10.27 (9.25-11.36) [/100,000]	0.97 (0.84-1.12)	0.95 (0.82-1.09)
Omphalocele	2022	3668528	350	9.54 (8.57-10.59) [/100,000]	0.90 (0.78-1.04)	0.88 (0.76-1.02)
Gastroschisis	2016	3949284	959	24.28 (22.77-25.87) [/100,000]	1.10 (1.00-1.21)	1.06 (0.97-1.17)

Gastroschisis	2017	3859817	926	23.99 (22.47-25.59) [/100,000]	1.09 (0.99-1.19)	1.07 (0.97-1.17)
Gastroschisis	2018	3795427	830	21.87 (20.41-23.41) [/100,000]	0.99 (0.90-1.09)	0.98 (0.89-1.08)
Gastroschisis	2019	3752114	829	22.09 (20.62-23.65) [/100,000]	1 (Reference)	1 (Reference)
Gastroschisis	2020	3614661	746	20.64 (19.18-22.17) [/100,000]	0.93 (0.85-1.03)	0.93 (0.85-1.03)
Gastroschisis	2021	3662380	689	18.81 (17.43-20.27) [/100,000]	0.85 (0.77-0.94)	0.86 (0.78-0.96)
Gastroschisis	2022	3668528	586	15.97 (14.71-17.32) [/100,000]	0.72 (0.65-0.80)	0.74 (0.66-0.82)
Limb reduction defect	2016	3949284	549	13.90 (12.76-15.11) [/100,000]	1.18 (1.04-1.34)	1.10 (0.97-1.25)
Limb reduction defect	2017	3859817	526	13.63 (12.49-14.84) [/100,000]	1.16 (1.02-1.32)	1.16 (1.02-1.32)
Limb reduction defect	2018	3795427	487	12.83 (11.72-14.02) [/100,000]	1.09 (0.96-1.24)	1.09 (0.96-1.24)
Limb reduction defect	2019	3752114	441	11.75 (10.68-12.90) [/100,000]	1 (Reference)	1 (Reference)
Limb reduction defect	2020	3614661	440	12.17 (11.06-13.36) [/100,000]	1.04 (0.91-1.18)	1.03 (0.91-1.18)
Limb reduction defect	2021	3662380	427	11.66 (10.58-12.82) [/100,000]	0.99 (0.87-1.13)	0.99 (0.87-1.13)
Limb reduction defect	2022	3668528	424	11.56 (10.48-12.71) [/100,000]	0.98 (0.86-1.12)	0.98 (0.86-1.13)
Cleft Lip with or without Cleft Palate	2016	3949284	2111	53.45 (51.20-55.78) [/100,000]	1.09 (1.02-1.16)	1.05 (0.99-1.12)
Cleft Lip with or without Cleft Palate	2017	3859817	1993	51.63 (49.39-53.95) [/100,000]	1.05 (0.99-1.12)	1.05 (0.99-1.12)
Cleft Lip with or without Cleft Palate	2018	3795427	2054	54.12 (51.80-56.51) [/100,000]	1.10 (1.04-1.18)	1.10 (1.04-1.17)
Cleft Lip with or without Cleft Palate	2019	3752114	1840	49.04 (46.82-51.33) [/100,000]	1 (Reference)	1 (Reference)
Cleft Lip with or without Cleft Palate	2020	3614661	1792	49.58 (47.31-51.93) [/100,000]	1.01 (0.95-1.08)	1.01 (0.95-1.08)
Cleft Lip with or without Cleft Palate	2021	3662380	1917	52.34 (50.03-54.74) [/100,000]	1.07 (1.00-1.14)	1.06 (1.00-1.13)
Cleft Lip with or without Cleft Palate	2022	3668528	1862	50.76 (48.48-53.11) [/100,000]	1.04 (0.97-1.10)	1.03 (0.97-1.10)
Cleft Palate alone	2016	3949284	915	23.17 (21.69-24.72) [/100,000]	1.00 (0.92-1.10)	0.95 (0.86-1.04)
Cleft Palate alone	2017	3859817	928	24.04 (22.52-25.64) [/100,000]	1.04 (0.95-1.14)	1.04 (0.95-1.15)
Cleft Palate alone	2018	3795427	920	24.24 (22.70-25.86) [/100,000]	1.05 (0.96-1.15)	1.05 (0.96-1.15)
Cleft Palate alone	2019	3752114	865	23.05 (21.54-24.64) [/100,000]	1 (Reference)	1 (Reference)
Cleft Palate alone	2020	3614661	846	23.40 (21.85-25.04) [/100,000]	1.02 (0.92-1.12)	1.01 (0.92-1.11)
Cleft Palate alone	2021	3662380	847	23.13 (21.60-24.74) [/100,000]	1.00 (0.91-1.10)	1.00 (0.91-1.10)
Cleft Palate alone	2022	3668528	821	22.38 (20.87-23.96) [/100,000]	0.97 (0.88-1.07)	0.97 (0.88-1.07)
Down syndrome	2016	3949284	2168	54.90 (52.61-57.26) [/100,000]	1.01 (0.96-1.08)	1.01 (0.95-1.08)
Down syndrome	2017	3859817	2044	52.96 (50.69-55.30) [/100,000]	0.98 (0.92-1.04)	1.01 (0.95-1.08)
Down syndrome	2018	3795427	2108	55.54 (53.20-57.96) [/100,000]	1.03 (0.97-1.09)	1.04 (0.98-1.11)
Down syndrome	2019	3752114	2030	54.10 (51.78-56.51) [/100,000]	1 (Reference)	1 (Reference)
Down syndrome	2020	3614661	1954	54.06 (51.69-56.51) [/100,000]	1.00 (0.94-1.06)	0.98 (0.92-1.04)
Down syndrome	2021	3662380	1903	51.96 (49.65-54.35) [/100,000]	0.96 (0.90-1.02)	0.93 (0.87-0.99)
Down syndrome	2022	3668528	1861	50.73 (48.45-53.09) [/100,000]	0.94 (0.88-1.00)	0.89 (0.83-0.94)
Suspected chromosomal disorder	2016	3949284	1700	43.05 (41.02-45.14) [/100,000]	0.97 (0.91-1.04)	0.94 (0.88-1.00)
Suspected chromosomal disorder	2017	3859817	1658	42.96 (40.91-45.07) [/100,000]	0.97 (0.90-1.03)	0.99 (0.92-1.05)
Suspected chromosomal disorder	2018	3795427	1726	45.48 (43.36-47.67) [/100,000]	1.02 (0.96-1.09)	1.03 (0.96-1.10)
Suspected chromosomal disorder	2019	3752114	1667	44.43 (42.32-46.61) [/100,000]	1 (Reference)	1 (Reference)
Suspected chromosomal disorder	2020	3614661	1652	45.70 (43.53-47.96) [/100,000]	1.03 (0.96-1.10)	1.02 (0.95-1.09)
Suspected chromosomal disorder	2021	3662380	1731	47.26 (45.06-49.54) [/100,000]	1.06 (0.99-1.14)	1.04 (0.97-1.11)
Suspected chromosomal disorder	2022	3668528	1595	43.48 (41.37-45.66) [/100,000]	0.98 (0.91-1.05)	0.95 (0.88-1.02)
Hypospadias	2016	3949284	2456	62.19 (59.75-64.70) [/100,000]	1.13 (1.06-1.20)	1.04 (0.98-1.10)
Hypospadias	2017	3859817	2317	60.03 (57.61-62.52) [/100,000]	1.09 (1.03-1.15)	1.09 (1.03-1.16)
Hypospadias	2018	3795427	2238	58.97 (56.55-61.46) [/100,000]	1.07 (1.01-1.13)	1.07 (1.01-1.13)
Hypospadias	2019	3752114	2070	55.17 (52.82-57.60) [/100,000]	1 (Reference)	1 (Reference)
Hypospadias	2020	3614661	2008	55.55 (53.15-58.03) [/100,000]	1.01 (0.95-1.07)	1.00 (0.94-1.07)

Hypospadias	2021	3662380	1956	53.41 (51.07-55.83) [/100,000]	0.97 (0.91-1.03)	0.96 (0.90-1.02)
Hypospadias	2022	3668528	1910	52.06 (49.76-54.45) [/100,000]	0.94 (0.89-1.00)	0.94 (0.89-1.00)
Transferred within 24 hours of delivery	2016	3953244	44602	1.13 (1.12-1.14) [/100]	0.96 (0.94-0.97)	0.94 (0.92-0.95)
Transferred within 24 hours of delivery	2017	3862267	44502	1.15 (1.14-1.16) [/100]	0.98 (0.97-0.99)	0.97 (0.96-0.99)
Transferred within 24 hours of delivery	2018	3799287	44523	1.17 (1.16-1.18) [/100]	0.99 (0.98-1.01)	0.99 (0.98-1.01)
Transferred within 24 hours of delivery	2019	3753809	44212	1.18 (1.17-1.19) [/100]	1 (Reference)	1 (Reference)
Transferred within 24 hours of delivery	2020	3615356	40640	1.12 (1.11-1.14) [/100]	0.95 (0.94-0.97)	0.96 (0.94-0.97)
Transferred within 24 hours of delivery	2021	3662837	42909	1.17 (1.16-1.18) [/100]	0.99 (0.98-1.01)	1.00 (0.99-1.02)
Transferred within 24 hours of delivery	2022	3667385	42283	1.15 (1.14-1.16) [/100]	0.98 (0.97-0.99)	0.99 (0.98-1.01)
Death before birth-certificate registration	2016	3949893	10860	2.75 (2.70-2.80) [/1000]	1.15 (1.12-1.18)	1.12 (1.09-1.15)
Death before birth-certificate registration	2017	3858314	10218	2.65 (2.60-2.70) [/1000]	1.11 (1.08-1.14)	1.12 (1.09-1.16)
Death before birth-certificate registration	2018	3794365	9683	2.55 (2.50-2.60) [/1000]	1.07 (1.04-1.10)	1.08 (1.05-1.11)
Death before birth-certificate registration	2019	3751303	8983	2.39 (2.35-2.44) [/1000]	1 (Reference)	1 (Reference)
Death before birth-certificate registration	2020	3613818	8323	2.30 (2.25-2.35) [/1000]	0.96 (0.93-0.99)	0.96 (0.93-0.99)
Death before birth-certificate registration	2021	3664198	7925	2.16 (2.12-2.21) [/1000]	0.90 (0.88-0.93)	0.90 (0.87-0.93)
Death before birth-certificate registration	2022	3670800	8145	2.22 (2.17-2.27) [/1000]	0.93 (0.90-0.95)	0.91 (0.89-0.94)

Note: Details about each variable are presented in Supplementary methods. Infants with missing outcomes were excluded from the analyses of the specific outcomes. The adjusted odds ratios were adjusted for maternal age, maternal residence, maternal ethnicity/race, maternal education attainment, principal source of payment for current delivery, and marital status.

Supplemental Table S5 Reported prevalence of the study outcomes by birth year (subgroup analysis, only infants delivered at ≥37 weeks' gestation)

Clinical outcome	Birth year	No. at risk	No. events	Reported prevalence (95% confidence interval)	Odds ratio (95% confidence interval)	
					Crude	Adjusted
Congenital anomalies	2016	3558996	10105	2.84 (2.78-2.90) [/1000]	1.03 (1.00-1.06)	0.99 (0.96-1.02)
Congenital anomalies	2017	3475385	9794	2.82 (2.76-2.87) [/1000]	1.03 (1.00-1.06)	1.04 (1.01-1.07)
Congenital anomalies	2018	3414338	9787	2.87 (2.81-2.92) [/1000]	1.04 (1.01-1.07)	1.05 (1.02-1.08)
Congenital anomalies	2019	3367677	9254	2.75 (2.69-2.80) [/1000]	1 (Reference)	1 (Reference)
Congenital anomalies	2020	3248797	9093	2.80 (2.74-2.86) [/1000]	1.02 (0.99-1.05)	1.01 (0.98-1.04)
Congenital anomalies	2021	3277276	9151	2.79 (2.74-2.85) [/1000]	1.02 (0.99-1.05)	1.00 (0.97-1.03)
Congenital anomalies	2022	3286661	9023	2.75 (2.69-2.80) [/1000]	1.00 (0.97-1.03)	0.98 (0.95-1.01)
Anencephaly	2016	3558996	221	6.21 (5.42-7.08) [/100,000]	1.05 (0.87-1.27)	0.98 (0.81-1.19)
Anencephaly	2017	3475385	248	7.14 (6.28-8.08) [/100,000]	1.21 (1.00-1.46)	1.21 (1.00-1.45)
Anencephaly	2018	3414338	219	6.41 (5.59-7.32) [/100,000]	1.09 (0.90-1.32)	1.09 (0.90-1.32)
Anencephaly	2019	3367677	199	5.91 (5.12-6.79) [/100,000]	1 (Reference)	1 (Reference)
Anencephaly	2020	3248797	197	6.06 (5.25-6.97) [/100,000]	1.03 (0.84-1.25)	1.03 (0.84-1.25)
Anencephaly	2021	3277276	188	5.74 (4.95-6.62) [/100,000]	0.97 (0.80-1.18)	0.97 (0.80-1.19)
Anencephaly	2022	3286661	239	7.27 (6.38-8.25) [/100,000]	1.23 (1.02-1.49)	1.23 (1.02-1.48)
Meningomyelocele/Spina bifida	2016	3558996	395	11.10 (10.03-12.25) [/100,000]	1.03 (0.89-1.19)	1.01 (0.88-1.17)
Meningomyelocele/Spina bifida	2017	3475385	407	11.71 (10.60-12.91) [/100,000]	1.09 (0.94-1.25)	1.10 (0.95-1.27)
Meningomyelocele/Spina bifida	2018	3414338	375	10.98 (9.90-12.15) [/100,000]	1.02 (0.88-1.18)	1.02 (0.89-1.18)
Meningomyelocele/Spina bifida	2019	3367677	363	10.78 (9.70-11.95) [/100,000]	1 (Reference)	1 (Reference)
Meningomyelocele/Spina bifida	2020	3248797	322	9.91 (8.86-11.06) [/100,000]	0.92 (0.79-1.07)	0.91 (0.78-1.06)
Meningomyelocele/Spina bifida	2021	3277276	337	10.28 (9.21-11.44) [/100,000]	0.95 (0.82-1.11)	0.93 (0.80-1.08)
Meningomyelocele/Spina bifida	2022	3286661	295	8.98 (7.98-10.06) [/100,000]	0.83 (0.71-0.97)	0.81 (0.69-0.94)
Cyanotic congenital heart disease	2016	3558996	1615	45.38 (43.19-47.65) [/100,000]	0.93 (0.87-0.99)	0.88 (0.82-0.94)
Cyanotic congenital heart disease	2017	3475385	1576	45.35 (43.14-47.64) [/100,000]	0.93 (0.86-0.99)	0.94 (0.88-1.01)
Cyanotic congenital heart disease	2018	3414338	1659	48.59 (46.28-50.98) [/100,000]	0.99 (0.93-1.06)	1.00 (0.93-1.07)
Cyanotic congenital heart disease	2019	3367677	1649	48.97 (46.63-51.39) [/100,000]	1 (Reference)	1 (Reference)
Cyanotic congenital heart disease	2020	3248797	1725	53.10 (50.62-55.66) [/100,000]	1.08 (1.01-1.16)	1.07 (1.00-1.15)
Cyanotic congenital heart disease	2021	3277276	1787	54.53 (52.03-57.11) [/100,000]	1.11 (1.04-1.19)	1.08 (1.01-1.16)
Cyanotic congenital heart disease	2022	3286661	1797	54.68 (52.18-57.26) [/100,000]	1.12 (1.04-1.19)	1.08 (1.01-1.15)
Congenital diaphragmatic hernia	2016	3558996	364	10.23 (9.20-11.33) [/100,000]	1.01 (0.87-1.17)	0.98 (0.84-1.13)
Congenital diaphragmatic hernia	2017	3475385	345	9.93 (8.91-11.03) [/100,000]	0.98 (0.84-1.14)	1.00 (0.86-1.16)
Congenital diaphragmatic hernia	2018	3414338	343	10.05 (9.01-11.17) [/100,000]	0.99 (0.85-1.15)	1.00 (0.86-1.16)
Congenital diaphragmatic hernia	2019	3367677	342	10.16 (9.11-11.29) [/100,000]	1 (Reference)	1 (Reference)
Congenital diaphragmatic hernia	2020	3248797	340	10.47 (9.38-11.64) [/100,000]	1.03 (0.89-1.20)	1.02 (0.88-1.18)
Congenital diaphragmatic hernia	2021	3277276	332	10.13 (9.07-11.28) [/100,000]	1.00 (0.86-1.16)	0.97 (0.83-1.13)
Congenital diaphragmatic hernia	2022	3286661	360	10.95 (9.85-12.15) [/100,000]	1.08 (0.93-1.25)	1.04 (0.90-1.20)
Omphalocele	2016	3558996	248	6.97 (6.13-7.89) [/100,000]	1.09 (0.91-1.30)	1.09 (0.91-1.31)
Omphalocele	2017	3475385	223	6.42 (5.60-7.32) [/100,000]	1.00 (0.83-1.21)	1.02 (0.84-1.22)
Omphalocele	2018	3414338	228	6.68 (5.84-7.60) [/100,000]	1.04 (0.86-1.25)	1.05 (0.87-1.26)
Omphalocele	2019	3367677	216	6.41 (5.59-7.33) [/100,000]	1 (Reference)	1 (Reference)
Omphalocele	2020	3248797	199	6.13 (5.30-7.04) [/100,000]	0.96 (0.79-1.16)	0.94 (0.78-1.14)
Omphalocele	2021	3277276	211	6.44 (5.60-7.37) [/100,000]	1.00 (0.83-1.21)	0.98 (0.81-1.18)
Omphalocele	2022	3286661	200	6.09 (5.27-6.99) [/100,000]	0.95 (0.78-1.15)	0.92 (0.76-1.12)
Gastroschisis	2016	3558996	361	10.14 (9.12-11.25) [/100,000]	1.09 (0.94-1.27)	1.06 (0.91-1.24)

Gastroschisis	2017	3475385	359	10.33 (9.29-11.46) [/100,000]	1.11 (0.96-1.30)	1.09 (0.94-1.27)
Gastroschisis	2018	3414338	317	9.28 (8.29-10.36) [/100,000]	1.00 (0.86-1.17)	1.00 (0.85-1.16)
Gastroschisis	2019	3367677	312	9.26 (8.26-10.35) [/100,000]	1 (Reference)	1 (Reference)
Gastroschisis	2020	3248797	272	8.37 (7.41-9.43) [/100,000]	0.90 (0.77-1.06)	0.90 (0.77-1.06)
Gastroschisis	2021	3277276	219	6.68 (5.83-7.63) [/100,000]	0.72 (0.61-0.86)	0.73 (0.61-0.87)
Gastroschisis	2022	3286661	195	5.93 (5.13-6.83) [/100,000]	0.64 (0.54-0.77)	0.65 (0.55-0.78)
Limb reduction defect	2016	3558996	411	11.55 (10.46-12.72) [/100,000]	1.22 (1.05-1.41)	1.13 (0.98-1.31)
Limb reduction defect	2017	3475385	385	11.08 (10.00-12.24) [/100,000]	1.17 (1.01-1.35)	1.17 (1.01-1.35)
Limb reduction defect	2018	3414338	362	10.60 (9.54-11.75) [/100,000]	1.12 (0.96-1.30)	1.12 (0.96-1.30)
Limb reduction defect	2019	3367677	320	9.50 (8.49-10.60) [/100,000]	1 (Reference)	1 (Reference)
Limb reduction defect	2020	3248797	320	9.85 (8.80-10.99) [/100,000]	1.04 (0.89-1.21)	1.03 (0.89-1.21)
Limb reduction defect	2021	3277276	320	9.76 (8.72-10.89) [/100,000]	1.03 (0.88-1.20)	1.03 (0.88-1.20)
Limb reduction defect	2022	3286661	327	9.95 (8.90-11.09) [/100,000]	1.05 (0.90-1.22)	1.05 (0.90-1.22)
Cleft Lip with or without Cleft Palate	2016	3558996	1744	49.00 (46.73-51.36) [/100,000]	1.10 (1.03-1.18)	1.07 (1.00-1.14)
Cleft Lip with or without Cleft Palate	2017	3475385	1630	46.90 (44.65-49.23) [/100,000]	1.06 (0.98-1.13)	1.05 (0.98-1.13)
Cleft Lip with or without Cleft Palate	2018	3414338	1699	49.76 (47.42-52.18) [/100,000]	1.12 (1.04-1.20)	1.12 (1.04-1.20)
Cleft Lip with or without Cleft Palate	2019	3367677	1497	44.45 (42.23-46.76) [/100,000]	1 (Reference)	1 (Reference)
Cleft Lip with or without Cleft Palate	2020	3248797	1473	45.34 (43.05-47.72) [/100,000]	1.02 (0.95-1.10)	1.02 (0.95-1.10)
Cleft Lip with or without Cleft Palate	2021	3277276	1558	47.54 (45.21-49.96) [/100,000]	1.07 (1.00-1.15)	1.07 (0.99-1.15)
Cleft Lip with or without Cleft Palate	2022	3286661	1537	46.76 (44.46-49.16) [/100,000]	1.05 (0.98-1.13)	1.05 (0.98-1.13)
Cleft Palate alone	2016	3558996	772	21.69 (20.19-23.28) [/100,000]	1.02 (0.92-1.12)	0.96 (0.86-1.06)
Cleft Palate alone	2017	3475385	754	21.70 (20.17-23.30) [/100,000]	1.02 (0.92-1.13)	1.02 (0.92-1.13)
Cleft Palate alone	2018	3414338	747	21.88 (20.34-23.50) [/100,000]	1.02 (0.93-1.14)	1.02 (0.92-1.13)
Cleft Palate alone	2019	3367677	719	21.35 (19.82-22.97) [/100,000]	1 (Reference)	1 (Reference)
Cleft Palate alone	2020	3248797	707	21.76 (20.19-23.43) [/100,000]	1.02 (0.92-1.13)	1.02 (0.92-1.13)
Cleft Palate alone	2021	3277276	697	21.27 (19.72-22.91) [/100,000]	1.00 (0.90-1.11)	0.99 (0.90-1.10)
Cleft Palate alone	2022	3286661	675	20.54 (19.02-22.15) [/100,000]	0.96 (0.87-1.07)	0.96 (0.87-1.07)
Down syndrome	2016	3558996	1561	43.86 (41.71-46.09) [/100,000]	1.01 (0.94-1.08)	1.00 (0.93-1.08)
Down syndrome	2017	3475385	1461	42.04 (39.91-44.25) [/100,000]	0.96 (0.90-1.04)	1.00 (0.93-1.07)
Down syndrome	2018	3414338	1529	44.78 (42.57-47.08) [/100,000]	1.03 (0.96-1.10)	1.04 (0.97-1.12)
Down syndrome	2019	3367677	1469	43.62 (41.42-45.91) [/100,000]	1 (Reference)	1 (Reference)
Down syndrome	2020	3248797	1370	42.17 (39.97-44.46) [/100,000]	0.97 (0.90-1.04)	0.95 (0.88-1.02)
Down syndrome	2021	3277276	1320	40.28 (38.13-42.51) [/100,000]	0.92 (0.86-0.99)	0.89 (0.83-0.96)
Down syndrome	2022	3286661	1325	40.31 (38.17-42.54) [/100,000]	0.92 (0.86-1.00)	0.87 (0.81-0.94)
Suspected chromosomal disorder	2016	3558996	1114	31.30 (29.49-33.19) [/100,000]	0.93 (0.86-1.01)	0.90 (0.83-0.98)
Suspected chromosomal disorder	2017	3475385	1112	32.00 (30.14-33.93) [/100,000]	0.95 (0.88-1.03)	0.97 (0.89-1.05)
Suspected chromosomal disorder	2018	3414338	1158	33.92 (31.99-35.93) [/100,000]	1.01 (0.93-1.10)	1.02 (0.94-1.10)
Suspected chromosomal disorder	2019	3367677	1131	33.58 (31.66-35.60) [/100,000]	1 (Reference)	1 (Reference)
Suspected chromosomal disorder	2020	3248797	1134	34.91 (32.90-37.00) [/100,000]	1.04 (0.96-1.13)	1.03 (0.95-1.12)
Suspected chromosomal disorder	2021	3277276	1182	36.07 (34.04-38.18) [/100,000]	1.07 (0.99-1.17)	1.05 (0.97-1.14)
Suspected chromosomal disorder	2022	3286661	1111	33.80 (31.84-35.85) [/100,000]	1.01 (0.93-1.09)	0.98 (0.90-1.06)
Hypospadias	2016	3558996	1991	55.94 (53.51-58.45) [/100,000]	1.12 (1.05-1.20)	1.03 (0.96-1.10)
Hypospadias	2017	3475385	1903	54.76 (52.32-57.27) [/100,000]	1.10 (1.03-1.17)	1.10 (1.03-1.17)
Hypospadias	2018	3414338	1811	53.04 (50.63-55.54) [/100,000]	1.06 (0.99-1.14)	1.06 (0.99-1.13)
Hypospadias	2019	3367677	1680	49.89 (47.53-52.33) [/100,000]	1 (Reference)	1 (Reference)
Hypospadias	2020	3248797	1614	49.68 (47.29-52.16) [/100,000]	1.00 (0.93-1.07)	0.99 (0.93-1.06)

Hypospadias	2021	3277276	1572	47.97 (45.63-50.40) [/100,000]	0.96 (0.90-1.03)	0.95 (0.89-1.02)
Hypospadias	2022	3286661	1540	46.86 (44.55-49.26) [/100,000]	0.94 (0.88-1.01)	0.94 (0.88-1.01)
Transferred within 24 hours of delivery	2016	3561901	23499	6.60 (6.51-6.68) [/1000]	0.93 (0.92-0.95)	0.91 (0.89-0.93)
Transferred within 24 hours of delivery	2017	3477000	23796	6.84 (6.76-6.93) [/1000]	0.97 (0.95-0.99)	0.97 (0.95-0.98)
Transferred within 24 hours of delivery	2018	3417029	24092	7.05 (6.96-7.14) [/1000]	1.00 (0.98-1.02)	1.00 (0.98-1.01)
Transferred within 24 hours of delivery	2019	3368494	23767	7.06 (6.97-7.15) [/1000]	1 (Reference)	1 (Reference)
Transferred within 24 hours of delivery	2020	3248969	21892	6.74 (6.65-6.83) [/1000]	0.95 (0.94-0.97)	0.96 (0.94-0.98)
Transferred within 24 hours of delivery	2021	3276832	22966	7.01 (6.92-7.10) [/1000]	0.99 (0.98-1.01)	1.00 (0.98-1.02)
Transferred within 24 hours of delivery	2022	3284902	22935	6.98 (6.89-7.07) [/1000]	0.99 (0.97-1.01)	1.00 (0.99-1.02)
Death before birth-certificate registration	2016	3560351	1297	36.43 (34.47-38.47) [/100,000]	1.39 (1.28-1.51)	1.35 (1.24-1.47)
Death before birth-certificate registration	2017	3474914	1039	29.90 (28.11-31.77) [/100,000]	1.14 (1.04-1.25)	1.15 (1.05-1.26)
Death before birth-certificate registration	2018	3414251	1055	30.90 (29.06-32.82) [/100,000]	1.18 (1.08-1.29)	1.19 (1.09-1.30)
Death before birth-certificate registration	2019	3367801	883	26.22 (24.52-28.01) [/100,000]	1 (Reference)	1 (Reference)
Death before birth-certificate registration	2020	3248889	881	27.12 (25.36-28.97) [/100,000]	1.03 (0.94-1.14)	1.03 (0.94-1.13)
Death before birth-certificate registration	2021	3279316	866	26.41 (24.68-28.23) [/100,000]	1.01 (0.92-1.11)	1.00 (0.91-1.10)
Death before birth-certificate registration	2022	3289280	902	27.42 (25.66-29.27) [/100,000]	1.05 (0.95-1.15)	1.03 (0.94-1.13)

Note: Details about each variable are presented in Supplementary methods. Infants with missing obstetric estimate of gestation, or missing outcomes were excluded from the analyses of the specific outcomes. The adjusted odds ratios were adjusted for maternal age, maternal residence, maternal ethnicity/race, maternal education attainment, principal source of payment for current delivery, and marital status.

Supplemental Table S6 Reported prevalence of the study outcomes by birth year (subgroup analysis, only infants delivered at <37 weeks' gestation)

Clinical outcome	Birth year	No. at risk	No. events	Reported prevalence (95% confidence interval)	Odds ratio (95% confidence interval)	
					Crude	Adjusted
Congenital anomalies	2016	387369	3676	9.49 (9.19-9.80) [/1000]	1.06 (1.01-1.11)	1.02 (0.98-1.07)
Congenital anomalies	2017	382205	3603	9.43 (9.12-9.74) [/1000]	1.05 (1.01-1.11)	1.06 (1.01-1.11)
Congenital anomalies	2018	379068	3520	9.29 (8.98-9.60) [/1000]	1.04 (0.99-1.09)	1.04 (1.00-1.09)
Congenital anomalies	2019	382519	3421	8.94 (8.65-9.25) [/1000]	1 (Reference)	1 (Reference)
Congenital anomalies	2020	363985	3288	9.03 (8.73-9.35) [/1000]	1.01 (0.96-1.06)	1.01 (0.96-1.06)
Congenital anomalies	2021	383110	3472	9.06 (8.77-9.37) [/1000]	1.01 (0.97-1.06)	1.00 (0.95-1.05)
Congenital anomalies	2022	379852	3246	8.55 (8.26-8.84) [/1000]	0.96 (0.91-1.00)	0.94 (0.90-0.99)
Anencephaly	2016	387369	141	36.40 (30.64-42.93) [/100,000]	1.06 (0.84-1.35)	1.00 (0.78-1.26)
Anencephaly	2017	382205	184	48.14 (41.44-55.62) [/100,000]	1.41 (1.12-1.76)	1.39 (1.11-1.74)
Anencephaly	2018	379068	135	35.61 (29.86-42.15) [/100,000]	1.04 (0.82-1.32)	1.03 (0.81-1.32)
Anencephaly	2019	382519	131	34.25 (28.63-40.64) [/100,000]	1 (Reference)	1 (Reference)
Anencephaly	2020	363985	133	36.54 (30.60-43.30) [/100,000]	1.07 (0.84-1.36)	1.07 (0.84-1.36)
Anencephaly	2021	383110	121	31.58 (26.21-37.74) [/100,000]	0.92 (0.72-1.18)	0.93 (0.73-1.19)
Anencephaly	2022	379852	146	38.44 (32.46-45.20) [/100,000]	1.12 (0.89-1.42)	1.13 (0.90-1.44)
Meningomyelocele/Spina bifida	2016	387369	152	39.24 (33.25-45.99) [/100,000]	0.86 (0.69-1.07)	0.86 (0.69-1.07)
Meningomyelocele/Spina bifida	2017	382205	189	49.45 (42.65-57.02) [/100,000]	1.08 (0.88-1.33)	1.10 (0.90-1.35)
Meningomyelocele/Spina bifida	2018	379068	180	47.48 (40.80-54.95) [/100,000]	1.04 (0.84-1.28)	1.05 (0.85-1.29)
Meningomyelocele/Spina bifida	2019	382519	175	45.75 (39.22-53.05) [/100,000]	1 (Reference)	1 (Reference)
Meningomyelocele/Spina bifida	2020	363985	168	46.16 (39.44-53.69) [/100,000]	1.01 (0.82-1.25)	1.01 (0.82-1.25)
Meningomyelocele/Spina bifida	2021	383110	160	41.76 (35.54-48.76) [/100,000]	0.91 (0.74-1.13)	0.89 (0.72-1.10)
Meningomyelocele/Spina bifida	2022	379852	202	53.18 (46.10-61.04) [/100,000]	1.16 (0.95-1.42)	1.13 (0.92-1.38)
Cyanotic congenital heart disease	2016	387369	585	1.51 (1.39-1.64) [/1000]	1.01 (0.90-1.14)	0.95 (0.84-1.06)
Cyanotic congenital heart disease	2017	382205	553	1.45 (1.33-1.57) [/1000]	0.97 (0.86-1.09)	0.98 (0.87-1.10)
Cyanotic congenital heart disease	2018	379068	555	1.46 (1.34-1.59) [/1000]	0.98 (0.87-1.10)	0.99 (0.88-1.11)
Cyanotic congenital heart disease	2019	382519	571	1.49 (1.37-1.62) [/1000]	1 (Reference)	1 (Reference)
Cyanotic congenital heart disease	2020	363985	563	1.55 (1.42-1.68) [/1000]	1.04 (0.92-1.16)	1.03 (0.92-1.16)
Cyanotic congenital heart disease	2021	383110	703	1.83 (1.70-1.98) [/1000]	1.23 (1.10-1.37)	1.20 (1.08-1.35)
Cyanotic congenital heart disease	2022	379852	628	1.65 (1.53-1.79) [/1000]	1.11 (0.99-1.24)	1.08 (0.97-1.21)
Congenital diaphragmatic hernia	2016	387369	136	35.11 (29.46-41.53) [/100,000]	1.03 (0.81-1.31)	1.00 (0.79-1.28)
Congenital diaphragmatic hernia	2017	382205	126	32.97 (27.46-39.25) [/100,000]	0.97 (0.76-1.24)	0.98 (0.77-1.26)
Congenital diaphragmatic hernia	2018	379068	129	34.03 (28.41-40.43) [/100,000]	1.00 (0.78-1.28)	1.01 (0.79-1.29)
Congenital diaphragmatic hernia	2019	382519	130	33.99 (28.40-40.35) [/100,000]	1 (Reference)	1 (Reference)
Congenital diaphragmatic hernia	2020	363985	129	35.44 (29.59-42.11) [/100,000]	1.04 (0.82-1.33)	1.04 (0.82-1.33)
Congenital diaphragmatic hernia	2021	383110	130	33.93 (28.35-40.29) [/100,000]	1.00 (0.78-1.27)	0.98 (0.77-1.25)
Congenital diaphragmatic hernia	2022	379852	126	33.17 (27.63-39.49) [/100,000]	0.98 (0.76-1.25)	0.96 (0.75-1.22)
Omphalocele	2016	387369	156	40.27 (34.20-47.11) [/100,000]	0.87 (0.70-1.08)	0.86 (0.69-1.07)
Omphalocele	2017	382205	177	46.31 (39.74-53.66) [/100,000]	1.00 (0.81-1.23)	1.01 (0.82-1.24)
Omphalocele	2018	379068	180	47.48 (40.80-54.95) [/100,000]	1.03 (0.83-1.26)	1.03 (0.84-1.27)
Omphalocele	2019	382519	177	46.27 (39.71-53.61) [/100,000]	1 (Reference)	1 (Reference)
Omphalocele	2020	363985	136	37.36 (31.35-44.20) [/100,000]	0.81 (0.65-1.01)	0.81 (0.64-1.01)
Omphalocele	2021	383110	164	42.81 (36.51-49.88) [/100,000]	0.93 (0.75-1.14)	0.91 (0.74-1.13)
Omphalocele	2022	379852	150	39.49 (33.42-46.34) [/100,000]	0.85 (0.69-1.06)	0.84 (0.68-1.05)
Gastroschisis	2016	387369	598	154.37 (142.26-167.25) [/100,000]	1.14 (1.02-1.29)	1.10 (0.98-1.24)

Gastroschisis	2017	382205	566	148.09 (136.15-160.80) [/100,000]	1.10 (0.97-1.24)	1.07 (0.95-1.20)
Gastroschisis	2018	379068	513	135.33 (123.88-147.56) [/100,000]	1.00 (0.89-1.13)	0.99 (0.88-1.12)
Gastroschisis	2019	382519	516	134.90 (123.51-147.04) [/100,000]	1 (Reference)	1 (Reference)
Gastroschisis	2020	363985	474	130.23 (118.77-142.48) [/100,000]	0.97 (0.85-1.09)	0.97 (0.86-1.10)
Gastroschisis	2021	383110	470	122.68 (111.84-134.28) [/100,000]	0.91 (0.80-1.03)	0.93 (0.82-1.05)
Gastroschisis	2022	379852	391	102.93 (92.99-113.65) [/100,000]	0.76 (0.67-0.87)	0.78 (0.69-0.90)
Limb reduction defect	2016	387369	138	35.62 (29.93-42.09) [/100,000]	1.13 (0.88-1.44)	1.05 (0.82-1.34)
Limb reduction defect	2017	382205	141	36.89 (31.05-43.51) [/100,000]	1.17 (0.91-1.49)	1.16 (0.91-1.48)
Limb reduction defect	2018	379068	125	32.98 (27.45-39.29) [/100,000]	1.04 (0.81-1.34)	1.04 (0.81-1.34)
Limb reduction defect	2019	382519	121	31.63 (26.25-37.80) [/100,000]	1 (Reference)	1 (Reference)
Limb reduction defect	2020	363985	120	32.97 (27.33-39.42) [/100,000]	1.04 (0.81-1.34)	1.04 (0.81-1.34)
Limb reduction defect	2021	383110	105	27.41 (22.42-33.18) [/100,000]	0.87 (0.67-1.13)	0.87 (0.67-1.13)
Limb reduction defect	2022	379852	97	25.54 (20.71-31.15) [/100,000]	0.81 (0.62-1.05)	0.81 (0.62-1.06)
Cleft Lip with or without Cleft Palate	2016	387369	365	94.23 (84.81-104.40) [/100,000]	1.06 (0.91-1.23)	1.02 (0.88-1.18)
Cleft Lip with or without Cleft Palate	2017	382205	363	94.98 (85.46-105.26) [/100,000]	1.07 (0.92-1.24)	1.07 (0.92-1.24)
Cleft Lip with or without Cleft Palate	2018	379068	353	93.12 (83.66-103.36) [/100,000]	1.04 (0.90-1.21)	1.05 (0.90-1.21)
Cleft Lip with or without Cleft Palate	2019	382519	341	89.15 (79.94-99.12) [/100,000]	1 (Reference)	1 (Reference)
Cleft Lip with or without Cleft Palate	2020	363985	316	86.82 (77.51-96.93) [/100,000]	0.97 (0.84-1.13)	0.97 (0.83-1.13)
Cleft Lip with or without Cleft Palate	2021	383110	355	92.66 (83.28-102.82) [/100,000]	1.04 (0.90-1.21)	1.03 (0.89-1.20)
Cleft Lip with or without Cleft Palate	2022	379852	322	84.77 (75.77-94.55) [/100,000]	0.95 (0.82-1.11)	0.94 (0.81-1.10)
Cleft Palate alone	2016	387369	143	36.92 (31.11-43.48) [/100,000]	0.97 (0.77-1.22)	0.92 (0.73-1.16)
Cleft Palate alone	2017	382205	174	45.53 (39.01-52.81) [/100,000]	1.19 (0.96-1.49)	1.20 (0.96-1.49)
Cleft Palate alone	2018	379068	173	45.64 (39.09-52.97) [/100,000]	1.20 (0.96-1.49)	1.20 (0.96-1.49)
Cleft Palate alone	2019	382519	146	38.17 (32.23-44.88) [/100,000]	1 (Reference)	1 (Reference)
Cleft Palate alone	2020	363985	137	37.64 (31.60-44.49) [/100,000]	0.99 (0.78-1.25)	0.98 (0.78-1.24)
Cleft Palate alone	2021	383110	149	38.89 (32.90-45.66) [/100,000]	1.02 (0.81-1.28)	1.01 (0.80-1.27)
Cleft Palate alone	2022	379852	143	37.65 (31.73-44.35) [/100,000]	0.99 (0.78-1.24)	0.98 (0.78-1.23)
Down syndrome	2016	387369	607	1.57 (1.44-1.70) [/1000]	1.08 (0.96-1.21)	1.07 (0.95-1.20)
Down syndrome	2017	382205	580	1.52 (1.40-1.65) [/1000]	1.04 (0.93-1.17)	1.08 (0.96-1.21)
Down syndrome	2018	379068	579	1.53 (1.41-1.66) [/1000]	1.05 (0.94-1.18)	1.07 (0.95-1.20)
Down syndrome	2019	382519	556	1.45 (1.34-1.58) [/1000]	1 (Reference)	1 (Reference)
Down syndrome	2020	363985	583	1.60 (1.47-1.74) [/1000]	1.10 (0.98-1.24)	1.09 (0.97-1.22)
Down syndrome	2021	383110	581	1.52 (1.40-1.64) [/1000]	1.04 (0.93-1.17)	1.01 (0.90-1.13)
Down syndrome	2022	379852	534	1.41 (1.29-1.53) [/1000]	0.97 (0.86-1.09)	0.92 (0.82-1.04)
Suspected chromosomal disorder	2016	387369	584	1.51 (1.39-1.63) [/1000]	1.08 (0.96-1.21)	1.04 (0.92-1.17)
Suspected chromosomal disorder	2017	382205	545	1.43 (1.31-1.55) [/1000]	1.02 (0.90-1.15)	1.03 (0.92-1.16)
Suspected chromosomal disorder	2018	379068	567	1.50 (1.38-1.62) [/1000]	1.07 (0.95-1.20)	1.07 (0.95-1.21)
Suspected chromosomal disorder	2019	382519	536	1.40 (1.29-1.52) [/1000]	1 (Reference)	1 (Reference)
Suspected chromosomal disorder	2020	363985	517	1.42 (1.30-1.55) [/1000]	1.01 (0.90-1.14)	1.01 (0.89-1.14)
Suspected chromosomal disorder	2021	383110	548	1.43 (1.31-1.56) [/1000]	1.02 (0.91-1.15)	1.00 (0.89-1.13)
Suspected chromosomal disorder	2022	379852	482	1.27 (1.16-1.39) [/1000]	0.91 (0.80-1.02)	0.88 (0.78-1.00)
Hypospadias	2016	387369	464	119.78 (109.14-131.19) [/100,000]	1.18 (1.03-1.35)	1.08 (0.95-1.24)
Hypospadias	2017	382205	411	107.53 (97.39-118.44) [/100,000]	1.06 (0.92-1.21)	1.06 (0.92-1.22)
Hypospadias	2018	379068	425	112.12 (101.72-123.29) [/100,000]	1.10 (0.96-1.27)	1.11 (0.96-1.27)
Hypospadias	2019	382519	389	101.69 (91.84-112.31) [/100,000]	1 (Reference)	1 (Reference)
Hypospadias	2020	363985	392	107.70 (97.30-118.90) [/100,000]	1.06 (0.92-1.22)	1.06 (0.92-1.22)

Hypospadias	2021	383110	383	99.97 (90.21-110.50) [/100,000]	0.98 (0.85-1.13)	0.97 (0.84-1.11)
Hypospadias	2022	379852	369	97.14 (87.49-107.57) [/100,000]	0.96 (0.83-1.10)	0.94 (0.82-1.09)
Transferred within 24 hours of delivery	2016	388635	20944	5.39 (5.32-5.46) [/100]	1.02 (1.00-1.04)	1.00 (0.98-1.02)
Transferred within 24 hours of delivery	2017	383105	20508	5.35 (5.28-5.42) [/100]	1.01 (0.99-1.03)	1.00 (0.98-1.02)
Transferred within 24 hours of delivery	2018	380214	20255	5.33 (5.26-5.40) [/100]	1.01 (0.99-1.03)	1.00 (0.98-1.02)
Transferred within 24 hours of delivery	2019	383429	20269	5.29 (5.22-5.36) [/100]	1 (Reference)	1 (Reference)
Transferred within 24 hours of delivery	2020	364495	18570	5.09 (5.02-5.17) [/100]	0.96 (0.94-0.98)	0.96 (0.94-0.98)
Transferred within 24 hours of delivery	2021	383897	19719	5.14 (5.07-5.21) [/100]	0.97 (0.95-0.99)	0.98 (0.96-1.00)
Transferred within 24 hours of delivery	2022	380450	19124	5.03 (4.96-5.10) [/100]	0.95 (0.93-0.97)	0.97 (0.95-0.99)
Death before birth-certificate registration	2016	386318	9359	2.42 (2.37-2.47) [/100]	1.17 (1.13-1.20)	1.15 (1.11-1.18)
Death before birth-certificate registration	2017	380820	8971	2.36 (2.31-2.40) [/100]	1.14 (1.10-1.17)	1.14 (1.11-1.18)
Death before birth-certificate registration	2018	377803	8469	2.24 (2.19-2.29) [/100]	1.08 (1.05-1.11)	1.09 (1.05-1.12)
Death before birth-certificate registration	2019	381303	7931	2.08 (2.03-2.13) [/100]	1 (Reference)	1 (Reference)
Death before birth-certificate registration	2020	362661	7281	2.01 (1.96-2.05) [/100]	0.96 (0.93-1.00)	0.96 (0.93-1.00)
Death before birth-certificate registration	2021	382387	6894	1.80 (1.76-1.85) [/100]	0.86 (0.84-0.89)	0.87 (0.84-0.89)
Death before birth-certificate registration	2022	379069	7093	1.87 (1.83-1.91) [/100]	0.90 (0.87-0.93)	0.90 (0.87-0.93)

Note: Details about each variable are presented in Supplementary methods. Infants with missing obstetric estimate of gestation, or missing outcomes were excluded from the analyses of the specific outcomes. The adjusted odds ratios were adjusted for maternal age, maternal residence, maternal ethnicity/race, maternal education attainment, principal source of payment for current delivery, and marital status.

Supplemental Table S7 Multiplicity-corrected results for the pre-specified family of contrasts

Family	Anomaly	Birth year	Adjusted OR (95% CI)	Exact p	Adjusted OR (Bonferroni-adjusted CI)	Bonferroni p	Benjamini-Hochberg q
Primary	Anencephaly	2020	1.03 (0.89-1.20)	0.658	1.03 (0.81-1.33)	1.000	0.878
Primary	Anencephaly	2021	0.96 (0.82-1.12)	0.571	0.96 (0.74-1.23)	1.000	0.857
Primary	Anencephaly	2022	1.19 (1.02-1.37)	0.022	1.19 (0.93-1.51)	0.804	0.115
Primary	Meningomyelocele/Spina bifida	2020	0.93 (0.83-1.06)	0.275	0.93 (0.77-1.14)	1.000	0.521
Primary	Meningomyelocele/Spina bifida	2021	0.92 (0.81-1.04)	0.183	0.92 (0.75-1.12)	1.000	0.388
Primary	Meningomyelocele/Spina bifida	2022	0.92 (0.81-1.03)	0.155	0.92 (0.75-1.12)	1.000	0.388
Primary	Cyanotic congenital heart disease	2020	1.06 (1.00-1.12)	0.062	1.06 (0.96-1.16)	1.000	0.201
Primary	Cyanotic congenital heart disease	2021	1.12 (1.05-1.18)	<0.001	1.12 (1.02-1.23)	0.005	0.002
Primary	Cyanotic congenital heart disease	2022	1.08 (1.02-1.14)	0.008	1.08 (0.98-1.19)	0.304	0.061
Primary	Congenital diaphragmatic hernia	2020	1.02 (0.90-1.16)	0.755	1.02 (0.83-1.26)	1.000	0.894
Primary	Congenital diaphragmatic hernia	2021	0.97 (0.86-1.11)	0.687	0.97 (0.79-1.20)	1.000	0.883
Primary	Congenital diaphragmatic hernia	2022	1.02 (0.89-1.15)	0.809	1.02 (0.83-1.25)	1.000	0.894
Primary	Omphalocele	2020	0.87 (0.75-1.01)	0.060	0.87 (0.69-1.10)	1.000	0.201
Primary	Omphalocele	2021	0.95 (0.82-1.09)	0.468	0.95 (0.75-1.19)	1.000	0.803
Primary	Omphalocele	2022	0.88 (0.76-1.02)	0.080	0.88 (0.70-1.11)	1.000	0.239
Primary	Gastroschisis	2020	0.93 (0.85-1.03)	0.183	0.93 (0.80-1.10)	1.000	0.388
Primary	Gastroschisis	2021	0.86 (0.78-0.96)	0.004	0.86 (0.73-1.02)	0.157	0.039
Primary	Gastroschisis	2022	0.74 (0.66-0.82)	<0.001	0.74 (0.62-0.88)	<0.001	<0.001
Primary	Limb reduction defect	2020	1.03 (0.91-1.18)	0.628	1.03 (0.83-1.28)	1.000	0.869
Primary	Limb reduction defect	2021	0.99 (0.87-1.13)	0.906	0.99 (0.80-1.23)	1.000	0.932
Primary	Limb reduction defect	2022	0.98 (0.86-1.13)	0.819	0.98 (0.79-1.22)	1.000	0.894
Primary	Cleft Lip with or without Cleft Palate	2020	1.01 (0.95-1.08)	0.783	1.01 (0.91-1.12)	1.000	0.894
Primary	Cleft Lip with or without Cleft Palate	2021	1.06 (1.00-1.13)	0.056	1.06 (0.96-1.18)	1.000	0.201
Primary	Cleft Lip with or without Cleft Palate	2022	1.03 (0.97-1.10)	0.347	1.03 (0.93-1.15)	1.000	0.625
Primary	Cleft Palate alone	2020	1.01 (0.92-1.11)	0.793	1.01 (0.87-1.18)	1.000	0.894
Primary	Cleft Palate alone	2021	1.00 (0.91-1.10)	0.984	1.00 (0.86-1.17)	1.000	0.984
Primary	Cleft Palate alone	2022	0.97 (0.88-1.07)	0.537	0.97 (0.83-1.13)	1.000	0.857
Primary	Down syndrome	2020	0.98 (0.92-1.04)	0.561	0.98 (0.89-1.09)	1.000	0.857
Primary	Down syndrome	2021	0.93 (0.87-0.99)	0.018	0.93 (0.84-1.03)	0.651	0.108
Primary	Down syndrome	2022	0.89 (0.83-0.94)	<0.001	0.89 (0.80-0.98)	0.005	0.002
Primary	Suspected chromosomal disorder	2020	1.02 (0.95-1.09)	0.607	1.02 (0.91-1.14)	1.000	0.869
Primary	Suspected chromosomal disorder	2021	1.04 (0.97-1.11)	0.238	1.04 (0.93-1.16)	1.000	0.477
Primary	Suspected chromosomal disorder	2022	0.95 (0.88-1.02)	0.126	0.95 (0.85-1.06)	1.000	0.350
Primary	Hypospadias	2020	1.00 (0.94-1.07)	0.905	1.00 (0.91-1.11)	1.000	0.932
Primary	Hypospadias	2021	0.96 (0.90-1.02)	0.180	0.96 (0.87-1.06)	1.000	0.388
Primary	Hypospadias	2022	0.94 (0.89-1.00)	0.060	0.94 (0.85-1.04)	1.000	0.201
Extended	Anencephaly	2016	0.98 (0.84-1.13)	0.742	0.98 (0.75-1.26)	1.000	0.891
Extended	Anencephaly	2017	1.26 (1.09-1.45)	0.002	1.26 (0.98-1.61)	0.116	0.023
Extended	Anencephaly	2018	1.05 (0.90-1.22)	0.516	1.05 (0.81-1.36)	1.000	0.844
Extended	Anencephaly	2020	1.03 (0.89-1.20)	0.658	1.03 (0.80-1.35)	1.000	0.891
Extended	Anencephaly	2021	0.96 (0.82-1.12)	0.571	0.96 (0.73-1.25)	1.000	0.875
Extended	Anencephaly	2022	1.19 (1.02-1.37)	0.022	1.19 (0.92-1.53)	1.000	0.134
Extended	Meningomyelocele/Spina bifida	2016	0.96 (0.85-1.08)	0.480	0.96 (0.78-1.18)	1.000	0.804
Extended	Meningomyelocele/Spina bifida	2017	1.09 (0.97-1.23)	0.145	1.09 (0.89-1.33)	1.000	0.418

Extended	Meningomyelocele/Spina bifida	2018	1.02 (0.91-1.15)	0.698	1.02 (0.83-1.26)	1.000	0.891
Extended	Meningomyelocele/Spina bifida	2020	0.93 (0.83-1.06)	0.275	0.93 (0.76-1.15)	1.000	0.535
Extended	Meningomyelocele/Spina bifida	2021	0.92 (0.81-1.04)	0.183	0.92 (0.75-1.14)	1.000	0.436
Extended	Meningomyelocele/Spina bifida	2022	0.92 (0.81-1.03)	0.155	0.92 (0.74-1.13)	1.000	0.429
Extended	Cyanotic congenital heart disease	2016	0.89 (0.84-0.95)	<0.001	0.89 (0.80-0.99)	0.009	0.003
Extended	Cyanotic congenital heart disease	2017	0.95 (0.89-1.01)	0.089	0.95 (0.86-1.05)	1.000	0.319
Extended	Cyanotic congenital heart disease	2018	0.99 (0.94-1.05)	0.839	0.99 (0.90-1.10)	1.000	0.916
Extended	Cyanotic congenital heart disease	2020	1.06 (1.00-1.12)	0.062	1.06 (0.96-1.17)	1.000	0.252
Extended	Cyanotic congenital heart disease	2021	1.12 (1.05-1.18)	<0.001	1.12 (1.01-1.23)	0.011	0.003
Extended	Cyanotic congenital heart disease	2022	1.08 (1.02-1.14)	0.008	1.08 (0.98-1.19)	0.608	0.068
Extended	Congenital diaphragmatic hernia	2016	0.98 (0.86-1.11)	0.755	0.98 (0.79-1.22)	1.000	0.891
Extended	Congenital diaphragmatic hernia	2017	0.99 (0.87-1.12)	0.853	0.99 (0.79-1.23)	1.000	0.917
Extended	Congenital diaphragmatic hernia	2018	1.00 (0.88-1.13)	0.958	1.00 (0.80-1.24)	1.000	0.972
Extended	Congenital diaphragmatic hernia	2020	1.02 (0.90-1.16)	0.755	1.02 (0.82-1.27)	1.000	0.891
Extended	Congenital diaphragmatic hernia	2021	0.97 (0.86-1.11)	0.687	0.97 (0.78-1.22)	1.000	0.891
Extended	Congenital diaphragmatic hernia	2022	1.02 (0.89-1.15)	0.809	1.02 (0.82-1.26)	1.000	0.908
Extended	Omphalocele	2016	0.97 (0.84-1.11)	0.622	0.97 (0.76-1.23)	1.000	0.891
Extended	Omphalocele	2017	0.99 (0.87-1.14)	0.934	0.99 (0.78-1.26)	1.000	0.961
Extended	Omphalocele	2018	1.02 (0.89-1.17)	0.752	1.02 (0.81-1.30)	1.000	0.891
Extended	Omphalocele	2020	0.87 (0.75-1.01)	0.060	0.87 (0.68-1.12)	1.000	0.252
Extended	Omphalocele	2021	0.95 (0.82-1.09)	0.468	0.95 (0.74-1.21)	1.000	0.803
Extended	Omphalocele	2022	0.88 (0.76-1.02)	0.080	0.88 (0.69-1.13)	1.000	0.302
Extended	Gastroschisis	2016	1.06 (0.97-1.17)	0.200	1.06 (0.90-1.25)	1.000	0.436
Extended	Gastroschisis	2017	1.07 (0.97-1.17)	0.188	1.07 (0.91-1.25)	1.000	0.436
Extended	Gastroschisis	2018	0.98 (0.89-1.08)	0.746	0.98 (0.83-1.16)	1.000	0.891
Extended	Gastroschisis	2020	0.93 (0.85-1.03)	0.183	0.93 (0.79-1.11)	1.000	0.436
Extended	Gastroschisis	2021	0.86 (0.78-0.96)	0.004	0.86 (0.72-1.03)	0.314	0.039
Extended	Gastroschisis	2022	0.74 (0.66-0.82)	<0.001	0.74 (0.61-0.89)	<0.001	<0.001
Extended	Limb reduction defect	2016	1.10 (0.97-1.25)	0.124	1.10 (0.89-1.37)	1.000	0.379
Extended	Limb reduction defect	2017	1.16 (1.02-1.32)	0.021	1.16 (0.93-1.44)	1.000	0.134
Extended	Limb reduction defect	2018	1.09 (0.96-1.24)	0.180	1.09 (0.87-1.36)	1.000	0.436
Extended	Limb reduction defect	2020	1.03 (0.91-1.18)	0.628	1.03 (0.82-1.30)	1.000	0.891
Extended	Limb reduction defect	2021	0.99 (0.87-1.13)	0.906	0.99 (0.79-1.25)	1.000	0.946
Extended	Limb reduction defect	2022	0.98 (0.86-1.13)	0.819	0.98 (0.78-1.24)	1.000	0.908
Extended	Cleft Lip with or without Cleft Palate	2016	1.05 (0.99-1.12)	0.096	1.05 (0.95-1.18)	1.000	0.330
Extended	Cleft Lip with or without Cleft Palate	2017	1.05 (0.99-1.12)	0.115	1.05 (0.94-1.17)	1.000	0.376
Extended	Cleft Lip with or without Cleft Palate	2018	1.10 (1.04-1.17)	0.002	1.10 (0.99-1.23)	0.164	0.027
Extended	Cleft Lip with or without Cleft Palate	2020	1.01 (0.95-1.08)	0.783	1.01 (0.90-1.13)	1.000	0.907
Extended	Cleft Lip with or without Cleft Palate	2021	1.06 (1.00-1.13)	0.056	1.06 (0.95-1.19)	1.000	0.252
Extended	Cleft Lip with or without Cleft Palate	2022	1.03 (0.97-1.10)	0.347	1.03 (0.92-1.15)	1.000	0.640
Extended	Cleft Palate alone	2016	0.95 (0.86-1.04)	0.253	0.95 (0.81-1.11)	1.000	0.505
Extended	Cleft Palate alone	2017	1.04 (0.95-1.15)	0.361	1.04 (0.89-1.23)	1.000	0.640
Extended	Cleft Palate alone	2018	1.05 (0.96-1.15)	0.292	1.05 (0.90-1.23)	1.000	0.552
Extended	Cleft Palate alone	2020	1.01 (0.92-1.11)	0.793	1.01 (0.86-1.19)	1.000	0.907
Extended	Cleft Palate alone	2021	1.00 (0.91-1.10)	0.984	1.00 (0.85-1.18)	1.000	0.984
Extended	Cleft Palate alone	2022	0.97 (0.88-1.07)	0.537	0.97 (0.82-1.14)	1.000	0.859

Extended	Down syndrome	2016	1.01 (0.95-1.08)	0.704	1.01 (0.91-1.12)	1.000	0.891
Extended	Down syndrome	2017	1.01 (0.95-1.08)	0.672	1.01 (0.91-1.13)	1.000	0.891
Extended	Down syndrome	2018	1.04 (0.98-1.11)	0.197	1.04 (0.94-1.16)	1.000	0.436
Extended	Down syndrome	2020	0.98 (0.92-1.04)	0.561	0.98 (0.88-1.09)	1.000	0.875
Extended	Down syndrome	2021	0.93 (0.87-0.99)	0.018	0.93 (0.83-1.03)	1.000	0.130
Extended	Down syndrome	2022	0.89 (0.83-0.94)	<0.001	0.89 (0.79-0.99)	0.011	0.003
Extended	Suspected chromosomal disorder	2016	0.94 (0.88-1.00)	0.063	0.94 (0.83-1.05)	1.000	0.252
Extended	Suspected chromosomal disorder	2017	0.99 (0.92-1.05)	0.670	0.99 (0.88-1.11)	1.000	0.891
Extended	Suspected chromosomal disorder	2018	1.03 (0.96-1.10)	0.365	1.03 (0.92-1.16)	1.000	0.640
Extended	Suspected chromosomal disorder	2020	1.02 (0.95-1.09)	0.607	1.02 (0.90-1.15)	1.000	0.891
Extended	Suspected chromosomal disorder	2021	1.04 (0.97-1.11)	0.238	1.04 (0.93-1.17)	1.000	0.490
Extended	Suspected chromosomal disorder	2022	0.95 (0.88-1.02)	0.126	0.95 (0.84-1.07)	1.000	0.379
Extended	Hypospadias	2016	1.04 (0.98-1.10)	0.236	1.04 (0.94-1.15)	1.000	0.490
Extended	Hypospadias	2017	1.09 (1.03-1.16)	0.004	1.09 (0.99-1.21)	0.269	0.038
Extended	Hypospadias	2018	1.07 (1.01-1.13)	0.030	1.07 (0.96-1.19)	1.000	0.164
Extended	Hypospadias	2020	1.00 (0.94-1.07)	0.905	1.00 (0.90-1.12)	1.000	0.946
Extended	Hypospadias	2021	0.96 (0.90-1.02)	0.180	0.96 (0.86-1.07)	1.000	0.436
Extended	Hypospadias	2022	0.94 (0.89-1.00)	0.060	0.94 (0.85-1.05)	1.000	0.252

Note: The Primary family comprises the 12 specific congenital anomalies by the three pandemic-year contrasts (2020, 2021 and 2022 versus birth year 2019), m=36. The Extended family additionally includes the three pre-pandemic contrasts (2016, 2017 and 2018 versus 2019), m=72, and is reported as a sensitivity analysis. Correction is applied within each family separately, so a contrast appears once in each block with different adjusted values. The composite outcome, transfer within 24 hours of delivery and death before birth-certificate registration were pre-specified as separate outcomes and are reported outside both families; the gestational-age–stratified analyses are sensitivity analyses and were not counted towards either family. Odds ratios are from the adjusted logistic models described in the Statistical analysis section (adjusted for maternal age, residence, ethnicity/race, education attainment, principal source of payment for the current delivery, and marital status). Exact p-values were recomputed from the fitted models rather than back-calculated from rounded confidence intervals. Bonferroni-adjusted confidence intervals are 1-0.05/m intervals; Bonferroni-adjusted p-values are the exact p-values multiplied by m and truncated at 1.000; q-values are from the Benjamini-Hochberg procedure applied within the family. CI, confidence interval; OR, odds ratio.

Supplemental Table S8 Reported prevalence of cyanotic congenital heart disease by birth year with additional adjustment

Additionally adjusted covariate	Birth year	Odds ratio (95% confidence interval)		
		Crude	Model 1	Model 2
First prenatal care visit	2016	0.94 (0.89-1.00)	0.89 (0.84-0.95)	0.89 (0.84-0.94)
	2017	0.93 (0.88-0.99)	0.95 (0.89-1.01)	0.95 (0.89-1.00)
	2018	0.99 (0.93-1.05)	0.99 (0.94-1.05)	0.99 (0.93-1.05)
	2019	1 (Reference)	1 (Reference)	1 (Reference)
	2020	1.07 (1.01-1.13)	1.06 (1.00-1.12)	1.06 (1.00-1.13)
	2021	1.15 (1.08-1.22)	1.12 (1.05-1.18)	1.12 (1.06-1.19)
	2022	1.12 (1.05-1.18)	1.08 (1.02-1.14)	1.08 (1.02-1.14)
WIC food during pregnancy	2016	0.94 (0.89-1.00)	0.89 (0.84-0.95)	0.89 (0.84-0.95)
	2017	0.93 (0.88-0.99)	0.95 (0.89-1.01)	0.95 (0.90-1.01)
	2018	0.99 (0.93-1.05)	0.99 (0.94-1.05)	0.99 (0.94-1.06)
	2019	1 (Reference)	1 (Reference)	1 (Reference)
	2020	1.07 (1.01-1.13)	1.06 (1.00-1.12)	1.06 (1.00-1.12)
	2021	1.15 (1.08-1.22)	1.12 (1.05-1.18)	1.12 (1.05-1.18)
	2022	1.12 (1.05-1.18)	1.08 (1.02-1.14)	1.08 (1.02-1.14)
Cigarette smoking during pregnancy	2016	0.94 (0.89-1.00)	0.89 (0.84-0.95)	0.89 (0.84-0.95)
	2017	0.93 (0.88-0.99)	0.95 (0.89-1.01)	0.95 (0.90-1.01)
	2018	0.99 (0.93-1.05)	0.99 (0.94-1.05)	0.99 (0.94-1.05)
	2019	1 (Reference)	1 (Reference)	1 (Reference)
	2020	1.07 (1.01-1.13)	1.06 (1.00-1.12)	1.06 (1.00-1.12)
	2021	1.15 (1.08-1.22)	1.12 (1.05-1.18)	1.12 (1.06-1.18)
	2022	1.12 (1.05-1.18)	1.08 (1.02-1.14)	1.08 (1.02-1.15)
Maternal BMI	2016	0.94 (0.89-1.00)	0.89 (0.84-0.95)	0.89 (0.84-0.95)
	2017	0.93 (0.88-0.99)	0.95 (0.89-1.01)	0.95 (0.89-1.01)
	2018	0.99 (0.93-1.05)	0.99 (0.94-1.05)	0.99 (0.94-1.05)
	2019	1 (Reference)	1 (Reference)	1 (Reference)
	2020	1.07 (1.01-1.13)	1.06 (1.00-1.12)	1.06 (1.00-1.12)
	2021	1.15 (1.08-1.22)	1.12 (1.05-1.18)	1.12 (1.06-1.18)
	2022	1.12 (1.05-1.18)	1.08 (1.02-1.14)	1.08 (1.02-1.15)
Prepregnancy diabetes	2016	0.94 (0.89-1.00)	0.89 (0.84-0.95)	0.89 (0.84-0.95)
	2017	0.93 (0.88-0.99)	0.95 (0.89-1.01)	0.95 (0.90-1.01)
	2018	0.99 (0.93-1.05)	0.99 (0.94-1.05)	0.99 (0.94-1.05)
	2019	1 (Reference)	1 (Reference)	1 (Reference)
	2020	1.07 (1.01-1.13)	1.06 (1.00-1.12)	1.05 (0.99-1.12)
	2021	1.15 (1.08-1.22)	1.12 (1.05-1.18)	1.11 (1.05-1.18)
	2022	1.12 (1.05-1.18)	1.08 (1.02-1.14)	1.07 (1.01-1.14)
Gestational diabetes	2016	0.94 (0.89-1.00)	0.89 (0.84-0.95)	0.89 (0.84-0.95)
	2017	0.93 (0.88-0.99)	0.95 (0.89-1.01)	0.95 (0.90-1.01)
	2018	0.99 (0.93-1.05)	0.99 (0.94-1.05)	0.99 (0.94-1.05)
	2019	1 (Reference)	1 (Reference)	1 (Reference)
	2020	1.07 (1.01-1.13)	1.06 (1.00-1.12)	1.05 (0.99-1.12)
	2021	1.15 (1.08-1.22)	1.12 (1.05-1.18)	1.11 (1.05-1.18)
	2022	1.12 (1.05-1.18)	1.08 (1.02-1.14)	1.08 (1.02-1.14)
Prepregnancy hypertension	2016	0.94 (0.89-1.00)	0.89 (0.84-0.95)	0.89 (0.84-0.95)
	2017	0.93 (0.88-0.99)	0.95 (0.89-1.01)	0.95 (0.90-1.01)
	2018	0.99 (0.93-1.05)	0.99 (0.94-1.05)	1.00 (0.94-1.06)
	2019	1 (Reference)	1 (Reference)	1 (Reference)
	2020	1.07 (1.01-1.13)	1.06 (1.00-1.12)	1.05 (0.99-1.12)
	2021	1.15 (1.08-1.22)	1.12 (1.05-1.18)	1.11 (1.05-1.18)
	2022	1.12 (1.05-1.18)	1.08 (1.02-1.14)	1.07 (1.01-1.14)
Gestational hypertension	2016	0.94 (0.89-1.00)	0.89 (0.84-0.95)	0.90 (0.85-0.95)
	2017	0.93 (0.88-0.99)	0.95 (0.89-1.01)	0.96 (0.90-1.01)
	2018	0.99 (0.93-1.05)	0.99 (0.94-1.05)	1.00 (0.94-1.06)
	2019	1 (Reference)	1 (Reference)	1 (Reference)
	2020	1.07 (1.01-1.13)	1.06 (1.00-1.12)	1.05 (0.99-1.12)
	2021	1.15 (1.08-1.22)	1.12 (1.05-1.18)	1.11 (1.05-1.18)
	2022	1.12 (1.05-1.18)	1.08 (1.02-1.14)	1.07 (1.01-1.14)
Pregnancy resulted from infertility treatment	2016	0.94 (0.89-1.00)	0.89 (0.84-0.95)	0.89 (0.84-0.95)
	2017	0.93 (0.88-0.99)	0.95 (0.89-1.01)	0.95 (0.90-1.01)
	2018	0.99 (0.93-1.05)	0.99 (0.94-1.05)	0.99 (0.94-1.05)
	2019	1 (Reference)	1 (Reference)	1 (Reference)
	2020	1.07 (1.01-1.13)	1.06 (1.00-1.12)	1.06 (1.00-1.12)
	2021	1.15 (1.08-1.22)	1.12 (1.05-1.18)	1.12 (1.05-1.18)
	2022	1.12 (1.05-1.18)	1.08 (1.02-1.14)	1.08 (1.02-1.14)
Fertility enhancing drugs/artificial insemination/intrauterine insemination	2016	0.94 (0.89-1.00)	0.89 (0.84-0.95)	0.89 (0.84-0.95)
	2017	0.93 (0.88-0.99)	0.95 (0.89-1.01)	0.95 (0.90-1.01)
	2018	0.99 (0.93-1.05)	0.99 (0.94-1.05)	0.99 (0.94-1.05)
	2019	1 (Reference)	1 (Reference)	1 (Reference)

	2020	1.07 (1.01-1.13)	1.06 (1.00-1.12)	1.06 (1.00-1.12)
	2021	1.15 (1.08-1.22)	1.12 (1.05-1.18)	1.12 (1.05-1.18)
	2022	1.12 (1.05-1.18)	1.08 (1.02-1.14)	1.08 (1.02-1.14)
Assisted reproductive technology	2016	0.94 (0.89-1.00)	0.89 (0.84-0.95)	0.89 (0.84-0.95)
	2017	0.93 (0.88-0.99)	0.95 (0.89-1.01)	0.95 (0.90-1.01)
	2018	0.99 (0.93-1.05)	0.99 (0.94-1.05)	0.99 (0.94-1.05)
	2019	1 (Reference)	1 (Reference)	1 (Reference)
	2020	1.07 (1.01-1.13)	1.06 (1.00-1.12)	1.06 (1.00-1.12)
	2021	1.15 (1.08-1.22)	1.12 (1.05-1.18)	1.12 (1.05-1.18)
	2022	1.12 (1.05-1.18)	1.08 (1.02-1.14)	1.08 (1.02-1.14)
Infections present and/or treated during this pregnancy	2016	0.94 (0.89-1.00)	0.89 (0.84-0.95)	0.89 (0.84-0.95)
	2017	0.93 (0.88-0.99)	0.95 (0.89-1.01)	0.95 (0.90-1.01)
	2018	0.99 (0.93-1.05)	0.99 (0.94-1.05)	0.99 (0.94-1.05)
	2019	1 (Reference)	1 (Reference)	1 (Reference)
	2020	1.07 (1.01-1.13)	1.06 (1.00-1.12)	1.06 (1.00-1.12)
	2021	1.15 (1.08-1.22)	1.12 (1.05-1.18)	1.12 (1.06-1.18)
	2022	1.12 (1.05-1.18)	1.08 (1.02-1.14)	1.08 (1.02-1.15)
Plurality	2016	0.94 (0.89-1.00)	0.89 (0.84-0.95)	0.89 (0.84-0.94)
	2017	0.93 (0.88-0.99)	0.95 (0.89-1.01)	0.95 (0.89-1.01)
	2018	0.99 (0.93-1.05)	0.99 (0.94-1.05)	0.99 (0.94-1.05)
	2019	1 (Reference)	1 (Reference)	1 (Reference)
	2020	1.07 (1.01-1.13)	1.06 (1.00-1.12)	1.06 (1.00-1.12)
	2021	1.15 (1.08-1.22)	1.12 (1.05-1.18)	1.12 (1.06-1.18)
	2022	1.12 (1.05-1.18)	1.08 (1.02-1.14)	1.08 (1.02-1.15)
Sex of the infant	2016	0.94 (0.89-1.00)	0.89 (0.84-0.95)	0.89 (0.84-0.95)
	2017	0.93 (0.88-0.99)	0.95 (0.89-1.01)	0.95 (0.89-1.01)
	2018	0.99 (0.93-1.05)	0.99 (0.94-1.05)	0.99 (0.94-1.05)
	2019	1 (Reference)	1 (Reference)	1 (Reference)
	2020	1.07 (1.01-1.13)	1.06 (1.00-1.12)	1.06 (1.00-1.12)
	2021	1.15 (1.08-1.22)	1.12 (1.05-1.18)	1.12 (1.05-1.18)
	2022	1.12 (1.05-1.18)	1.08 (1.02-1.14)	1.08 (1.02-1.14)

Note: Details about each variable are presented in Supplementary methods. Infants with missing outcomes were excluded from the analyses of the specific outcomes. Model 1 was adjusted for maternal age, maternal residence, maternal ethnicity/race, maternal education attainment, principal source of payment for current delivery, and marital status. Model 2 was additionally adjusted for the specific covariate on the basis of Model 1.

Supplemental Table S9 Fully adjusted models for cyanotic congenital heart disease, and collinearity diagnostics

Model	Birth year	Odds ratio versus 2019 (95% CI)	p
Base model	2016	0.89 (0.84-0.95)	<0.001
Base model	2017	0.95 (0.89-1.01)	0.089
Base model	2018	0.99 (0.94-1.05)	0.839
Base model	2020	1.06 (1.00-1.12)	0.062
Base model	2021	1.12 (1.05-1.18)	<0.001
Base model	2022	1.08 (1.02-1.14)	0.008
Fully adjusted 1	2016	0.91 (0.86-0.96)	0.001
Fully adjusted 1	2017	0.96 (0.90-1.01)	0.134
Fully adjusted 1	2018	0.99 (0.94-1.05)	0.846
Fully adjusted 1	2020	1.05 (0.99-1.12)	0.078
Fully adjusted 1	2021	1.11 (1.04-1.17)	<0.001
Fully adjusted 1	2022	1.06 (1.00-1.12)	0.060
Fully adjusted 2	2016	0.91 (0.85-0.96)	0.001
Fully adjusted 2	2017	0.95 (0.90-1.01)	0.128
Fully adjusted 2	2018	0.99 (0.94-1.05)	0.838
Fully adjusted 2	2020	1.05 (0.99-1.12)	0.074
Fully adjusted 2	2021	1.11 (1.05-1.17)	<0.001
Fully adjusted 2	2022	1.06 (1.00-1.12)	0.055

Model	Covariate	df	Generalized VIF	Adjusted generalized VIF
Fully adjusted 1	Birth year	6	1.03	1.00
Fully adjusted 1	Maternal age	1	1.34	1.16
Fully adjusted 1	Maternal residence	3	1.08	1.01
Fully adjusted 1	Maternal ethnicity/race	7	1.82	1.04
Fully adjusted 1	Maternal education attainment	8	2.61	1.06
Fully adjusted 1	Principal source of payment for current delivery	4	2.00	1.09
Fully adjusted 1	Marital status	2	1.55	1.12
Fully adjusted 1	First prenatal care visit	4	1.22	1.03
Fully adjusted 1	WIC food during pregnancy	2	1.65	1.13
Fully adjusted 1	Cigarette smoking during pregnancy	2	1.26	1.06
Fully adjusted 1	Maternal BMI	6	1.33	1.02
Fully adjusted 1	Prepregnancy diabetes	1	1.10	1.05
Fully adjusted 1	Gestational diabetes	1	1.07	1.03
Fully adjusted 1	Prepregnancy hypertension	1	1.12	1.06
Fully adjusted 1	Gestational hypertension	1	1.05	1.03
Fully adjusted 1	Infertility treatment	4	1.07	1.01
Fully adjusted 1	Risk factor with Unknown level	1	1.13	1.06
Fully adjusted 1	Infections present and/or treated during current pregnancy	2	1.18	1.04
Fully adjusted 2	Birth year	6	1.03	1.00
Fully adjusted 2	Maternal age	1	1.34	1.16
Fully adjusted 2	Maternal residence	3	1.08	1.01
Fully adjusted 2	Maternal ethnicity/race	7	1.82	1.04
Fully adjusted 2	Maternal education attainment	8	2.61	1.06
Fully adjusted 2	Principal source of payment for current delivery	4	2.00	1.09
Fully adjusted 2	Marital status	2	1.55	1.12
Fully adjusted 2	First prenatal care visit	4	1.22	1.03
Fully adjusted 2	WIC food during pregnancy	2	1.65	1.13
Fully adjusted 2	Cigarette smoking during pregnancy	2	1.26	1.06
Fully adjusted 2	Maternal BMI	6	1.33	1.02
Fully adjusted 2	Prepregnancy diabetes	1	1.10	1.05
Fully adjusted 2	Gestational diabetes	1	1.07	1.03
Fully adjusted 2	Prepregnancy hypertension	1	1.12	1.06
Fully adjusted 2	Gestational hypertension	1	1.06	1.03
Fully adjusted 2	Infertility treatment	4	1.11	1.01
Fully adjusted 2	Risk factor with Unknown level	1	1.13	1.06
Fully adjusted 2	Infections present and/or treated during current pregnancy	2	1.18	1.04
Fully adjusted 2	Plurality	3	1.05	1.01
Fully adjusted 2	Sex of the infant	1	1.00	1.00

Note: n = 26,302,211 live births with a non-missing cyanotic congenital heart disease item, of whom 15,971 had the item checked. The base model is adjusted for maternal age, residence, ethnicity/race, education attainment, principal source of payment for the current delivery, and marital status. Fully adjusted model 1 adds all remaining maternal and prenatal covariates simultaneously; fully adjusted model 2 additionally includes plurality and sex of the infant. Two groups of categories on the birth certificate are structurally redundant and made the model rank-deficient as originally coded: the “unknown or not stated” categories of the pre-pregnancy diabetes, gestational diabetes, pre-pregnancy hypertension, gestational hypertension and infertility-treatment items are recorded jointly and mark the same records, and the “not applicable” categories of the fertility-enhancing-drug and assisted-reproductive-technology items are determined by construction by the infertility-treatment item. Under the original coding seven indicator columns were therefore aliased and dropped by the fitting routine, and variance inflation factors were undefined. The models reported here reparameterise those items into a single shared “unknown” indicator (shown as “Risk factor with Unknown level”) and a single fertility variable built from the observed combinations of the three fertility items (shown as “Infertility treatment”, 4 degrees of freedom). The reparameterisation spans the same column space that the fitting routine

estimates: the birth-year odds ratios, the residual degrees of freedom and the deviance are identical to those of the original coding, and no coefficient is aliased. Generalised variance inflation factors follow Fox and Monette; the adjusted value, GVIF^(1/2df), is comparable across terms with different degrees of freedom. Every adjusted value is below 1.20, and birth year is essentially orthogonal to the covariate set (adjusted GVIF 1.00), so collinearity does not threaten the year contrasts. CI, confidence interval; GVIF, generalised variance inflation factor; df, degrees of freedom.

Supplemental Table S10 Singleton-restricted and confirmation-status sensitivity analyses

Analysis	Outcome	Birth year	No. at risk	No. events	Reported prevalence per 100,000 (95% CI)	Adjusted OR (95% CI)	p
Singletons only	Congenital anomalies	2016	3813576	13183	345.7 (339.8-351.6)	0.99 (0.96-1.01)	0.363
Singletons only	Congenital anomalies	2017	3727574	12876	345.4 (339.5-351.4)	1.04 (1.01-1.07)	0.002
Singletons only	Congenital anomalies	2018	3668346	12826	349.6 (343.6-355.7)	1.05 (1.02-1.07)	<0.001
Singletons only	Congenital anomalies	2019	3628428	12191	336.0 (330.1-342.0)	1 (Reference)	-
Singletons only	Congenital anomalies	2020	3499314	11934	341.0 (335.0-347.2)	1.01 (0.98-1.03)	0.596
Singletons only	Congenital anomalies	2021	3545355	12085	340.9 (334.8-347.0)	1.00 (0.97-1.02)	0.826
Singletons only	Congenital anomalies	2022	3551200	11801	332.3 (326.4-338.4)	0.97 (0.94-0.99)	0.013
Singletons only	Cyanotic congenital heart disease	2016	3813576	2083	54.6 (52.3-57.0)	0.89 (0.84-0.94)	<0.001
Singletons only	Cyanotic congenital heart disease	2017	3727574	2019	54.2 (51.8-56.6)	0.95 (0.89-1.01)	0.089
Singletons only	Cyanotic congenital heart disease	2018	3668346	2127	58.0 (55.5-60.5)	1.00 (0.95-1.07)	0.889
Singletons only	Cyanotic congenital heart disease	2019	3628428	2111	58.2 (55.7-60.7)	1 (Reference)	-
Singletons only	Cyanotic congenital heart disease	2020	3499314	2169	62.0 (59.4-64.6)	1.05 (0.99-1.12)	0.093
Singletons only	Cyanotic congenital heart disease	2021	3545355	2341	66.0 (63.4-68.8)	1.10 (1.04-1.17)	0.001
Singletons only	Cyanotic congenital heart disease	2022	3551200	2300	64.8 (62.1-67.5)	1.08 (1.01-1.14)	0.015
Confirmed records only	Down syndrome	2016	3948055	939	23.8 (22.3-25.4)	0.98 (0.90-1.08)	0.715
Confirmed records only	Down syndrome	2017	3858674	901	23.3 (21.9-24.9)	0.99 (0.90-1.08)	0.789
Confirmed records only	Down syndrome	2018	3794240	921	24.3 (22.7-25.9)	1.00 (0.92-1.10)	0.952
Confirmed records only	Down syndrome	2019	3751007	923	24.6 (23.0-26.2)	1 (Reference)	-
Confirmed records only	Down syndrome	2020	3613601	894	24.7 (23.1-26.4)	0.99 (0.90-1.08)	0.753
Confirmed records only	Down syndrome	2021	3661334	857	23.4 (21.9-25.0)	0.91 (0.83-1.00)	0.055
Confirmed records only	Down syndrome	2022	3667485	818	22.3 (20.8-23.9)	0.85 (0.77-0.93)	<0.001
Confirmed records only	Suspected chromosomal disorder	2016	3948167	583	14.8 (13.6-16.0)	0.97 (0.86-1.09)	0.595
Confirmed records only	Suspected chromosomal disorder	2017	3858696	537	13.9 (12.8-15.1)	0.94 (0.84-1.06)	0.343
Confirmed records only	Suspected chromosomal disorder	2018	3794283	582	15.3 (14.1-16.6)	1.02 (0.91-1.15)	0.678
Confirmed records only	Suspected chromosomal disorder	2019	3751015	568	15.1 (13.9-16.4)	1 (Reference)	-
Confirmed records only	Suspected chromosomal disorder	2020	3613535	526	14.6 (13.3-15.9)	0.95 (0.84-1.07)	0.370
Confirmed records only	Suspected chromosomal disorder	2021	3661230	581	15.9 (14.6-17.2)	1.01 (0.90-1.14)	0.819
Confirmed records only	Suspected chromosomal disorder	2022	3667485	552	15.1 (13.8-16.4)	0.95 (0.84-1.07)	0.367
Pending records only	Down syndrome	2016	3948345	1229	31.1 (29.4-32.9)	1.04 (0.95-1.12)	0.402
Pending records only	Down syndrome	2017	3858916	1143	29.6 (27.9-31.4)	1.03 (0.95-1.12)	0.415
Pending records only	Down syndrome	2018	3794506	1187	31.3 (29.5-33.1)	1.07 (0.99-1.16)	0.093
Pending records only	Down syndrome	2019	3751191	1107	29.5 (27.8-31.3)	1 (Reference)	-
Pending records only	Down syndrome	2020	3613767	1060	29.3 (27.6-31.2)	0.98 (0.90-1.06)	0.612
Pending records only	Down syndrome	2021	3661523	1046	28.6 (26.9-30.4)	0.94 (0.86-1.02)	0.145
Pending records only	Down syndrome	2022	3667710	1043	28.4 (26.7-30.2)	0.92 (0.84-1.00)	0.042
Pending records only	Suspected chromosomal disorder	2016	3948701	1117	28.3 (26.7-30.0)	0.92 (0.85-1.00)	0.058
Pending records only	Suspected chromosomal disorder	2017	3859280	1121	29.0 (27.4-30.8)	1.01 (0.93-1.09)	0.885
Pending records only	Suspected chromosomal disorder	2018	3794845	1144	30.1 (28.4-31.9)	1.04 (0.95-1.12)	0.412
Pending records only	Suspected chromosomal disorder	2019	3751546	1099	29.3 (27.6-31.1)	1 (Reference)	-
Pending records only	Suspected chromosomal disorder	2020	3614135	1126	31.2 (29.4-33.0)	1.05 (0.97-1.15)	0.208
Pending records only	Suspected chromosomal disorder	2021	3661799	1150	31.4 (29.6-33.3)	1.06 (0.97-1.15)	0.198
Pending records only	Suspected chromosomal disorder	2022	3667976	1043	28.4 (26.7-30.2)	0.95 (0.87-1.03)	0.215

Note: Reported prevalence is the birth-certificate–reported prevalence at live birth, per 100,000 live births, with 95% Clopper-Pearson confidence intervals; odds ratios are adjusted as in the primary analysis (maternal age, residence, ethnicity/race, education attainment, principal source of payment for the current delivery, and marital status), with birth year 2019 as the reference. The singleton-restricted analysis addresses the non-

independence of observations within multiple births, which could not be modelled because the data do not identify infants belonging to the same pregnancy; among singletons the observations are independent by construction. In the confirmation-status analyses, records marked “pending” were excluded from the confirmed-only comparison and records marked “confirmed” were excluded from the pending-only comparison, so that the two comparisons use mutually exclusive sets of cases against the same non-case denominator; this addresses the concern that karyotype confirmation often occurs after the certificate is registered. CI, confidence interval; OR, odds ratio.

Supplemental Table S11 Sensitivity analysis re-indexed by estimated year of conception

Outcome	Estimated conception year	No. at risk	No. events	Reported prevalence per 100,000 (95% CI)	Adjusted OR versus 2019 (95% CI)	p
Congenital anomalies	2016	3870775	13416	346.6 (340.8-352.5)	1.01 (0.99-1.04)	0.378
Congenital anomalies	2017	3811945	13269	348.1 (342.2-354.1)	1.03 (1.00-1.05)	0.045
Congenital anomalies	2018	3761079	12874	342.3 (336.4-348.2)	1.00 (0.98-1.03)	0.783
Congenital anomalies	2019	3674014	12617	343.4 (337.5-349.4)	1 (Reference)	-
Congenital anomalies	2020	3598619	12454	346.1 (340.0-352.2)	1.00 (0.97-1.02)	0.898
Congenital anomalies	2021	3671433	12447	339.0 (333.1-345.0)	0.97 (0.95-1.00)	0.031
Anencephaly	2016	3870775	378	9.8 (8.8-10.8)	1.03 (0.89-1.19)	0.678
Anencephaly	2017	3811945	388	10.2 (9.2-11.2)	1.10 (0.95-1.27)	0.207
Anencephaly	2018	3761079	333	8.9 (7.9-9.9)	0.96 (0.82-1.11)	0.560
Anencephaly	2019	3674014	340	9.3 (8.3-10.3)	1 (Reference)	-
Anencephaly	2020	3598619	302	8.4 (7.5-9.4)	0.91 (0.78-1.06)	0.227
Anencephaly	2021	3671433	371	10.1 (9.1-11.2)	1.09 (0.94-1.26)	0.247
Meningomyelocele/Spina bifida	2016	3870775	576	14.9 (13.7-16.1)	1.13 (1.00-1.27)	0.051
Meningomyelocele/Spina bifida	2017	3811945	558	14.6 (13.4-15.9)	1.11 (0.98-1.25)	0.098
Meningomyelocele/Spina bifida	2018	3761079	552	14.7 (13.5-16.0)	1.10 (0.98-1.25)	0.110
Meningomyelocele/Spina bifida	2019	3674014	493	13.4 (12.3-14.7)	1 (Reference)	-
Meningomyelocele/Spina bifida	2020	3598619	516	14.3 (13.1-15.6)	1.05 (0.93-1.19)	0.434
Meningomyelocele/Spina bifida	2021	3671433	497	13.5 (12.4-14.8)	0.98 (0.87-1.11)	0.775
Cyanotic congenital heart disease	2016	3870775	2104	54.4 (52.1-56.7)	0.85 (0.81-0.91)	<0.001
Cyanotic congenital heart disease	2017	3811945	2184	57.3 (54.9-59.7)	0.91 (0.86-0.97)	0.002
Cyanotic congenital heart disease	2018	3761079	2198	58.4 (56.0-60.9)	0.92 (0.87-0.98)	0.008
Cyanotic congenital heart disease	2019	3674014	2347	63.9 (61.3-66.5)	1 (Reference)	-
Cyanotic congenital heart disease	2020	3598619	2396	66.6 (63.9-69.3)	1.03 (0.97-1.09)	0.383
Cyanotic congenital heart disease	2021	3671433	2460	67.0 (64.4-69.7)	1.02 (0.97-1.08)	0.412
Congenital diaphragmatic hernia	2016	3870775	476	12.3 (11.2-13.5)	0.96 (0.85-1.09)	0.529
Congenital diaphragmatic hernia	2017	3811945	459	12.0 (11.0-13.2)	0.95 (0.83-1.07)	0.387
Congenital diaphragmatic hernia	2018	3761079	473	12.6 (11.5-13.8)	0.98 (0.86-1.11)	0.748
Congenital diaphragmatic hernia	2019	3674014	477	13.0 (11.8-14.2)	1 (Reference)	-
Congenital diaphragmatic hernia	2020	3598619	451	12.5 (11.4-13.7)	0.95 (0.83-1.08)	0.419
Congenital diaphragmatic hernia	2021	3671433	484	13.2 (12.0-14.4)	0.99 (0.87-1.12)	0.840
Omphalocele	2016	3870775	383	9.9 (8.9-10.9)	1.02 (0.88-1.18)	0.787
Omphalocele	2017	3811945	406	10.7 (9.6-11.7)	1.09 (0.95-1.26)	0.216
Omphalocele	2018	3761079	398	10.6 (9.6-11.7)	1.08 (0.94-1.25)	0.284
Omphalocele	2019	3674014	363	9.9 (8.9-11.0)	1 (Reference)	-
Omphalocele	2020	3598619	358	9.9 (8.9-11.0)	0.99 (0.86-1.15)	0.940
Omphalocele	2021	3671433	354	9.6 (8.7-10.7)	0.96 (0.83-1.11)	0.578
Gastroschisis	2016	3870775	948	24.5 (23.0-26.1)	1.13 (1.03-1.24)	0.011
Gastroschisis	2017	3811945	862	22.6 (21.1-24.2)	1.06 (0.96-1.17)	0.229
Gastroschisis	2018	3761079	821	21.8 (20.4-23.4)	1.03 (0.94-1.14)	0.524
Gastroschisis	2019	3674014	774	21.1 (19.6-22.6)	1 (Reference)	-
Gastroschisis	2020	3598619	721	20.0 (18.6-21.6)	0.95 (0.86-1.06)	0.358
Gastroschisis	2021	3671433	620	16.9 (15.6-18.3)	0.82 (0.74-0.91)	<0.001
Limb reduction defect	2016	3870775	522	13.5 (12.4-14.7)	1.08 (0.95-1.23)	0.215
Limb reduction defect	2017	3811945	503	13.2 (12.1-14.4)	1.08 (0.95-1.23)	0.227

Limb reduction defect	2018	3761079	449	11.9 (10.9-13.1)	0.98 (0.86-1.12)	0.743
Limb reduction defect	2019	3674014	449	12.2 (11.1-13.4)	1 (Reference)	-
Limb reduction defect	2020	3598619	425	11.8 (10.7-13.0)	0.97 (0.85-1.10)	0.606
Limb reduction defect	2021	3671433	424	11.5 (10.5-12.7)	0.95 (0.83-1.08)	0.423
Cleft Lip with or without Cleft Palate	2016	3870775	2016	52.1 (49.8-54.4)	1.06 (0.99-1.13)	0.087
Cleft Lip with or without Cleft Palate	2017	3811945	2012	52.8 (50.5-55.1)	1.08 (1.02-1.15)	0.016
Cleft Lip with or without Cleft Palate	2018	3761079	1915	50.9 (48.7-53.2)	1.04 (0.98-1.11)	0.188
Cleft Lip with or without Cleft Palate	2019	3674014	1792	48.8 (46.5-51.1)	1 (Reference)	-
Cleft Lip with or without Cleft Palate	2020	3598619	1865	51.8 (49.5-54.2)	1.06 (0.99-1.13)	0.078
Cleft Lip with or without Cleft Palate	2021	3671433	1853	50.5 (48.2-52.8)	1.03 (0.97-1.10)	0.334
Cleft Palate alone	2016	3870775	911	23.5 (22.0-25.1)	0.97 (0.88-1.06)	0.527
Cleft Palate alone	2017	3811945	940	24.7 (23.1-26.3)	1.03 (0.94-1.13)	0.479
Cleft Palate alone	2018	3761079	870	23.1 (21.6-24.7)	0.97 (0.88-1.07)	0.523
Cleft Palate alone	2019	3674014	877	23.9 (22.3-25.5)	1 (Reference)	-
Cleft Palate alone	2020	3598619	815	22.6 (21.1-24.3)	0.95 (0.86-1.04)	0.249
Cleft Palate alone	2021	3671433	826	22.5 (21.0-24.1)	0.94 (0.86-1.04)	0.228
Down syndrome	2016	3870775	2117	54.7 (52.4-57.1)	1.03 (0.97-1.10)	0.306
Down syndrome	2017	3811945	2077	54.5 (52.2-56.9)	1.02 (0.96-1.09)	0.445
Down syndrome	2018	3761079	2016	53.6 (51.3-56.0)	0.99 (0.93-1.06)	0.806
Down syndrome	2019	3674014	2020	55.0 (52.6-57.4)	1 (Reference)	-
Down syndrome	2020	3598619	1906	53.0 (50.6-55.4)	0.95 (0.89-1.01)	0.118
Down syndrome	2021	3671433	1916	52.2 (49.9-54.6)	0.91 (0.86-0.97)	0.004
Suspected chromosomal disorder	2016	3870775	1635	42.2 (40.2-44.3)	0.95 (0.89-1.02)	0.162
Suspected chromosomal disorder	2017	3811945	1708	44.8 (42.7-47.0)	1.02 (0.95-1.09)	0.613
Suspected chromosomal disorder	2018	3761079	1705	45.3 (43.2-47.5)	1.02 (0.95-1.09)	0.539
Suspected chromosomal disorder	2019	3674014	1647	44.8 (42.7-47.0)	1 (Reference)	-
Suspected chromosomal disorder	2020	3598619	1712	47.6 (45.3-49.9)	1.05 (0.98-1.13)	0.145
Suspected chromosomal disorder	2021	3671433	1627	44.3 (42.2-46.5)	0.97 (0.90-1.04)	0.342
Hypospadias	2016	3870775	2367	61.2 (58.7-63.7)	1.07 (1.01-1.14)	0.024
Hypospadias	2017	3811945	2243	58.8 (56.4-61.3)	1.05 (0.99-1.12)	0.090
Hypospadias	2018	3761079	2126	56.5 (54.1-59.0)	1.01 (0.95-1.07)	0.720
Hypospadias	2019	3674014	2057	56.0 (53.6-58.5)	1 (Reference)	-
Hypospadias	2020	3598619	1938	53.9 (51.5-56.3)	0.95 (0.90-1.02)	0.142
Hypospadias	2021	3671433	1939	52.8 (50.5-55.2)	0.94 (0.89-1.00)	0.061
Transferred within 24 hours of delivery	2016	3873742	43990	1135.6 (1125.1-1146.2)	0.98 (0.97-1.00)	0.016
Transferred within 24 hours of delivery	2017	3815773	44429	1164.4 (1153.6-1175.2)	1.02 (1.00-1.03)	0.021
Transferred within 24 hours of delivery	2018	3763293	44155	1173.3 (1162.5-1184.2)	1.03 (1.01-1.04)	<0.001
Transferred within 24 hours of delivery	2019	3675111	41948	1141.4 (1130.6-1152.3)	1 (Reference)	-
Transferred within 24 hours of delivery	2020	3598854	41364	1149.4 (1138.4-1160.4)	1.01 (1.00-1.03)	0.094
Transferred within 24 hours of delivery	2021	3669664	42402	1155.5 (1144.6-1166.5)	1.02 (1.01-1.04)	<0.001
Death before birth-certificate registration	2016	3869524	10284	265.8 (260.7-270.9)	1.14 (1.10-1.17)	<0.001
Death before birth-certificate registration	2017	3810785	9829	257.9 (252.9-263.1)	1.11 (1.08-1.15)	<0.001
Death before birth-certificate registration	2018	3759939	9215	245.1 (240.1-250.1)	1.05 (1.02-1.08)	<0.001
Death before birth-certificate registration	2019	3672688	8626	234.9 (229.9-239.9)	1 (Reference)	-
Death before birth-certificate registration	2020	3598839	7844	218.0 (213.2-222.8)	0.93 (0.90-0.96)	<0.001
Death before birth-certificate registration	2021	3673190	7757	211.2 (206.5-215.9)	0.89 (0.86-0.92)	<0.001

Note: Estimated conception was derived from the reported birth month and the obstetric estimate of gestation, taking conception as the last menstrual period plus two weeks and placing birth at the middle of the reported birth month, because the exact day of birth is not released by NCHS. Because the accessed files cover births in 2016-2022, conception cohorts are complete only for conception years 2016-2021, to which the analysis was restricted; 22,387,865 infants (85.1% of live births with a non-missing composite outcome) were assignable, the remaining 14.9% being conceived in 2015 or in 2022 and therefore belonging to an incomplete cohort. Conception year 2019 is the reference, its first trimester falling entirely before the pandemic; conception years 2020 and 2021 are the cohorts whose first trimester fell within the pandemic. Reported prevalence is presented per 100,000 live births for every outcome, including the composite outcome, for consistency across rows, with 95% Clopper-Pearson confidence intervals. Odds ratios are adjusted as in the primary analysis. The estimate of conception inherits the imprecision of the obstetric estimate of gestation and of the mid-month approximation, which will misassign infants born close to a year boundary and biases the comparison towards the null. CI, confidence interval; OR, odds ratio.

Supplemental Table S12 Reported prevalence of the study outcomes by birth month

Clinical outcome	Birth year	Birth month	No. at risk	No. events	Reported prevalence (95% confidence interval)
Congenital anomalies	2016	1	317114	1117	3.52 (3.32-3.73) [/1000]
Congenital anomalies	2016	2	306225	1143	3.73 (3.52-3.95) [/1000]
Congenital anomalies	2016	3	328615	1210	3.68 (3.48-3.90) [/1000]
Congenital anomalies	2016	4	313767	1117	3.56 (3.35-3.77) [/1000]
Congenital anomalies	2016	5	327877	1176	3.59 (3.38-3.80) [/1000]
Congenital anomalies	2016	6	332521	1141	3.43 (3.24-3.64) [/1000]
Congenital anomalies	2016	7	343039	1197	3.49 (3.29-3.69) [/1000]
Congenital anomalies	2016	8	358174	1257	3.51 (3.32-3.71) [/1000]
Congenital anomalies	2016	9	346017	1159	3.35 (3.16-3.55) [/1000]
Congenital anomalies	2016	10	331330	1098	3.31 (3.12-3.52) [/1000]
Congenital anomalies	2016	11	319684	1083	3.39 (3.19-3.60) [/1000]
Congenital anomalies	2016	12	324921	1093	3.36 (3.17-3.57) [/1000]
Congenital anomalies	2017	1	314080	1078	3.43 (3.23-3.64) [/1000]
Congenital anomalies	2017	2	289301	1022	3.53 (3.32-3.76) [/1000]
Congenital anomalies	2017	3	319946	1112	3.48 (3.27-3.69) [/1000]
Congenital anomalies	2017	4	300459	1030	3.43 (3.22-3.64) [/1000]
Congenital anomalies	2017	5	322813	1142	3.54 (3.34-3.75) [/1000]
Congenital anomalies	2017	6	324241	1161	3.58 (3.38-3.79) [/1000]
Congenital anomalies	2017	7	335296	1188	3.54 (3.34-3.75) [/1000]
Congenital anomalies	2017	8	352660	1231	3.49 (3.30-3.69) [/1000]
Congenital anomalies	2017	9	337673	1162	3.44 (3.25-3.64) [/1000]
Congenital anomalies	2017	10	330310	1124	3.40 (3.21-3.61) [/1000]
Congenital anomalies	2017	11	316776	1057	3.34 (3.14-3.54) [/1000]
Congenital anomalies	2017	12	316262	1098	3.47 (3.27-3.68) [/1000]
Congenital anomalies	2018	1	315049	1136	3.61 (3.40-3.82) [/1000]
Congenital anomalies	2018	2	284508	1039	3.65 (3.43-3.88) [/1000]
Congenital anomalies	2018	3	316374	1120	3.54 (3.34-3.75) [/1000]
Congenital anomalies	2018	4	298702	1091	3.65 (3.44-3.88) [/1000]
Congenital anomalies	2018	5	320843	1113	3.47 (3.27-3.68) [/1000]
Congenital anomalies	2018	6	314959	1097	3.48 (3.28-3.69) [/1000]
Congenital anomalies	2018	7	329189	1109	3.37 (3.17-3.57) [/1000]
Congenital anomalies	2018	8	345135	1169	3.39 (3.20-3.59) [/1000]
Congenital anomalies	2018	9	323243	1096	3.39 (3.19-3.60) [/1000]
Congenital anomalies	2018	10	327299	1242	3.79 (3.59-4.01) [/1000]
Congenital anomalies	2018	11	309004	1071	3.47 (3.26-3.68) [/1000]
Congenital anomalies	2018	12	311122	1031	3.31 (3.11-3.52) [/1000]
Congenital anomalies	2019	1	311189	1059	3.40 (3.20-3.61) [/1000]
Congenital anomalies	2019	2	280254	1013	3.61 (3.40-3.84) [/1000]
Congenital anomalies	2019	3	304527	1058	3.47 (3.27-3.69) [/1000]
Congenital anomalies	2019	4	299272	972	3.25 (3.05-3.46) [/1000]
Congenital anomalies	2019	5	316562	1034	3.27 (3.07-3.47) [/1000]
Congenital anomalies	2019	6	304319	1081	3.55 (3.34-3.77) [/1000]
Congenital anomalies	2019	7	334075	1085	3.25 (3.06-3.45) [/1000]
Congenital anomalies	2019	8	342126	1149	3.36 (3.17-3.56) [/1000]
Congenital anomalies	2019	9	326234	1134	3.48 (3.28-3.68) [/1000]
Congenital anomalies	2019	10	325771	1054	3.24 (3.04-3.44) [/1000]
Congenital anomalies	2019	11	298501	1007	3.37 (3.17-3.59) [/1000]
Congenital anomalies	2019	12	309284	1044	3.38 (3.17-3.59) [/1000]
Congenital anomalies	2020	1	305211	1085	3.55 (3.35-3.77) [/1000]
Congenital anomalies	2020	2	283058	963	3.40 (3.19-3.62) [/1000]
Congenital anomalies	2020	3	302012	1009	3.34 (3.14-3.55) [/1000]
Congenital anomalies	2020	4	290578	972	3.35 (3.14-3.56) [/1000]
Congenital anomalies	2020	5	301454	1013	3.36 (3.16-3.57) [/1000]
Congenital anomalies	2020	6	302144	1110	3.67 (3.46-3.90) [/1000]
Congenital anomalies	2020	7	321591	1092	3.40 (3.20-3.60) [/1000]
Congenital anomalies	2020	8	319610	1087	3.40 (3.20-3.61) [/1000]
Congenital anomalies	2020	9	311656	1066	3.42 (3.22-3.63) [/1000]
Congenital anomalies	2020	10	305015	1026	3.36 (3.16-3.58) [/1000]
Congenital anomalies	2020	11	282530	977	3.46 (3.24-3.68) [/1000]
Congenital anomalies	2020	12	289802	990	3.42 (3.21-3.64) [/1000]
Congenital anomalies	2021	1	277054	991	3.58 (3.36-3.81) [/1000]
Congenital anomalies	2021	2	266235	982	3.69 (3.46-3.93) [/1000]
Congenital anomalies	2021	3	302457	1105	3.65 (3.44-3.87) [/1000]
Congenital anomalies	2021	4	293104	1073	3.66 (3.45-3.89) [/1000]
Congenital anomalies	2021	5	300804	1031	3.43 (3.22-3.64) [/1000]
Congenital anomalies	2021	6	313498	1040	3.32 (3.12-3.52) [/1000]
Congenital anomalies	2021	7	325951	1110	3.41 (3.21-3.61) [/1000]
Congenital anomalies	2021	8	329954	1138	3.45 (3.25-3.65) [/1000]

Congenital anomalies	2021	9	325613	1062	3.26 (3.07-3.46) [/1000]
Congenital anomalies	2021	10	314971	1080	3.43 (3.23-3.64) [/1000]
Congenital anomalies	2021	11	301665	987	3.27 (3.07-3.48) [/1000]
Congenital anomalies	2021	12	311074	1037	3.33 (3.13-3.54) [/1000]
Congenital anomalies	2022	1	294903	985	3.34 (3.14-3.55) [/1000]
Congenital anomalies	2022	2	275665	980	3.56 (3.34-3.78) [/1000]
Congenital anomalies	2022	3	307473	1060	3.45 (3.24-3.66) [/1000]
Congenital anomalies	2022	4	288688	947	3.28 (3.08-3.50) [/1000]
Congenital anomalies	2022	5	297684	1013	3.40 (3.20-3.62) [/1000]
Congenital anomalies	2022	6	305797	1036	3.39 (3.19-3.60) [/1000]
Congenital anomalies	2022	7	320845	1129	3.52 (3.32-3.73) [/1000]
Congenital anomalies	2022	8	336052	1142	3.40 (3.20-3.60) [/1000]
Congenital anomalies	2022	9	312405	1034	3.31 (3.11-3.52) [/1000]
Congenital anomalies	2022	10	306926	1000	3.26 (3.06-3.47) [/1000]
Congenital anomalies	2022	11	307231	957	3.11 (2.92-3.32) [/1000]
Congenital anomalies	2022	12	314859	1000	3.18 (2.98-3.38) [/1000]
Anencephaly	2016	1	317114	30	9.46 (6.38-13.50) [/100,000]
Anencephaly	2016	2	306225	37	12.08 (8.51-16.65) [/100,000]
Anencephaly	2016	3	328615	26	7.91 (5.17-11.59) [/100,000]
Anencephaly	2016	4	313767	32	10.20 (6.98-14.40) [/100,000]
Anencephaly	2016	5	327877	24	7.32 (4.69-10.89) [/100,000]
Anencephaly	2016	6	332521	33	9.92 (6.83-13.94) [/100,000]
Anencephaly	2016	7	343039	43	12.54 (9.07-16.88) [/100,000]
Anencephaly	2016	8	358174	34	9.49 (6.57-13.26) [/100,000]
Anencephaly	2016	9	346017	28	8.09 (5.38-11.70) [/100,000]
Anencephaly	2016	10	331330	20	6.04 (3.69-9.32) [/100,000]
Anencephaly	2016	11	319684	30	9.38 (6.33-13.40) [/100,000]
Anencephaly	2016	12	324921	29	8.93 (5.98-12.82) [/100,000]
Anencephaly	2017	1	314080	37	11.78 (8.29-16.24) [/100,000]
Anencephaly	2017	2	289301	24	8.30 (5.32-12.34) [/100,000]
Anencephaly	2017	3	319946	42	13.13 (9.46-17.74) [/100,000]
Anencephaly	2017	4	300459	30	9.98 (6.74-14.25) [/100,000]
Anencephaly	2017	5	322813	35	10.84 (7.55-15.08) [/100,000]
Anencephaly	2017	6	324241	28	8.64 (5.74-12.48) [/100,000]
Anencephaly	2017	7	335296	35	10.44 (7.27-14.52) [/100,000]
Anencephaly	2017	8	352660	46	13.04 (9.55-17.40) [/100,000]
Anencephaly	2017	9	337673	55	16.29 (12.27-21.20) [/100,000]
Anencephaly	2017	10	330310	32	9.69 (6.63-13.68) [/100,000]
Anencephaly	2017	11	316776	33	10.42 (7.17-14.63) [/100,000]
Anencephaly	2017	12	316262	35	11.07 (7.71-15.39) [/100,000]
Anencephaly	2018	1	315049	29	9.20 (6.16-13.22) [/100,000]
Anencephaly	2018	2	284508	25	8.79 (5.69-12.97) [/100,000]
Anencephaly	2018	3	316374	31	9.80 (6.66-13.91) [/100,000]
Anencephaly	2018	4	298702	34	11.38 (7.88-15.91) [/100,000]
Anencephaly	2018	5	320843	33	10.29 (7.08-14.44) [/100,000]
Anencephaly	2018	6	314959	34	10.80 (7.48-15.08) [/100,000]
Anencephaly	2018	7	329189	26	7.90 (5.16-11.57) [/100,000]
Anencephaly	2018	8	345135	24	6.95 (4.46-10.35) [/100,000]
Anencephaly	2018	9	323243	34	10.52 (7.28-14.70) [/100,000]
Anencephaly	2018	10	327299	25	7.64 (4.94-11.28) [/100,000]
Anencephaly	2018	11	309004	36	11.65 (8.16-16.13) [/100,000]
Anencephaly	2018	12	311122	23	7.39 (4.69-11.09) [/100,000]
Anencephaly	2019	1	311189	24	7.71 (4.94-11.48) [/100,000]
Anencephaly	2019	2	280254	26	9.28 (6.06-13.59) [/100,000]
Anencephaly	2019	3	304527	31	10.18 (6.92-14.45) [/100,000]
Anencephaly	2019	4	299272	21	7.02 (4.34-10.73) [/100,000]
Anencephaly	2019	5	316562	32	10.11 (6.91-14.27) [/100,000]
Anencephaly	2019	6	304319	29	9.53 (6.38-13.69) [/100,000]
Anencephaly	2019	7	334075	26	7.78 (5.08-11.40) [/100,000]
Anencephaly	2019	8	342126	25	7.31 (4.73-10.79) [/100,000]
Anencephaly	2019	9	326234	36	11.04 (7.73-15.28) [/100,000]
Anencephaly	2019	10	325771	27	8.29 (5.46-12.06) [/100,000]
Anencephaly	2019	11	298501	33	11.06 (7.61-15.53) [/100,000]
Anencephaly	2019	12	309284	23	7.44 (4.71-11.16) [/100,000]
Anencephaly	2020	1	305211	28	9.17 (6.10-13.26) [/100,000]
Anencephaly	2020	2	283058	27	9.54 (6.29-13.88) [/100,000]
Anencephaly	2020	3	302012	30	9.93 (6.70-14.18) [/100,000]
Anencephaly	2020	4	290578	26	8.95 (5.85-13.11) [/100,000]
Anencephaly	2020	5	301454	27	8.96 (5.90-13.03) [/100,000]
Anencephaly	2020	6	302144	27	8.94 (5.89-13.00) [/100,000]
Anencephaly	2020	7	321591	31	9.64 (6.55-13.68) [/100,000]

Anencephaly	2020	8	319610	31	9.70 (6.59-13.77) [/100,000]
Anencephaly	2020	9	311656	22	7.06 (4.42-10.69) [/100,000]
Anencephaly	2020	10	305015	25	8.20 (5.30-12.10) [/100,000]
Anencephaly	2020	11	282530	34	12.03 (8.33-16.82) [/100,000]
Anencephaly	2020	12	289802	24	8.28 (5.31-12.32) [/100,000]
Anencephaly	2021	1	277054	24	8.66 (5.55-12.89) [/100,000]
Anencephaly	2021	2	266235	22	8.26 (5.18-12.51) [/100,000]
Anencephaly	2021	3	302457	22	7.27 (4.56-11.01) [/100,000]
Anencephaly	2021	4	293104	29	9.89 (6.63-14.21) [/100,000]
Anencephaly	2021	5	300804	27	8.98 (5.92-13.06) [/100,000]
Anencephaly	2021	6	313498	18	5.74 (3.40-9.07) [/100,000]
Anencephaly	2021	7	325951	35	10.74 (7.48-14.93) [/100,000]
Anencephaly	2021	8	329954	23	6.97 (4.42-10.46) [/100,000]
Anencephaly	2021	9	325613	29	8.91 (5.96-12.79) [/100,000]
Anencephaly	2021	10	314971	23	7.30 (4.63-10.96) [/100,000]
Anencephaly	2021	11	301665	22	7.29 (4.57-11.04) [/100,000]
Anencephaly	2021	12	311074	36	11.57 (8.11-16.02) [/100,000]
Anencephaly	2022	1	294903	28	9.49 (6.31-13.72) [/100,000]
Anencephaly	2022	2	275665	32	11.61 (7.94-16.39) [/100,000]
Anencephaly	2022	3	307473	28	9.11 (6.05-13.16) [/100,000]
Anencephaly	2022	4	288688	28	9.70 (6.45-14.02) [/100,000]
Anencephaly	2022	5	297684	45	15.12 (11.03-20.23) [/100,000]
Anencephaly	2022	6	305797	30	9.81 (6.62-14.00) [/100,000]
Anencephaly	2022	7	320845	32	9.97 (6.82-14.08) [/100,000]
Anencephaly	2022	8	336052	39	11.61 (8.25-15.86) [/100,000]
Anencephaly	2022	9	312405	30	9.60 (6.48-13.71) [/100,000]
Anencephaly	2022	10	306926	29	9.45 (6.33-13.57) [/100,000]
Anencephaly	2022	11	307231	28	9.11 (6.06-13.17) [/100,000]
Anencephaly	2022	12	314859	38	12.07 (8.54-16.57) [/100,000]
Meningomyelocele/Spina bifida	2016	1	317114	39	12.30 (8.75-16.81) [/100,000]
Meningomyelocele/Spina bifida	2016	2	306225	52	16.98 (12.68-22.27) [/100,000]
Meningomyelocele/Spina bifida	2016	3	328615	40	12.17 (8.70-16.57) [/100,000]
Meningomyelocele/Spina bifida	2016	4	313767	46	14.66 (10.73-19.55) [/100,000]
Meningomyelocele/Spina bifida	2016	5	327877	50	15.25 (11.32-20.10) [/100,000]
Meningomyelocele/Spina bifida	2016	6	332521	44	13.23 (9.61-17.76) [/100,000]
Meningomyelocele/Spina bifida	2016	7	343039	51	14.87 (11.07-19.55) [/100,000]
Meningomyelocele/Spina bifida	2016	8	358174	51	14.24 (10.60-18.72) [/100,000]
Meningomyelocele/Spina bifida	2016	9	346017	46	13.29 (9.73-17.73) [/100,000]
Meningomyelocele/Spina bifida	2016	10	331330	43	12.98 (9.39-17.48) [/100,000]
Meningomyelocele/Spina bifida	2016	11	319684	42	13.14 (9.47-17.76) [/100,000]
Meningomyelocele/Spina bifida	2016	12	324921	44	13.54 (9.84-18.18) [/100,000]
Meningomyelocele/Spina bifida	2017	1	314080	43	13.69 (9.91-18.44) [/100,000]
Meningomyelocele/Spina bifida	2017	2	289301	54	18.67 (14.02-24.35) [/100,000]
Meningomyelocele/Spina bifida	2017	3	319946	48	15.00 (11.06-19.89) [/100,000]
Meningomyelocele/Spina bifida	2017	4	300459	43	14.31 (10.36-19.28) [/100,000]
Meningomyelocele/Spina bifida	2017	5	322813	53	16.42 (12.30-21.47) [/100,000]
Meningomyelocele/Spina bifida	2017	6	324241	44	13.57 (9.86-18.22) [/100,000]
Meningomyelocele/Spina bifida	2017	7	335296	56	16.70 (12.62-21.69) [/100,000]
Meningomyelocele/Spina bifida	2017	8	352660	58	16.45 (12.49-21.26) [/100,000]
Meningomyelocele/Spina bifida	2017	9	337673	48	14.21 (10.48-18.85) [/100,000]
Meningomyelocele/Spina bifida	2017	10	330310	42	12.72 (9.16-17.19) [/100,000]
Meningomyelocele/Spina bifida	2017	11	316776	49	15.47 (11.44-20.45) [/100,000]
Meningomyelocele/Spina bifida	2017	12	316262	58	18.34 (13.93-23.71) [/100,000]
Meningomyelocele/Spina bifida	2018	1	315049	55	17.46 (13.15-22.72) [/100,000]
Meningomyelocele/Spina bifida	2018	2	284508	45	15.82 (11.54-21.16) [/100,000]
Meningomyelocele/Spina bifida	2018	3	316374	48	15.17 (11.19-20.12) [/100,000]
Meningomyelocele/Spina bifida	2018	4	298702	45	15.07 (10.99-20.16) [/100,000]
Meningomyelocele/Spina bifida	2018	5	320843	40	12.47 (8.91-16.98) [/100,000]
Meningomyelocele/Spina bifida	2018	6	314959	48	15.24 (11.24-20.21) [/100,000]
Meningomyelocele/Spina bifida	2018	7	329189	43	13.06 (9.45-17.59) [/100,000]
Meningomyelocele/Spina bifida	2018	8	345135	41	11.88 (8.53-16.12) [/100,000]
Meningomyelocele/Spina bifida	2018	9	323243	44	13.61 (9.89-18.27) [/100,000]
Meningomyelocele/Spina bifida	2018	10	327299	43	13.14 (9.51-17.70) [/100,000]
Meningomyelocele/Spina bifida	2018	11	309004	55	17.80 (13.41-23.17) [/100,000]
Meningomyelocele/Spina bifida	2018	12	311122	48	15.43 (11.38-20.45) [/100,000]
Meningomyelocele/Spina bifida	2019	1	311189	40	12.85 (9.18-17.50) [/100,000]
Meningomyelocele/Spina bifida	2019	2	280254	37	13.20 (9.30-18.20) [/100,000]
Meningomyelocele/Spina bifida	2019	3	304527	40	13.14 (9.38-17.89) [/100,000]
Meningomyelocele/Spina bifida	2019	4	299272	50	16.71 (12.40-22.03) [/100,000]
Meningomyelocele/Spina bifida	2019	5	316562	41	12.95 (9.29-17.57) [/100,000]
Meningomyelocele/Spina bifida	2019	6	304319	55	18.07 (13.62-23.52) [/100,000]

Meningomyelocele/Spina bifida	2019	7	334075	57	17.06 (12.92-22.11) [/100,000]
Meningomyelocele/Spina bifida	2019	8	342126	51	14.91 (11.10-19.60) [/100,000]
Meningomyelocele/Spina bifida	2019	9	326234	44	13.49 (9.80-18.11) [/100,000]
Meningomyelocele/Spina bifida	2019	10	325771	51	15.66 (11.66-20.58) [/100,000]
Meningomyelocele/Spina bifida	2019	11	298501	42	14.07 (10.14-19.02) [/100,000]
Meningomyelocele/Spina bifida	2019	12	309284	31	10.02 (6.81-14.23) [/100,000]
Meningomyelocele/Spina bifida	2020	1	305211	46	15.07 (11.03-20.10) [/100,000]
Meningomyelocele/Spina bifida	2020	2	283058	32	11.31 (7.73-15.96) [/100,000]
Meningomyelocele/Spina bifida	2020	3	302012	45	14.90 (10.87-19.94) [/100,000]
Meningomyelocele/Spina bifida	2020	4	290578	38	13.08 (9.25-17.95) [/100,000]
Meningomyelocele/Spina bifida	2020	5	301454	37	12.27 (8.64-16.92) [/100,000]
Meningomyelocele/Spina bifida	2020	6	302144	31	10.26 (6.97-14.56) [/100,000]
Meningomyelocele/Spina bifida	2020	7	321591	45	13.99 (10.21-18.72) [/100,000]
Meningomyelocele/Spina bifida	2020	8	319610	45	14.08 (10.27-18.84) [/100,000]
Meningomyelocele/Spina bifida	2020	9	311656	35	11.23 (7.82-15.62) [/100,000]
Meningomyelocele/Spina bifida	2020	10	305015	44	14.43 (10.48-19.37) [/100,000]
Meningomyelocele/Spina bifida	2020	11	282530	42	14.87 (10.71-20.09) [/100,000]
Meningomyelocele/Spina bifida	2020	12	289802	50	17.25 (12.81-22.75) [/100,000]
Meningomyelocele/Spina bifida	2021	1	277054	41	14.80 (10.62-20.08) [/100,000]
Meningomyelocele/Spina bifida	2021	2	266235	39	14.65 (10.42-20.02) [/100,000]
Meningomyelocele/Spina bifida	2021	3	302457	40	13.23 (9.45-18.01) [/100,000]
Meningomyelocele/Spina bifida	2021	4	293104	37	12.62 (8.89-17.40) [/100,000]
Meningomyelocele/Spina bifida	2021	5	300804	53	17.62 (13.20-23.05) [/100,000]
Meningomyelocele/Spina bifida	2021	6	313498	35	11.16 (7.78-15.53) [/100,000]
Meningomyelocele/Spina bifida	2021	7	325951	41	12.58 (9.03-17.06) [/100,000]
Meningomyelocele/Spina bifida	2021	8	329954	51	15.46 (11.51-20.32) [/100,000]
Meningomyelocele/Spina bifida	2021	9	325613	49	15.05 (11.13-19.89) [/100,000]
Meningomyelocele/Spina bifida	2021	10	314971	35	11.11 (7.74-15.45) [/100,000]
Meningomyelocele/Spina bifida	2021	11	301665	35	11.60 (8.08-16.14) [/100,000]
Meningomyelocele/Spina bifida	2021	12	311074	42	13.50 (9.73-18.25) [/100,000]
Meningomyelocele/Spina bifida	2022	1	294903	47	15.94 (11.71-21.19) [/100,000]
Meningomyelocele/Spina bifida	2022	2	275665	47	17.05 (12.53-22.67) [/100,000]
Meningomyelocele/Spina bifida	2022	3	307473	42	13.66 (9.84-18.46) [/100,000]
Meningomyelocele/Spina bifida	2022	4	288688	37	12.82 (9.02-17.67) [/100,000]
Meningomyelocele/Spina bifida	2022	5	297684	58	19.48 (14.80-25.19) [/100,000]
Meningomyelocele/Spina bifida	2022	6	305797	34	11.12 (7.70-15.54) [/100,000]
Meningomyelocele/Spina bifida	2022	7	320845	45	14.03 (10.23-18.77) [/100,000]
Meningomyelocele/Spina bifida	2022	8	336052	32	9.52 (6.51-13.44) [/100,000]
Meningomyelocele/Spina bifida	2022	9	312405	46	14.72 (10.78-19.64) [/100,000]
Meningomyelocele/Spina bifida	2022	10	306926	33	10.75 (7.40-15.10) [/100,000]
Meningomyelocele/Spina bifida	2022	11	307231	43	14.00 (10.13-18.85) [/100,000]
Meningomyelocele/Spina bifida	2022	12	314859	35	11.12 (7.74-15.46) [/100,000]
Cyanotic congenital heart disease	2016	1	317114	183	57.71 (49.65-66.70) [/100,000]
Cyanotic congenital heart disease	2016	2	306225	192	62.70 (54.15-72.22) [/100,000]
Cyanotic congenital heart disease	2016	3	328615	194	59.04 (51.02-67.95) [/100,000]
Cyanotic congenital heart disease	2016	4	313767	189	60.24 (51.96-69.46) [/100,000]
Cyanotic congenital heart disease	2016	5	327877	181	55.20 (47.46-63.86) [/100,000]
Cyanotic congenital heart disease	2016	6	332521	195	58.64 (50.70-67.47) [/100,000]
Cyanotic congenital heart disease	2016	7	343039	180	52.47 (45.09-60.72) [/100,000]
Cyanotic congenital heart disease	2016	8	358174	201	56.12 (48.63-64.43) [/100,000]
Cyanotic congenital heart disease	2016	9	346017	181	52.31 (44.97-60.51) [/100,000]
Cyanotic congenital heart disease	2016	10	331330	182	54.93 (47.24-63.51) [/100,000]
Cyanotic congenital heart disease	2016	11	319684	147	45.98 (38.85-54.04) [/100,000]
Cyanotic congenital heart disease	2016	12	324921	175	53.86 (46.18-62.45) [/100,000]
Cyanotic congenital heart disease	2017	1	314080	160	50.94 (43.36-59.47) [/100,000]
Cyanotic congenital heart disease	2017	2	289301	154	53.23 (45.16-62.33) [/100,000]
Cyanotic congenital heart disease	2017	3	319946	177	55.32 (47.47-64.10) [/100,000]
Cyanotic congenital heart disease	2017	4	300459	151	50.26 (42.56-58.94) [/100,000]
Cyanotic congenital heart disease	2017	5	322813	185	57.31 (49.35-66.19) [/100,000]
Cyanotic congenital heart disease	2017	6	324241	194	59.83 (51.71-68.87) [/100,000]
Cyanotic congenital heart disease	2017	7	335296	197	58.75 (50.84-67.55) [/100,000]
Cyanotic congenital heart disease	2017	8	352660	191	54.16 (46.75-62.41) [/100,000]
Cyanotic congenital heart disease	2017	9	337673	185	54.79 (47.18-63.27) [/100,000]
Cyanotic congenital heart disease	2017	10	330310	196	59.34 (51.32-68.25) [/100,000]
Cyanotic congenital heart disease	2017	11	316776	173	54.61 (46.78-63.38) [/100,000]
Cyanotic congenital heart disease	2017	12	316262	167	52.80 (45.10-61.45) [/100,000]
Cyanotic congenital heart disease	2018	1	315049	172	54.59 (46.74-63.39) [/100,000]
Cyanotic congenital heart disease	2018	2	284508	175	61.51 (52.74-71.33) [/100,000]
Cyanotic congenital heart disease	2018	3	316374	182	57.53 (49.47-66.52) [/100,000]
Cyanotic congenital heart disease	2018	4	298702	186	62.27 (53.64-71.89) [/100,000]
Cyanotic congenital heart disease	2018	5	320843	187	58.28 (50.23-67.26) [/100,000]

Cyanotic congenital heart disease	2018	6	314959	189	60.01 (51.76-69.20) [/100,000]
Cyanotic congenital heart disease	2018	7	329189	198	60.15 (52.06-69.13) [/100,000]
Cyanotic congenital heart disease	2018	8	345135	189	54.76 (47.23-63.15) [/100,000]
Cyanotic congenital heart disease	2018	9	323243	147	45.48 (38.42-53.45) [/100,000]
Cyanotic congenital heart disease	2018	10	327299	207	63.24 (54.92-72.47) [/100,000]
Cyanotic congenital heart disease	2018	11	309004	188	60.84 (52.46-70.18) [/100,000]
Cyanotic congenital heart disease	2018	12	311122	196	63.00 (54.49-72.46) [/100,000]
Cyanotic congenital heart disease	2019	1	311189	171	54.95 (47.02-63.83) [/100,000]
Cyanotic congenital heart disease	2019	2	280254	157	56.02 (47.60-65.50) [/100,000]
Cyanotic congenital heart disease	2019	3	304527	191	62.72 (54.14-72.27) [/100,000]
Cyanotic congenital heart disease	2019	4	299272	182	60.81 (52.30-70.32) [/100,000]
Cyanotic congenital heart disease	2019	5	316562	187	59.07 (50.91-68.17) [/100,000]
Cyanotic congenital heart disease	2019	6	304319	195	64.08 (55.40-73.73) [/100,000]
Cyanotic congenital heart disease	2019	7	334075	177	52.98 (45.47-61.39) [/100,000]
Cyanotic congenital heart disease	2019	8	342126	210	61.38 (53.36-70.26) [/100,000]
Cyanotic congenital heart disease	2019	9	326234	183	56.09 (48.26-64.83) [/100,000]
Cyanotic congenital heart disease	2019	10	325771	203	62.31 (54.04-71.50) [/100,000]
Cyanotic congenital heart disease	2019	11	298501	172	57.62 (49.33-66.90) [/100,000]
Cyanotic congenital heart disease	2019	12	309284	193	62.40 (53.91-71.85) [/100,000]
Cyanotic congenital heart disease	2020	1	305211	168	55.04 (47.04-64.02) [/100,000]
Cyanotic congenital heart disease	2020	2	283058	154	54.41 (46.15-63.71) [/100,000]
Cyanotic congenital heart disease	2020	3	302012	212	70.20 (61.07-80.30) [/100,000]
Cyanotic congenital heart disease	2020	4	290578	190	65.39 (56.42-75.37) [/100,000]
Cyanotic congenital heart disease	2020	5	301454	192	63.69 (55.00-73.36) [/100,000]
Cyanotic congenital heart disease	2020	6	302144	211	69.83 (60.73-79.92) [/100,000]
Cyanotic congenital heart disease	2020	7	321591	216	67.17 (58.51-76.74) [/100,000]
Cyanotic congenital heart disease	2020	8	319610	208	65.08 (56.54-74.55) [/100,000]
Cyanotic congenital heart disease	2020	9	311656	214	68.67 (59.78-78.50) [/100,000]
Cyanotic congenital heart disease	2020	10	305015	179	58.69 (50.41-67.94) [/100,000]
Cyanotic congenital heart disease	2020	11	282530	153	54.15 (45.91-63.44) [/100,000]
Cyanotic congenital heart disease	2020	12	289802	191	65.91 (56.89-75.94) [/100,000]
Cyanotic congenital heart disease	2021	1	277054	184	66.41 (57.17-76.73) [/100,000]
Cyanotic congenital heart disease	2021	2	266235	207	77.75 (67.52-89.09) [/100,000]
Cyanotic congenital heart disease	2021	3	302457	247	81.66 (71.80-92.50) [/100,000]
Cyanotic congenital heart disease	2021	4	293104	225	76.76 (67.06-87.47) [/100,000]
Cyanotic congenital heart disease	2021	5	300804	193	64.16 (55.43-73.88) [/100,000]
Cyanotic congenital heart disease	2021	6	313498	187	59.65 (51.41-68.84) [/100,000]
Cyanotic congenital heart disease	2021	7	325951	209	64.12 (55.72-73.42) [/100,000]
Cyanotic congenital heart disease	2021	8	329954	238	72.13 (63.26-81.90) [/100,000]
Cyanotic congenital heart disease	2021	9	325613	210	64.49 (56.07-73.83) [/100,000]
Cyanotic congenital heart disease	2021	10	314971	213	67.63 (58.85-77.34) [/100,000]
Cyanotic congenital heart disease	2021	11	301665	198	65.64 (56.81-75.44) [/100,000]
Cyanotic congenital heart disease	2021	12	311074	179	57.54 (49.42-66.61) [/100,000]
Cyanotic congenital heart disease	2022	1	294903	186	63.07 (54.34-72.81) [/100,000]
Cyanotic congenital heart disease	2022	2	275665	188	68.20 (58.80-78.67) [/100,000]
Cyanotic congenital heart disease	2022	3	307473	213	69.27 (60.29-79.22) [/100,000]
Cyanotic congenital heart disease	2022	4	288688	194	67.20 (58.08-77.35) [/100,000]
Cyanotic congenital heart disease	2022	5	297684	195	65.51 (56.64-75.37) [/100,000]
Cyanotic congenital heart disease	2022	6	305797	216	70.64 (61.53-80.71) [/100,000]
Cyanotic congenital heart disease	2022	7	320845	231	72.00 (63.01-81.90) [/100,000]
Cyanotic congenital heart disease	2022	8	336052	234	69.63 (61.00-79.15) [/100,000]
Cyanotic congenital heart disease	2022	9	312405	211	67.54 (58.74-77.29) [/100,000]
Cyanotic congenital heart disease	2022	10	306926	186	60.60 (52.21-69.96) [/100,000]
Cyanotic congenital heart disease	2022	11	307231	177	57.61 (49.44-66.75) [/100,000]
Cyanotic congenital heart disease	2022	12	314859	195	61.93 (53.55-71.26) [/100,000]
Congenital diaphragmatic hernia	2016	1	317114	31	9.78 (6.64-13.88) [/100,000]
Congenital diaphragmatic hernia	2016	2	306225	46	15.02 (11.00-20.04) [/100,000]
Congenital diaphragmatic hernia	2016	3	328615	50	15.22 (11.29-20.06) [/100,000]
Congenital diaphragmatic hernia	2016	4	313767	37	11.79 (8.30-16.25) [/100,000]
Congenital diaphragmatic hernia	2016	5	327877	37	11.28 (7.95-15.55) [/100,000]
Congenital diaphragmatic hernia	2016	6	332521	53	15.94 (11.94-20.85) [/100,000]
Congenital diaphragmatic hernia	2016	7	343039	57	16.62 (12.59-21.53) [/100,000]
Congenital diaphragmatic hernia	2016	8	358174	47	13.12 (9.64-17.45) [/100,000]
Congenital diaphragmatic hernia	2016	9	346017	29	8.38 (5.61-12.04) [/100,000]
Congenital diaphragmatic hernia	2016	10	331330	35	10.56 (7.36-14.69) [/100,000]
Congenital diaphragmatic hernia	2016	11	319684	38	11.89 (8.41-16.32) [/100,000]
Congenital diaphragmatic hernia	2016	12	324921	41	12.62 (9.06-17.12) [/100,000]
Congenital diaphragmatic hernia	2017	1	314080	32	10.19 (6.97-14.38) [/100,000]
Congenital diaphragmatic hernia	2017	2	289301	42	14.52 (10.46-19.62) [/100,000]
Congenital diaphragmatic hernia	2017	3	319946	45	14.06 (10.26-18.82) [/100,000]
Congenital diaphragmatic hernia	2017	4	300459	37	12.31 (8.67-16.97) [/100,000]

Congenital diaphragmatic hernia	2017	5	322813	33	10.22 (7.04-14.36) [/100,000]
Congenital diaphragmatic hernia	2017	6	324241	41	12.64 (9.07-17.15) [/100,000]
Congenital diaphragmatic hernia	2017	7	335296	55	16.40 (12.36-21.35) [/100,000]
Congenital diaphragmatic hernia	2017	8	352660	45	12.76 (9.31-17.07) [/100,000]
Congenital diaphragmatic hernia	2017	9	337673	33	9.77 (6.73-13.72) [/100,000]
Congenital diaphragmatic hernia	2017	10	330310	41	12.41 (8.91-16.84) [/100,000]
Congenital diaphragmatic hernia	2017	11	316776	33	10.42 (7.17-14.63) [/100,000]
Congenital diaphragmatic hernia	2017	12	316262	34	10.75 (7.45-15.02) [/100,000]
Congenital diaphragmatic hernia	2018	1	315049	46	14.60 (10.69-19.48) [/100,000]
Congenital diaphragmatic hernia	2018	2	284508	35	12.30 (8.57-17.11) [/100,000]
Congenital diaphragmatic hernia	2018	3	316374	41	12.96 (9.30-17.58) [/100,000]
Congenital diaphragmatic hernia	2018	4	298702	36	12.05 (8.44-16.68) [/100,000]
Congenital diaphragmatic hernia	2018	5	320843	46	14.34 (10.50-19.12) [/100,000]
Congenital diaphragmatic hernia	2018	6	314959	35	11.11 (7.74-15.45) [/100,000]
Congenital diaphragmatic hernia	2018	7	329189	34	10.33 (7.15-14.43) [/100,000]
Congenital diaphragmatic hernia	2018	8	345135	44	12.75 (9.26-17.11) [/100,000]
Congenital diaphragmatic hernia	2018	9	323243	40	12.37 (8.84-16.85) [/100,000]
Congenital diaphragmatic hernia	2018	10	327299	45	13.75 (10.03-18.40) [/100,000]
Congenital diaphragmatic hernia	2018	11	309004	39	12.62 (8.98-17.25) [/100,000]
Congenital diaphragmatic hernia	2018	12	311122	31	9.96 (6.77-14.14) [/100,000]
Congenital diaphragmatic hernia	2019	1	311189	39	12.53 (8.91-17.13) [/100,000]
Congenital diaphragmatic hernia	2019	2	280254	32	11.42 (7.81-16.12) [/100,000]
Congenital diaphragmatic hernia	2019	3	304527	43	14.12 (10.22-19.02) [/100,000]
Congenital diaphragmatic hernia	2019	4	299272	31	10.36 (7.04-14.70) [/100,000]
Congenital diaphragmatic hernia	2019	5	316562	40	12.64 (9.03-17.21) [/100,000]
Congenital diaphragmatic hernia	2019	6	304319	39	12.82 (9.11-17.52) [/100,000]
Congenital diaphragmatic hernia	2019	7	334075	52	15.57 (11.63-20.41) [/100,000]
Congenital diaphragmatic hernia	2019	8	342126	38	11.11 (7.86-15.24) [/100,000]
Congenital diaphragmatic hernia	2019	9	326234	41	12.57 (9.02-17.05) [/100,000]
Congenital diaphragmatic hernia	2019	10	325771	45	13.81 (10.08-18.48) [/100,000]
Congenital diaphragmatic hernia	2019	11	298501	31	10.39 (7.06-14.74) [/100,000]
Congenital diaphragmatic hernia	2019	12	309284	41	13.26 (9.51-17.98) [/100,000]
Congenital diaphragmatic hernia	2020	1	305211	42	13.76 (9.92-18.60) [/100,000]
Congenital diaphragmatic hernia	2020	2	283058	29	10.25 (6.86-14.71) [/100,000]
Congenital diaphragmatic hernia	2020	3	302012	43	14.24 (10.30-19.18) [/100,000]
Congenital diaphragmatic hernia	2020	4	290578	44	15.14 (11.00-20.33) [/100,000]
Congenital diaphragmatic hernia	2020	5	301454	35	11.61 (8.09-16.15) [/100,000]
Congenital diaphragmatic hernia	2020	6	302144	44	14.56 (10.58-19.55) [/100,000]
Congenital diaphragmatic hernia	2020	7	321591	39	12.13 (8.62-16.58) [/100,000]
Congenital diaphragmatic hernia	2020	8	319610	36	11.26 (7.89-15.59) [/100,000]
Congenital diaphragmatic hernia	2020	9	311656	41	13.16 (9.44-17.85) [/100,000]
Congenital diaphragmatic hernia	2020	10	305015	42	13.77 (9.92-18.61) [/100,000]
Congenital diaphragmatic hernia	2020	11	282530	42	14.87 (10.71-20.09) [/100,000]
Congenital diaphragmatic hernia	2020	12	289802	32	11.04 (7.55-15.59) [/100,000]
Congenital diaphragmatic hernia	2021	1	277054	46	16.60 (12.16-22.15) [/100,000]
Congenital diaphragmatic hernia	2021	2	266235	34	12.77 (8.84-17.85) [/100,000]
Congenital diaphragmatic hernia	2021	3	302457	43	14.22 (10.29-19.15) [/100,000]
Congenital diaphragmatic hernia	2021	4	293104	44	15.01 (10.91-20.15) [/100,000]
Congenital diaphragmatic hernia	2021	5	300804	26	8.64 (5.65-12.66) [/100,000]
Congenital diaphragmatic hernia	2021	6	313498	37	11.80 (8.31-16.27) [/100,000]
Congenital diaphragmatic hernia	2021	7	325951	26	7.98 (5.21-11.69) [/100,000]
Congenital diaphragmatic hernia	2021	8	329954	38	11.52 (8.15-15.81) [/100,000]
Congenital diaphragmatic hernia	2021	9	325613	46	14.13 (10.34-18.84) [/100,000]
Congenital diaphragmatic hernia	2021	10	314971	42	13.33 (9.61-18.02) [/100,000]
Congenital diaphragmatic hernia	2021	11	301665	35	11.60 (8.08-16.14) [/100,000]
Congenital diaphragmatic hernia	2021	12	311074	45	14.47 (10.55-19.36) [/100,000]
Congenital diaphragmatic hernia	2022	1	294903	42	14.24 (10.26-19.25) [/100,000]
Congenital diaphragmatic hernia	2022	2	275665	32	11.61 (7.94-16.39) [/100,000]
Congenital diaphragmatic hernia	2022	3	307473	42	13.66 (9.84-18.46) [/100,000]
Congenital diaphragmatic hernia	2022	4	288688	49	16.97 (12.56-22.44) [/100,000]
Congenital diaphragmatic hernia	2022	5	297684	36	12.09 (8.47-16.74) [/100,000]
Congenital diaphragmatic hernia	2022	6	305797	37	12.10 (8.52-16.68) [/100,000]
Congenital diaphragmatic hernia	2022	7	320845	28	8.73 (5.80-12.61) [/100,000]
Congenital diaphragmatic hernia	2022	8	336052	51	15.18 (11.30-19.95) [/100,000]
Congenital diaphragmatic hernia	2022	9	312405	47	15.04 (11.05-20.01) [/100,000]
Congenital diaphragmatic hernia	2022	10	306926	32	10.43 (7.13-14.72) [/100,000]
Congenital diaphragmatic hernia	2022	11	307231	40	13.02 (9.30-17.73) [/100,000]
Congenital diaphragmatic hernia	2022	12	314859	50	15.88 (11.79-20.94) [/100,000]
Omphalocele	2016	1	317114	32	10.09 (6.90-14.25) [/100,000]
Omphalocele	2016	2	306225	26	8.49 (5.55-12.44) [/100,000]
Omphalocele	2016	3	328615	45	13.69 (9.99-18.32) [/100,000]

Omphalocele	2016	4	313767	35	11.15 (7.77-15.51) [/100,000]
Omphalocele	2016	5	327877	43	13.11 (9.49-17.66) [/100,000]
Omphalocele	2016	6	332521	30	9.02 (6.09-12.88) [/100,000]
Omphalocele	2016	7	343039	35	10.20 (7.11-14.19) [/100,000]
Omphalocele	2016	8	358174	33	9.21 (6.34-12.94) [/100,000]
Omphalocele	2016	9	346017	25	7.23 (4.68-10.67) [/100,000]
Omphalocele	2016	10	331330	29	8.75 (5.86-12.57) [/100,000]
Omphalocele	2016	11	319684	30	9.38 (6.33-13.40) [/100,000]
Omphalocele	2016	12	324921	41	12.62 (9.06-17.12) [/100,000]
Omphalocele	2017	1	314080	31	9.87 (6.71-14.01) [/100,000]
Omphalocele	2017	2	289301	35	12.10 (8.43-16.83) [/100,000]
Omphalocele	2017	3	319946	37	11.56 (8.14-15.94) [/100,000]
Omphalocele	2017	4	300459	30	9.98 (6.74-14.25) [/100,000]
Omphalocele	2017	5	322813	29	8.98 (6.02-12.90) [/100,000]
Omphalocele	2017	6	324241	31	9.56 (6.50-13.57) [/100,000]
Omphalocele	2017	7	335296	23	6.86 (4.35-10.29) [/100,000]
Omphalocele	2017	8	352660	41	11.63 (8.34-15.77) [/100,000]
Omphalocele	2017	9	337673	34	10.07 (6.97-14.07) [/100,000]
Omphalocele	2017	10	330310	38	11.50 (8.14-15.79) [/100,000]
Omphalocele	2017	11	316776	32	10.10 (6.91-14.26) [/100,000]
Omphalocele	2017	12	316262	39	12.33 (8.77-16.86) [/100,000]
Omphalocele	2018	1	315049	39	12.38 (8.80-16.92) [/100,000]
Omphalocele	2018	2	284508	31	10.90 (7.40-15.47) [/100,000]
Omphalocele	2018	3	316374	47	14.86 (10.92-19.75) [/100,000]
Omphalocele	2018	4	298702	32	10.71 (7.33-15.12) [/100,000]
Omphalocele	2018	5	320843	24	7.48 (4.79-11.13) [/100,000]
Omphalocele	2018	6	314959	33	10.48 (7.21-14.71) [/100,000]
Omphalocele	2018	7	329189	21	6.38 (3.95-9.75) [/100,000]
Omphalocele	2018	8	345135	43	12.46 (9.02-16.78) [/100,000]
Omphalocele	2018	9	323243	37	11.45 (8.06-15.78) [/100,000]
Omphalocele	2018	10	327299	31	9.47 (6.44-13.44) [/100,000]
Omphalocele	2018	11	309004	39	12.62 (8.98-17.25) [/100,000]
Omphalocele	2018	12	311122	31	9.96 (6.77-14.14) [/100,000]
Omphalocele	2019	1	311189	28	9.00 (5.98-13.00) [/100,000]
Omphalocele	2019	2	280254	34	12.13 (8.40-16.95) [/100,000]
Omphalocele	2019	3	304527	27	8.87 (5.84-12.90) [/100,000]
Omphalocele	2019	4	299272	41	13.70 (9.83-18.59) [/100,000]
Omphalocele	2019	5	316562	34	10.74 (7.44-15.01) [/100,000]
Omphalocele	2019	6	304319	31	10.19 (6.92-14.46) [/100,000]
Omphalocele	2019	7	334075	34	10.18 (7.05-14.22) [/100,000]
Omphalocele	2019	8	342126	34	9.94 (6.88-13.89) [/100,000]
Omphalocele	2019	9	326234	41	12.57 (9.02-17.05) [/100,000]
Omphalocele	2019	10	325771	33	10.13 (6.97-14.23) [/100,000]
Omphalocele	2019	11	298501	30	10.05 (6.78-14.35) [/100,000]
Omphalocele	2019	12	309284	30	9.70 (6.54-13.85) [/100,000]
Omphalocele	2020	1	305211	27	8.85 (5.83-12.87) [/100,000]
Omphalocele	2020	2	283058	25	8.83 (5.72-13.04) [/100,000]
Omphalocele	2020	3	302012	27	8.94 (5.89-13.01) [/100,000]
Omphalocele	2020	4	290578	26	8.95 (5.85-13.11) [/100,000]
Omphalocele	2020	5	301454	35	11.61 (8.09-16.15) [/100,000]
Omphalocele	2020	6	302144	31	10.26 (6.97-14.56) [/100,000]
Omphalocele	2020	7	321591	24	7.46 (4.78-11.10) [/100,000]
Omphalocele	2020	8	319610	26	8.13 (5.31-11.92) [/100,000]
Omphalocele	2020	9	311656	37	11.87 (8.36-16.36) [/100,000]
Omphalocele	2020	10	305015	24	7.87 (5.04-11.71) [/100,000]
Omphalocele	2020	11	282530	27	9.56 (6.30-13.90) [/100,000]
Omphalocele	2020	12	289802	27	9.32 (6.14-13.56) [/100,000]
Omphalocele	2021	1	277054	34	12.27 (8.50-17.15) [/100,000]
Omphalocele	2021	2	266235	26	9.77 (6.38-14.31) [/100,000]
Omphalocele	2021	3	302457	24	7.94 (5.08-11.81) [/100,000]
Omphalocele	2021	4	293104	34	11.60 (8.03-16.21) [/100,000]
Omphalocele	2021	5	300804	34	11.30 (7.83-15.79) [/100,000]
Omphalocele	2021	6	313498	44	14.04 (10.20-18.84) [/100,000]
Omphalocele	2021	7	325951	28	8.59 (5.71-12.42) [/100,000]
Omphalocele	2021	8	329954	31	9.40 (6.38-13.34) [/100,000]
Omphalocele	2021	9	325613	36	11.06 (7.74-15.31) [/100,000]
Omphalocele	2021	10	314971	29	9.21 (6.17-13.22) [/100,000]
Omphalocele	2021	11	301665	22	7.29 (4.57-11.04) [/100,000]
Omphalocele	2021	12	311074	34	10.93 (7.57-15.27) [/100,000]
Omphalocele	2022	1	294903	32	10.85 (7.42-15.32) [/100,000]
Omphalocele	2022	2	275665	23	8.34 (5.29-12.52) [/100,000]

Omphalocele	2022	3	307473	32	10.41 (7.12-14.69) [/100,000]
Omphalocele	2022	4	288688	22	7.62 (4.78-11.54) [/100,000]
Omphalocele	2022	5	297684	32	10.75 (7.35-15.17) [/100,000]
Omphalocele	2022	6	305797	27	8.83 (5.82-12.85) [/100,000]
Omphalocele	2022	7	320845	29	9.04 (6.05-12.98) [/100,000]
Omphalocele	2022	8	336052	33	9.82 (6.76-13.79) [/100,000]
Omphalocele	2022	9	312405	36	11.52 (8.07-15.95) [/100,000]
Omphalocele	2022	10	306926	33	10.75 (7.40-15.10) [/100,000]
Omphalocele	2022	11	307231	28	9.11 (6.06-13.17) [/100,000]
Omphalocele	2022	12	314859	23	7.30 (4.63-10.96) [/100,000]
Gastroschisis	2016	1	317114	87	27.43 (21.97-33.84) [/100,000]
Gastroschisis	2016	2	306225	78	25.47 (20.13-31.79) [/100,000]
Gastroschisis	2016	3	328615	94	28.60 (23.12-35.00) [/100,000]
Gastroschisis	2016	4	313767	67	21.35 (16.55-27.12) [/100,000]
Gastroschisis	2016	5	327877	89	27.14 (21.80-33.40) [/100,000]
Gastroschisis	2016	6	332521	69	20.75 (16.15-26.26) [/100,000]
Gastroschisis	2016	7	343039	74	21.57 (16.94-27.08) [/100,000]
Gastroschisis	2016	8	358174	83	23.17 (18.46-28.73) [/100,000]
Gastroschisis	2016	9	346017	92	26.59 (21.43-32.61) [/100,000]
Gastroschisis	2016	10	331330	67	20.22 (15.67-25.68) [/100,000]
Gastroschisis	2016	11	319684	81	25.34 (20.12-31.49) [/100,000]
Gastroschisis	2016	12	324921	78	24.01 (18.98-29.96) [/100,000]
Gastroschisis	2017	1	314080	96	30.57 (24.76-37.32) [/100,000]
Gastroschisis	2017	2	289301	62	21.43 (16.43-27.47) [/100,000]
Gastroschisis	2017	3	319946	70	21.88 (17.06-27.64) [/100,000]
Gastroschisis	2017	4	300459	71	23.63 (18.46-29.81) [/100,000]
Gastroschisis	2017	5	322813	75	23.23 (18.27-29.12) [/100,000]
Gastroschisis	2017	6	324241	93	28.68 (23.15-35.14) [/100,000]
Gastroschisis	2017	7	335296	87	25.95 (20.78-32.00) [/100,000]
Gastroschisis	2017	8	352660	75	21.27 (16.73-26.66) [/100,000]
Gastroschisis	2017	9	337673	74	21.91 (17.21-27.51) [/100,000]
Gastroschisis	2017	10	330310	66	19.98 (15.45-25.42) [/100,000]
Gastroschisis	2017	11	316776	89	28.10 (22.56-34.57) [/100,000]
Gastroschisis	2017	12	316262	68	21.50 (16.70-27.26) [/100,000]
Gastroschisis	2018	1	315049	73	23.17 (18.16-29.13) [/100,000]
Gastroschisis	2018	2	284508	55	19.33 (14.56-25.16) [/100,000]
Gastroschisis	2018	3	316374	72	22.76 (17.81-28.66) [/100,000]
Gastroschisis	2018	4	298702	75	25.11 (19.75-31.47) [/100,000]
Gastroschisis	2018	5	320843	80	24.93 (19.77-31.03) [/100,000]
Gastroschisis	2018	6	314959	75	23.81 (18.73-29.85) [/100,000]
Gastroschisis	2018	7	329189	74	22.48 (17.65-28.22) [/100,000]
Gastroschisis	2018	8	345135	62	17.96 (13.77-23.03) [/100,000]
Gastroschisis	2018	9	323243	66	20.42 (15.79-25.98) [/100,000]
Gastroschisis	2018	10	327299	65	19.86 (15.33-25.31) [/100,000]
Gastroschisis	2018	11	309004	62	20.06 (15.38-25.72) [/100,000]
Gastroschisis	2018	12	311122	71	22.82 (17.82-28.78) [/100,000]
Gastroschisis	2019	1	311189	66	21.21 (16.40-26.98) [/100,000]
Gastroschisis	2019	2	280254	69	24.62 (19.16-31.16) [/100,000]
Gastroschisis	2019	3	304527	67	22.00 (17.05-27.94) [/100,000]
Gastroschisis	2019	4	299272	54	18.04 (13.56-23.54) [/100,000]
Gastroschisis	2019	5	316562	75	23.69 (18.64-29.70) [/100,000]
Gastroschisis	2019	6	304319	74	24.32 (19.09-30.53) [/100,000]
Gastroschisis	2019	7	334075	68	20.35 (15.81-25.80) [/100,000]
Gastroschisis	2019	8	342126	76	22.21 (17.50-27.80) [/100,000]
Gastroschisis	2019	9	326234	86	26.36 (21.09-32.56) [/100,000]
Gastroschisis	2019	10	325771	59	18.11 (13.79-23.36) [/100,000]
Gastroschisis	2019	11	298501	64	21.44 (16.51-27.38) [/100,000]
Gastroschisis	2019	12	309284	71	22.96 (17.93-28.96) [/100,000]
Gastroschisis	2020	1	305211	58	19.00 (14.43-24.57) [/100,000]
Gastroschisis	2020	2	283058	61	21.55 (16.48-27.68) [/100,000]
Gastroschisis	2020	3	302012	60	19.87 (15.16-25.57) [/100,000]
Gastroschisis	2020	4	290578	66	22.71 (17.57-28.90) [/100,000]
Gastroschisis	2020	5	301454	62	20.57 (15.77-26.37) [/100,000]
Gastroschisis	2020	6	302144	63	20.85 (16.02-26.68) [/100,000]
Gastroschisis	2020	7	321591	69	21.46 (16.69-27.15) [/100,000]
Gastroschisis	2020	8	319610	54	16.90 (12.69-22.04) [/100,000]
Gastroschisis	2020	9	311656	54	17.33 (13.02-22.61) [/100,000]
Gastroschisis	2020	10	305015	72	23.61 (18.47-29.73) [/100,000]
Gastroschisis	2020	11	282530	65	23.01 (17.76-29.32) [/100,000]
Gastroschisis	2020	12	289802	62	21.39 (16.40-27.43) [/100,000]
Gastroschisis	2021	1	277054	65	23.46 (18.11-29.90) [/100,000]

Gastroschisis	2021	2	266235	56	21.03 (15.89-27.31) [/100,000]
Gastroschisis	2021	3	302457	59	19.51 (14.85-25.16) [/100,000]
Gastroschisis	2021	4	293104	47	16.04 (11.78-21.32) [/100,000]
Gastroschisis	2021	5	300804	59	19.61 (14.93-25.30) [/100,000]
Gastroschisis	2021	6	313498	66	21.05 (16.28-26.78) [/100,000]
Gastroschisis	2021	7	325951	63	19.33 (14.85-24.73) [/100,000]
Gastroschisis	2021	8	329954	51	15.46 (11.51-20.32) [/100,000]
Gastroschisis	2021	9	325613	52	15.97 (11.93-20.94) [/100,000]
Gastroschisis	2021	10	314971	60	19.05 (14.54-24.52) [/100,000]
Gastroschisis	2021	11	301665	45	14.92 (10.88-19.96) [/100,000]
Gastroschisis	2021	12	311074	66	21.22 (16.41-26.99) [/100,000]
Gastroschisis	2022	1	294903	58	19.67 (14.93-25.42) [/100,000]
Gastroschisis	2022	2	275665	59	21.40 (16.29-27.61) [/100,000]
Gastroschisis	2022	3	307473	44	14.31 (10.40-19.21) [/100,000]
Gastroschisis	2022	4	288688	50	17.32 (12.86-22.83) [/100,000]
Gastroschisis	2022	5	297684	49	16.46 (12.18-21.76) [/100,000]
Gastroschisis	2022	6	305797	55	17.99 (13.55-23.41) [/100,000]
Gastroschisis	2022	7	320845	43	13.40 (9.70-18.05) [/100,000]
Gastroschisis	2022	8	336052	43	12.80 (9.26-17.24) [/100,000]
Gastroschisis	2022	9	312405	48	15.36 (11.33-20.37) [/100,000]
Gastroschisis	2022	10	306926	53	17.27 (12.94-22.59) [/100,000]
Gastroschisis	2022	11	307231	46	14.97 (10.96-19.97) [/100,000]
Gastroschisis	2022	12	314859	38	12.07 (8.54-16.57) [/100,000]
Limb reduction defect	2016	1	317114	45	14.19 (10.35-18.99) [/100,000]
Limb reduction defect	2016	2	306225	45	14.70 (10.72-19.66) [/100,000]
Limb reduction defect	2016	3	328615	39	11.87 (8.44-16.22) [/100,000]
Limb reduction defect	2016	4	313767	45	14.34 (10.46-19.19) [/100,000]
Limb reduction defect	2016	5	327877	49	14.94 (11.06-19.76) [/100,000]
Limb reduction defect	2016	6	332521	40	12.03 (8.59-16.38) [/100,000]
Limb reduction defect	2016	7	343039	57	16.62 (12.59-21.53) [/100,000]
Limb reduction defect	2016	8	358174	61	17.03 (13.03-21.88) [/100,000]
Limb reduction defect	2016	9	346017	41	11.85 (8.50-16.07) [/100,000]
Limb reduction defect	2016	10	331330	44	13.28 (9.65-17.83) [/100,000]
Limb reduction defect	2016	11	319684	41	12.83 (9.20-17.40) [/100,000]
Limb reduction defect	2016	12	324921	42	12.93 (9.32-17.47) [/100,000]
Limb reduction defect	2017	1	314080	35	11.14 (7.76-15.50) [/100,000]
Limb reduction defect	2017	2	289301	40	13.83 (9.88-18.83) [/100,000]
Limb reduction defect	2017	3	319946	46	14.38 (10.53-19.18) [/100,000]
Limb reduction defect	2017	4	300459	43	14.31 (10.36-19.28) [/100,000]
Limb reduction defect	2017	5	322813	43	13.32 (9.64-17.94) [/100,000]
Limb reduction defect	2017	6	324241	58	17.89 (13.58-23.12) [/100,000]
Limb reduction defect	2017	7	335296	36	10.74 (7.52-14.86) [/100,000]
Limb reduction defect	2017	8	352660	41	11.63 (8.34-15.77) [/100,000]
Limb reduction defect	2017	9	337673	43	12.73 (9.22-17.15) [/100,000]
Limb reduction defect	2017	10	330310	45	13.62 (9.94-18.23) [/100,000]
Limb reduction defect	2017	11	316776	47	14.84 (10.90-19.73) [/100,000]
Limb reduction defect	2017	12	316262	49	15.49 (11.46-20.48) [/100,000]
Limb reduction defect	2018	1	315049	48	15.24 (11.23-20.20) [/100,000]
Limb reduction defect	2018	2	284508	34	11.95 (8.28-16.70) [/100,000]
Limb reduction defect	2018	3	316374	40	12.64 (9.03-17.22) [/100,000]
Limb reduction defect	2018	4	298702	42	14.06 (10.13-19.01) [/100,000]
Limb reduction defect	2018	5	320843	35	10.91 (7.60-15.17) [/100,000]
Limb reduction defect	2018	6	314959	45	14.29 (10.42-19.12) [/100,000]
Limb reduction defect	2018	7	329189	36	10.94 (7.66-15.14) [/100,000]
Limb reduction defect	2018	8	345135	48	13.91 (10.25-18.44) [/100,000]
Limb reduction defect	2018	9	323243	36	11.14 (7.80-15.42) [/100,000]
Limb reduction defect	2018	10	327299	49	14.97 (11.08-19.79) [/100,000]
Limb reduction defect	2018	11	309004	43	13.92 (10.07-18.74) [/100,000]
Limb reduction defect	2018	12	311122	31	9.96 (6.77-14.14) [/100,000]
Limb reduction defect	2019	1	311189	43	13.82 (10.00-18.61) [/100,000]
Limb reduction defect	2019	2	280254	57	20.34 (15.40-26.35) [/100,000]
Limb reduction defect	2019	3	304527	31	10.18 (6.92-14.45) [/100,000]
Limb reduction defect	2019	4	299272	24	8.02 (5.14-11.93) [/100,000]
Limb reduction defect	2019	5	316562	28	8.85 (5.88-12.78) [/100,000]
Limb reduction defect	2019	6	304319	36	11.83 (8.29-16.38) [/100,000]
Limb reduction defect	2019	7	334075	39	11.67 (8.30-15.96) [/100,000]
Limb reduction defect	2019	8	342126	32	9.35 (6.40-13.20) [/100,000]
Limb reduction defect	2019	9	326234	41	12.57 (9.02-17.05) [/100,000]
Limb reduction defect	2019	10	325771	34	10.44 (7.23-14.58) [/100,000]
Limb reduction defect	2019	11	298501	31	10.39 (7.06-14.74) [/100,000]
Limb reduction defect	2019	12	309284	45	14.55 (10.61-19.47) [/100,000]

Limb reduction defect	2020	1	305211	40	13.11 (9.36-17.85) [/100,000]
Limb reduction defect	2020	2	283058	46	16.25 (11.90-21.68) [/100,000]
Limb reduction defect	2020	3	302012	46	15.23 (11.15-20.32) [/100,000]
Limb reduction defect	2020	4	290578	29	9.98 (6.68-14.33) [/100,000]
Limb reduction defect	2020	5	301454	30	9.95 (6.71-14.21) [/100,000]
Limb reduction defect	2020	6	302144	35	11.58 (8.07-16.11) [/100,000]
Limb reduction defect	2020	7	321591	33	10.26 (7.06-14.41) [/100,000]
Limb reduction defect	2020	8	319610	44	13.77 (10.00-18.48) [/100,000]
Limb reduction defect	2020	9	311656	34	10.91 (7.56-15.24) [/100,000]
Limb reduction defect	2020	10	305015	40	13.11 (9.37-17.86) [/100,000]
Limb reduction defect	2020	11	282530	30	10.62 (7.16-15.16) [/100,000]
Limb reduction defect	2020	12	289802	33	11.39 (7.84-15.99) [/100,000]
Limb reduction defect	2021	1	277054	29	10.47 (7.01-15.03) [/100,000]
Limb reduction defect	2021	2	266235	23	8.64 (5.48-12.96) [/100,000]
Limb reduction defect	2021	3	302457	42	13.89 (10.01-18.77) [/100,000]
Limb reduction defect	2021	4	293104	34	11.60 (8.03-16.21) [/100,000]
Limb reduction defect	2021	5	300804	28	9.31 (6.19-13.45) [/100,000]
Limb reduction defect	2021	6	313498	48	15.31 (11.29-20.30) [/100,000]
Limb reduction defect	2021	7	325951	37	11.35 (7.99-15.65) [/100,000]
Limb reduction defect	2021	8	329954	40	12.12 (8.66-16.51) [/100,000]
Limb reduction defect	2021	9	325613	36	11.06 (7.74-15.31) [/100,000]
Limb reduction defect	2021	10	314971	37	11.75 (8.27-16.19) [/100,000]
Limb reduction defect	2021	11	301665	38	12.60 (8.91-17.29) [/100,000]
Limb reduction defect	2021	12	311074	35	11.25 (7.84-15.65) [/100,000]
Limb reduction defect	2022	1	294903	35	11.87 (8.27-16.51) [/100,000]
Limb reduction defect	2022	2	275665	34	12.33 (8.54-17.23) [/100,000]
Limb reduction defect	2022	3	307473	39	12.68 (9.02-17.34) [/100,000]
Limb reduction defect	2022	4	288688	31	10.74 (7.30-15.24) [/100,000]
Limb reduction defect	2022	5	297684	36	12.09 (8.47-16.74) [/100,000]
Limb reduction defect	2022	6	305797	31	10.14 (6.89-14.39) [/100,000]
Limb reduction defect	2022	7	320845	39	12.16 (8.64-16.62) [/100,000]
Limb reduction defect	2022	8	336052	39	11.61 (8.25-15.86) [/100,000]
Limb reduction defect	2022	9	312405	32	10.24 (7.01-14.46) [/100,000]
Limb reduction defect	2022	10	306926	36	11.73 (8.22-16.24) [/100,000]
Limb reduction defect	2022	11	307231	34	11.07 (7.66-15.46) [/100,000]
Limb reduction defect	2022	12	314859	38	12.07 (8.54-16.57) [/100,000]
Cleft Lip with or without Cleft Palate	2016	1	317114	172	54.24 (46.44-62.98) [/100,000]
Cleft Lip with or without Cleft Palate	2016	2	306225	178	58.13 (49.90-67.32) [/100,000]
Cleft Lip with or without Cleft Palate	2016	3	328615	165	50.21 (42.84-58.48) [/100,000]
Cleft Lip with or without Cleft Palate	2016	4	313767	178	56.73 (48.70-65.70) [/100,000]
Cleft Lip with or without Cleft Palate	2016	5	327877	180	54.90 (47.17-63.53) [/100,000]
Cleft Lip with or without Cleft Palate	2016	6	332521	205	61.65 (53.50-70.69) [/100,000]
Cleft Lip with or without Cleft Palate	2016	7	343039	182	53.06 (45.63-61.35) [/100,000]
Cleft Lip with or without Cleft Palate	2016	8	358174	190	53.05 (45.77-61.15) [/100,000]
Cleft Lip with or without Cleft Palate	2016	9	346017	170	49.13 (42.02-57.09) [/100,000]
Cleft Lip with or without Cleft Palate	2016	10	331330	159	47.99 (40.82-56.05) [/100,000]
Cleft Lip with or without Cleft Palate	2016	11	319684	168	52.55 (44.91-61.12) [/100,000]
Cleft Lip with or without Cleft Palate	2016	12	324921	164	50.47 (43.05-58.81) [/100,000]
Cleft Lip with or without Cleft Palate	2017	1	314080	163	51.90 (44.24-60.50) [/100,000]
Cleft Lip with or without Cleft Palate	2017	2	289301	154	53.23 (45.16-62.33) [/100,000]
Cleft Lip with or without Cleft Palate	2017	3	319946	182	56.88 (48.92-65.77) [/100,000]
Cleft Lip with or without Cleft Palate	2017	4	300459	147	48.93 (41.34-57.50) [/100,000]
Cleft Lip with or without Cleft Palate	2017	5	322813	188	58.24 (50.21-67.18) [/100,000]
Cleft Lip with or without Cleft Palate	2017	6	324241	164	50.58 (43.14-58.94) [/100,000]
Cleft Lip with or without Cleft Palate	2017	7	335296	194	57.86 (50.01-66.60) [/100,000]
Cleft Lip with or without Cleft Palate	2017	8	352660	170	48.21 (41.23-56.02) [/100,000]
Cleft Lip with or without Cleft Palate	2017	9	337673	166	49.16 (41.97-57.23) [/100,000]
Cleft Lip with or without Cleft Palate	2017	10	330310	171	51.77 (44.30-60.13) [/100,000]
Cleft Lip with or without Cleft Palate	2017	11	316776	133	41.99 (35.15-49.76) [/100,000]
Cleft Lip with or without Cleft Palate	2017	12	316262	161	50.91 (43.35-59.40) [/100,000]
Cleft Lip with or without Cleft Palate	2018	1	315049	183	58.09 (49.98-67.14) [/100,000]
Cleft Lip with or without Cleft Palate	2018	2	284508	142	49.91 (42.04-58.83) [/100,000]
Cleft Lip with or without Cleft Palate	2018	3	316374	200	63.22 (54.76-72.61) [/100,000]
Cleft Lip with or without Cleft Palate	2018	4	298702	173	57.92 (49.61-67.22) [/100,000]
Cleft Lip with or without Cleft Palate	2018	5	320843	180	56.10 (48.21-64.92) [/100,000]
Cleft Lip with or without Cleft Palate	2018	6	314959	154	48.90 (41.48-57.25) [/100,000]
Cleft Lip with or without Cleft Palate	2018	7	329189	179	54.38 (46.70-62.95) [/100,000]
Cleft Lip with or without Cleft Palate	2018	8	345135	183	53.02 (45.62-61.28) [/100,000]
Cleft Lip with or without Cleft Palate	2018	9	323243	158	48.88 (41.56-57.12) [/100,000]
Cleft Lip with or without Cleft Palate	2018	10	327299	181	55.30 (47.54-63.97) [/100,000]
Cleft Lip with or without Cleft Palate	2018	11	309004	144	46.60 (39.30-54.86) [/100,000]

Cleft Lip with or without Cleft Palate	2018	12	311122	177	56.89 (48.82-65.91) [/100,000]
Cleft Lip with or without Cleft Palate	2019	1	311189	159	51.09 (43.46-59.68) [/100,000]
Cleft Lip with or without Cleft Palate	2019	2	280254	152	54.24 (45.96-63.57) [/100,000]
Cleft Lip with or without Cleft Palate	2019	3	304527	151	49.59 (41.99-58.15) [/100,000]
Cleft Lip with or without Cleft Palate	2019	4	299272	147	49.12 (41.50-57.73) [/100,000]
Cleft Lip with or without Cleft Palate	2019	5	316562	152	48.02 (40.69-56.28) [/100,000]
Cleft Lip with or without Cleft Palate	2019	6	304319	163	53.56 (45.66-62.44) [/100,000]
Cleft Lip with or without Cleft Palate	2019	7	334075	147	44.00 (37.18-51.72) [/100,000]
Cleft Lip with or without Cleft Palate	2019	8	342126	174	50.86 (43.58-59.00) [/100,000]
Cleft Lip with or without Cleft Palate	2019	9	326234	161	49.35 (42.02-57.59) [/100,000]
Cleft Lip with or without Cleft Palate	2019	10	325771	142	43.59 (36.72-51.37) [/100,000]
Cleft Lip with or without Cleft Palate	2019	11	298501	141	47.24 (39.76-55.71) [/100,000]
Cleft Lip with or without Cleft Palate	2019	12	309284	151	48.82 (41.35-57.26) [/100,000]
Cleft Lip with or without Cleft Palate	2020	1	305211	183	59.96 (51.59-69.30) [/100,000]
Cleft Lip with or without Cleft Palate	2020	2	283058	139	49.11 (41.28-57.98) [/100,000]
Cleft Lip with or without Cleft Palate	2020	3	302012	130	43.04 (35.96-51.11) [/100,000]
Cleft Lip with or without Cleft Palate	2020	4	290578	120	41.30 (34.24-49.38) [/100,000]
Cleft Lip with or without Cleft Palate	2020	5	301454	155	51.42 (43.64-60.18) [/100,000]
Cleft Lip with or without Cleft Palate	2020	6	302144	160	52.95 (45.07-61.82) [/100,000]
Cleft Lip with or without Cleft Palate	2020	7	321591	153	47.58 (40.34-55.74) [/100,000]
Cleft Lip with or without Cleft Palate	2020	8	319610	152	47.56 (40.30-55.75) [/100,000]
Cleft Lip with or without Cleft Palate	2020	9	311656	154	49.41 (41.92-57.86) [/100,000]
Cleft Lip with or without Cleft Palate	2020	10	305015	167	54.75 (46.76-63.71) [/100,000]
Cleft Lip with or without Cleft Palate	2020	11	282530	125	44.24 (36.83-52.71) [/100,000]
Cleft Lip with or without Cleft Palate	2020	12	289802	154	53.14 (45.08-62.22) [/100,000]
Cleft Lip with or without Cleft Palate	2021	1	277054	152	54.86 (46.49-64.31) [/100,000]
Cleft Lip with or without Cleft Palate	2021	2	266235	143	53.71 (45.27-63.27) [/100,000]
Cleft Lip with or without Cleft Palate	2021	3	302457	187	61.83 (53.28-71.35) [/100,000]
Cleft Lip with or without Cleft Palate	2021	4	293104	161	54.93 (46.77-64.10) [/100,000]
Cleft Lip with or without Cleft Palate	2021	5	300804	151	50.20 (42.51-58.87) [/100,000]
Cleft Lip with or without Cleft Palate	2021	6	313498	145	46.25 (39.03-54.42) [/100,000]
Cleft Lip with or without Cleft Palate	2021	7	325951	182	55.84 (48.02-64.56) [/100,000]
Cleft Lip with or without Cleft Palate	2021	8	329954	174	52.73 (45.19-61.18) [/100,000]
Cleft Lip with or without Cleft Palate	2021	9	325613	158	48.52 (41.25-56.71) [/100,000]
Cleft Lip with or without Cleft Palate	2021	10	314971	160	50.80 (43.23-59.31) [/100,000]
Cleft Lip with or without Cleft Palate	2021	11	301665	142	47.07 (39.65-55.48) [/100,000]
Cleft Lip with or without Cleft Palate	2021	12	311074	162	52.08 (44.37-60.74) [/100,000]
Cleft Lip with or without Cleft Palate	2022	1	294903	137	46.46 (39.00-54.92) [/100,000]
Cleft Lip with or without Cleft Palate	2022	2	275665	142	51.51 (43.39-60.71) [/100,000]
Cleft Lip with or without Cleft Palate	2022	3	307473	157	51.06 (43.39-59.70) [/100,000]
Cleft Lip with or without Cleft Palate	2022	4	288688	146	50.57 (42.70-59.47) [/100,000]
Cleft Lip with or without Cleft Palate	2022	5	297684	150	50.39 (42.65-59.13) [/100,000]
Cleft Lip with or without Cleft Palate	2022	6	305797	155	50.69 (43.02-59.32) [/100,000]
Cleft Lip with or without Cleft Palate	2022	7	320845	162	50.49 (43.02-58.89) [/100,000]
Cleft Lip with or without Cleft Palate	2022	8	336052	179	53.27 (45.75-61.66) [/100,000]
Cleft Lip with or without Cleft Palate	2022	9	312405	168	53.78 (45.95-62.55) [/100,000]
Cleft Lip with or without Cleft Palate	2022	10	306926	139	45.29 (38.07-53.47) [/100,000]
Cleft Lip with or without Cleft Palate	2022	11	307231	157	51.10 (43.42-59.75) [/100,000]
Cleft Lip with or without Cleft Palate	2022	12	314859	170	53.99 (46.18-62.74) [/100,000]
Cleft Palate alone	2016	1	317114	75	23.65 (18.60-29.65) [/100,000]
Cleft Palate alone	2016	2	306225	70	22.86 (17.82-28.88) [/100,000]
Cleft Palate alone	2016	3	328615	70	21.30 (16.61-26.91) [/100,000]
Cleft Palate alone	2016	4	313767	74	23.58 (18.52-29.61) [/100,000]
Cleft Palate alone	2016	5	327877	83	25.31 (20.16-31.38) [/100,000]
Cleft Palate alone	2016	6	332521	74	22.25 (17.47-27.94) [/100,000]
Cleft Palate alone	2016	7	343039	82	23.90 (19.01-29.67) [/100,000]
Cleft Palate alone	2016	8	358174	83	23.17 (18.46-28.73) [/100,000]
Cleft Palate alone	2016	9	346017	71	20.52 (16.03-25.88) [/100,000]
Cleft Palate alone	2016	10	331330	75	22.64 (17.81-28.37) [/100,000]
Cleft Palate alone	2016	11	319684	75	23.46 (18.45-29.41) [/100,000]
Cleft Palate alone	2016	12	324921	83	25.54 (20.35-31.67) [/100,000]
Cleft Palate alone	2017	1	314080	62	19.74 (15.14-25.31) [/100,000]
Cleft Palate alone	2017	2	289301	75	25.92 (20.39-32.50) [/100,000]
Cleft Palate alone	2017	3	319946	76	23.75 (18.72-29.73) [/100,000]
Cleft Palate alone	2017	4	300459	89	29.62 (23.79-36.45) [/100,000]
Cleft Palate alone	2017	5	322813	63	19.52 (15.00-24.97) [/100,000]
Cleft Palate alone	2017	6	324241	60	18.50 (14.12-23.82) [/100,000]
Cleft Palate alone	2017	7	335296	86	25.65 (20.52-31.68) [/100,000]
Cleft Palate alone	2017	8	352660	90	25.52 (20.52-31.37) [/100,000]
Cleft Palate alone	2017	9	337673	98	29.02 (23.56-35.37) [/100,000]
Cleft Palate alone	2017	10	330310	74	22.40 (17.59-28.12) [/100,000]

Cleft Palate alone	2017	11	316776	75	23.68 (18.62-29.68) [/100,000]
Cleft Palate alone	2017	12	316262	80	25.30 (20.06-31.48) [/100,000]
Cleft Palate alone	2018	1	315049	77	24.44 (19.29-30.55) [/100,000]
Cleft Palate alone	2018	2	284508	81	28.47 (22.61-35.38) [/100,000]
Cleft Palate alone	2018	3	316374	86	27.18 (21.74-33.57) [/100,000]
Cleft Palate alone	2018	4	298702	56	18.75 (14.16-24.34) [/100,000]
Cleft Palate alone	2018	5	320843	75	23.38 (18.39-29.30) [/100,000]
Cleft Palate alone	2018	6	314959	74	23.50 (18.45-29.50) [/100,000]
Cleft Palate alone	2018	7	329189	79	24.00 (19.00-29.91) [/100,000]
Cleft Palate alone	2018	8	345135	88	25.50 (20.45-31.41) [/100,000]
Cleft Palate alone	2018	9	323243	73	22.58 (17.70-28.39) [/100,000]
Cleft Palate alone	2018	10	327299	77	23.53 (18.57-29.40) [/100,000]
Cleft Palate alone	2018	11	309004	68	22.01 (17.09-27.90) [/100,000]
Cleft Palate alone	2018	12	311122	86	27.64 (22.11-34.14) [/100,000]
Cleft Palate alone	2019	1	311189	71	22.82 (17.82-28.78) [/100,000]
Cleft Palate alone	2019	2	280254	66	23.55 (18.21-29.96) [/100,000]
Cleft Palate alone	2019	3	304527	78	25.61 (20.25-31.97) [/100,000]
Cleft Palate alone	2019	4	299272	67	22.39 (17.35-28.43) [/100,000]
Cleft Palate alone	2019	5	316562	75	23.69 (18.64-29.70) [/100,000]
Cleft Palate alone	2019	6	304319	67	22.02 (17.06-27.96) [/100,000]
Cleft Palate alone	2019	7	334075	72	21.55 (16.86-27.14) [/100,000]
Cleft Palate alone	2019	8	342126	77	22.51 (17.76-28.13) [/100,000]
Cleft Palate alone	2019	9	326234	68	20.84 (16.19-26.42) [/100,000]
Cleft Palate alone	2019	10	325771	70	21.49 (16.75-27.15) [/100,000]
Cleft Palate alone	2019	11	298501	73	24.46 (19.17-30.75) [/100,000]
Cleft Palate alone	2019	12	309284	81	26.19 (20.80-32.55) [/100,000]
Cleft Palate alone	2020	1	305211	75	24.57 (19.33-30.80) [/100,000]
Cleft Palate alone	2020	2	283058	59	20.84 (15.87-26.89) [/100,000]
Cleft Palate alone	2020	3	302012	75	24.83 (19.53-31.13) [/100,000]
Cleft Palate alone	2020	4	290578	71	24.43 (19.08-30.82) [/100,000]
Cleft Palate alone	2020	5	301454	74	24.55 (19.28-30.82) [/100,000]
Cleft Palate alone	2020	6	302144	91	30.12 (24.25-36.98) [/100,000]
Cleft Palate alone	2020	7	321591	74	23.01 (18.07-28.89) [/100,000]
Cleft Palate alone	2020	8	319610	65	20.34 (15.70-25.92) [/100,000]
Cleft Palate alone	2020	9	311656	78	25.03 (19.78-31.23) [/100,000]
Cleft Palate alone	2020	10	305015	69	22.62 (17.60-28.63) [/100,000]
Cleft Palate alone	2020	11	282530	63	22.30 (17.14-28.53) [/100,000]
Cleft Palate alone	2020	12	289802	52	17.94 (13.40-23.53) [/100,000]
Cleft Palate alone	2021	1	277054	55	19.85 (14.96-25.84) [/100,000]
Cleft Palate alone	2021	2	266235	69	25.92 (20.17-32.80) [/100,000]
Cleft Palate alone	2021	3	302457	78	25.79 (20.39-32.18) [/100,000]
Cleft Palate alone	2021	4	293104	74	25.25 (19.82-31.69) [/100,000]
Cleft Palate alone	2021	5	300804	64	21.28 (16.39-27.17) [/100,000]
Cleft Palate alone	2021	6	313498	72	22.97 (17.97-28.92) [/100,000]
Cleft Palate alone	2021	7	325951	77	23.62 (18.64-29.52) [/100,000]
Cleft Palate alone	2021	8	329954	73	22.12 (17.34-27.82) [/100,000]
Cleft Palate alone	2021	9	325613	60	18.43 (14.06-23.72) [/100,000]
Cleft Palate alone	2021	10	314971	70	22.22 (17.33-28.08) [/100,000]
Cleft Palate alone	2021	11	301665	86	28.51 (22.80-35.21) [/100,000]
Cleft Palate alone	2021	12	311074	69	22.18 (17.26-28.07) [/100,000]
Cleft Palate alone	2022	1	294903	60	20.35 (15.53-26.19) [/100,000]
Cleft Palate alone	2022	2	275665	61	22.13 (16.93-28.42) [/100,000]
Cleft Palate alone	2022	3	307473	64	20.81 (16.03-26.58) [/100,000]
Cleft Palate alone	2022	4	288688	65	22.52 (17.38-28.70) [/100,000]
Cleft Palate alone	2022	5	297684	59	19.82 (15.09-25.57) [/100,000]
Cleft Palate alone	2022	6	305797	66	21.58 (16.69-27.46) [/100,000]
Cleft Palate alone	2022	7	320845	86	26.80 (21.44-33.10) [/100,000]
Cleft Palate alone	2022	8	336052	72	21.43 (16.76-26.98) [/100,000]
Cleft Palate alone	2022	9	312405	77	24.65 (19.45-30.80) [/100,000]
Cleft Palate alone	2022	10	306926	74	24.11 (18.93-30.27) [/100,000]
Cleft Palate alone	2022	11	307231	58	18.88 (14.34-24.40) [/100,000]
Cleft Palate alone	2022	12	314859	79	25.09 (19.86-31.27) [/100,000]
Down syndrome	2016	1	317114	176	55.50 (47.61-64.33) [/100,000]
Down syndrome	2016	2	306225	169	55.19 (47.18-64.16) [/100,000]
Down syndrome	2016	3	328615	176	53.56 (45.94-62.08) [/100,000]
Down syndrome	2016	4	313767	174	55.46 (47.52-64.33) [/100,000]
Down syndrome	2016	5	327877	176	53.68 (46.04-62.22) [/100,000]
Down syndrome	2016	6	332521	172	51.73 (44.29-60.06) [/100,000]
Down syndrome	2016	7	343039	171	49.85 (42.66-57.90) [/100,000]
Down syndrome	2016	8	358174	208	58.07 (50.45-66.52) [/100,000]
Down syndrome	2016	9	346017	196	56.64 (48.99-65.15) [/100,000]

Down syndrome	2016	10	331330	179	54.02 (46.40-62.54) [/100,000]
Down syndrome	2016	11	319684	189	59.12 (50.99-68.17) [/100,000]
Down syndrome	2016	12	324921	182	56.01 (48.17-64.77) [/100,000]
Down syndrome	2017	1	314080	165	52.53 (44.83-61.19) [/100,000]
Down syndrome	2017	2	289301	147	50.81 (42.93-59.72) [/100,000]
Down syndrome	2017	3	319946	165	51.57 (44.00-60.07) [/100,000]
Down syndrome	2017	4	300459	149	49.59 (41.95-58.22) [/100,000]
Down syndrome	2017	5	322813	174	53.90 (46.19-62.53) [/100,000]
Down syndrome	2017	6	324241	197	60.76 (52.57-69.86) [/100,000]
Down syndrome	2017	7	335296	169	50.40 (43.09-58.60) [/100,000]
Down syndrome	2017	8	352660	198	56.14 (48.60-64.53) [/100,000]
Down syndrome	2017	9	337673	175	51.83 (44.43-60.10) [/100,000]
Down syndrome	2017	10	330310	164	49.65 (42.34-57.86) [/100,000]
Down syndrome	2017	11	316776	167	52.72 (45.03-61.35) [/100,000]
Down syndrome	2017	12	316262	174	55.02 (47.15-63.82) [/100,000]
Down syndrome	2018	1	315049	179	56.82 (48.80-65.77) [/100,000]
Down syndrome	2018	2	284508	190	66.78 (57.63-76.98) [/100,000]
Down syndrome	2018	3	316374	169	53.42 (45.67-62.10) [/100,000]
Down syndrome	2018	4	298702	166	55.57 (47.44-64.70) [/100,000]
Down syndrome	2018	5	320843	162	50.49 (43.02-58.89) [/100,000]
Down syndrome	2018	6	314959	174	55.25 (47.34-64.09) [/100,000]
Down syndrome	2018	7	329189	169	51.34 (43.89-59.69) [/100,000]
Down syndrome	2018	8	345135	181	52.44 (45.08-60.66) [/100,000]
Down syndrome	2018	9	323243	186	57.54 (49.57-66.43) [/100,000]
Down syndrome	2018	10	327299	213	65.08 (56.63-74.43) [/100,000]
Down syndrome	2018	11	309004	170	55.02 (47.06-63.93) [/100,000]
Down syndrome	2018	12	311122	149	47.89 (40.51-56.23) [/100,000]
Down syndrome	2019	1	311189	164	52.70 (44.95-61.41) [/100,000]
Down syndrome	2019	2	280254	165	58.88 (50.24-68.57) [/100,000]
Down syndrome	2019	3	304527	179	58.78 (50.49-68.05) [/100,000]
Down syndrome	2019	4	299272	149	49.79 (42.12-58.45) [/100,000]
Down syndrome	2019	5	316562	164	51.81 (44.18-60.37) [/100,000]
Down syndrome	2019	6	304319	147	48.30 (40.81-56.77) [/100,000]
Down syndrome	2019	7	334075	163	48.79 (41.59-56.88) [/100,000]
Down syndrome	2019	8	342126	182	53.20 (45.75-61.51) [/100,000]
Down syndrome	2019	9	326234	189	57.93 (49.97-66.81) [/100,000]
Down syndrome	2019	10	325771	175	53.72 (46.06-62.29) [/100,000]
Down syndrome	2019	11	298501	187	62.65 (53.99-72.29) [/100,000]
Down syndrome	2019	12	309284	166	53.67 (45.82-62.48) [/100,000]
Down syndrome	2020	1	305211	172	56.35 (48.25-65.43) [/100,000]
Down syndrome	2020	2	283058	166	58.65 (50.07-68.27) [/100,000]
Down syndrome	2020	3	302012	150	49.67 (42.04-58.28) [/100,000]
Down syndrome	2020	4	290578	152	52.31 (44.33-61.32) [/100,000]
Down syndrome	2020	5	301454	162	53.74 (45.78-62.68) [/100,000]
Down syndrome	2020	6	302144	161	53.29 (45.37-62.18) [/100,000]
Down syndrome	2020	7	321591	165	51.31 (43.78-59.76) [/100,000]
Down syndrome	2020	8	319610	179	56.01 (48.10-64.84) [/100,000]
Down syndrome	2020	9	311656	172	55.19 (47.25-64.08) [/100,000]
Down syndrome	2020	10	305015	151	49.51 (41.93-58.06) [/100,000]
Down syndrome	2020	11	282530	182	64.42 (55.40-74.48) [/100,000]
Down syndrome	2020	12	289802	142	49.00 (41.27-57.75) [/100,000]
Down syndrome	2021	1	277054	161	58.11 (49.48-67.81) [/100,000]
Down syndrome	2021	2	266235	153	57.47 (48.72-67.33) [/100,000]
Down syndrome	2021	3	302457	149	49.26 (41.67-57.84) [/100,000]
Down syndrome	2021	4	293104	150	51.18 (43.32-60.05) [/100,000]
Down syndrome	2021	5	300804	151	50.20 (42.51-58.87) [/100,000]
Down syndrome	2021	6	313498	168	53.59 (45.79-62.33) [/100,000]
Down syndrome	2021	7	325951	154	47.25 (40.08-55.32) [/100,000]
Down syndrome	2021	8	329954	167	50.61 (43.23-58.90) [/100,000]
Down syndrome	2021	9	325613	174	53.44 (45.79-61.99) [/100,000]
Down syndrome	2021	10	314971	170	53.97 (46.17-62.72) [/100,000]
Down syndrome	2021	11	301665	151	50.06 (42.39-58.70) [/100,000]
Down syndrome	2021	12	311074	155	49.83 (42.29-58.32) [/100,000]
Down syndrome	2022	1	294903	162	54.93 (46.80-64.07) [/100,000]
Down syndrome	2022	2	275665	157	56.95 (48.39-66.59) [/100,000]
Down syndrome	2022	3	307473	162	52.69 (44.89-61.45) [/100,000]
Down syndrome	2022	4	288688	124	42.95 (35.73-51.21) [/100,000]
Down syndrome	2022	5	297684	154	51.73 (43.89-60.58) [/100,000]
Down syndrome	2022	6	305797	155	50.69 (43.02-59.32) [/100,000]
Down syndrome	2022	7	320845	197	61.40 (53.13-70.60) [/100,000]
Down syndrome	2022	8	336052	169	50.29 (42.99-58.47) [/100,000]

Down syndrome	2022	9	312405	149	47.69 (40.35-55.99) [/100,000]
Down syndrome	2022	10	306926	146	47.57 (40.17-55.94) [/100,000]
Down syndrome	2022	11	307231	136	44.27 (37.14-52.36) [/100,000]
Down syndrome	2022	12	314859	150	47.64 (40.32-55.90) [/100,000]
Suspected chromosomal disorder	2016	1	317114	132	41.63 (34.83-49.36) [/100,000]
Suspected chromosomal disorder	2016	2	306225	137	44.74 (37.56-52.89) [/100,000]
Suspected chromosomal disorder	2016	3	328615	164	49.91 (42.56-58.15) [/100,000]
Suspected chromosomal disorder	2016	4	313767	146	46.53 (39.29-54.72) [/100,000]
Suspected chromosomal disorder	2016	5	327877	131	39.95 (33.41-47.41) [/100,000]
Suspected chromosomal disorder	2016	6	332521	141	42.40 (35.69-50.01) [/100,000]
Suspected chromosomal disorder	2016	7	343039	166	48.39 (41.31-56.34) [/100,000]
Suspected chromosomal disorder	2016	8	358174	137	38.25 (32.11-45.22) [/100,000]
Suspected chromosomal disorder	2016	9	346017	142	41.04 (34.57-48.37) [/100,000]
Suspected chromosomal disorder	2016	10	331330	151	45.57 (38.60-53.45) [/100,000]
Suspected chromosomal disorder	2016	11	319684	131	40.98 (34.26-48.62) [/100,000]
Suspected chromosomal disorder	2016	12	324921	122	37.55 (31.18-44.83) [/100,000]
Suspected chromosomal disorder	2017	1	314080	127	40.44 (33.71-48.11) [/100,000]
Suspected chromosomal disorder	2017	2	289301	127	43.90 (36.60-52.23) [/100,000]
Suspected chromosomal disorder	2017	3	319946	140	43.76 (36.81-51.63) [/100,000]
Suspected chromosomal disorder	2017	4	300459	140	46.60 (39.20-54.98) [/100,000]
Suspected chromosomal disorder	2017	5	322813	142	43.99 (37.05-51.85) [/100,000]
Suspected chromosomal disorder	2017	6	324241	146	45.03 (38.02-52.95) [/100,000]
Suspected chromosomal disorder	2017	7	335296	143	42.65 (35.95-50.24) [/100,000]
Suspected chromosomal disorder	2017	8	352660	128	36.30 (30.28-43.15) [/100,000]
Suspected chromosomal disorder	2017	9	337673	155	45.90 (38.96-53.72) [/100,000]
Suspected chromosomal disorder	2017	10	330310	158	47.83 (40.67-55.90) [/100,000]
Suspected chromosomal disorder	2017	11	316776	121	38.20 (31.70-45.64) [/100,000]
Suspected chromosomal disorder	2017	12	316262	131	41.42 (34.63-49.15) [/100,000]
Suspected chromosomal disorder	2018	1	315049	157	49.83 (42.34-58.26) [/100,000]
Suspected chromosomal disorder	2018	2	284508	140	49.21 (41.40-58.06) [/100,000]
Suspected chromosomal disorder	2018	3	316374	139	43.94 (36.94-51.87) [/100,000]
Suspected chromosomal disorder	2018	4	298702	136	45.53 (38.20-53.86) [/100,000]
Suspected chromosomal disorder	2018	5	320843	141	43.95 (36.99-51.83) [/100,000]
Suspected chromosomal disorder	2018	6	314959	146	46.36 (39.14-54.51) [/100,000]
Suspected chromosomal disorder	2018	7	329189	147	44.66 (37.73-52.48) [/100,000]
Suspected chromosomal disorder	2018	8	345135	156	45.20 (38.39-52.87) [/100,000]
Suspected chromosomal disorder	2018	9	323243	144	44.55 (37.57-52.45) [/100,000]
Suspected chromosomal disorder	2018	10	327299	178	54.38 (46.69-62.98) [/100,000]
Suspected chromosomal disorder	2018	11	309004	128	41.42 (34.56-49.25) [/100,000]
Suspected chromosomal disorder	2018	12	311122	114	36.64 (30.23-44.02) [/100,000]
Suspected chromosomal disorder	2019	1	311189	157	50.45 (42.87-58.99) [/100,000]
Suspected chromosomal disorder	2019	2	280254	153	54.59 (46.29-63.96) [/100,000]
Suspected chromosomal disorder	2019	3	304527	125	41.05 (34.17-48.90) [/100,000]
Suspected chromosomal disorder	2019	4	299272	139	46.45 (39.05-54.84) [/100,000]
Suspected chromosomal disorder	2019	5	316562	137	43.28 (36.34-51.16) [/100,000]
Suspected chromosomal disorder	2019	6	304319	140	46.00 (38.70-54.28) [/100,000]
Suspected chromosomal disorder	2019	7	334075	142	42.51 (35.80-50.10) [/100,000]
Suspected chromosomal disorder	2019	8	342126	135	39.46 (33.08-46.70) [/100,000]
Suspected chromosomal disorder	2019	9	326234	148	45.37 (38.35-53.29) [/100,000]
Suspected chromosomal disorder	2019	10	325771	128	39.29 (32.78-46.72) [/100,000]
Suspected chromosomal disorder	2019	11	298501	119	39.87 (33.03-47.70) [/100,000]
Suspected chromosomal disorder	2019	12	309284	144	46.56 (39.27-54.81) [/100,000]
Suspected chromosomal disorder	2020	1	305211	150	49.15 (41.60-57.67) [/100,000]
Suspected chromosomal disorder	2020	2	283058	140	49.46 (41.61-58.36) [/100,000]
Suspected chromosomal disorder	2020	3	302012	131	43.38 (36.27-51.47) [/100,000]
Suspected chromosomal disorder	2020	4	290578	109	37.51 (30.80-45.25) [/100,000]
Suspected chromosomal disorder	2020	5	301454	134	44.45 (37.25-52.64) [/100,000]
Suspected chromosomal disorder	2020	6	302144	153	50.64 (42.93-59.33) [/100,000]
Suspected chromosomal disorder	2020	7	321591	142	44.16 (37.19-52.04) [/100,000]
Suspected chromosomal disorder	2020	8	319610	145	45.37 (38.29-53.38) [/100,000]
Suspected chromosomal disorder	2020	9	311656	143	45.88 (38.67-54.05) [/100,000]
Suspected chromosomal disorder	2020	10	305015	142	46.56 (39.21-54.87) [/100,000]
Suspected chromosomal disorder	2020	11	282530	124	43.89 (36.51-52.33) [/100,000]
Suspected chromosomal disorder	2020	12	289802	139	47.96 (40.32-56.63) [/100,000]
Suspected chromosomal disorder	2021	1	277054	119	42.95 (35.58-51.40) [/100,000]
Suspected chromosomal disorder	2021	2	266235	144	54.09 (45.62-63.68) [/100,000]
Suspected chromosomal disorder	2021	3	302457	152	50.26 (42.58-58.91) [/100,000]
Suspected chromosomal disorder	2021	4	293104	148	50.49 (42.69-59.31) [/100,000]
Suspected chromosomal disorder	2021	5	300804	143	47.54 (40.07-56.00) [/100,000]
Suspected chromosomal disorder	2021	6	313498	148	47.21 (39.91-55.45) [/100,000]
Suspected chromosomal disorder	2021	7	325951	169	51.85 (44.33-60.28) [/100,000]

Suspected chromosomal disorder	2021	8	329954	152	46.07 (39.04-54.00) [/100,000]
Suspected chromosomal disorder	2021	9	325613	134	41.15 (34.48-48.74) [/100,000]
Suspected chromosomal disorder	2021	10	314971	148	46.99 (39.72-55.20) [/100,000]
Suspected chromosomal disorder	2021	11	301665	127	42.10 (35.10-50.09) [/100,000]
Suspected chromosomal disorder	2021	12	311074	147	47.26 (39.93-55.54) [/100,000]
Suspected chromosomal disorder	2022	1	294903	129	43.74 (36.52-51.97) [/100,000]
Suspected chromosomal disorder	2022	2	275665	135	48.97 (41.06-57.96) [/100,000]
Suspected chromosomal disorder	2022	3	307473	132	42.93 (35.92-50.91) [/100,000]
Suspected chromosomal disorder	2022	4	288688	122	42.26 (35.10-50.46) [/100,000]
Suspected chromosomal disorder	2022	5	297684	113	37.96 (31.29-45.64) [/100,000]
Suspected chromosomal disorder	2022	6	305797	149	48.73 (41.22-57.20) [/100,000]
Suspected chromosomal disorder	2022	7	320845	134	41.76 (34.99-49.46) [/100,000]
Suspected chromosomal disorder	2022	8	336052	133	39.58 (33.14-46.90) [/100,000]
Suspected chromosomal disorder	2022	9	312405	135	43.21 (36.23-51.15) [/100,000]
Suspected chromosomal disorder	2022	10	306926	148	48.22 (40.77-56.64) [/100,000]
Suspected chromosomal disorder	2022	11	307231	139	45.24 (38.04-53.42) [/100,000]
Suspected chromosomal disorder	2022	12	314859	126	40.02 (33.34-47.64) [/100,000]
Hypospadias	2016	1	317114	198	62.44 (54.05-71.76) [/100,000]
Hypospadias	2016	2	306225	205	66.94 (58.10-76.76) [/100,000]
Hypospadias	2016	3	328615	231	70.30 (61.52-79.96) [/100,000]
Hypospadias	2016	4	313767	198	63.10 (54.62-72.53) [/100,000]
Hypospadias	2016	5	327877	220	67.10 (58.53-76.57) [/100,000]
Hypospadias	2016	6	332521	187	56.24 (48.47-64.90) [/100,000]
Hypospadias	2016	7	343039	192	55.97 (48.33-64.47) [/100,000]
Hypospadias	2016	8	358174	225	62.82 (54.88-71.58) [/100,000]
Hypospadias	2016	9	346017	208	60.11 (52.22-68.86) [/100,000]
Hypospadias	2016	10	331330	199	60.06 (52.01-69.01) [/100,000]
Hypospadias	2016	11	319684	206	64.44 (55.94-73.86) [/100,000]
Hypospadias	2016	12	324921	187	57.55 (49.60-66.42) [/100,000]
Hypospadias	2017	1	314080	194	61.77 (53.38-71.09) [/100,000]
Hypospadias	2017	2	289301	187	64.64 (55.71-74.59) [/100,000]
Hypospadias	2017	3	319946	167	52.20 (44.58-60.74) [/100,000]
Hypospadias	2017	4	300459	193	64.24 (55.49-73.96) [/100,000]
Hypospadias	2017	5	322813	207	64.12 (55.69-73.48) [/100,000]
Hypospadias	2017	6	324241	199	61.37 (53.14-70.52) [/100,000]
Hypospadias	2017	7	335296	199	59.35 (51.39-68.19) [/100,000]
Hypospadias	2017	8	352660	238	67.49 (59.19-76.62) [/100,000]
Hypospadias	2017	9	337673	180	53.31 (45.80-61.69) [/100,000]
Hypospadias	2017	10	330310	183	55.40 (47.67-64.03) [/100,000]
Hypospadias	2017	11	316776	182	57.45 (49.41-66.43) [/100,000]
Hypospadias	2017	12	316262	188	59.44 (51.25-68.57) [/100,000]
Hypospadias	2018	1	315049	174	55.23 (47.33-64.07) [/100,000]
Hypospadias	2018	2	284508	180	63.27 (54.36-73.21) [/100,000]
Hypospadias	2018	3	316374	180	56.89 (48.89-65.84) [/100,000]
Hypospadias	2018	4	298702	193	64.61 (55.82-74.40) [/100,000]
Hypospadias	2018	5	320843	197	61.40 (53.13-70.60) [/100,000]
Hypospadias	2018	6	314959	182	57.79 (49.70-66.81) [/100,000]
Hypospadias	2018	7	329189	193	58.63 (50.65-67.51) [/100,000]
Hypospadias	2018	8	345135	194	56.21 (48.58-64.70) [/100,000]
Hypospadias	2018	9	323243	196	60.64 (52.45-69.74) [/100,000]
Hypospadias	2018	10	327299	220	67.22 (58.63-76.71) [/100,000]
Hypospadias	2018	11	309004	173	55.99 (47.96-64.98) [/100,000]
Hypospadias	2018	12	311122	156	50.14 (42.58-58.65) [/100,000]
Hypospadias	2019	1	311189	170	54.63 (46.73-63.48) [/100,000]
Hypospadias	2019	2	280254	161	57.45 (48.92-67.04) [/100,000]
Hypospadias	2019	3	304527	181	59.44 (51.09-68.75) [/100,000]
Hypospadias	2019	4	299272	156	52.13 (44.27-60.98) [/100,000]
Hypospadias	2019	5	316562	175	55.28 (47.40-64.10) [/100,000]
Hypospadias	2019	6	304319	188	61.78 (53.26-71.26) [/100,000]
Hypospadias	2019	7	334075	170	50.89 (43.53-59.14) [/100,000]
Hypospadias	2019	8	342126	185	54.07 (46.56-62.45) [/100,000]
Hypospadias	2019	9	326234	188	57.63 (49.69-66.48) [/100,000]
Hypospadias	2019	10	325771	170	52.18 (44.64-60.64) [/100,000]
Hypospadias	2019	11	298501	177	59.30 (50.88-68.70) [/100,000]
Hypospadias	2019	12	309284	149	48.18 (40.75-56.56) [/100,000]
Hypospadias	2020	1	305211	183	59.96 (51.59-69.30) [/100,000]
Hypospadias	2020	2	283058	168	59.35 (50.72-69.03) [/100,000]
Hypospadias	2020	3	302012	137	45.36 (38.09-53.62) [/100,000]
Hypospadias	2020	4	290578	173	59.54 (51.00-69.10) [/100,000]
Hypospadias	2020	5	301454	153	50.75 (43.03-59.46) [/100,000]
Hypospadias	2020	6	302144	199	65.86 (57.03-75.67) [/100,000]

Hypospadias	2020	7	321591	179	55.66 (47.81-64.44) [/100,000]
Hypospadias	2020	8	319610	194	60.70 (52.46-69.86) [/100,000]
Hypospadias	2020	9	311656	175	56.15 (48.14-65.11) [/100,000]
Hypospadias	2020	10	305015	150	49.18 (41.62-57.71) [/100,000]
Hypospadias	2020	11	282530	155	54.86 (46.57-64.21) [/100,000]
Hypospadias	2020	12	289802	142	49.00 (41.27-57.75) [/100,000]
Hypospadias	2021	1	277054	154	55.58 (47.15-65.09) [/100,000]
Hypospadias	2021	2	266235	160	60.10 (51.15-70.16) [/100,000]
Hypospadias	2021	3	302457	156	51.58 (43.80-60.33) [/100,000]
Hypospadias	2021	4	293104	162	55.27 (47.09-64.46) [/100,000]
Hypospadias	2021	5	300804	184	61.17 (52.65-70.67) [/100,000]
Hypospadias	2021	6	313498	145	46.25 (39.03-54.42) [/100,000]
Hypospadias	2021	7	325951	196	60.13 (52.01-69.16) [/100,000]
Hypospadias	2021	8	329954	180	54.55 (46.88-63.13) [/100,000]
Hypospadias	2021	9	325613	156	47.91 (40.69-56.04) [/100,000]
Hypospadias	2021	10	314971	170	53.97 (46.17-62.72) [/100,000]
Hypospadias	2021	11	301665	148	49.06 (41.48-57.63) [/100,000]
Hypospadias	2021	12	311074	145	46.61 (39.34-54.84) [/100,000]
Hypospadias	2022	1	294903	148	50.19 (42.43-58.95) [/100,000]
Hypospadias	2022	2	275665	153	55.50 (47.06-65.02) [/100,000]
Hypospadias	2022	3	307473	172	55.94 (47.89-64.95) [/100,000]
Hypospadias	2022	4	288688	152	52.65 (44.62-61.72) [/100,000]
Hypospadias	2022	5	297684	160	53.75 (45.74-62.75) [/100,000]
Hypospadias	2022	6	305797	162	52.98 (45.13-61.79) [/100,000]
Hypospadias	2022	7	320845	185	57.66 (49.65-66.59) [/100,000]
Hypospadias	2022	8	336052	193	57.43 (49.62-66.13) [/100,000]
Hypospadias	2022	9	312405	137	43.85 (36.82-51.84) [/100,000]
Hypospadias	2022	10	306926	170	55.39 (47.38-64.37) [/100,000]
Hypospadias	2022	11	307231	133	43.29 (36.25-51.30) [/100,000]
Hypospadias	2022	12	314859	145	46.05 (38.86-54.19) [/100,000]
Transferred within 24 hours of delivery	2016	1	317179	3803	1.20 (1.16-1.24) [/100]
Transferred within 24 hours of delivery	2016	2	306559	3537	1.15 (1.12-1.19) [/100]
Transferred within 24 hours of delivery	2016	3	329135	3739	1.14 (1.10-1.17) [/100]
Transferred within 24 hours of delivery	2016	4	314067	3598	1.15 (1.11-1.18) [/100]
Transferred within 24 hours of delivery	2016	5	328201	3685	1.12 (1.09-1.16) [/100]
Transferred within 24 hours of delivery	2016	6	332946	3732	1.12 (1.09-1.16) [/100]
Transferred within 24 hours of delivery	2016	7	343396	4034	1.17 (1.14-1.21) [/100]
Transferred within 24 hours of delivery	2016	8	358476	3945	1.10 (1.07-1.14) [/100]
Transferred within 24 hours of delivery	2016	9	346260	3728	1.08 (1.04-1.11) [/100]
Transferred within 24 hours of delivery	2016	10	331689	3672	1.11 (1.07-1.14) [/100]
Transferred within 24 hours of delivery	2016	11	320029	3439	1.07 (1.04-1.11) [/100]
Transferred within 24 hours of delivery	2016	12	325307	3690	1.13 (1.10-1.17) [/100]
Transferred within 24 hours of delivery	2017	1	314409	3644	1.16 (1.12-1.20) [/100]
Transferred within 24 hours of delivery	2017	2	289484	3336	1.15 (1.11-1.19) [/100]
Transferred within 24 hours of delivery	2017	3	320086	3642	1.14 (1.10-1.18) [/100]
Transferred within 24 hours of delivery	2017	4	300594	3405	1.13 (1.10-1.17) [/100]
Transferred within 24 hours of delivery	2017	5	322959	3679	1.14 (1.10-1.18) [/100]
Transferred within 24 hours of delivery	2017	6	324432	3811	1.17 (1.14-1.21) [/100]
Transferred within 24 hours of delivery	2017	7	335508	3955	1.18 (1.14-1.22) [/100]
Transferred within 24 hours of delivery	2017	8	352840	4109	1.16 (1.13-1.20) [/100]
Transferred within 24 hours of delivery	2017	9	337831	3786	1.12 (1.09-1.16) [/100]
Transferred within 24 hours of delivery	2017	10	330565	3791	1.15 (1.11-1.18) [/100]
Transferred within 24 hours of delivery	2017	11	317065	3665	1.16 (1.12-1.19) [/100]
Transferred within 24 hours of delivery	2017	12	316494	3679	1.16 (1.13-1.20) [/100]
Transferred within 24 hours of delivery	2018	1	315419	3722	1.18 (1.14-1.22) [/100]
Transferred within 24 hours of delivery	2018	2	284769	3292	1.16 (1.12-1.20) [/100]
Transferred within 24 hours of delivery	2018	3	316646	3656	1.15 (1.12-1.19) [/100]
Transferred within 24 hours of delivery	2018	4	298979	3522	1.18 (1.14-1.22) [/100]
Transferred within 24 hours of delivery	2018	5	321306	3808	1.19 (1.15-1.22) [/100]
Transferred within 24 hours of delivery	2018	6	315405	3702	1.17 (1.14-1.21) [/100]
Transferred within 24 hours of delivery	2018	7	329668	3917	1.19 (1.15-1.23) [/100]
Transferred within 24 hours of delivery	2018	8	345522	3979	1.15 (1.12-1.19) [/100]
Transferred within 24 hours of delivery	2018	9	323471	3891	1.20 (1.17-1.24) [/100]
Transferred within 24 hours of delivery	2018	10	327503	3872	1.18 (1.15-1.22) [/100]
Transferred within 24 hours of delivery	2018	11	309244	3509	1.13 (1.10-1.17) [/100]
Transferred within 24 hours of delivery	2018	12	311355	3653	1.17 (1.14-1.21) [/100]
Transferred within 24 hours of delivery	2019	1	311436	3591	1.15 (1.12-1.19) [/100]
Transferred within 24 hours of delivery	2019	2	280403	3342	1.19 (1.15-1.23) [/100]
Transferred within 24 hours of delivery	2019	3	304628	3605	1.18 (1.15-1.22) [/100]
Transferred within 24 hours of delivery	2019	4	299373	3491	1.17 (1.13-1.21) [/100]
Transferred within 24 hours of delivery	2019	5	316777	3687	1.16 (1.13-1.20) [/100]

Transferred within 24 hours of delivery	2019	6	304525	3681	1.21 (1.17-1.25) [/100]
Transferred within 24 hours of delivery	2019	7	334184	3965	1.19 (1.15-1.22) [/100]
Transferred within 24 hours of delivery	2019	8	342267	4148	1.21 (1.18-1.25) [/100]
Transferred within 24 hours of delivery	2019	9	326365	3751	1.15 (1.11-1.19) [/100]
Transferred within 24 hours of delivery	2019	10	325870	3912	1.20 (1.16-1.24) [/100]
Transferred within 24 hours of delivery	2019	11	298633	3526	1.18 (1.14-1.22) [/100]
Transferred within 24 hours of delivery	2019	12	309348	3513	1.14 (1.10-1.17) [/100]
Transferred within 24 hours of delivery	2020	1	305256	3561	1.17 (1.13-1.21) [/100]
Transferred within 24 hours of delivery	2020	2	283142	3291	1.16 (1.12-1.20) [/100]
Transferred within 24 hours of delivery	2020	3	302047	3327	1.10 (1.06-1.14) [/100]
Transferred within 24 hours of delivery	2020	4	290656	3144	1.08 (1.04-1.12) [/100]
Transferred within 24 hours of delivery	2020	5	301573	3329	1.10 (1.07-1.14) [/100]
Transferred within 24 hours of delivery	2020	6	302231	3437	1.14 (1.10-1.18) [/100]
Transferred within 24 hours of delivery	2020	7	321715	3738	1.16 (1.13-1.20) [/100]
Transferred within 24 hours of delivery	2020	8	319713	3458	1.08 (1.05-1.12) [/100]
Transferred within 24 hours of delivery	2020	9	311713	3394	1.09 (1.05-1.13) [/100]
Transferred within 24 hours of delivery	2020	10	305134	3525	1.16 (1.12-1.19) [/100]
Transferred within 24 hours of delivery	2020	11	282474	3148	1.11 (1.08-1.15) [/100]
Transferred within 24 hours of delivery	2020	12	289702	3288	1.13 (1.10-1.17) [/100]
Transferred within 24 hours of delivery	2021	1	277212	3254	1.17 (1.13-1.21) [/100]
Transferred within 24 hours of delivery	2021	2	266415	3099	1.16 (1.12-1.20) [/100]
Transferred within 24 hours of delivery	2021	3	302696	3553	1.17 (1.14-1.21) [/100]
Transferred within 24 hours of delivery	2021	4	293179	3411	1.16 (1.12-1.20) [/100]
Transferred within 24 hours of delivery	2021	5	300705	3519	1.17 (1.13-1.21) [/100]
Transferred within 24 hours of delivery	2021	6	313357	3752	1.20 (1.16-1.24) [/100]
Transferred within 24 hours of delivery	2021	7	325853	3864	1.19 (1.15-1.22) [/100]
Transferred within 24 hours of delivery	2021	8	330015	3966	1.20 (1.16-1.24) [/100]
Transferred within 24 hours of delivery	2021	9	325589	3720	1.14 (1.11-1.18) [/100]
Transferred within 24 hours of delivery	2021	10	315216	3662	1.16 (1.12-1.20) [/100]
Transferred within 24 hours of delivery	2021	11	301635	3401	1.13 (1.09-1.17) [/100]
Transferred within 24 hours of delivery	2021	12	310965	3708	1.19 (1.15-1.23) [/100]
Transferred within 24 hours of delivery	2022	1	294834	3399	1.15 (1.11-1.19) [/100]
Transferred within 24 hours of delivery	2022	2	275573	3060	1.11 (1.07-1.15) [/100]
Transferred within 24 hours of delivery	2022	3	307195	3405	1.11 (1.07-1.15) [/100]
Transferred within 24 hours of delivery	2022	4	288330	3418	1.19 (1.15-1.23) [/100]
Transferred within 24 hours of delivery	2022	5	297402	3512	1.18 (1.14-1.22) [/100]
Transferred within 24 hours of delivery	2022	6	305611	3555	1.16 (1.13-1.20) [/100]
Transferred within 24 hours of delivery	2022	7	320556	3843	1.20 (1.16-1.24) [/100]
Transferred within 24 hours of delivery	2022	8	335777	3877	1.15 (1.12-1.19) [/100]
Transferred within 24 hours of delivery	2022	9	312430	3491	1.12 (1.08-1.15) [/100]
Transferred within 24 hours of delivery	2022	10	307071	3606	1.17 (1.14-1.21) [/100]
Transferred within 24 hours of delivery	2022	11	307468	3558	1.16 (1.12-1.20) [/100]
Transferred within 24 hours of delivery	2022	12	315138	3559	1.13 (1.09-1.17) [/100]
Death before birth-certificate registration	2016	1	316923	898	2.83 (2.65-3.02) [/1000]
Death before birth-certificate registration	2016	2	306323	859	2.80 (2.62-3.00) [/1000]
Death before birth-certificate registration	2016	3	328830	944	2.87 (2.69-3.06) [/1000]
Death before birth-certificate registration	2016	4	313833	909	2.90 (2.71-3.09) [/1000]
Death before birth-certificate registration	2016	5	327892	884	2.70 (2.52-2.88) [/1000]
Death before birth-certificate registration	2016	6	332647	938	2.82 (2.64-3.01) [/1000]
Death before birth-certificate registration	2016	7	343050	955	2.78 (2.61-2.97) [/1000]
Death before birth-certificate registration	2016	8	358180	944	2.64 (2.47-2.81) [/1000]
Death before birth-certificate registration	2016	9	346024	923	2.67 (2.50-2.84) [/1000]
Death before birth-certificate registration	2016	10	331396	895	2.70 (2.53-2.88) [/1000]
Death before birth-certificate registration	2016	11	319769	862	2.70 (2.52-2.88) [/1000]
Death before birth-certificate registration	2016	12	325026	849	2.61 (2.44-2.79) [/1000]
Death before birth-certificate registration	2017	1	314061	887	2.82 (2.64-3.02) [/1000]
Death before birth-certificate registration	2017	2	289207	771	2.67 (2.48-2.86) [/1000]
Death before birth-certificate registration	2017	3	319785	836	2.61 (2.44-2.80) [/1000]
Death before birth-certificate registration	2017	4	300285	910	3.03 (2.84-3.23) [/1000]
Death before birth-certificate registration	2017	5	322662	867	2.69 (2.51-2.87) [/1000]
Death before birth-certificate registration	2017	6	324101	922	2.84 (2.66-3.03) [/1000]
Death before birth-certificate registration	2017	7	335131	852	2.54 (2.37-2.72) [/1000]
Death before birth-certificate registration	2017	8	352465	908	2.58 (2.41-2.75) [/1000]
Death before birth-certificate registration	2017	9	337466	838	2.48 (2.32-2.66) [/1000]
Death before birth-certificate registration	2017	10	330211	825	2.50 (2.33-2.67) [/1000]
Death before birth-certificate registration	2017	11	316757	785	2.48 (2.31-2.66) [/1000]
Death before birth-certificate registration	2017	12	316183	817	2.58 (2.41-2.77) [/1000]
Death before birth-certificate registration	2018	1	315035	879	2.79 (2.61-2.98) [/1000]
Death before birth-certificate registration	2018	2	284436	765	2.69 (2.50-2.89) [/1000]
Death before birth-certificate registration	2018	3	316264	852	2.69 (2.52-2.88) [/1000]
Death before birth-certificate registration	2018	4	298594	809	2.71 (2.53-2.90) [/1000]

Death before birth-certificate registration	2018	5	320849	799	2.49 (2.32-2.67) [/1000]
Death before birth-certificate registration	2018	6	315008	779	2.47 (2.30-2.65) [/1000]
Death before birth-certificate registration	2018	7	329199	837	2.54 (2.37-2.72) [/1000]
Death before birth-certificate registration	2018	8	344954	864	2.50 (2.34-2.68) [/1000]
Death before birth-certificate registration	2018	9	323052	821	2.54 (2.37-2.72) [/1000]
Death before birth-certificate registration	2018	10	327103	828	2.53 (2.36-2.71) [/1000]
Death before birth-certificate registration	2018	11	308864	737	2.39 (2.22-2.56) [/1000]
Death before birth-certificate registration	2018	12	311007	713	2.29 (2.13-2.47) [/1000]
Death before birth-certificate registration	2019	1	311182	712	2.29 (2.12-2.46) [/1000]
Death before birth-certificate registration	2019	2	280218	712	2.54 (2.36-2.73) [/1000]
Death before birth-certificate registration	2019	3	304471	805	2.64 (2.46-2.83) [/1000]
Death before birth-certificate registration	2019	4	299213	790	2.64 (2.46-2.83) [/1000]
Death before birth-certificate registration	2019	5	316627	772	2.44 (2.27-2.62) [/1000]
Death before birth-certificate registration	2019	6	304319	780	2.56 (2.39-2.75) [/1000]
Death before birth-certificate registration	2019	7	333942	775	2.32 (2.16-2.49) [/1000]
Death before birth-certificate registration	2019	8	342030	775	2.27 (2.11-2.43) [/1000]
Death before birth-certificate registration	2019	9	326131	701	2.15 (1.99-2.31) [/1000]
Death before birth-certificate registration	2019	10	325661	751	2.31 (2.14-2.48) [/1000]
Death before birth-certificate registration	2019	11	298405	675	2.26 (2.09-2.44) [/1000]
Death before birth-certificate registration	2019	12	309104	735	2.38 (2.21-2.56) [/1000]
Death before birth-certificate registration	2020	1	305060	747	2.45 (2.28-2.63) [/1000]
Death before birth-certificate registration	2020	2	282942	704	2.49 (2.31-2.68) [/1000]
Death before birth-certificate registration	2020	3	301870	750	2.48 (2.31-2.67) [/1000]
Death before birth-certificate registration	2020	4	290510	715	2.46 (2.28-2.65) [/1000]
Death before birth-certificate registration	2020	5	301486	746	2.47 (2.30-2.66) [/1000]
Death before birth-certificate registration	2020	6	302086	736	2.44 (2.26-2.62) [/1000]
Death before birth-certificate registration	2020	7	321560	718	2.23 (2.07-2.40) [/1000]
Death before birth-certificate registration	2020	8	319603	646	2.02 (1.87-2.18) [/1000]
Death before birth-certificate registration	2020	9	311656	627	2.01 (1.86-2.18) [/1000]
Death before birth-certificate registration	2020	10	305012	680	2.23 (2.07-2.40) [/1000]
Death before birth-certificate registration	2020	11	282413	623	2.21 (2.04-2.39) [/1000]
Death before birth-certificate registration	2020	12	289620	631	2.18 (2.01-2.36) [/1000]
Death before birth-certificate registration	2021	1	277182	693	2.50 (2.32-2.69) [/1000]
Death before birth-certificate registration	2021	2	266360	611	2.29 (2.12-2.48) [/1000]
Death before birth-certificate registration	2021	3	302700	629	2.08 (1.92-2.25) [/1000]
Death before birth-certificate registration	2021	4	293201	729	2.49 (2.31-2.67) [/1000]
Death before birth-certificate registration	2021	5	300874	688	2.29 (2.12-2.46) [/1000]
Death before birth-certificate registration	2021	6	313524	618	1.97 (1.82-2.13) [/1000]
Death before birth-certificate registration	2021	7	325989	701	2.15 (1.99-2.32) [/1000]
Death before birth-certificate registration	2021	8	330180	665	2.01 (1.86-2.17) [/1000]
Death before birth-certificate registration	2021	9	325788	659	2.02 (1.87-2.18) [/1000]
Death before birth-certificate registration	2021	10	315357	671	2.13 (1.97-2.29) [/1000]
Death before birth-certificate registration	2021	11	301861	601	1.99 (1.84-2.16) [/1000]
Death before birth-certificate registration	2021	12	311182	660	2.12 (1.96-2.29) [/1000]
Death before birth-certificate registration	2022	1	295017	647	2.19 (2.03-2.37) [/1000]
Death before birth-certificate registration	2022	2	275853	596	2.16 (1.99-2.34) [/1000]
Death before birth-certificate registration	2022	3	307698	667	2.17 (2.01-2.34) [/1000]
Death before birth-certificate registration	2022	4	288835	658	2.28 (2.11-2.46) [/1000]
Death before birth-certificate registration	2022	5	297794	710	2.38 (2.21-2.57) [/1000]
Death before birth-certificate registration	2022	6	306016	734	2.40 (2.23-2.58) [/1000]
Death before birth-certificate registration	2022	7	321035	703	2.19 (2.03-2.36) [/1000]
Death before birth-certificate registration	2022	8	336231	722	2.15 (1.99-2.31) [/1000]
Death before birth-certificate registration	2022	9	312573	642	2.05 (1.90-2.22) [/1000]
Death before birth-certificate registration	2022	10	307156	685	2.23 (2.07-2.40) [/1000]
Death before birth-certificate registration	2022	11	307476	666	2.17 (2.00-2.34) [/1000]
Death before birth-certificate registration	2022	12	315116	715	2.27 (2.11-2.44) [/1000]

Note: Details about each variable are presented in Supplementary methods. Infants with missing outcomes were excluded from the analyses of the specific outcomes.

Supplemental Table S13 Reported prevalence of the study outcomes by birth month (subgroup analysis, only infants delivered at ≥37 weeks' gestation)

Clinical outcome	Birth year	Birth month	No. at risk	No. events	Reported prevalence (95% confidence interval)
Congenital anomalies	2016	1	285262	813	2.85 (2.66-3.05) [/1000]
Congenital anomalies	2016	2	276149	855	3.10 (2.89-3.31) [/1000]
Congenital anomalies	2016	3	296302	890	3.00 (2.81-3.21) [/1000]
Congenital anomalies	2016	4	282255	815	2.89 (2.69-3.09) [/1000]
Congenital anomalies	2016	5	295006	850	2.88 (2.69-3.08) [/1000]
Congenital anomalies	2016	6	299365	837	2.80 (2.61-2.99) [/1000]
Congenital anomalies	2016	7	308619	866	2.81 (2.62-3.00) [/1000]
Congenital anomalies	2016	8	323786	925	2.86 (2.68-3.05) [/1000]
Congenital anomalies	2016	9	313643	856	2.73 (2.55-2.92) [/1000]
Congenital anomalies	2016	10	298695	829	2.78 (2.59-2.97) [/1000]
Congenital anomalies	2016	11	288188	800	2.78 (2.59-2.97) [/1000]
Congenital anomalies	2016	12	291726	769	2.64 (2.45-2.83) [/1000]
Congenital anomalies	2017	1	282016	770	2.73 (2.54-2.93) [/1000]
Congenital anomalies	2017	2	260712	759	2.91 (2.71-3.13) [/1000]
Congenital anomalies	2017	3	288281	810	2.81 (2.62-3.01) [/1000]
Congenital anomalies	2017	4	270374	761	2.81 (2.62-3.02) [/1000]
Congenital anomalies	2017	5	290447	827	2.85 (2.66-3.05) [/1000]
Congenital anomalies	2017	6	291352	839	2.88 (2.69-3.08) [/1000]
Congenital anomalies	2017	7	301469	834	2.77 (2.58-2.96) [/1000]
Congenital anomalies	2017	8	318057	907	2.85 (2.67-3.04) [/1000]
Congenital anomalies	2017	9	305500	863	2.82 (2.64-3.02) [/1000]
Congenital anomalies	2017	10	297762	831	2.79 (2.60-2.99) [/1000]
Congenital anomalies	2017	11	285506	768	2.69 (2.50-2.89) [/1000]
Congenital anomalies	2017	12	283909	825	2.91 (2.71-3.11) [/1000]
Congenital anomalies	2018	1	282988	819	2.89 (2.70-3.10) [/1000]
Congenital anomalies	2018	2	256322	770	3.00 (2.80-3.22) [/1000]
Congenital anomalies	2018	3	285104	834	2.93 (2.73-3.13) [/1000]
Congenital anomalies	2018	4	268820	790	2.94 (2.74-3.15) [/1000]
Congenital anomalies	2018	5	288175	792	2.75 (2.56-2.95) [/1000]
Congenital anomalies	2018	6	282960	805	2.84 (2.65-3.05) [/1000]
Congenital anomalies	2018	7	295835	824	2.79 (2.60-2.98) [/1000]
Congenital anomalies	2018	8	311337	869	2.79 (2.61-2.98) [/1000]
Congenital anomalies	2018	9	291771	815	2.79 (2.61-2.99) [/1000]
Congenital anomalies	2018	10	294411	911	3.09 (2.90-3.30) [/1000]
Congenital anomalies	2018	11	277618	787	2.83 (2.64-3.04) [/1000]
Congenital anomalies	2018	12	278997	771	2.76 (2.57-2.97) [/1000]
Congenital anomalies	2019	1	279333	756	2.71 (2.52-2.91) [/1000]
Congenital anomalies	2019	2	251637	736	2.92 (2.72-3.14) [/1000]
Congenital anomalies	2019	3	273087	768	2.81 (2.62-3.02) [/1000]
Congenital anomalies	2019	4	268825	702	2.61 (2.42-2.81) [/1000]
Congenital anomalies	2019	5	283937	760	2.68 (2.49-2.87) [/1000]
Congenital anomalies	2019	6	272242	772	2.84 (2.64-3.04) [/1000]
Congenital anomalies	2019	7	299580	781	2.61 (2.43-2.80) [/1000]
Congenital anomalies	2019	8	307625	846	2.75 (2.57-2.94) [/1000]
Congenital anomalies	2019	9	294673	857	2.91 (2.72-3.11) [/1000]
Congenital anomalies	2019	10	292702	764	2.61 (2.43-2.80) [/1000]
Congenital anomalies	2019	11	267192	739	2.77 (2.57-2.97) [/1000]
Congenital anomalies	2019	12	276844	773	2.79 (2.60-3.00) [/1000]
Congenital anomalies	2020	1	272987	810	2.97 (2.77-3.18) [/1000]
Congenital anomalies	2020	2	253500	699	2.76 (2.56-2.97) [/1000]
Congenital anomalies	2020	3	272222	729	2.68 (2.49-2.88) [/1000]
Congenital anomalies	2020	4	261951	720	2.75 (2.55-2.96) [/1000]
Congenital anomalies	2020	5	270970	737	2.72 (2.53-2.92) [/1000]
Congenital anomalies	2020	6	271183	831	3.06 (2.86-3.28) [/1000]
Congenital anomalies	2020	7	288818	795	2.75 (2.56-2.95) [/1000]
Congenital anomalies	2020	8	287391	829	2.88 (2.69-3.09) [/1000]
Congenital anomalies	2020	9	281560	782	2.78 (2.59-2.98) [/1000]
Congenital anomalies	2020	10	273929	744	2.72 (2.52-2.92) [/1000]
Congenital anomalies	2020	11	254215	701	2.76 (2.56-2.97) [/1000]
Congenital anomalies	2020	12	260071	716	2.75 (2.56-2.96) [/1000]
Congenital anomalies	2021	1	246479	709	2.88 (2.67-3.10) [/1000]
Congenital anomalies	2021	2	238352	703	2.95 (2.74-3.18) [/1000]
Congenital anomalies	2021	3	271449	822	3.03 (2.82-3.24) [/1000]
Congenital anomalies	2021	4	261970	791	3.02 (2.81-3.24) [/1000]
Congenital anomalies	2021	5	269110	752	2.79 (2.60-3.00) [/1000]
Congenital anomalies	2021	6	280679	756	2.69 (2.51-2.89) [/1000]
Congenital anomalies	2021	7	291919	777	2.66 (2.48-2.86) [/1000]

Congenital anomalies	2021	8	295288	824	2.79 (2.60-2.99) [/1000]
Congenital anomalies	2021	9	292566	760	2.60 (2.42-2.79) [/1000]
Congenital anomalies	2021	10	281977	798	2.83 (2.64-3.03) [/1000]
Congenital anomalies	2021	11	270183	740	2.74 (2.55-2.94) [/1000]
Congenital anomalies	2021	12	277304	719	2.59 (2.41-2.79) [/1000]
Congenital anomalies	2022	1	262670	708	2.70 (2.50-2.90) [/1000]
Congenital anomalies	2022	2	247298	734	2.97 (2.76-3.19) [/1000]
Congenital anomalies	2022	3	276060	739	2.68 (2.49-2.88) [/1000]
Congenital anomalies	2022	4	258699	709	2.74 (2.54-2.95) [/1000]
Congenital anomalies	2022	5	266100	744	2.80 (2.60-3.00) [/1000]
Congenital anomalies	2022	6	273442	747	2.73 (2.54-2.93) [/1000]
Congenital anomalies	2022	7	287161	839	2.92 (2.73-3.13) [/1000]
Congenital anomalies	2022	8	303036	850	2.80 (2.62-3.00) [/1000]
Congenital anomalies	2022	9	281272	773	2.75 (2.56-2.95) [/1000]
Congenital anomalies	2022	10	274216	727	2.65 (2.46-2.85) [/1000]
Congenital anomalies	2022	11	275054	704	2.56 (2.37-2.76) [/1000]
Congenital anomalies	2022	12	281653	749	2.66 (2.47-2.86) [/1000]
Anencephaly	2016	1	285262	17	5.96 (3.47-9.54) [/100,000]
Anencephaly	2016	2	276149	28	10.14 (6.74-14.65) [/100,000]
Anencephaly	2016	3	296302	14	4.72 (2.58-7.93) [/100,000]
Anencephaly	2016	4	282255	18	6.38 (3.78-10.08) [/100,000]
Anencephaly	2016	5	295006	12	4.07 (2.10-7.11) [/100,000]
Anencephaly	2016	6	299365	21	7.01 (4.34-10.72) [/100,000]
Anencephaly	2016	7	308619	29	9.40 (6.29-13.49) [/100,000]
Anencephaly	2016	8	323786	20	6.18 (3.77-9.54) [/100,000]
Anencephaly	2016	9	313643	19	6.06 (3.65-9.46) [/100,000]
Anencephaly	2016	10	298695	9	3.01 (1.38-5.72) [/100,000]
Anencephaly	2016	11	288188	19	6.59 (3.97-10.30) [/100,000]
Anencephaly	2016	12	291726	15	5.14 (2.88-8.48) [/100,000]
Anencephaly	2017	1	282016	21	7.45 (4.61-11.38) [/100,000]
Anencephaly	2017	2	260712	15	5.75 (3.22-9.49) [/100,000]
Anencephaly	2017	3	288281	23	7.98 (5.06-11.97) [/100,000]
Anencephaly	2017	4	270374	14	5.18 (2.83-8.69) [/100,000]
Anencephaly	2017	5	290447	16	5.51 (3.15-8.95) [/100,000]
Anencephaly	2017	6	291352	15	5.15 (2.88-8.49) [/100,000]
Anencephaly	2017	7	301469	24	7.96 (5.10-11.85) [/100,000]
Anencephaly	2017	8	318057	30	9.43 (6.36-13.46) [/100,000]
Anencephaly	2017	9	305500	33	10.80 (7.44-15.17) [/100,000]
Anencephaly	2017	10	297762	19	6.38 (3.84-9.96) [/100,000]
Anencephaly	2017	11	285506	18	6.30 (3.74-9.96) [/100,000]
Anencephaly	2017	12	283909	20	7.04 (4.30-10.88) [/100,000]
Anencephaly	2018	1	282988	18	6.36 (3.77-10.05) [/100,000]
Anencephaly	2018	2	256322	13	5.07 (2.70-8.67) [/100,000]
Anencephaly	2018	3	285104	24	8.42 (5.39-12.53) [/100,000]
Anencephaly	2018	4	268820	22	8.18 (5.13-12.39) [/100,000]
Anencephaly	2018	5	288175	15	5.21 (2.91-8.58) [/100,000]
Anencephaly	2018	6	282960	23	8.13 (5.15-12.20) [/100,000]
Anencephaly	2018	7	295835	13	4.39 (2.34-7.51) [/100,000]
Anencephaly	2018	8	311337	17	5.46 (3.18-8.74) [/100,000]
Anencephaly	2018	9	291771	17	5.83 (3.39-9.33) [/100,000]
Anencephaly	2018	10	294411	16	5.43 (3.11-8.83) [/100,000]
Anencephaly	2018	11	277618	22	7.92 (4.97-12.00) [/100,000]
Anencephaly	2018	12	278997	19	6.81 (4.10-10.63) [/100,000]
Anencephaly	2019	1	279333	15	5.37 (3.01-8.86) [/100,000]
Anencephaly	2019	2	251637	17	6.76 (3.94-10.82) [/100,000]
Anencephaly	2019	3	273087	19	6.96 (4.19-10.86) [/100,000]
Anencephaly	2019	4	268825	15	5.58 (3.12-9.20) [/100,000]
Anencephaly	2019	5	283937	18	6.34 (3.76-10.02) [/100,000]
Anencephaly	2019	6	272242	9	3.31 (1.51-6.28) [/100,000]
Anencephaly	2019	7	299580	13	4.34 (2.31-7.42) [/100,000]
Anencephaly	2019	8	307625	13	4.23 (2.25-7.23) [/100,000]
Anencephaly	2019	9	294673	25	8.48 (5.49-12.52) [/100,000]
Anencephaly	2019	10	292702	19	6.49 (3.91-10.14) [/100,000]
Anencephaly	2019	11	267192	24	8.98 (5.76-13.36) [/100,000]
Anencephaly	2019	12	276844	12	4.33 (2.24-7.57) [/100,000]
Anencephaly	2020	1	272987	21	7.69 (4.76-11.76) [/100,000]
Anencephaly	2020	2	253500	14	5.52 (3.02-9.27) [/100,000]
Anencephaly	2020	3	272222	19	6.98 (4.20-10.90) [/100,000]
Anencephaly	2020	4	261951	14	5.34 (2.92-8.97) [/100,000]
Anencephaly	2020	5	270970	18	6.64 (3.94-10.50) [/100,000]
Anencephaly	2020	6	271183	15	5.53 (3.10-9.12) [/100,000]

Anencephaly	2020	7	288818	16	5.54 (3.17-9.00) [/100,000]
Anencephaly	2020	8	287391	22	7.66 (4.80-11.59) [/100,000]
Anencephaly	2020	9	281560	12	4.26 (2.20-7.44) [/100,000]
Anencephaly	2020	10	273929	15	5.48 (3.06-9.03) [/100,000]
Anencephaly	2020	11	254215	17	6.69 (3.90-10.71) [/100,000]
Anencephaly	2020	12	260071	14	5.38 (2.94-9.03) [/100,000]
Anencephaly	2021	1	246479	14	5.68 (3.11-9.53) [/100,000]
Anencephaly	2021	2	238352	14	5.87 (3.21-9.85) [/100,000]
Anencephaly	2021	3	271449	11	4.05 (2.02-7.25) [/100,000]
Anencephaly	2021	4	261970	19	7.25 (4.37-11.33) [/100,000]
Anencephaly	2021	5	269110	20	7.43 (4.54-11.48) [/100,000]
Anencephaly	2021	6	280679	9	3.21 (1.47-6.09) [/100,000]
Anencephaly	2021	7	291919	16	5.48 (3.13-8.90) [/100,000]
Anencephaly	2021	8	295288	15	5.08 (2.84-8.38) [/100,000]
Anencephaly	2021	9	292566	20	6.84 (4.18-10.56) [/100,000]
Anencephaly	2021	10	281977	17	6.03 (3.51-9.65) [/100,000]
Anencephaly	2021	11	270183	11	4.07 (2.03-7.28) [/100,000]
Anencephaly	2021	12	277304	22	7.93 (4.97-12.01) [/100,000]
Anencephaly	2022	1	262670	17	6.47 (3.77-10.36) [/100,000]
Anencephaly	2022	2	247298	24	9.70 (6.22-14.44) [/100,000]
Anencephaly	2022	3	276060	15	5.43 (3.04-8.96) [/100,000]
Anencephaly	2022	4	258699	19	7.34 (4.42-11.47) [/100,000]
Anencephaly	2022	5	266100	26	9.77 (6.38-14.32) [/100,000]
Anencephaly	2022	6	273442	16	5.85 (3.34-9.50) [/100,000]
Anencephaly	2022	7	287161	20	6.96 (4.25-10.76) [/100,000]
Anencephaly	2022	8	303036	22	7.26 (4.55-10.99) [/100,000]
Anencephaly	2022	9	281272	19	6.76 (4.07-10.55) [/100,000]
Anencephaly	2022	10	274216	17	6.20 (3.61-9.93) [/100,000]
Anencephaly	2022	11	275054	18	6.54 (3.88-10.34) [/100,000]
Anencephaly	2022	12	281653	26	9.23 (6.03-13.53) [/100,000]
Meningomyelocele/Spina bifida	2016	1	285262	29	10.17 (6.81-14.60) [/100,000]
Meningomyelocele/Spina bifida	2016	2	276149	37	13.40 (9.43-18.47) [/100,000]
Meningomyelocele/Spina bifida	2016	3	296302	34	11.47 (7.95-16.03) [/100,000]
Meningomyelocele/Spina bifida	2016	4	282255	31	10.98 (7.46-15.59) [/100,000]
Meningomyelocele/Spina bifida	2016	5	295006	31	10.51 (7.14-14.92) [/100,000]
Meningomyelocele/Spina bifida	2016	6	299365	30	10.02 (6.76-14.31) [/100,000]
Meningomyelocele/Spina bifida	2016	7	308619	40	12.96 (9.26-17.65) [/100,000]
Meningomyelocele/Spina bifida	2016	8	323786	37	11.43 (8.05-15.75) [/100,000]
Meningomyelocele/Spina bifida	2016	9	313643	39	12.43 (8.84-17.00) [/100,000]
Meningomyelocele/Spina bifida	2016	10	298695	30	10.04 (6.78-14.34) [/100,000]
Meningomyelocele/Spina bifida	2016	11	288188	28	9.72 (6.46-14.04) [/100,000]
Meningomyelocele/Spina bifida	2016	12	291726	29	9.94 (6.66-14.28) [/100,000]
Meningomyelocele/Spina bifida	2017	1	282016	27	9.57 (6.31-13.93) [/100,000]
Meningomyelocele/Spina bifida	2017	2	260712	38	14.58 (10.31-20.01) [/100,000]
Meningomyelocele/Spina bifida	2017	3	288281	29	10.06 (6.74-14.45) [/100,000]
Meningomyelocele/Spina bifida	2017	4	270374	27	9.99 (6.58-14.53) [/100,000]
Meningomyelocele/Spina bifida	2017	5	290447	36	12.39 (8.68-17.16) [/100,000]
Meningomyelocele/Spina bifida	2017	6	291352	30	10.30 (6.95-14.70) [/100,000]
Meningomyelocele/Spina bifida	2017	7	301469	37	12.27 (8.64-16.92) [/100,000]
Meningomyelocele/Spina bifida	2017	8	318057	38	11.95 (8.45-16.40) [/100,000]
Meningomyelocele/Spina bifida	2017	9	305500	35	11.46 (7.98-15.93) [/100,000]
Meningomyelocele/Spina bifida	2017	10	297762	33	11.08 (7.63-15.56) [/100,000]
Meningomyelocele/Spina bifida	2017	11	285506	37	12.96 (9.12-17.86) [/100,000]
Meningomyelocele/Spina bifida	2017	12	283909	40	14.09 (10.07-19.18) [/100,000]
Meningomyelocele/Spina bifida	2018	1	282988	34	12.01 (8.32-16.79) [/100,000]
Meningomyelocele/Spina bifida	2018	2	256322	32	12.48 (8.54-17.62) [/100,000]
Meningomyelocele/Spina bifida	2018	3	285104	37	12.98 (9.14-17.89) [/100,000]
Meningomyelocele/Spina bifida	2018	4	268820	26	9.67 (6.32-14.17) [/100,000]
Meningomyelocele/Spina bifida	2018	5	288175	32	11.10 (7.60-15.68) [/100,000]
Meningomyelocele/Spina bifida	2018	6	282960	29	10.25 (6.86-14.72) [/100,000]
Meningomyelocele/Spina bifida	2018	7	295835	26	8.79 (5.74-12.88) [/100,000]
Meningomyelocele/Spina bifida	2018	8	311337	25	8.03 (5.20-11.85) [/100,000]
Meningomyelocele/Spina bifida	2018	9	291771	34	11.65 (8.07-16.28) [/100,000]
Meningomyelocele/Spina bifida	2018	10	294411	31	10.53 (7.15-14.95) [/100,000]
Meningomyelocele/Spina bifida	2018	11	277618	33	11.89 (8.18-16.69) [/100,000]
Meningomyelocele/Spina bifida	2018	12	278997	36	12.90 (9.04-17.86) [/100,000]
Meningomyelocele/Spina bifida	2019	1	279333	28	10.02 (6.66-14.49) [/100,000]
Meningomyelocele/Spina bifida	2019	2	251637	27	10.73 (7.07-15.61) [/100,000]
Meningomyelocele/Spina bifida	2019	3	273087	30	10.99 (7.41-15.68) [/100,000]
Meningomyelocele/Spina bifida	2019	4	268825	30	11.16 (7.53-15.93) [/100,000]
Meningomyelocele/Spina bifida	2019	5	283937	27	9.51 (6.27-13.84) [/100,000]

Meningomyelocele/Spina bifida	2019	6	272242	33	12.12 (8.34-17.02) [/100,000]
Meningomyelocele/Spina bifida	2019	7	299580	35	11.68 (8.14-16.25) [/100,000]
Meningomyelocele/Spina bifida	2019	8	307625	32	10.40 (7.12-14.68) [/100,000]
Meningomyelocele/Spina bifida	2019	9	294673	32	10.86 (7.43-15.33) [/100,000]
Meningomyelocele/Spina bifida	2019	10	292702	36	12.30 (8.61-17.03) [/100,000]
Meningomyelocele/Spina bifida	2019	11	267192	35	13.10 (9.12-18.22) [/100,000]
Meningomyelocele/Spina bifida	2019	12	276844	18	6.50 (3.85-10.28) [/100,000]
Meningomyelocele/Spina bifida	2020	1	272987	26	9.52 (6.22-13.95) [/100,000]
Meningomyelocele/Spina bifida	2020	2	253500	22	8.68 (5.44-13.14) [/100,000]
Meningomyelocele/Spina bifida	2020	3	272222	30	11.02 (7.44-15.73) [/100,000]
Meningomyelocele/Spina bifida	2020	4	261951	26	9.93 (6.48-14.54) [/100,000]
Meningomyelocele/Spina bifida	2020	5	270970	22	8.12 (5.09-12.29) [/100,000]
Meningomyelocele/Spina bifida	2020	6	271183	20	7.38 (4.50-11.39) [/100,000]
Meningomyelocele/Spina bifida	2020	7	288818	23	7.96 (5.05-11.95) [/100,000]
Meningomyelocele/Spina bifida	2020	8	287391	30	10.44 (7.04-14.90) [/100,000]
Meningomyelocele/Spina bifida	2020	9	281560	25	8.88 (5.75-13.11) [/100,000]
Meningomyelocele/Spina bifida	2020	10	273929	30	10.95 (7.39-15.63) [/100,000]
Meningomyelocele/Spina bifida	2020	11	254215	32	12.59 (8.61-17.77) [/100,000]
Meningomyelocele/Spina bifida	2020	12	260071	36	13.84 (9.70-19.16) [/100,000]
Meningomyelocele/Spina bifida	2021	1	246479	27	10.95 (7.22-15.94) [/100,000]
Meningomyelocele/Spina bifida	2021	2	238352	25	10.49 (6.79-15.48) [/100,000]
Meningomyelocele/Spina bifida	2021	3	271449	27	9.95 (6.55-14.47) [/100,000]
Meningomyelocele/Spina bifida	2021	4	261970	25	9.54 (6.18-14.09) [/100,000]
Meningomyelocele/Spina bifida	2021	5	269110	34	12.63 (8.75-17.65) [/100,000]
Meningomyelocele/Spina bifida	2021	6	280679	24	8.55 (5.48-12.72) [/100,000]
Meningomyelocele/Spina bifida	2021	7	291919	28	9.59 (6.37-13.86) [/100,000]
Meningomyelocele/Spina bifida	2021	8	295288	35	11.85 (8.26-16.48) [/100,000]
Meningomyelocele/Spina bifida	2021	9	292566	37	12.65 (8.90-17.43) [/100,000]
Meningomyelocele/Spina bifida	2021	10	281977	25	8.87 (5.74-13.09) [/100,000]
Meningomyelocele/Spina bifida	2021	11	270183	25	9.25 (5.99-13.66) [/100,000]
Meningomyelocele/Spina bifida	2021	12	277304	25	9.02 (5.83-13.31) [/100,000]
Meningomyelocele/Spina bifida	2022	1	262670	29	11.04 (7.39-15.86) [/100,000]
Meningomyelocele/Spina bifida	2022	2	247298	27	10.92 (7.20-15.88) [/100,000]
Meningomyelocele/Spina bifida	2022	3	276060	24	8.69 (5.57-12.94) [/100,000]
Meningomyelocele/Spina bifida	2022	4	258699	18	6.96 (4.12-11.00) [/100,000]
Meningomyelocele/Spina bifida	2022	5	266100	33	12.40 (8.54-17.42) [/100,000]
Meningomyelocele/Spina bifida	2022	6	273442	17	6.22 (3.62-9.95) [/100,000]
Meningomyelocele/Spina bifida	2022	7	287161	28	9.75 (6.48-14.09) [/100,000]
Meningomyelocele/Spina bifida	2022	8	303036	24	7.92 (5.07-11.78) [/100,000]
Meningomyelocele/Spina bifida	2022	9	281272	26	9.24 (6.04-13.54) [/100,000]
Meningomyelocele/Spina bifida	2022	10	274216	23	8.39 (5.32-12.59) [/100,000]
Meningomyelocele/Spina bifida	2022	11	275054	30	10.91 (7.36-15.57) [/100,000]
Meningomyelocele/Spina bifida	2022	12	281653	16	5.68 (3.25-9.23) [/100,000]
Cyanotic congenital heart disease	2016	1	285262	144	50.48 (42.57-59.43) [/100,000]
Cyanotic congenital heart disease	2016	2	276149	145	52.51 (44.31-61.78) [/100,000]
Cyanotic congenital heart disease	2016	3	296302	142	47.92 (40.37-56.48) [/100,000]
Cyanotic congenital heart disease	2016	4	282255	135	47.83 (40.10-56.61) [/100,000]
Cyanotic congenital heart disease	2016	5	295006	132	44.74 (37.44-53.06) [/100,000]
Cyanotic congenital heart disease	2016	6	299365	138	46.10 (38.73-54.46) [/100,000]
Cyanotic congenital heart disease	2016	7	308619	135	43.74 (36.68-51.77) [/100,000]
Cyanotic congenital heart disease	2016	8	323786	142	43.86 (36.94-51.69) [/100,000]
Cyanotic congenital heart disease	2016	9	313643	140	44.64 (37.55-52.67) [/100,000]
Cyanotic congenital heart disease	2016	10	298695	127	42.52 (35.45-50.59) [/100,000]
Cyanotic congenital heart disease	2016	11	288188	104	36.09 (29.49-43.72) [/100,000]
Cyanotic congenital heart disease	2016	12	291726	131	44.91 (37.55-53.28) [/100,000]
Cyanotic congenital heart disease	2017	1	282016	122	43.26 (35.93-51.65) [/100,000]
Cyanotic congenital heart disease	2017	2	260712	107	41.04 (33.64-49.59) [/100,000]
Cyanotic congenital heart disease	2017	3	288281	120	41.63 (34.51-49.77) [/100,000]
Cyanotic congenital heart disease	2017	4	270374	112	41.42 (34.11-49.84) [/100,000]
Cyanotic congenital heart disease	2017	5	290447	141	48.55 (40.87-57.25) [/100,000]
Cyanotic congenital heart disease	2017	6	291352	146	50.11 (42.31-58.93) [/100,000]
Cyanotic congenital heart disease	2017	7	301469	141	46.77 (39.37-55.16) [/100,000]
Cyanotic congenital heart disease	2017	8	318057	142	44.65 (37.61-52.62) [/100,000]
Cyanotic congenital heart disease	2017	9	305500	144	47.14 (39.75-55.49) [/100,000]
Cyanotic congenital heart disease	2017	10	297762	149	50.04 (42.33-58.75) [/100,000]
Cyanotic congenital heart disease	2017	11	285506	120	42.03 (34.85-50.26) [/100,000]
Cyanotic congenital heart disease	2017	12	283909	132	46.49 (38.90-55.13) [/100,000]
Cyanotic congenital heart disease	2018	1	282988	129	45.58 (38.06-54.16) [/100,000]
Cyanotic congenital heart disease	2018	2	256322	128	49.94 (41.66-59.37) [/100,000]
Cyanotic congenital heart disease	2018	3	285104	133	46.65 (39.06-55.28) [/100,000]
Cyanotic congenital heart disease	2018	4	268820	143	53.20 (44.84-62.66) [/100,000]

Cyanotic congenital heart disease	2018	5	288175	142	49.28 (41.51-58.08) [/100,000]
Cyanotic congenital heart disease	2018	6	282960	137	48.42 (40.65-57.23) [/100,000]
Cyanotic congenital heart disease	2018	7	295835	155	52.39 (44.47-61.32) [/100,000]
Cyanotic congenital heart disease	2018	8	311337	144	46.25 (39.01-54.45) [/100,000]
Cyanotic congenital heart disease	2018	9	291771	104	35.64 (29.12-43.19) [/100,000]
Cyanotic congenital heart disease	2018	10	294411	156	52.99 (45.00-61.98) [/100,000]
Cyanotic congenital heart disease	2018	11	277618	140	50.43 (42.42-59.51) [/100,000]
Cyanotic congenital heart disease	2018	12	278997	148	53.05 (44.85-62.31) [/100,000]
Cyanotic congenital heart disease	2019	1	279333	137	49.05 (41.18-57.98) [/100,000]
Cyanotic congenital heart disease	2019	2	251637	118	46.89 (38.82-56.15) [/100,000]
Cyanotic congenital heart disease	2019	3	273087	135	49.43 (41.45-58.51) [/100,000]
Cyanotic congenital heart disease	2019	4	268825	134	49.85 (41.77-59.03) [/100,000]
Cyanotic congenital heart disease	2019	5	283937	145	51.07 (43.10-60.09) [/100,000]
Cyanotic congenital heart disease	2019	6	272242	150	55.10 (46.64-64.65) [/100,000]
Cyanotic congenital heart disease	2019	7	299580	131	43.73 (36.56-51.89) [/100,000]
Cyanotic congenital heart disease	2019	8	307625	162	52.66 (44.87-61.42) [/100,000]
Cyanotic congenital heart disease	2019	9	294673	138	46.83 (39.35-55.33) [/100,000]
Cyanotic congenital heart disease	2019	10	292702	144	49.20 (41.49-57.92) [/100,000]
Cyanotic congenital heart disease	2019	11	267192	114	42.67 (35.20-51.25) [/100,000]
Cyanotic congenital heart disease	2019	12	276844	141	50.93 (42.87-60.06) [/100,000]
Cyanotic congenital heart disease	2020	1	272987	133	48.72 (40.79-57.74) [/100,000]
Cyanotic congenital heart disease	2020	2	253500	114	44.97 (37.10-54.02) [/100,000]
Cyanotic congenital heart disease	2020	3	272222	159	58.41 (49.68-68.22) [/100,000]
Cyanotic congenital heart disease	2020	4	261951	151	57.64 (48.82-67.60) [/100,000]
Cyanotic congenital heart disease	2020	5	270970	136	50.19 (42.11-59.37) [/100,000]
Cyanotic congenital heart disease	2020	6	271183	158	58.26 (49.53-68.09) [/100,000]
Cyanotic congenital heart disease	2020	7	288818	172	59.55 (50.99-69.15) [/100,000]
Cyanotic congenital heart disease	2020	8	287391	165	57.41 (48.99-66.87) [/100,000]
Cyanotic congenital heart disease	2020	9	281560	165	58.60 (50.00-68.25) [/100,000]
Cyanotic congenital heart disease	2020	10	273929	132	48.19 (40.32-57.14) [/100,000]
Cyanotic congenital heart disease	2020	11	254215	105	41.30 (33.78-50.00) [/100,000]
Cyanotic congenital heart disease	2020	12	260071	135	51.91 (43.52-61.44) [/100,000]
Cyanotic congenital heart disease	2021	1	246479	125	50.71 (42.22-60.42) [/100,000]
Cyanotic congenital heart disease	2021	2	238352	150	62.93 (53.27-73.84) [/100,000]
Cyanotic congenital heart disease	2021	3	271449	182	67.05 (57.66-77.52) [/100,000]
Cyanotic congenital heart disease	2021	4	261970	171	65.27 (55.86-75.82) [/100,000]
Cyanotic congenital heart disease	2021	5	269110	151	56.11 (47.52-65.81) [/100,000]
Cyanotic congenital heart disease	2021	6	280679	136	48.45 (40.65-57.31) [/100,000]
Cyanotic congenital heart disease	2021	7	291919	144	49.33 (41.60-58.07) [/100,000]
Cyanotic congenital heart disease	2021	8	295288	172	58.25 (49.87-67.63) [/100,000]
Cyanotic congenital heart disease	2021	9	292566	144	49.22 (41.51-57.94) [/100,000]
Cyanotic congenital heart disease	2021	10	281977	147	52.13 (44.05-61.27) [/100,000]
Cyanotic congenital heart disease	2021	11	270183	147	54.41 (45.97-63.94) [/100,000]
Cyanotic congenital heart disease	2021	12	277304	118	42.55 (35.22-50.96) [/100,000]
Cyanotic congenital heart disease	2022	1	262670	133	50.63 (42.40-60.00) [/100,000]
Cyanotic congenital heart disease	2022	2	247298	133	53.78 (45.03-63.73) [/100,000]
Cyanotic congenital heart disease	2022	3	276060	161	58.32 (49.66-68.05) [/100,000]
Cyanotic congenital heart disease	2022	4	258699	144	55.66 (46.95-65.53) [/100,000]
Cyanotic congenital heart disease	2022	5	266100	134	50.36 (42.19-59.64) [/100,000]
Cyanotic congenital heart disease	2022	6	273442	154	56.32 (47.78-65.95) [/100,000]
Cyanotic congenital heart disease	2022	7	287161	185	64.42 (55.48-74.40) [/100,000]
Cyanotic congenital heart disease	2022	8	303036	171	56.43 (48.29-65.55) [/100,000]
Cyanotic congenital heart disease	2022	9	281272	160	56.88 (48.41-66.41) [/100,000]
Cyanotic congenital heart disease	2022	10	274216	134	48.87 (40.94-57.87) [/100,000]
Cyanotic congenital heart disease	2022	11	275054	132	47.99 (40.15-56.91) [/100,000]
Cyanotic congenital heart disease	2022	12	281653	156	55.39 (47.04-64.79) [/100,000]
Congenital diaphragmatic hernia	2016	1	285262	25	8.76 (5.67-12.94) [/100,000]
Congenital diaphragmatic hernia	2016	2	276149	33	11.95 (8.23-16.78) [/100,000]
Congenital diaphragmatic hernia	2016	3	296302	36	12.15 (8.51-16.82) [/100,000]
Congenital diaphragmatic hernia	2016	4	282255	25	8.86 (5.73-13.07) [/100,000]
Congenital diaphragmatic hernia	2016	5	295006	27	9.15 (6.03-13.32) [/100,000]
Congenital diaphragmatic hernia	2016	6	299365	42	14.03 (10.11-18.96) [/100,000]
Congenital diaphragmatic hernia	2016	7	308619	40	12.96 (9.26-17.65) [/100,000]
Congenital diaphragmatic hernia	2016	8	323786	35	10.81 (7.53-15.03) [/100,000]
Congenital diaphragmatic hernia	2016	9	313643	17	5.42 (3.16-8.68) [/100,000]
Congenital diaphragmatic hernia	2016	10	298695	25	8.37 (5.42-12.36) [/100,000]
Congenital diaphragmatic hernia	2016	11	288188	28	9.72 (6.46-14.04) [/100,000]
Congenital diaphragmatic hernia	2016	12	291726	31	10.63 (7.22-15.08) [/100,000]
Congenital diaphragmatic hernia	2017	1	282016	24	8.51 (5.45-12.66) [/100,000]
Congenital diaphragmatic hernia	2017	2	260712	26	9.97 (6.51-14.61) [/100,000]
Congenital diaphragmatic hernia	2017	3	288281	37	12.83 (9.04-17.69) [/100,000]

Congenital diaphragmatic hernia	2017	4	270374	26	9.62 (6.28-14.09) [/100,000]
Congenital diaphragmatic hernia	2017	5	290447	21	7.23 (4.48-11.05) [/100,000]
Congenital diaphragmatic hernia	2017	6	291352	30	10.30 (6.95-14.70) [/100,000]
Congenital diaphragmatic hernia	2017	7	301469	40	13.27 (9.48-18.07) [/100,000]
Congenital diaphragmatic hernia	2017	8	318057	39	12.26 (8.72-16.76) [/100,000]
Congenital diaphragmatic hernia	2017	9	305500	23	7.53 (4.77-11.30) [/100,000]
Congenital diaphragmatic hernia	2017	10	297762	28	9.40 (6.25-13.59) [/100,000]
Congenital diaphragmatic hernia	2017	11	285506	25	8.76 (5.67-12.93) [/100,000]
Congenital diaphragmatic hernia	2017	12	283909	26	9.16 (5.98-13.42) [/100,000]
Congenital diaphragmatic hernia	2018	1	282988	33	11.66 (8.03-16.38) [/100,000]
Congenital diaphragmatic hernia	2018	2	256322	26	10.14 (6.63-14.86) [/100,000]
Congenital diaphragmatic hernia	2018	3	285104	25	8.77 (5.67-12.94) [/100,000]
Congenital diaphragmatic hernia	2018	4	268820	28	10.42 (6.92-15.05) [/100,000]
Congenital diaphragmatic hernia	2018	5	288175	36	12.49 (8.75-17.29) [/100,000]
Congenital diaphragmatic hernia	2018	6	282960	24	8.48 (5.43-12.62) [/100,000]
Congenital diaphragmatic hernia	2018	7	295835	23	7.77 (4.93-11.67) [/100,000]
Congenital diaphragmatic hernia	2018	8	311337	35	11.24 (7.83-15.63) [/100,000]
Congenital diaphragmatic hernia	2018	9	291771	31	10.62 (7.22-15.08) [/100,000]
Congenital diaphragmatic hernia	2018	10	294411	32	10.87 (7.43-15.34) [/100,000]
Congenital diaphragmatic hernia	2018	11	277618	24	8.64 (5.54-12.86) [/100,000]
Congenital diaphragmatic hernia	2018	12	278997	26	9.32 (6.09-13.65) [/100,000]
Congenital diaphragmatic hernia	2019	1	279333	23	8.23 (5.22-12.35) [/100,000]
Congenital diaphragmatic hernia	2019	2	251637	20	7.95 (4.85-12.27) [/100,000]
Congenital diaphragmatic hernia	2019	3	273087	36	13.18 (9.23-18.25) [/100,000]
Congenital diaphragmatic hernia	2019	4	268825	22	8.18 (5.13-12.39) [/100,000]
Congenital diaphragmatic hernia	2019	5	283937	34	11.97 (8.29-16.73) [/100,000]
Congenital diaphragmatic hernia	2019	6	272242	27	9.92 (6.54-14.43) [/100,000]
Congenital diaphragmatic hernia	2019	7	299580	33	11.02 (7.58-15.47) [/100,000]
Congenital diaphragmatic hernia	2019	8	307625	30	9.75 (6.58-13.92) [/100,000]
Congenital diaphragmatic hernia	2019	9	294673	27	9.16 (6.04-13.33) [/100,000]
Congenital diaphragmatic hernia	2019	10	292702	35	11.96 (8.33-16.63) [/100,000]
Congenital diaphragmatic hernia	2019	11	267192	21	7.86 (4.87-12.01) [/100,000]
Congenital diaphragmatic hernia	2019	12	276844	34	12.28 (8.51-17.16) [/100,000]
Congenital diaphragmatic hernia	2020	1	272987	33	12.09 (8.32-16.98) [/100,000]
Congenital diaphragmatic hernia	2020	2	253500	21	8.28 (5.13-12.66) [/100,000]
Congenital diaphragmatic hernia	2020	3	272222	28	10.29 (6.83-14.87) [/100,000]
Congenital diaphragmatic hernia	2020	4	261951	31	11.83 (8.04-16.80) [/100,000]
Congenital diaphragmatic hernia	2020	5	270970	26	9.60 (6.27-14.06) [/100,000]
Congenital diaphragmatic hernia	2020	6	271183	35	12.91 (8.99-17.95) [/100,000]
Congenital diaphragmatic hernia	2020	7	288818	23	7.96 (5.05-11.95) [/100,000]
Congenital diaphragmatic hernia	2020	8	287391	30	10.44 (7.04-14.90) [/100,000]
Congenital diaphragmatic hernia	2020	9	281560	30	10.65 (7.19-15.21) [/100,000]
Congenital diaphragmatic hernia	2020	10	273929	27	9.86 (6.50-14.34) [/100,000]
Congenital diaphragmatic hernia	2020	11	254215	30	11.80 (7.96-16.85) [/100,000]
Congenital diaphragmatic hernia	2020	12	260071	26	10.00 (6.53-14.65) [/100,000]
Congenital diaphragmatic hernia	2021	1	246479	35	14.20 (9.89-19.75) [/100,000]
Congenital diaphragmatic hernia	2021	2	238352	18	7.55 (4.48-11.93) [/100,000]
Congenital diaphragmatic hernia	2021	3	271449	32	11.79 (8.06-16.64) [/100,000]
Congenital diaphragmatic hernia	2021	4	261970	31	11.83 (8.04-16.80) [/100,000]
Congenital diaphragmatic hernia	2021	5	269110	19	7.06 (4.25-11.03) [/100,000]
Congenital diaphragmatic hernia	2021	6	280679	31	11.04 (7.50-15.68) [/100,000]
Congenital diaphragmatic hernia	2021	7	291919	15	5.14 (2.88-8.47) [/100,000]
Congenital diaphragmatic hernia	2021	8	295288	27	9.14 (6.03-13.30) [/100,000]
Congenital diaphragmatic hernia	2021	9	292566	38	12.99 (9.19-17.83) [/100,000]
Congenital diaphragmatic hernia	2021	10	281977	31	10.99 (7.47-15.60) [/100,000]
Congenital diaphragmatic hernia	2021	11	270183	21	7.77 (4.81-11.88) [/100,000]
Congenital diaphragmatic hernia	2021	12	277304	34	12.26 (8.49-17.13) [/100,000]
Congenital diaphragmatic hernia	2022	1	262670	30	11.42 (7.71-16.30) [/100,000]
Congenital diaphragmatic hernia	2022	2	247298	25	10.11 (6.54-14.92) [/100,000]
Congenital diaphragmatic hernia	2022	3	276060	34	12.32 (8.53-17.21) [/100,000]
Congenital diaphragmatic hernia	2022	4	258699	35	13.53 (9.42-18.82) [/100,000]
Congenital diaphragmatic hernia	2022	5	266100	25	9.39 (6.08-13.87) [/100,000]
Congenital diaphragmatic hernia	2022	6	273442	23	8.41 (5.33-12.62) [/100,000]
Congenital diaphragmatic hernia	2022	7	287161	17	5.92 (3.45-9.48) [/100,000]
Congenital diaphragmatic hernia	2022	8	303036	40	13.20 (9.43-17.97) [/100,000]
Congenital diaphragmatic hernia	2022	9	281272	37	13.15 (9.26-18.13) [/100,000]
Congenital diaphragmatic hernia	2022	10	274216	24	8.75 (5.61-13.02) [/100,000]
Congenital diaphragmatic hernia	2022	11	275054	31	11.27 (7.66-16.00) [/100,000]
Congenital diaphragmatic hernia	2022	12	281653	39	13.85 (9.85-18.93) [/100,000]
Omphalocele	2016	1	285262	20	7.01 (4.28-10.83) [/100,000]
Omphalocele	2016	2	276149	12	4.35 (2.25-7.59) [/100,000]

Omphalocele	2016	3	296302	28	9.45 (6.28-13.66) [/100,000]
Omphalocele	2016	4	282255	23	8.15 (5.17-12.23) [/100,000]
Omphalocele	2016	5	295006	23	7.80 (4.94-11.70) [/100,000]
Omphalocele	2016	6	299365	17	5.68 (3.31-9.09) [/100,000]
Omphalocele	2016	7	308619	24	7.78 (4.98-11.57) [/100,000]
Omphalocele	2016	8	323786	20	6.18 (3.77-9.54) [/100,000]
Omphalocele	2016	9	313643	16	5.10 (2.92-8.28) [/100,000]
Omphalocele	2016	10	298695	20	6.70 (4.09-10.34) [/100,000]
Omphalocele	2016	11	288188	23	7.98 (5.06-11.98) [/100,000]
Omphalocele	2016	12	291726	22	7.54 (4.73-11.42) [/100,000]
Omphalocele	2017	1	282016	18	6.38 (3.78-10.09) [/100,000]
Omphalocele	2017	2	260712	20	7.67 (4.69-11.85) [/100,000]
Omphalocele	2017	3	288281	20	6.94 (4.24-10.71) [/100,000]
Omphalocele	2017	4	270374	15	5.55 (3.11-9.15) [/100,000]
Omphalocele	2017	5	290447	15	5.16 (2.89-8.52) [/100,000]
Omphalocele	2017	6	291352	16	5.49 (3.14-8.92) [/100,000]
Omphalocele	2017	7	301469	10	3.32 (1.59-6.10) [/100,000]
Omphalocele	2017	8	318057	22	6.92 (4.33-10.47) [/100,000]
Omphalocele	2017	9	305500	22	7.20 (4.51-10.90) [/100,000]
Omphalocele	2017	10	297762	24	8.06 (5.16-11.99) [/100,000]
Omphalocele	2017	11	285506	21	7.36 (4.55-11.24) [/100,000]
Omphalocele	2017	12	283909	20	7.04 (4.30-10.88) [/100,000]
Omphalocele	2018	1	282988	17	6.01 (3.50-9.62) [/100,000]
Omphalocele	2018	2	256322	18	7.02 (4.16-11.10) [/100,000]
Omphalocele	2018	3	285104	29	10.17 (6.81-14.61) [/100,000]
Omphalocele	2018	4	268820	19	7.07 (4.26-11.04) [/100,000]
Omphalocele	2018	5	288175	12	4.16 (2.15-7.27) [/100,000]
Omphalocele	2018	6	282960	16	5.65 (3.23-9.18) [/100,000]
Omphalocele	2018	7	295835	12	4.06 (2.10-7.09) [/100,000]
Omphalocele	2018	8	311337	25	8.03 (5.20-11.85) [/100,000]
Omphalocele	2018	9	291771	20	6.85 (4.19-10.59) [/100,000]
Omphalocele	2018	10	294411	16	5.43 (3.11-8.83) [/100,000]
Omphalocele	2018	11	277618	20	7.20 (4.40-11.13) [/100,000]
Omphalocele	2018	12	278997	24	8.60 (5.51-12.80) [/100,000]
Omphalocele	2019	1	279333	13	4.65 (2.48-7.96) [/100,000]
Omphalocele	2019	2	251637	19	7.55 (4.55-11.79) [/100,000]
Omphalocele	2019	3	273087	14	5.13 (2.80-8.60) [/100,000]
Omphalocele	2019	4	268825	25	9.30 (6.02-13.73) [/100,000]
Omphalocele	2019	5	283937	22	7.75 (4.86-11.73) [/100,000]
Omphalocele	2019	6	272242	15	5.51 (3.08-9.09) [/100,000]
Omphalocele	2019	7	299580	19	6.34 (3.82-9.90) [/100,000]
Omphalocele	2019	8	307625	17	5.53 (3.22-8.85) [/100,000]
Omphalocele	2019	9	294673	27	9.16 (6.04-13.33) [/100,000]
Omphalocele	2019	10	292702	16	5.47 (3.12-8.88) [/100,000]
Omphalocele	2019	11	267192	14	5.24 (2.86-8.79) [/100,000]
Omphalocele	2019	12	276844	15	5.42 (3.03-8.94) [/100,000]
Omphalocele	2020	1	272987	15	5.49 (3.08-9.06) [/100,000]
Omphalocele	2020	2	253500	16	6.31 (3.61-10.25) [/100,000]
Omphalocele	2020	3	272222	12	4.41 (2.28-7.70) [/100,000]
Omphalocele	2020	4	261951	18	6.87 (4.07-10.86) [/100,000]
Omphalocele	2020	5	270970	23	8.49 (5.38-12.74) [/100,000]
Omphalocele	2020	6	271183	23	8.48 (5.38-12.73) [/100,000]
Omphalocele	2020	7	288818	11	3.81 (1.90-6.81) [/100,000]
Omphalocele	2020	8	287391	15	5.22 (2.92-8.61) [/100,000]
Omphalocele	2020	9	281560	19	6.75 (4.06-10.54) [/100,000]
Omphalocele	2020	10	273929	12	4.38 (2.26-7.65) [/100,000]
Omphalocele	2020	11	254215	18	7.08 (4.20-11.19) [/100,000]
Omphalocele	2020	12	260071	17	6.54 (3.81-10.47) [/100,000]
Omphalocele	2021	1	246479	13	5.27 (2.81-9.02) [/100,000]
Omphalocele	2021	2	238352	14	5.87 (3.21-9.85) [/100,000]
Omphalocele	2021	3	271449	19	7.00 (4.21-10.93) [/100,000]
Omphalocele	2021	4	261970	19	7.25 (4.37-11.33) [/100,000]
Omphalocele	2021	5	269110	22	8.18 (5.12-12.38) [/100,000]
Omphalocele	2021	6	280679	27	9.62 (6.34-14.00) [/100,000]
Omphalocele	2021	7	291919	15	5.14 (2.88-8.47) [/100,000]
Omphalocele	2021	8	295288	17	5.76 (3.35-9.22) [/100,000]
Omphalocele	2021	9	292566	16	5.47 (3.13-8.88) [/100,000]
Omphalocele	2021	10	281977	14	4.96 (2.71-8.33) [/100,000]
Omphalocele	2021	11	270183	19	7.03 (4.23-10.98) [/100,000]
Omphalocele	2021	12	277304	16	5.77 (3.30-9.37) [/100,000]
Omphalocele	2022	1	262670	19	7.23 (4.36-11.30) [/100,000]

Omphalocele	2022	2	247298	15	6.07 (3.39-10.00) [/100,000]
Omphalocele	2022	3	276060	18	6.52 (3.86-10.30) [/100,000]
Omphalocele	2022	4	258699	10	3.87 (1.85-7.11) [/100,000]
Omphalocele	2022	5	266100	21	7.89 (4.89-12.06) [/100,000]
Omphalocele	2022	6	273442	17	6.22 (3.62-9.95) [/100,000]
Omphalocele	2022	7	287161	15	5.22 (2.92-8.62) [/100,000]
Omphalocele	2022	8	303036	17	5.61 (3.27-8.98) [/100,000]
Omphalocele	2022	9	281272	19	6.76 (4.07-10.55) [/100,000]
Omphalocele	2022	10	274216	20	7.29 (4.46-11.26) [/100,000]
Omphalocele	2022	11	275054	12	4.36 (2.25-7.62) [/100,000]
Omphalocele	2022	12	281653	17	6.04 (3.52-9.66) [/100,000]
Gastroschisis	2016	1	285262	32	11.22 (7.67-15.84) [/100,000]
Gastroschisis	2016	2	276149	31	11.23 (7.63-15.93) [/100,000]
Gastroschisis	2016	3	296302	38	12.82 (9.08-17.60) [/100,000]
Gastroschisis	2016	4	282255	25	8.86 (5.73-13.07) [/100,000]
Gastroschisis	2016	5	295006	31	10.51 (7.14-14.92) [/100,000]
Gastroschisis	2016	6	299365	29	9.69 (6.49-13.91) [/100,000]
Gastroschisis	2016	7	308619	21	6.80 (4.21-10.40) [/100,000]
Gastroschisis	2016	8	323786	28	8.65 (5.75-12.50) [/100,000]
Gastroschisis	2016	9	313643	35	11.16 (7.77-15.52) [/100,000]
Gastroschisis	2016	10	298695	31	10.38 (7.05-14.73) [/100,000]
Gastroschisis	2016	11	288188	30	10.41 (7.02-14.86) [/100,000]
Gastroschisis	2016	12	291726	30	10.28 (6.94-14.68) [/100,000]
Gastroschisis	2017	1	282016	37	13.12 (9.24-18.08) [/100,000]
Gastroschisis	2017	2	260712	23	8.82 (5.59-13.24) [/100,000]
Gastroschisis	2017	3	288281	28	9.71 (6.45-14.04) [/100,000]
Gastroschisis	2017	4	270374	30	11.10 (7.49-15.84) [/100,000]
Gastroschisis	2017	5	290447	26	8.95 (5.85-13.12) [/100,000]
Gastroschisis	2017	6	291352	37	12.70 (8.94-17.50) [/100,000]
Gastroschisis	2017	7	301469	32	10.61 (7.26-14.98) [/100,000]
Gastroschisis	2017	8	318057	26	8.17 (5.34-11.98) [/100,000]
Gastroschisis	2017	9	305500	34	11.13 (7.71-15.55) [/100,000]
Gastroschisis	2017	10	297762	22	7.39 (4.63-11.19) [/100,000]
Gastroschisis	2017	11	285506	36	12.61 (8.83-17.46) [/100,000]
Gastroschisis	2017	12	283909	28	9.86 (6.55-14.25) [/100,000]
Gastroschisis	2018	1	282988	29	10.25 (6.86-14.72) [/100,000]
Gastroschisis	2018	2	256322	23	8.97 (5.69-13.46) [/100,000]
Gastroschisis	2018	3	285104	33	11.57 (7.97-16.25) [/100,000]
Gastroschisis	2018	4	268820	20	7.44 (4.54-11.49) [/100,000]
Gastroschisis	2018	5	288175	25	8.68 (5.61-12.81) [/100,000]
Gastroschisis	2018	6	282960	35	12.37 (8.62-17.20) [/100,000]
Gastroschisis	2018	7	295835	32	10.82 (7.40-15.27) [/100,000]
Gastroschisis	2018	8	311337	18	5.78 (3.43-9.14) [/100,000]
Gastroschisis	2018	9	291771	24	8.23 (5.27-12.24) [/100,000]
Gastroschisis	2018	10	294411	27	9.17 (6.04-13.34) [/100,000]
Gastroschisis	2018	11	277618	28	10.09 (6.70-14.58) [/100,000]
Gastroschisis	2018	12	278997	23	8.24 (5.23-12.37) [/100,000]
Gastroschisis	2019	1	279333	25	8.95 (5.79-13.21) [/100,000]
Gastroschisis	2019	2	251637	21	8.35 (5.17-12.76) [/100,000]
Gastroschisis	2019	3	273087	16	5.86 (3.35-9.51) [/100,000]
Gastroschisis	2019	4	268825	22	8.18 (5.13-12.39) [/100,000]
Gastroschisis	2019	5	283937	30	10.57 (7.13-15.08) [/100,000]
Gastroschisis	2019	6	272242	36	13.22 (9.26-18.31) [/100,000]
Gastroschisis	2019	7	299580	30	10.01 (6.76-14.30) [/100,000]
Gastroschisis	2019	8	307625	29	9.43 (6.31-13.54) [/100,000]
Gastroschisis	2019	9	294673	35	11.88 (8.27-16.52) [/100,000]
Gastroschisis	2019	10	292702	16	5.47 (3.12-8.88) [/100,000]
Gastroschisis	2019	11	267192	26	9.73 (6.36-14.26) [/100,000]
Gastroschisis	2019	12	276844	26	9.39 (6.13-13.76) [/100,000]
Gastroschisis	2020	1	272987	25	9.16 (5.93-13.52) [/100,000]
Gastroschisis	2020	2	253500	23	9.07 (5.75-13.61) [/100,000]
Gastroschisis	2020	3	272222	24	8.82 (5.65-13.12) [/100,000]
Gastroschisis	2020	4	261951	24	9.16 (5.87-13.63) [/100,000]
Gastroschisis	2020	5	270970	16	5.90 (3.38-9.59) [/100,000]
Gastroschisis	2020	6	271183	22	8.11 (5.08-12.28) [/100,000]
Gastroschisis	2020	7	288818	32	11.08 (7.58-15.64) [/100,000]
Gastroschisis	2020	8	287391	18	6.26 (3.71-9.90) [/100,000]
Gastroschisis	2020	9	281560	17	6.04 (3.52-9.67) [/100,000]
Gastroschisis	2020	10	273929	26	9.49 (6.20-13.91) [/100,000]
Gastroschisis	2020	11	254215	24	9.44 (6.05-14.05) [/100,000]
Gastroschisis	2020	12	260071	21	8.07 (5.00-12.34) [/100,000]

Gastroschisis	2021	1	246479	18	7.30 (4.33-11.54) [/100,000]
Gastroschisis	2021	2	238352	21	8.81 (5.45-13.47) [/100,000]
Gastroschisis	2021	3	271449	22	8.10 (5.08-12.27) [/100,000]
Gastroschisis	2021	4	261970	19	7.25 (4.37-11.33) [/100,000]
Gastroschisis	2021	5	269110	13	4.83 (2.57-8.26) [/100,000]
Gastroschisis	2021	6	280679	22	7.84 (4.91-11.87) [/100,000]
Gastroschisis	2021	7	291919	22	7.54 (4.72-11.41) [/100,000]
Gastroschisis	2021	8	295288	14	4.74 (2.59-7.95) [/100,000]
Gastroschisis	2021	9	292566	13	4.44 (2.37-7.60) [/100,000]
Gastroschisis	2021	10	281977	18	6.38 (3.78-10.09) [/100,000]
Gastroschisis	2021	11	270183	20	7.40 (4.52-11.43) [/100,000]
Gastroschisis	2021	12	277304	17	6.13 (3.57-9.82) [/100,000]
Gastroschisis	2022	1	262670	17	6.47 (3.77-10.36) [/100,000]
Gastroschisis	2022	2	247298	16	6.47 (3.70-10.51) [/100,000]
Gastroschisis	2022	3	276060	13	4.71 (2.51-8.05) [/100,000]
Gastroschisis	2022	4	258699	23	8.89 (5.64-13.34) [/100,000]
Gastroschisis	2022	5	266100	14	5.26 (2.88-8.83) [/100,000]
Gastroschisis	2022	6	273442	20	7.31 (4.47-11.30) [/100,000]
Gastroschisis	2022	7	287161	14	4.88 (2.67-8.18) [/100,000]
Gastroschisis	2022	8	303036	14	4.62 (2.53-7.75) [/100,000]
Gastroschisis	2022	9	281272	19	6.76 (4.07-10.55) [/100,000]
Gastroschisis	2022	10	274216	17	6.20 (3.61-9.93) [/100,000]
Gastroschisis	2022	11	275054	14	5.09 (2.78-8.54) [/100,000]
Gastroschisis	2022	12	281653	14	4.97 (2.72-8.34) [/100,000]
Limb reduction defect	2016	1	285262	37	12.97 (9.13-17.88) [/100,000]
Limb reduction defect	2016	2	276149	33	11.95 (8.23-16.78) [/100,000]
Limb reduction defect	2016	3	296302	28	9.45 (6.28-13.66) [/100,000]
Limb reduction defect	2016	4	282255	38	13.46 (9.53-18.48) [/100,000]
Limb reduction defect	2016	5	295006	39	13.22 (9.40-18.07) [/100,000]
Limb reduction defect	2016	6	299365	24	8.02 (5.14-11.93) [/100,000]
Limb reduction defect	2016	7	308619	42	13.61 (9.81-18.39) [/100,000]
Limb reduction defect	2016	8	323786	43	13.28 (9.61-17.89) [/100,000]
Limb reduction defect	2016	9	313643	30	9.57 (6.45-13.65) [/100,000]
Limb reduction defect	2016	10	298695	37	12.39 (8.72-17.07) [/100,000]
Limb reduction defect	2016	11	288188	30	10.41 (7.02-14.86) [/100,000]
Limb reduction defect	2016	12	291726	30	10.28 (6.94-14.68) [/100,000]
Limb reduction defect	2017	1	282016	25	8.86 (5.74-13.09) [/100,000]
Limb reduction defect	2017	2	260712	31	11.89 (8.08-16.88) [/100,000]
Limb reduction defect	2017	3	288281	31	10.75 (7.31-15.26) [/100,000]
Limb reduction defect	2017	4	270374	33	12.21 (8.40-17.14) [/100,000]
Limb reduction defect	2017	5	290447	29	9.98 (6.69-14.34) [/100,000]
Limb reduction defect	2017	6	291352	37	12.70 (8.94-17.50) [/100,000]
Limb reduction defect	2017	7	301469	25	8.29 (5.37-12.24) [/100,000]
Limb reduction defect	2017	8	318057	32	10.06 (6.88-14.20) [/100,000]
Limb reduction defect	2017	9	305500	36	11.78 (8.25-16.31) [/100,000]
Limb reduction defect	2017	10	297762	34	11.42 (7.91-15.96) [/100,000]
Limb reduction defect	2017	11	285506	34	11.91 (8.25-16.64) [/100,000]
Limb reduction defect	2017	12	283909	38	13.38 (9.47-18.37) [/100,000]
Limb reduction defect	2018	1	282988	33	11.66 (8.03-16.38) [/100,000]
Limb reduction defect	2018	2	256322	28	10.92 (7.26-15.79) [/100,000]
Limb reduction defect	2018	3	285104	29	10.17 (6.81-14.61) [/100,000]
Limb reduction defect	2018	4	268820	33	12.28 (8.45-17.24) [/100,000]
Limb reduction defect	2018	5	288175	26	9.02 (5.89-13.22) [/100,000]
Limb reduction defect	2018	6	282960	32	11.31 (7.74-15.96) [/100,000]
Limb reduction defect	2018	7	295835	27	9.13 (6.01-13.28) [/100,000]
Limb reduction defect	2018	8	311337	36	11.56 (8.10-16.01) [/100,000]
Limb reduction defect	2018	9	291771	28	9.60 (6.38-13.87) [/100,000]
Limb reduction defect	2018	10	294411	34	11.55 (8.00-16.14) [/100,000]
Limb reduction defect	2018	11	277618	34	12.25 (8.48-17.11) [/100,000]
Limb reduction defect	2018	12	278997	22	7.89 (4.94-11.94) [/100,000]
Limb reduction defect	2019	1	279333	34	12.17 (8.43-17.01) [/100,000]
Limb reduction defect	2019	2	251637	36	14.31 (10.02-19.81) [/100,000]
Limb reduction defect	2019	3	273087	24	8.79 (5.63-13.08) [/100,000]
Limb reduction defect	2019	4	268825	20	7.44 (4.54-11.49) [/100,000]
Limb reduction defect	2019	5	283937	21	7.40 (4.58-11.31) [/100,000]
Limb reduction defect	2019	6	272242	27	9.92 (6.54-14.43) [/100,000]
Limb reduction defect	2019	7	299580	27	9.01 (5.94-13.11) [/100,000]
Limb reduction defect	2019	8	307625	22	7.15 (4.48-10.83) [/100,000]
Limb reduction defect	2019	9	294673	33	11.20 (7.71-15.73) [/100,000]
Limb reduction defect	2019	10	292702	22	7.52 (4.71-11.38) [/100,000]
Limb reduction defect	2019	11	267192	20	7.49 (4.57-11.56) [/100,000]

Limb reduction defect	2019	12	276844	34	12.28 (8.51-17.16) [/100,000]
Limb reduction defect	2020	1	272987	27	9.89 (6.52-14.39) [/100,000]
Limb reduction defect	2020	2	253500	27	10.65 (7.02-15.50) [/100,000]
Limb reduction defect	2020	3	272222	36	13.22 (9.26-18.31) [/100,000]
Limb reduction defect	2020	4	261951	21	8.02 (4.96-12.25) [/100,000]
Limb reduction defect	2020	5	270970	25	9.23 (5.97-13.62) [/100,000]
Limb reduction defect	2020	6	271183	28	10.33 (6.86-14.92) [/100,000]
Limb reduction defect	2020	7	288818	23	7.96 (5.05-11.95) [/100,000]
Limb reduction defect	2020	8	287391	32	11.13 (7.62-15.72) [/100,000]
Limb reduction defect	2020	9	281560	22	7.81 (4.90-11.83) [/100,000]
Limb reduction defect	2020	10	273929	28	10.22 (6.79-14.77) [/100,000]
Limb reduction defect	2020	11	254215	25	9.83 (6.36-14.52) [/100,000]
Limb reduction defect	2020	12	260071	26	10.00 (6.53-14.65) [/100,000]
Limb reduction defect	2021	1	246479	26	10.55 (6.89-15.46) [/100,000]
Limb reduction defect	2021	2	238352	14	5.87 (3.21-9.85) [/100,000]
Limb reduction defect	2021	3	271449	33	12.16 (8.37-17.07) [/100,000]
Limb reduction defect	2021	4	261970	27	10.31 (6.79-15.00) [/100,000]
Limb reduction defect	2021	5	269110	21	7.80 (4.83-11.93) [/100,000]
Limb reduction defect	2021	6	280679	34	12.11 (8.39-16.93) [/100,000]
Limb reduction defect	2021	7	291919	23	7.88 (4.99-11.82) [/100,000]
Limb reduction defect	2021	8	295288	32	10.84 (7.41-15.30) [/100,000]
Limb reduction defect	2021	9	292566	27	9.23 (6.08-13.43) [/100,000]
Limb reduction defect	2021	10	281977	33	11.70 (8.06-16.44) [/100,000]
Limb reduction defect	2021	11	270183	25	9.25 (5.99-13.66) [/100,000]
Limb reduction defect	2021	12	277304	25	9.02 (5.83-13.31) [/100,000]
Limb reduction defect	2022	1	262670	28	10.66 (7.08-15.41) [/100,000]
Limb reduction defect	2022	2	247298	27	10.92 (7.20-15.88) [/100,000]
Limb reduction defect	2022	3	276060	30	10.87 (7.33-15.51) [/100,000]
Limb reduction defect	2022	4	258699	25	9.66 (6.25-14.27) [/100,000]
Limb reduction defect	2022	5	266100	30	11.27 (7.61-16.09) [/100,000]
Limb reduction defect	2022	6	273442	25	9.14 (5.92-13.50) [/100,000]
Limb reduction defect	2022	7	287161	30	10.45 (7.05-14.91) [/100,000]
Limb reduction defect	2022	8	303036	29	9.57 (6.41-13.74) [/100,000]
Limb reduction defect	2022	9	281272	24	8.53 (5.47-12.70) [/100,000]
Limb reduction defect	2022	10	274216	25	9.12 (5.90-13.46) [/100,000]
Limb reduction defect	2022	11	275054	23	8.36 (5.30-12.55) [/100,000]
Limb reduction defect	2022	12	281653	31	11.01 (7.48-15.62) [/100,000]
Cleft Lip with or without Cleft Palate	2016	1	285262	141	49.43 (41.61-58.29) [/100,000]
Cleft Lip with or without Cleft Palate	2016	2	276149	155	56.13 (47.64-65.69) [/100,000]
Cleft Lip with or without Cleft Palate	2016	3	296302	138	46.57 (39.13-55.02) [/100,000]
Cleft Lip with or without Cleft Palate	2016	4	282255	144	51.02 (43.03-60.06) [/100,000]
Cleft Lip with or without Cleft Palate	2016	5	295006	145	49.15 (41.48-57.83) [/100,000]
Cleft Lip with or without Cleft Palate	2016	6	299365	176	58.79 (50.43-68.14) [/100,000]
Cleft Lip with or without Cleft Palate	2016	7	308619	150	48.60 (41.14-57.03) [/100,000]
Cleft Lip with or without Cleft Palate	2016	8	323786	160	49.42 (42.06-57.69) [/100,000]
Cleft Lip with or without Cleft Palate	2016	9	313643	132	42.09 (35.21-49.91) [/100,000]
Cleft Lip with or without Cleft Palate	2016	10	298695	131	43.86 (36.67-52.04) [/100,000]
Cleft Lip with or without Cleft Palate	2016	11	288188	144	49.97 (42.14-58.82) [/100,000]
Cleft Lip with or without Cleft Palate	2016	12	291726	128	43.88 (36.61-52.17) [/100,000]
Cleft Lip with or without Cleft Palate	2017	1	282016	125	44.32 (36.90-52.81) [/100,000]
Cleft Lip with or without Cleft Palate	2017	2	260712	131	50.25 (42.01-59.62) [/100,000]
Cleft Lip with or without Cleft Palate	2017	3	288281	152	52.73 (44.68-61.80) [/100,000]
Cleft Lip with or without Cleft Palate	2017	4	270374	121	44.75 (37.14-53.47) [/100,000]
Cleft Lip with or without Cleft Palate	2017	5	290447	160	55.09 (46.88-64.31) [/100,000]
Cleft Lip with or without Cleft Palate	2017	6	291352	131	44.96 (37.59-53.35) [/100,000]
Cleft Lip with or without Cleft Palate	2017	7	301469	154	51.08 (43.34-59.82) [/100,000]
Cleft Lip with or without Cleft Palate	2017	8	318057	141	44.33 (37.32-52.28) [/100,000]
Cleft Lip with or without Cleft Palate	2017	9	305500	137	44.84 (37.65-53.01) [/100,000]
Cleft Lip with or without Cleft Palate	2017	10	297762	133	44.67 (37.40-52.93) [/100,000]
Cleft Lip with or without Cleft Palate	2017	11	285506	111	38.88 (31.98-46.82) [/100,000]
Cleft Lip with or without Cleft Palate	2017	12	283909	134	47.20 (39.55-55.90) [/100,000]
Cleft Lip with or without Cleft Palate	2018	1	282988	149	52.65 (44.54-61.82) [/100,000]
Cleft Lip with or without Cleft Palate	2018	2	256322	119	46.43 (38.46-55.55) [/100,000]
Cleft Lip with or without Cleft Palate	2018	3	285104	167	58.58 (50.03-68.16) [/100,000]
Cleft Lip with or without Cleft Palate	2018	4	268820	138	51.34 (43.13-60.65) [/100,000]
Cleft Lip with or without Cleft Palate	2018	5	288175	151	52.40 (44.38-61.45) [/100,000]
Cleft Lip with or without Cleft Palate	2018	6	282960	126	44.53 (37.10-53.02) [/100,000]
Cleft Lip with or without Cleft Palate	2018	7	295835	143	48.34 (40.74-56.94) [/100,000]
Cleft Lip with or without Cleft Palate	2018	8	311337	159	51.07 (43.44-59.65) [/100,000]
Cleft Lip with or without Cleft Palate	2018	9	291771	130	44.56 (37.23-52.90) [/100,000]
Cleft Lip with or without Cleft Palate	2018	10	294411	143	48.57 (40.94-57.21) [/100,000]

Cleft Lip with or without Cleft Palate	2018	11	277618	124	44.67 (37.15-53.25) [/100,000]
Cleft Lip with or without Cleft Palate	2018	12	278997	150	53.76 (45.51-63.09) [/100,000]
Cleft Lip with or without Cleft Palate	2019	1	279333	130	46.54 (38.88-55.26) [/100,000]
Cleft Lip with or without Cleft Palate	2019	2	251637	127	50.47 (42.08-60.05) [/100,000]
Cleft Lip with or without Cleft Palate	2019	3	273087	133	48.70 (40.78-57.72) [/100,000]
Cleft Lip with or without Cleft Palate	2019	4	268825	122	45.38 (37.69-54.18) [/100,000]
Cleft Lip with or without Cleft Palate	2019	5	283937	126	44.38 (36.97-52.83) [/100,000]
Cleft Lip with or without Cleft Palate	2019	6	272242	122	44.81 (37.22-53.50) [/100,000]
Cleft Lip with or without Cleft Palate	2019	7	299580	121	40.39 (33.52-48.26) [/100,000]
Cleft Lip with or without Cleft Palate	2019	8	307625	135	43.88 (36.80-51.94) [/100,000]
Cleft Lip with or without Cleft Palate	2019	9	294673	135	45.81 (38.41-54.22) [/100,000]
Cleft Lip with or without Cleft Palate	2019	10	292702	113	38.61 (31.82-46.41) [/100,000]
Cleft Lip with or without Cleft Palate	2019	11	267192	114	42.67 (35.20-51.25) [/100,000]
Cleft Lip with or without Cleft Palate	2019	12	276844	119	42.98 (35.61-51.44) [/100,000]
Cleft Lip with or without Cleft Palate	2020	1	272987	151	55.31 (46.85-64.87) [/100,000]
Cleft Lip with or without Cleft Palate	2020	2	253500	115	45.36 (37.45-54.45) [/100,000]
Cleft Lip with or without Cleft Palate	2020	3	272222	106	38.94 (31.88-47.09) [/100,000]
Cleft Lip with or without Cleft Palate	2020	4	261951	99	37.79 (30.72-46.01) [/100,000]
Cleft Lip with or without Cleft Palate	2020	5	270970	128	47.24 (39.41-56.16) [/100,000]
Cleft Lip with or without Cleft Palate	2020	6	271183	127	46.83 (39.04-55.72) [/100,000]
Cleft Lip with or without Cleft Palate	2020	7	288818	127	43.97 (36.66-52.32) [/100,000]
Cleft Lip with or without Cleft Palate	2020	8	287391	125	43.49 (36.21-51.82) [/100,000]
Cleft Lip with or without Cleft Palate	2020	9	281560	129	45.82 (38.25-54.44) [/100,000]
Cleft Lip with or without Cleft Palate	2020	10	273929	139	50.74 (42.66-59.91) [/100,000]
Cleft Lip with or without Cleft Palate	2020	11	254215	99	38.94 (31.65-47.41) [/100,000]
Cleft Lip with or without Cleft Palate	2020	12	260071	128	49.22 (41.06-58.52) [/100,000]
Cleft Lip with or without Cleft Palate	2021	1	246479	127	51.53 (42.96-61.30) [/100,000]
Cleft Lip with or without Cleft Palate	2021	2	238352	111	46.57 (38.31-56.08) [/100,000]
Cleft Lip with or without Cleft Palate	2021	3	271449	148	54.52 (46.09-64.04) [/100,000]
Cleft Lip with or without Cleft Palate	2021	4	261970	129	49.24 (41.11-58.51) [/100,000]
Cleft Lip with or without Cleft Palate	2021	5	269110	122	45.33 (37.65-54.13) [/100,000]
Cleft Lip with or without Cleft Palate	2021	6	280679	111	39.55 (32.53-47.62) [/100,000]
Cleft Lip with or without Cleft Palate	2021	7	291919	136	46.59 (39.09-55.11) [/100,000]
Cleft Lip with or without Cleft Palate	2021	8	295288	146	49.44 (41.75-58.14) [/100,000]
Cleft Lip with or without Cleft Palate	2021	9	292566	130	44.43 (37.13-52.76) [/100,000]
Cleft Lip with or without Cleft Palate	2021	10	281977	139	49.29 (41.44-58.20) [/100,000]
Cleft Lip with or without Cleft Palate	2021	11	270183	121	44.78 (37.16-53.51) [/100,000]
Cleft Lip with or without Cleft Palate	2021	12	277304	138	49.76 (41.81-58.79) [/100,000]
Cleft Lip with or without Cleft Palate	2022	1	262670	106	40.35 (33.04-48.81) [/100,000]
Cleft Lip with or without Cleft Palate	2022	2	247298	120	48.52 (40.23-58.02) [/100,000]
Cleft Lip with or without Cleft Palate	2022	3	276060	117	42.38 (35.05-50.79) [/100,000]
Cleft Lip with or without Cleft Palate	2022	4	258699	122	47.16 (39.16-56.31) [/100,000]
Cleft Lip with or without Cleft Palate	2022	5	266100	124	46.60 (38.76-55.56) [/100,000]
Cleft Lip with or without Cleft Palate	2022	6	273442	133	48.64 (40.73-57.64) [/100,000]
Cleft Lip with or without Cleft Palate	2022	7	287161	134	46.66 (39.10-55.26) [/100,000]
Cleft Lip with or without Cleft Palate	2022	8	303036	148	48.84 (41.29-57.37) [/100,000]
Cleft Lip with or without Cleft Palate	2022	9	281272	144	51.20 (43.18-60.27) [/100,000]
Cleft Lip with or without Cleft Palate	2022	10	274216	115	41.94 (34.63-50.34) [/100,000]
Cleft Lip with or without Cleft Palate	2022	11	275054	128	46.54 (38.83-55.33) [/100,000]
Cleft Lip with or without Cleft Palate	2022	12	281653	146	51.84 (43.77-60.96) [/100,000]
Cleft Palate alone	2016	1	285262	66	23.14 (17.89-29.43) [/100,000]
Cleft Palate alone	2016	2	276149	62	22.45 (17.21-28.78) [/100,000]
Cleft Palate alone	2016	3	296302	60	20.25 (15.45-26.06) [/100,000]
Cleft Palate alone	2016	4	282255	56	19.84 (14.99-25.76) [/100,000]
Cleft Palate alone	2016	5	295006	68	23.05 (17.90-29.22) [/100,000]
Cleft Palate alone	2016	6	299365	61	20.38 (15.59-26.17) [/100,000]
Cleft Palate alone	2016	7	308619	74	23.98 (18.83-30.10) [/100,000]
Cleft Palate alone	2016	8	323786	71	21.93 (17.13-27.66) [/100,000]
Cleft Palate alone	2016	9	313643	60	19.13 (14.60-24.62) [/100,000]
Cleft Palate alone	2016	10	298695	65	21.76 (16.80-27.74) [/100,000]
Cleft Palate alone	2016	11	288188	62	21.51 (16.49-27.58) [/100,000]
Cleft Palate alone	2016	12	291726	67	22.97 (17.80-29.17) [/100,000]
Cleft Palate alone	2017	1	282016	50	17.73 (13.16-23.37) [/100,000]
Cleft Palate alone	2017	2	260712	65	24.93 (19.24-31.78) [/100,000]
Cleft Palate alone	2017	3	288281	55	19.08 (14.37-24.83) [/100,000]
Cleft Palate alone	2017	4	270374	76	28.11 (22.15-35.18) [/100,000]
Cleft Palate alone	2017	5	290447	54	18.59 (13.97-24.26) [/100,000]
Cleft Palate alone	2017	6	291352	51	17.50 (13.03-23.01) [/100,000]
Cleft Palate alone	2017	7	301469	67	22.22 (17.22-28.22) [/100,000]
Cleft Palate alone	2017	8	318057	73	22.95 (17.99-28.86) [/100,000]
Cleft Palate alone	2017	9	305500	75	24.55 (19.31-30.77) [/100,000]

Cleft Palate alone	2017	10	297762	62	20.82 (15.96-26.69) [/100,000]
Cleft Palate alone	2017	11	285506	59	20.67 (15.73-26.66) [/100,000]
Cleft Palate alone	2017	12	283909	67	23.60 (18.29-29.97) [/100,000]
Cleft Palate alone	2018	1	282988	62	21.91 (16.80-28.09) [/100,000]
Cleft Palate alone	2018	2	256322	61	23.80 (18.20-30.57) [/100,000]
Cleft Palate alone	2018	3	285104	73	25.60 (20.07-32.19) [/100,000]
Cleft Palate alone	2018	4	268820	47	17.48 (12.85-23.25) [/100,000]
Cleft Palate alone	2018	5	288175	59	20.47 (15.59-26.41) [/100,000]
Cleft Palate alone	2018	6	282960	59	20.85 (15.87-26.90) [/100,000]
Cleft Palate alone	2018	7	295835	67	22.65 (17.55-28.76) [/100,000]
Cleft Palate alone	2018	8	311337	72	23.13 (18.10-29.12) [/100,000]
Cleft Palate alone	2018	9	291771	62	21.25 (16.29-27.24) [/100,000]
Cleft Palate alone	2018	10	294411	58	19.70 (14.96-25.47) [/100,000]
Cleft Palate alone	2018	11	277618	57	20.53 (15.55-26.60) [/100,000]
Cleft Palate alone	2018	12	278997	70	25.09 (19.56-31.70) [/100,000]
Cleft Palate alone	2019	1	279333	53	18.97 (14.21-24.82) [/100,000]
Cleft Palate alone	2019	2	251637	54	21.46 (16.12-28.00) [/100,000]
Cleft Palate alone	2019	3	273087	61	22.34 (17.09-28.69) [/100,000]
Cleft Palate alone	2019	4	268825	54	20.09 (15.09-26.21) [/100,000]
Cleft Palate alone	2019	5	283937	70	24.65 (19.22-31.15) [/100,000]
Cleft Palate alone	2019	6	272242	54	19.84 (14.90-25.88) [/100,000]
Cleft Palate alone	2019	7	299580	59	19.69 (14.99-25.40) [/100,000]
Cleft Palate alone	2019	8	307625	67	21.78 (16.88-27.66) [/100,000]
Cleft Palate alone	2019	9	294673	61	20.70 (15.83-26.59) [/100,000]
Cleft Palate alone	2019	10	292702	58	19.82 (15.05-25.62) [/100,000]
Cleft Palate alone	2019	11	267192	62	23.20 (17.79-29.75) [/100,000]
Cleft Palate alone	2019	12	276844	66	23.84 (18.44-30.33) [/100,000]
Cleft Palate alone	2020	1	272987	67	24.54 (19.02-31.17) [/100,000]
Cleft Palate alone	2020	2	253500	50	19.72 (14.64-26.00) [/100,000]
Cleft Palate alone	2020	3	272222	64	23.51 (18.11-30.02) [/100,000]
Cleft Palate alone	2020	4	261951	63	24.05 (18.48-30.77) [/100,000]
Cleft Palate alone	2020	5	270970	61	22.51 (17.22-28.92) [/100,000]
Cleft Palate alone	2020	6	271183	73	26.92 (21.10-33.85) [/100,000]
Cleft Palate alone	2020	7	288818	59	20.43 (15.55-26.35) [/100,000]
Cleft Palate alone	2020	8	287391	52	18.09 (13.51-23.73) [/100,000]
Cleft Palate alone	2020	9	281560	66	23.44 (18.13-29.82) [/100,000]
Cleft Palate alone	2020	10	273929	56	20.44 (15.44-26.55) [/100,000]
Cleft Palate alone	2020	11	254215	54	21.24 (15.96-27.72) [/100,000]
Cleft Palate alone	2020	12	260071	42	16.15 (11.64-21.83) [/100,000]
Cleft Palate alone	2021	1	246479	50	20.29 (15.06-26.74) [/100,000]
Cleft Palate alone	2021	2	238352	54	22.66 (17.02-29.56) [/100,000]
Cleft Palate alone	2021	3	271449	65	23.95 (18.48-30.52) [/100,000]
Cleft Palate alone	2021	4	261970	64	24.43 (18.81-31.20) [/100,000]
Cleft Palate alone	2021	5	269110	53	19.69 (14.75-25.76) [/100,000]
Cleft Palate alone	2021	6	280679	55	19.60 (14.76-25.51) [/100,000]
Cleft Palate alone	2021	7	291919	63	21.58 (16.58-27.61) [/100,000]
Cleft Palate alone	2021	8	295288	60	20.32 (15.51-26.15) [/100,000]
Cleft Palate alone	2021	9	292566	45	15.38 (11.22-20.58) [/100,000]
Cleft Palate alone	2021	10	281977	62	21.99 (16.86-28.19) [/100,000]
Cleft Palate alone	2021	11	270183	72	26.65 (20.85-33.56) [/100,000]
Cleft Palate alone	2021	12	277304	54	19.47 (14.63-25.41) [/100,000]
Cleft Palate alone	2022	1	262670	53	20.18 (15.11-26.39) [/100,000]
Cleft Palate alone	2022	2	247298	54	21.84 (16.40-28.49) [/100,000]
Cleft Palate alone	2022	3	276060	45	16.30 (11.89-21.81) [/100,000]
Cleft Palate alone	2022	4	258699	55	21.26 (16.02-27.67) [/100,000]
Cleft Palate alone	2022	5	266100	49	18.41 (13.62-24.34) [/100,000]
Cleft Palate alone	2022	6	273442	56	20.48 (15.47-26.59) [/100,000]
Cleft Palate alone	2022	7	287161	65	22.64 (17.47-28.85) [/100,000]
Cleft Palate alone	2022	8	303036	57	18.81 (14.25-24.37) [/100,000]
Cleft Palate alone	2022	9	281272	67	23.82 (18.46-30.25) [/100,000]
Cleft Palate alone	2022	10	274216	64	23.34 (17.97-29.80) [/100,000]
Cleft Palate alone	2022	11	275054	46	16.72 (12.24-22.31) [/100,000]
Cleft Palate alone	2022	12	281653	64	22.72 (17.50-29.02) [/100,000]
Down syndrome	2016	1	285262	119	41.72 (34.56-49.92) [/100,000]
Down syndrome	2016	2	276149	125	45.27 (37.68-53.93) [/100,000]
Down syndrome	2016	3	296302	139	46.91 (39.44-55.39) [/100,000]
Down syndrome	2016	4	282255	126	44.64 (37.19-53.15) [/100,000]
Down syndrome	2016	5	295006	133	45.08 (37.75-53.43) [/100,000]
Down syndrome	2016	6	299365	120	40.08 (33.24-47.93) [/100,000]
Down syndrome	2016	7	308619	113	36.61 (30.18-44.02) [/100,000]
Down syndrome	2016	8	323786	152	46.94 (39.78-55.03) [/100,000]

Down syndrome	2016	9	313643	145	46.23 (39.01-54.40) [/100,000]
Down syndrome	2016	10	298695	130	43.52 (36.36-51.68) [/100,000]
Down syndrome	2016	11	288188	133	46.15 (38.64-54.69) [/100,000]
Down syndrome	2016	12	291726	126	43.19 (35.98-51.42) [/100,000]
Down syndrome	2017	1	282016	113	40.07 (33.02-48.17) [/100,000]
Down syndrome	2017	2	260712	111	42.58 (35.03-51.27) [/100,000]
Down syndrome	2017	3	288281	126	43.71 (36.41-52.04) [/100,000]
Down syndrome	2017	4	270374	108	39.94 (32.77-48.22) [/100,000]
Down syndrome	2017	5	290447	117	40.28 (33.32-48.28) [/100,000]
Down syndrome	2017	6	291352	145	49.77 (42.00-58.56) [/100,000]
Down syndrome	2017	7	301469	114	37.81 (31.19-45.43) [/100,000]
Down syndrome	2017	8	318057	141	44.33 (37.32-52.28) [/100,000]
Down syndrome	2017	9	305500	123	40.26 (33.46-48.04) [/100,000]
Down syndrome	2017	10	297762	124	41.64 (34.64-49.65) [/100,000]
Down syndrome	2017	11	285506	115	40.28 (33.26-48.35) [/100,000]
Down syndrome	2017	12	283909	124	43.68 (36.33-52.07) [/100,000]
Down syndrome	2018	1	282988	128	45.23 (37.74-53.78) [/100,000]
Down syndrome	2018	2	256322	143	55.79 (47.02-65.72) [/100,000]
Down syndrome	2018	3	285104	123	43.14 (35.86-51.47) [/100,000]
Down syndrome	2018	4	268820	121	45.01 (37.35-53.78) [/100,000]
Down syndrome	2018	5	288175	108	37.48 (30.74-45.25) [/100,000]
Down syndrome	2018	6	282960	127	44.88 (37.42-53.40) [/100,000]
Down syndrome	2018	7	295835	123	41.58 (34.56-49.61) [/100,000]
Down syndrome	2018	8	311337	134	43.04 (36.06-50.97) [/100,000]
Down syndrome	2018	9	291771	137	46.95 (39.42-55.51) [/100,000]
Down syndrome	2018	10	294411	158	53.67 (45.63-62.71) [/100,000]
Down syndrome	2018	11	277618	114	41.06 (33.87-49.33) [/100,000]
Down syndrome	2018	12	278997	113	40.50 (33.38-48.69) [/100,000]
Down syndrome	2019	1	279333	116	41.53 (34.32-49.81) [/100,000]
Down syndrome	2019	2	251637	120	47.69 (39.54-57.02) [/100,000]
Down syndrome	2019	3	273087	127	46.51 (38.77-55.33) [/100,000]
Down syndrome	2019	4	268825	103	38.31 (31.27-46.47) [/100,000]
Down syndrome	2019	5	283937	112	39.45 (32.48-47.46) [/100,000]
Down syndrome	2019	6	272242	111	40.77 (33.54-49.10) [/100,000]
Down syndrome	2019	7	299580	115	38.39 (31.69-46.08) [/100,000]
Down syndrome	2019	8	307625	135	43.88 (36.80-51.94) [/100,000]
Down syndrome	2019	9	294673	138	46.83 (39.35-55.33) [/100,000]
Down syndrome	2019	10	292702	126	43.05 (35.86-51.25) [/100,000]
Down syndrome	2019	11	267192	143	53.52 (45.11-63.04) [/100,000]
Down syndrome	2019	12	276844	123	44.43 (36.93-53.01) [/100,000]
Down syndrome	2020	1	272987	121	44.32 (36.78-52.96) [/100,000]
Down syndrome	2020	2	253500	123	48.52 (40.33-57.89) [/100,000]
Down syndrome	2020	3	272222	98	36.00 (29.23-43.87) [/100,000]
Down syndrome	2020	4	261951	102	38.94 (31.75-47.27) [/100,000]
Down syndrome	2020	5	270970	117	43.18 (35.71-51.75) [/100,000]
Down syndrome	2020	6	271183	119	43.88 (36.35-52.51) [/100,000]
Down syndrome	2020	7	288818	114	39.47 (32.56-47.42) [/100,000]
Down syndrome	2020	8	287391	135	46.97 (39.39-55.60) [/100,000]
Down syndrome	2020	9	281560	111	39.42 (32.43-47.47) [/100,000]
Down syndrome	2020	10	273929	109	39.79 (32.67-48.00) [/100,000]
Down syndrome	2020	11	254215	126	49.56 (41.29-59.01) [/100,000]
Down syndrome	2020	12	260071	95	36.53 (29.55-44.65) [/100,000]
Down syndrome	2021	1	246479	110	44.63 (36.68-53.79) [/100,000]
Down syndrome	2021	2	238352	99	41.54 (33.76-50.57) [/100,000]
Down syndrome	2021	3	271449	106	39.05 (31.97-47.23) [/100,000]
Down syndrome	2021	4	261970	109	41.61 (34.17-50.19) [/100,000]
Down syndrome	2021	5	269110	106	39.39 (32.25-47.64) [/100,000]
Down syndrome	2021	6	280679	116	41.33 (34.15-49.57) [/100,000]
Down syndrome	2021	7	291919	98	33.57 (27.26-40.91) [/100,000]
Down syndrome	2021	8	295288	112	37.93 (31.23-45.64) [/100,000]
Down syndrome	2021	9	292566	127	43.41 (36.19-51.65) [/100,000]
Down syndrome	2021	10	281977	120	42.56 (35.28-50.89) [/100,000]
Down syndrome	2021	11	270183	105	38.86 (31.79-47.04) [/100,000]
Down syndrome	2021	12	277304	112	40.39 (33.26-48.60) [/100,000]
Down syndrome	2022	1	262670	115	43.78 (36.15-52.55) [/100,000]
Down syndrome	2022	2	247298	117	47.31 (39.13-56.70) [/100,000]
Down syndrome	2022	3	276060	108	39.12 (32.09-47.23) [/100,000]
Down syndrome	2022	4	258699	85	32.86 (26.25-40.63) [/100,000]
Down syndrome	2022	5	266100	113	42.47 (35.00-51.05) [/100,000]
Down syndrome	2022	6	273442	115	42.06 (34.72-50.48) [/100,000]
Down syndrome	2022	7	287161	143	49.80 (41.97-58.66) [/100,000]

Down syndrome	2022	8	303036	124	40.92 (34.04-48.79) [/100,000]
Down syndrome	2022	9	281272	102	36.26 (29.57-44.02) [/100,000]
Down syndrome	2022	10	274216	104	37.93 (30.99-45.95) [/100,000]
Down syndrome	2022	11	275054	100	36.36 (29.58-44.22) [/100,000]
Down syndrome	2022	12	281653	99	35.15 (28.57-42.79) [/100,000]
Suspected chromosomal disorder	2016	1	285262	94	32.95 (26.63-40.32) [/100,000]
Suspected chromosomal disorder	2016	2	276149	88	31.87 (25.56-39.26) [/100,000]
Suspected chromosomal disorder	2016	3	296302	100	33.75 (27.46-41.05) [/100,000]
Suspected chromosomal disorder	2016	4	282255	88	31.18 (25.01-38.41) [/100,000]
Suspected chromosomal disorder	2016	5	295006	79	26.78 (21.20-33.37) [/100,000]
Suspected chromosomal disorder	2016	6	299365	89	29.73 (23.88-36.58) [/100,000]
Suspected chromosomal disorder	2016	7	308619	107	34.67 (28.41-41.89) [/100,000]
Suspected chromosomal disorder	2016	8	323786	89	27.49 (22.08-33.82) [/100,000]
Suspected chromosomal disorder	2016	9	313643	105	33.48 (27.38-40.53) [/100,000]
Suspected chromosomal disorder	2016	10	298695	108	36.16 (29.66-43.65) [/100,000]
Suspected chromosomal disorder	2016	11	288188	90	31.23 (25.11-38.39) [/100,000]
Suspected chromosomal disorder	2016	12	291726	77	26.39 (20.83-32.99) [/100,000]
Suspected chromosomal disorder	2017	1	282016	85	30.14 (24.08-37.27) [/100,000]
Suspected chromosomal disorder	2017	2	260712	90	34.52 (27.76-42.43) [/100,000]
Suspected chromosomal disorder	2017	3	288281	93	32.26 (26.04-39.52) [/100,000]
Suspected chromosomal disorder	2017	4	270374	100	36.99 (30.09-44.98) [/100,000]
Suspected chromosomal disorder	2017	5	290447	90	30.99 (24.92-38.09) [/100,000]
Suspected chromosomal disorder	2017	6	291352	99	33.98 (27.62-41.37) [/100,000]
Suspected chromosomal disorder	2017	7	301469	81	26.87 (21.34-33.39) [/100,000]
Suspected chromosomal disorder	2017	8	318057	85	26.72 (21.35-33.04) [/100,000]
Suspected chromosomal disorder	2017	9	305500	104	34.04 (27.82-41.25) [/100,000]
Suspected chromosomal disorder	2017	10	297762	110	36.94 (30.36-44.52) [/100,000]
Suspected chromosomal disorder	2017	11	285506	82	28.72 (22.84-35.65) [/100,000]
Suspected chromosomal disorder	2017	12	283909	93	32.76 (26.44-40.13) [/100,000]
Suspected chromosomal disorder	2018	1	282988	105	37.10 (30.35-44.91) [/100,000]
Suspected chromosomal disorder	2018	2	256322	87	33.94 (27.19-41.87) [/100,000]
Suspected chromosomal disorder	2018	3	285104	93	32.62 (26.33-39.96) [/100,000]
Suspected chromosomal disorder	2018	4	268820	83	30.88 (24.59-38.27) [/100,000]
Suspected chromosomal disorder	2018	5	288175	89	30.88 (24.80-38.00) [/100,000]
Suspected chromosomal disorder	2018	6	282960	101	35.69 (29.07-43.37) [/100,000]
Suspected chromosomal disorder	2018	7	295835	106	35.83 (29.34-43.33) [/100,000]
Suspected chromosomal disorder	2018	8	311337	105	33.73 (27.58-40.83) [/100,000]
Suspected chromosomal disorder	2018	9	291771	96	32.90 (26.65-40.18) [/100,000]
Suspected chromosomal disorder	2018	10	294411	120	40.76 (33.79-48.74) [/100,000]
Suspected chromosomal disorder	2018	11	277618	91	32.78 (26.39-40.24) [/100,000]
Suspected chromosomal disorder	2018	12	278997	82	29.39 (23.38-36.48) [/100,000]
Suspected chromosomal disorder	2019	1	279333	103	36.87 (30.10-44.72) [/100,000]
Suspected chromosomal disorder	2019	2	251637	105	41.73 (34.13-50.51) [/100,000]
Suspected chromosomal disorder	2019	3	273087	79	28.93 (22.90-36.05) [/100,000]
Suspected chromosomal disorder	2019	4	268825	90	33.48 (26.92-41.15) [/100,000]
Suspected chromosomal disorder	2019	5	283937	90	31.70 (25.49-38.96) [/100,000]
Suspected chromosomal disorder	2019	6	272242	91	33.43 (26.91-41.04) [/100,000]
Suspected chromosomal disorder	2019	7	299580	100	33.38 (27.16-40.60) [/100,000]
Suspected chromosomal disorder	2019	8	307625	96	31.21 (25.28-38.11) [/100,000]
Suspected chromosomal disorder	2019	9	294673	99	33.60 (27.31-40.90) [/100,000]
Suspected chromosomal disorder	2019	10	292702	90	30.75 (24.73-37.79) [/100,000]
Suspected chromosomal disorder	2019	11	267192	83	31.06 (24.74-38.51) [/100,000]
Suspected chromosomal disorder	2019	12	276844	105	37.93 (31.02-45.91) [/100,000]
Suspected chromosomal disorder	2020	1	272987	109	39.93 (32.79-48.16) [/100,000]
Suspected chromosomal disorder	2020	2	253500	96	37.87 (30.68-46.24) [/100,000]
Suspected chromosomal disorder	2020	3	272222	84	30.86 (24.61-38.20) [/100,000]
Suspected chromosomal disorder	2020	4	261951	69	26.34 (20.50-33.33) [/100,000]
Suspected chromosomal disorder	2020	5	270970	93	34.32 (27.70-42.04) [/100,000]
Suspected chromosomal disorder	2020	6	271183	115	42.41 (35.01-50.90) [/100,000]
Suspected chromosomal disorder	2020	7	288818	101	34.97 (28.48-42.49) [/100,000]
Suspected chromosomal disorder	2020	8	287391	101	35.14 (28.63-42.70) [/100,000]
Suspected chromosomal disorder	2020	9	281560	89	31.61 (25.39-38.90) [/100,000]
Suspected chromosomal disorder	2020	10	273929	100	36.51 (29.70-44.40) [/100,000]
Suspected chromosomal disorder	2020	11	254215	88	34.62 (27.76-42.65) [/100,000]
Suspected chromosomal disorder	2020	12	260071	89	34.22 (27.48-42.11) [/100,000]
Suspected chromosomal disorder	2021	1	246479	80	32.46 (25.74-40.39) [/100,000]
Suspected chromosomal disorder	2021	2	238352	102	42.79 (34.89-51.95) [/100,000]
Suspected chromosomal disorder	2021	3	271449	119	43.84 (36.32-52.46) [/100,000]
Suspected chromosomal disorder	2021	4	261970	99	37.79 (30.72-46.01) [/100,000]
Suspected chromosomal disorder	2021	5	269110	96	35.67 (28.90-43.56) [/100,000]
Suspected chromosomal disorder	2021	6	280679	113	40.26 (33.18-48.40) [/100,000]

Suspected chromosomal disorder	2021	7	291919	115	39.39 (32.53-47.29) [/100,000]
Suspected chromosomal disorder	2021	8	295288	97	32.85 (26.64-40.07) [/100,000]
Suspected chromosomal disorder	2021	9	292566	84	28.71 (22.90-35.55) [/100,000]
Suspected chromosomal disorder	2021	10	281977	102	36.17 (29.50-43.91) [/100,000]
Suspected chromosomal disorder	2021	11	270183	84	31.09 (24.80-38.49) [/100,000]
Suspected chromosomal disorder	2021	12	277304	91	32.82 (26.42-40.29) [/100,000]
Suspected chromosomal disorder	2022	1	262670	91	34.64 (27.89-42.53) [/100,000]
Suspected chromosomal disorder	2022	2	247298	99	40.03 (32.54-48.74) [/100,000]
Suspected chromosomal disorder	2022	3	276060	81	29.34 (23.30-36.47) [/100,000]
Suspected chromosomal disorder	2022	4	258699	88	34.02 (27.28-41.91) [/100,000]
Suspected chromosomal disorder	2022	5	266100	86	32.32 (25.85-39.91) [/100,000]
Suspected chromosomal disorder	2022	6	273442	103	37.67 (30.75-45.68) [/100,000]
Suspected chromosomal disorder	2022	7	287161	87	30.30 (24.27-37.37) [/100,000]
Suspected chromosomal disorder	2022	8	303036	95	31.35 (25.36-38.32) [/100,000]
Suspected chromosomal disorder	2022	9	281272	102	36.26 (29.57-44.02) [/100,000]
Suspected chromosomal disorder	2022	10	274216	102	37.20 (30.33-45.15) [/100,000]
Suspected chromosomal disorder	2022	11	275054	103	37.45 (30.57-45.41) [/100,000]
Suspected chromosomal disorder	2022	12	281653	74	26.27 (20.63-32.98) [/100,000]
Hypospadias	2016	1	285262	155	54.34 (46.12-63.59) [/100,000]
Hypospadias	2016	2	276149	168	60.84 (51.99-70.76) [/100,000]
Hypospadias	2016	3	296302	186	62.77 (54.08-72.47) [/100,000]
Hypospadias	2016	4	282255	162	57.39 (48.90-66.94) [/100,000]
Hypospadias	2016	5	295006	183	62.03 (53.37-71.70) [/100,000]
Hypospadias	2016	6	299365	149	49.77 (42.10-58.43) [/100,000]
Hypospadias	2016	7	308619	157	50.87 (43.23-59.48) [/100,000]
Hypospadias	2016	8	323786	190	58.68 (50.64-67.64) [/100,000]
Hypospadias	2016	9	313643	165	52.61 (44.89-61.27) [/100,000]
Hypospadias	2016	10	298695	171	57.25 (48.99-66.50) [/100,000]
Hypospadias	2016	11	288188	166	57.60 (49.17-67.06) [/100,000]
Hypospadias	2016	12	291726	139	47.65 (40.06-56.26) [/100,000]
Hypospadias	2017	1	282016	158	56.03 (47.63-65.47) [/100,000]
Hypospadias	2017	2	260712	154	59.07 (50.11-69.17) [/100,000]
Hypospadias	2017	3	288281	135	46.83 (39.26-55.43) [/100,000]
Hypospadias	2017	4	270374	160	59.18 (50.37-69.09) [/100,000]
Hypospadias	2017	5	290447	173	59.56 (51.02-69.13) [/100,000]
Hypospadias	2017	6	291352	165	56.63 (48.32-65.96) [/100,000]
Hypospadias	2017	7	301469	155	51.41 (43.64-60.17) [/100,000]
Hypospadias	2017	8	318057	196	61.62 (53.30-70.88) [/100,000]
Hypospadias	2017	9	305500	150	49.10 (41.56-57.61) [/100,000]
Hypospadias	2017	10	297762	146	49.03 (41.40-57.66) [/100,000]
Hypospadias	2017	11	285506	157	54.99 (46.73-64.29) [/100,000]
Hypospadias	2017	12	283909	154	54.24 (46.02-63.52) [/100,000]
Hypospadias	2018	1	282988	137	48.41 (40.65-57.23) [/100,000]
Hypospadias	2018	2	256322	148	57.74 (48.81-67.82) [/100,000]
Hypospadias	2018	3	285104	144	50.51 (42.60-59.46) [/100,000]
Hypospadias	2018	4	268820	154	57.29 (48.60-67.08) [/100,000]
Hypospadias	2018	5	288175	152	52.75 (44.70-61.83) [/100,000]
Hypospadias	2018	6	282960	143	50.54 (42.60-59.53) [/100,000]
Hypospadias	2018	7	295835	166	56.11 (47.90-65.32) [/100,000]
Hypospadias	2018	8	311337	156	50.11 (42.55-58.61) [/100,000]
Hypospadias	2018	9	291771	172	58.95 (50.47-68.45) [/100,000]
Hypospadias	2018	10	294411	178	60.46 (51.91-70.02) [/100,000]
Hypospadias	2018	11	277618	143	51.51 (43.42-60.67) [/100,000]
Hypospadias	2018	12	278997	118	42.29 (35.01-50.65) [/100,000]
Hypospadias	2019	1	279333	122	43.68 (36.27-52.15) [/100,000]
Hypospadias	2019	2	251637	136	54.05 (45.35-63.93) [/100,000]
Hypospadias	2019	3	273087	152	55.66 (47.17-65.24) [/100,000]
Hypospadias	2019	4	268825	120	44.64 (37.01-53.37) [/100,000]
Hypospadias	2019	5	283937	142	50.01 (42.13-58.94) [/100,000]
Hypospadias	2019	6	272242	148	54.36 (45.96-63.86) [/100,000]
Hypospadias	2019	7	299580	138	46.06 (38.70-54.42) [/100,000]
Hypospadias	2019	8	307625	155	50.39 (42.77-58.97) [/100,000]
Hypospadias	2019	9	294673	158	53.62 (45.59-62.66) [/100,000]
Hypospadias	2019	10	292702	141	48.17 (40.55-56.81) [/100,000]
Hypospadias	2019	11	267192	142	53.15 (44.77-62.64) [/100,000]
Hypospadias	2019	12	276844	126	45.51 (37.91-54.19) [/100,000]
Hypospadias	2020	1	272987	140	51.28 (43.14-60.51) [/100,000]
Hypospadias	2020	2	253500	127	50.10 (41.77-59.61) [/100,000]
Hypospadias	2020	3	272222	111	40.78 (33.54-49.10) [/100,000]
Hypospadias	2020	4	261951	145	55.35 (46.71-65.13) [/100,000]
Hypospadias	2020	5	270970	123	45.39 (37.73-54.16) [/100,000]

Hypospadias	2020	6	271183	161	59.37 (50.56-69.28) [/100,000]
Hypospadias	2020	7	288818	139	48.13 (40.46-56.82) [/100,000]
Hypospadias	2020	8	287391	158	54.98 (46.74-64.25) [/100,000]
Hypospadias	2020	9	281560	148	52.56 (44.44-61.74) [/100,000]
Hypospadias	2020	10	273929	121	44.17 (36.65-52.78) [/100,000]
Hypospadias	2020	11	254215	122	47.99 (39.86-57.30) [/100,000]
Hypospadias	2020	12	260071	119	45.76 (37.91-54.75) [/100,000]
Hypospadias	2021	1	246479	126	51.12 (42.59-60.86) [/100,000]
Hypospadias	2021	2	238352	128	53.70 (44.80-63.85) [/100,000]
Hypospadias	2021	3	271449	125	46.05 (38.33-54.86) [/100,000]
Hypospadias	2021	4	261970	125	47.72 (39.72-56.85) [/100,000]
Hypospadias	2021	5	269110	143	53.14 (44.79-62.59) [/100,000]
Hypospadias	2021	6	280679	121	43.11 (35.77-51.51) [/100,000]
Hypospadias	2021	7	291919	163	55.84 (47.60-65.09) [/100,000]
Hypospadias	2021	8	295288	143	48.43 (40.82-57.04) [/100,000]
Hypospadias	2021	9	292566	120	41.02 (34.01-49.04) [/100,000]
Hypospadias	2021	10	281977	143	50.71 (42.74-59.74) [/100,000]
Hypospadias	2021	11	270183	126	46.64 (38.85-55.52) [/100,000]
Hypospadias	2021	12	277304	109	39.31 (32.28-47.41) [/100,000]
Hypospadias	2022	1	262670	120	45.68 (37.88-54.63) [/100,000]
Hypospadias	2022	2	247298	125	50.55 (42.08-60.22) [/100,000]
Hypospadias	2022	3	276060	130	47.09 (39.35-55.91) [/100,000]
Hypospadias	2022	4	258699	127	49.09 (40.93-58.41) [/100,000]
Hypospadias	2022	5	266100	134	50.36 (42.19-59.64) [/100,000]
Hypospadias	2022	6	273442	126	46.08 (38.39-54.86) [/100,000]
Hypospadias	2022	7	287161	155	53.98 (45.82-63.17) [/100,000]
Hypospadias	2022	8	303036	152	50.16 (42.50-58.79) [/100,000]
Hypospadias	2022	9	281272	110	39.11 (32.14-47.13) [/100,000]
Hypospadias	2022	10	274216	133	48.50 (40.61-57.48) [/100,000]
Hypospadias	2022	11	275054	107	38.90 (31.88-47.01) [/100,000]
Hypospadias	2022	12	281653	121	42.96 (35.65-51.33) [/100,000]
Transferred within 24 hours of delivery	2016	1	285304	1974	6.92 (6.62-7.23) [/1000]
Transferred within 24 hours of delivery	2016	2	276414	1884	6.82 (6.51-7.13) [/1000]
Transferred within 24 hours of delivery	2016	3	296672	2021	6.81 (6.52-7.11) [/1000]
Transferred within 24 hours of delivery	2016	4	282477	1863	6.60 (6.30-6.90) [/1000]
Transferred within 24 hours of delivery	2016	5	295234	1907	6.46 (6.17-6.75) [/1000]
Transferred within 24 hours of delivery	2016	6	299670	1958	6.53 (6.25-6.83) [/1000]
Transferred within 24 hours of delivery	2016	7	308873	2084	6.75 (6.46-7.04) [/1000]
Transferred within 24 hours of delivery	2016	8	323996	2169	6.69 (6.42-6.98) [/1000]
Transferred within 24 hours of delivery	2016	9	313816	1989	6.34 (6.06-6.62) [/1000]
Transferred within 24 hours of delivery	2016	10	298991	1906	6.37 (6.09-6.67) [/1000]
Transferred within 24 hours of delivery	2016	11	288429	1813	6.29 (6.00-6.58) [/1000]
Transferred within 24 hours of delivery	2016	12	292025	1931	6.61 (6.32-6.91) [/1000]
Transferred within 24 hours of delivery	2017	1	282262	1848	6.55 (6.25-6.85) [/1000]
Transferred within 24 hours of delivery	2017	2	260844	1785	6.84 (6.53-7.17) [/1000]
Transferred within 24 hours of delivery	2017	3	288345	1937	6.72 (6.42-7.02) [/1000]
Transferred within 24 hours of delivery	2017	4	270462	1817	6.72 (6.41-7.03) [/1000]
Transferred within 24 hours of delivery	2017	5	290536	1941	6.68 (6.39-6.98) [/1000]
Transferred within 24 hours of delivery	2017	6	291479	2044	7.01 (6.71-7.32) [/1000]
Transferred within 24 hours of delivery	2017	7	301598	2089	6.93 (6.63-7.23) [/1000]
Transferred within 24 hours of delivery	2017	8	318156	2200	6.91 (6.63-7.21) [/1000]
Transferred within 24 hours of delivery	2017	9	305582	2061	6.74 (6.46-7.04) [/1000]
Transferred within 24 hours of delivery	2017	10	297953	2091	7.02 (6.72-7.32) [/1000]
Transferred within 24 hours of delivery	2017	11	285718	2032	7.11 (6.81-7.43) [/1000]
Transferred within 24 hours of delivery	2017	12	284065	1951	6.87 (6.57-7.18) [/1000]
Transferred within 24 hours of delivery	2018	1	283242	2007	7.09 (6.78-7.40) [/1000]
Transferred within 24 hours of delivery	2018	2	256490	1799	7.01 (6.69-7.34) [/1000]
Transferred within 24 hours of delivery	2018	3	285290	1984	6.95 (6.65-7.27) [/1000]
Transferred within 24 hours of delivery	2018	4	268986	1915	7.12 (6.81-7.44) [/1000]
Transferred within 24 hours of delivery	2018	5	288521	2003	6.94 (6.64-7.25) [/1000]
Transferred within 24 hours of delivery	2018	6	283282	1913	6.75 (6.45-7.06) [/1000]
Transferred within 24 hours of delivery	2018	7	296175	2101	7.09 (6.79-7.40) [/1000]
Transferred within 24 hours of delivery	2018	8	311611	2240	7.19 (6.89-7.49) [/1000]
Transferred within 24 hours of delivery	2018	9	291921	2103	7.20 (6.90-7.52) [/1000]
Transferred within 24 hours of delivery	2018	10	294550	2155	7.32 (7.01-7.63) [/1000]
Transferred within 24 hours of delivery	2018	11	277778	1880	6.77 (6.47-7.08) [/1000]
Transferred within 24 hours of delivery	2018	12	279183	1992	7.14 (6.83-7.45) [/1000]
Transferred within 24 hours of delivery	2019	1	279446	1971	7.05 (6.75-7.37) [/1000]
Transferred within 24 hours of delivery	2019	2	251712	1818	7.22 (6.90-7.56) [/1000]
Transferred within 24 hours of delivery	2019	3	273112	1902	6.96 (6.66-7.28) [/1000]
Transferred within 24 hours of delivery	2019	4	268874	1866	6.94 (6.63-7.26) [/1000]

Transferred within 24 hours of delivery	2019	5	284085	1928	6.79 (6.49-7.10) [/1000]
Transferred within 24 hours of delivery	2019	6	272358	1931	7.09 (6.78-7.41) [/1000]
Transferred within 24 hours of delivery	2019	7	299607	2125	7.09 (6.80-7.40) [/1000]
Transferred within 24 hours of delivery	2019	8	307694	2286	7.43 (7.13-7.74) [/1000]
Transferred within 24 hours of delivery	2019	9	294744	2096	7.11 (6.81-7.42) [/1000]
Transferred within 24 hours of delivery	2019	10	292744	2129	7.27 (6.97-7.59) [/1000]
Transferred within 24 hours of delivery	2019	11	267255	1860	6.96 (6.65-7.28) [/1000]
Transferred within 24 hours of delivery	2019	12	276863	1855	6.70 (6.40-7.01) [/1000]
Transferred within 24 hours of delivery	2020	1	272981	1882	6.89 (6.59-7.21) [/1000]
Transferred within 24 hours of delivery	2020	2	253520	1741	6.87 (6.55-7.20) [/1000]
Transferred within 24 hours of delivery	2020	3	272222	1831	6.73 (6.42-7.04) [/1000]
Transferred within 24 hours of delivery	2020	4	261994	1645	6.28 (5.98-6.59) [/1000]
Transferred within 24 hours of delivery	2020	5	271036	1769	6.53 (6.23-6.84) [/1000]
Transferred within 24 hours of delivery	2020	6	271225	1775	6.54 (6.24-6.86) [/1000]
Transferred within 24 hours of delivery	2020	7	288883	2029	7.02 (6.72-7.33) [/1000]
Transferred within 24 hours of delivery	2020	8	287440	1886	6.56 (6.27-6.86) [/1000]
Transferred within 24 hours of delivery	2020	9	281595	1905	6.77 (6.47-7.07) [/1000]
Transferred within 24 hours of delivery	2020	10	273999	1927	7.03 (6.72-7.35) [/1000]
Transferred within 24 hours of delivery	2020	11	254130	1713	6.74 (6.43-7.07) [/1000]
Transferred within 24 hours of delivery	2020	12	259944	1789	6.88 (6.57-7.21) [/1000]
Transferred within 24 hours of delivery	2021	1	246569	1721	6.98 (6.65-7.32) [/1000]
Transferred within 24 hours of delivery	2021	2	238471	1654	6.94 (6.61-7.28) [/1000]
Transferred within 24 hours of delivery	2021	3	271598	1952	7.19 (6.87-7.51) [/1000]
Transferred within 24 hours of delivery	2021	4	261960	1865	7.12 (6.80-7.45) [/1000]
Transferred within 24 hours of delivery	2021	5	268971	1870	6.95 (6.64-7.27) [/1000]
Transferred within 24 hours of delivery	2021	6	280478	1983	7.07 (6.76-7.39) [/1000]
Transferred within 24 hours of delivery	2021	7	291730	2087	7.15 (6.85-7.47) [/1000]
Transferred within 24 hours of delivery	2021	8	295260	2030	6.88 (6.58-7.18) [/1000]
Transferred within 24 hours of delivery	2021	9	292462	2032	6.95 (6.65-7.26) [/1000]
Transferred within 24 hours of delivery	2021	10	282069	1968	6.98 (6.67-7.29) [/1000]
Transferred within 24 hours of delivery	2021	11	270102	1829	6.77 (6.47-7.09) [/1000]
Transferred within 24 hours of delivery	2021	12	277162	1975	7.13 (6.82-7.45) [/1000]
Transferred within 24 hours of delivery	2022	1	262550	1775	6.76 (6.45-7.08) [/1000]
Transferred within 24 hours of delivery	2022	2	247158	1638	6.63 (6.31-6.96) [/1000]
Transferred within 24 hours of delivery	2022	3	275773	1827	6.63 (6.33-6.93) [/1000]
Transferred within 24 hours of delivery	2022	4	258321	1898	7.35 (7.02-7.68) [/1000]
Transferred within 24 hours of delivery	2022	5	265799	1913	7.20 (6.88-7.53) [/1000]
Transferred within 24 hours of delivery	2022	6	273232	1904	6.97 (6.66-7.29) [/1000]
Transferred within 24 hours of delivery	2022	7	286820	2036	7.10 (6.79-7.41) [/1000]
Transferred within 24 hours of delivery	2022	8	302744	2158	7.13 (6.83-7.43) [/1000]
Transferred within 24 hours of delivery	2022	9	281218	1934	6.88 (6.58-7.19) [/1000]
Transferred within 24 hours of delivery	2022	10	274245	1921	7.00 (6.70-7.32) [/1000]
Transferred within 24 hours of delivery	2022	11	275203	1979	7.19 (6.88-7.51) [/1000]
Transferred within 24 hours of delivery	2022	12	281839	1952	6.93 (6.62-7.24) [/1000]
Death before birth-certificate registration	2016	1	285201	108	37.87 (31.06-45.72) [/100,000]
Death before birth-certificate registration	2016	2	276293	103	37.28 (30.43-45.21) [/100,000]
Death before birth-certificate registration	2016	3	296511	92	31.03 (25.01-38.05) [/100,000]
Death before birth-certificate registration	2016	4	282377	111	39.31 (32.34-47.34) [/100,000]
Death before birth-certificate registration	2016	5	295068	92	31.18 (25.14-38.24) [/100,000]
Death before birth-certificate registration	2016	6	299537	110	36.72 (30.18-44.26) [/100,000]
Death before birth-certificate registration	2016	7	308712	127	41.14 (34.30-48.95) [/100,000]
Death before birth-certificate registration	2016	8	323834	104	32.12 (26.24-38.91) [/100,000]
Death before birth-certificate registration	2016	9	313715	137	43.67 (36.67-51.62) [/100,000]
Death before birth-certificate registration	2016	10	298871	107	35.80 (29.34-43.26) [/100,000]
Death before birth-certificate registration	2016	11	288324	116	40.23 (33.25-48.25) [/100,000]
Death before birth-certificate registration	2016	12	291908	90	30.83 (24.79-37.90) [/100,000]
Death before birth-certificate registration	2017	1	282080	81	28.72 (22.80-35.69) [/100,000]
Death before birth-certificate registration	2017	2	260703	78	29.92 (23.65-37.34) [/100,000]
Death before birth-certificate registration	2017	3	288187	82	28.45 (22.63-35.32) [/100,000]
Death before birth-certificate registration	2017	4	270278	84	31.08 (24.79-38.48) [/100,000]
Death before birth-certificate registration	2017	5	290391	80	27.55 (21.85-34.29) [/100,000]
Death before birth-certificate registration	2017	6	291287	102	35.02 (28.55-42.51) [/100,000]
Death before birth-certificate registration	2017	7	301402	83	27.54 (21.93-34.14) [/100,000]
Death before birth-certificate registration	2017	8	317977	103	32.39 (26.44-39.28) [/100,000]
Death before birth-certificate registration	2017	9	305402	101	33.07 (26.94-40.18) [/100,000]
Death before birth-certificate registration	2017	10	297773	83	27.87 (22.20-34.55) [/100,000]
Death before birth-certificate registration	2017	11	285543	84	29.42 (23.47-36.42) [/100,000]
Death before birth-certificate registration	2017	12	283891	78	27.48 (21.72-34.29) [/100,000]
Death before birth-certificate registration	2018	1	283032	115	40.63 (33.55-48.77) [/100,000]
Death before birth-certificate registration	2018	2	256300	83	32.38 (25.79-40.14) [/100,000]
Death before birth-certificate registration	2018	3	285072	95	33.32 (26.96-40.74) [/100,000]

Death before birth-certificate registration	2018	4	268743	107	39.81 (32.63-48.11) [/100,000]
Death before birth-certificate registration	2018	5	288249	89	30.88 (24.80-37.99) [/100,000]
Death before birth-certificate registration	2018	6	283089	93	32.85 (26.52-40.24) [/100,000]
Death before birth-certificate registration	2018	7	295931	72	24.33 (19.04-30.64) [/100,000]
Death before birth-certificate registration	2018	8	311261	77	24.74 (19.52-30.92) [/100,000]
Death before birth-certificate registration	2018	9	291691	81	27.77 (22.05-34.51) [/100,000]
Death before birth-certificate registration	2018	10	294329	78	26.50 (20.95-33.07) [/100,000]
Death before birth-certificate registration	2018	11	277569	79	28.46 (22.53-35.47) [/100,000]
Death before birth-certificate registration	2018	12	278985	86	30.83 (24.66-38.07) [/100,000]
Death before birth-certificate registration	2019	1	279344	67	23.98 (18.59-30.46) [/100,000]
Death before birth-certificate registration	2019	2	251614	66	26.23 (20.29-33.37) [/100,000]
Death before birth-certificate registration	2019	3	273087	60	21.97 (16.77-28.28) [/100,000]
Death before birth-certificate registration	2019	4	268849	69	25.66 (19.97-32.48) [/100,000]
Death before birth-certificate registration	2019	5	284081	79	27.81 (22.02-34.66) [/100,000]
Death before birth-certificate registration	2019	6	272342	90	33.05 (26.57-40.62) [/100,000]
Death before birth-certificate registration	2019	7	299552	89	29.71 (23.86-36.56) [/100,000]
Death before birth-certificate registration	2019	8	307657	90	29.25 (23.52-35.96) [/100,000]
Death before birth-certificate registration	2019	9	294648	54	18.33 (13.77-23.91) [/100,000]
Death before birth-certificate registration	2019	10	292681	77	26.31 (20.76-32.88) [/100,000]
Death before birth-certificate registration	2019	11	267176	61	22.83 (17.46-29.33) [/100,000]
Death before birth-certificate registration	2019	12	276770	81	29.27 (23.24-36.37) [/100,000]
Death before birth-certificate registration	2020	1	272930	86	31.51 (25.20-38.91) [/100,000]
Death before birth-certificate registration	2020	2	253464	73	28.80 (22.58-36.21) [/100,000]
Death before birth-certificate registration	2020	3	272166	68	24.98 (19.40-31.67) [/100,000]
Death before birth-certificate registration	2020	4	261972	69	26.34 (20.49-33.33) [/100,000]
Death before birth-certificate registration	2020	5	271024	70	25.83 (20.13-32.63) [/100,000]
Death before birth-certificate registration	2020	6	271203	82	30.24 (24.05-37.53) [/100,000]
Death before birth-certificate registration	2020	7	288885	86	29.77 (23.81-36.76) [/100,000]
Death before birth-certificate registration	2020	8	287460	84	29.22 (23.31-36.18) [/100,000]
Death before birth-certificate registration	2020	9	281633	77	27.34 (21.58-34.17) [/100,000]
Death before birth-certificate registration	2020	10	273997	67	24.45 (18.95-31.05) [/100,000]
Death before birth-certificate registration	2020	11	254177	58	22.82 (17.33-29.50) [/100,000]
Death before birth-certificate registration	2020	12	259978	61	23.46 (17.95-30.14) [/100,000]
Death before birth-certificate registration	2021	1	246637	87	35.27 (28.25-43.51) [/100,000]
Death before birth-certificate registration	2021	2	238494	60	25.16 (19.20-32.38) [/100,000]
Death before birth-certificate registration	2021	3	271717	71	26.13 (20.41-32.96) [/100,000]
Death before birth-certificate registration	2021	4	262084	71	27.09 (21.16-34.17) [/100,000]
Death before birth-certificate registration	2021	5	269238	66	24.51 (18.96-31.19) [/100,000]
Death before birth-certificate registration	2021	6	280755	60	21.37 (16.31-27.51) [/100,000]
Death before birth-certificate registration	2021	7	291981	83	28.43 (22.64-35.24) [/100,000]
Death before birth-certificate registration	2021	8	295532	79	26.73 (21.16-33.31) [/100,000]
Death before birth-certificate registration	2021	9	292737	82	28.01 (22.28-34.77) [/100,000]
Death before birth-certificate registration	2021	10	282312	81	28.69 (22.79-35.66) [/100,000]
Death before birth-certificate registration	2021	11	270394	59	21.82 (16.61-28.15) [/100,000]
Death before birth-certificate registration	2021	12	277435	67	24.15 (18.72-30.67) [/100,000]
Death before birth-certificate registration	2022	1	262818	71	27.01 (21.10-34.07) [/100,000]
Death before birth-certificate registration	2022	2	247530	77	31.11 (24.55-38.88) [/100,000]
Death before birth-certificate registration	2022	3	276306	46	16.65 (12.19-22.21) [/100,000]
Death before birth-certificate registration	2022	4	258871	67	25.88 (20.06-32.87) [/100,000]
Death before birth-certificate registration	2022	5	266222	77	28.92 (22.83-36.15) [/100,000]
Death before birth-certificate registration	2022	6	273688	77	28.13 (22.20-35.16) [/100,000]
Death before birth-certificate registration	2022	7	287394	75	26.10 (20.53-32.71) [/100,000]
Death before birth-certificate registration	2022	8	303246	85	28.03 (22.39-34.66) [/100,000]
Death before birth-certificate registration	2022	9	281453	71	25.23 (19.70-31.82) [/100,000]
Death before birth-certificate registration	2022	10	274468	90	32.79 (26.37-40.30) [/100,000]
Death before birth-certificate registration	2022	11	275333	83	30.15 (24.01-37.37) [/100,000]
Death before birth-certificate registration	2022	12	281951	83	29.44 (23.45-36.49) [/100,000]

Note: Details about each variable are presented in Supplementary methods. Infants with missing obstetric estimate of gestation, or missing outcomes were excluded from the analyses of the specific outcomes.

Supplemental Table S14 Reported prevalence of the study outcomes by birth month (subgroup analysis, only infants delivered at <37 weeks' gestation)

Clinical outcome	Birth year	Birth month	No. at risk	No. events	Reported prevalence (95% confidence interval)
Congenital anomalies	2016	1	31578	303	9.60 (8.55-10.73) [/1000]
Congenital anomalies	2016	2	29832	288	9.65 (8.58-10.83) [/1000]
Congenital anomalies	2016	3	32104	319	9.94 (8.88-11.08) [/1000]
Congenital anomalies	2016	4	31273	301	9.62 (8.57-10.77) [/1000]
Congenital anomalies	2016	5	32674	325	9.95 (8.90-11.08) [/1000]
Congenital anomalies	2016	6	32921	304	9.23 (8.23-10.33) [/1000]
Congenital anomalies	2016	7	34139	328	9.61 (8.60-10.70) [/1000]
Congenital anomalies	2016	8	34122	331	9.70 (8.69-10.80) [/1000]
Congenital anomalies	2016	9	32134	303	9.43 (8.40-10.55) [/1000]
Congenital anomalies	2016	10	32377	268	8.28 (7.32-9.33) [/1000]
Congenital anomalies	2016	11	31275	283	9.05 (8.03-10.16) [/1000]
Congenital anomalies	2016	12	32940	323	9.81 (8.77-10.93) [/1000]
Congenital anomalies	2017	1	31868	308	9.66 (8.62-10.80) [/1000]
Congenital anomalies	2017	2	28411	262	9.22 (8.14-10.40) [/1000]
Congenital anomalies	2017	3	31453	301	9.57 (8.52-10.71) [/1000]
Congenital anomalies	2017	4	29890	269	9.00 (7.96-10.14) [/1000]
Congenital anomalies	2017	5	32190	315	9.79 (8.74-10.92) [/1000]
Congenital anomalies	2017	6	32693	322	9.85 (8.81-10.98) [/1000]
Congenital anomalies	2017	7	33660	352	10.46 (9.40-11.60) [/1000]
Congenital anomalies	2017	8	34410	323	9.39 (8.39-10.46) [/1000]
Congenital anomalies	2017	9	31997	298	9.31 (8.29-10.43) [/1000]
Congenital anomalies	2017	10	32373	292	9.02 (8.02-10.11) [/1000]
Congenital anomalies	2017	11	31107	288	9.26 (8.22-10.39) [/1000]
Congenital anomalies	2017	12	32153	273	8.49 (7.52-9.55) [/1000]
Congenital anomalies	2018	1	31897	317	9.94 (8.88-11.09) [/1000]
Congenital anomalies	2018	2	27999	268	9.57 (8.46-10.78) [/1000]
Congenital anomalies	2018	3	31093	286	9.20 (8.17-10.32) [/1000]
Congenital anomalies	2018	4	29726	300	10.09 (8.99-11.29) [/1000]
Congenital anomalies	2018	5	32506	321	9.88 (8.83-11.01) [/1000]
Congenital anomalies	2018	6	31835	292	9.17 (8.15-10.28) [/1000]
Congenital anomalies	2018	7	33193	283	8.53 (7.56-9.57) [/1000]
Congenital anomalies	2018	8	33603	298	8.87 (7.89-9.93) [/1000]
Congenital anomalies	2018	9	31319	281	8.97 (7.96-10.08) [/1000]
Congenital anomalies	2018	10	32726	330	10.08 (9.03-11.23) [/1000]
Congenital anomalies	2018	11	31223	284	9.10 (8.07-10.21) [/1000]
Congenital anomalies	2018	12	31948	260	8.14 (7.18-9.19) [/1000]
Congenital anomalies	2019	1	31726	303	9.55 (8.51-10.68) [/1000]
Congenital anomalies	2019	2	28476	277	9.73 (8.62-10.94) [/1000]
Congenital anomalies	2019	3	31269	288	9.21 (8.18-10.33) [/1000]
Congenital anomalies	2019	4	30256	268	8.86 (7.83-9.98) [/1000]
Congenital anomalies	2019	5	32428	273	8.42 (7.45-9.47) [/1000]
Congenital anomalies	2019	6	31895	306	9.59 (8.55-10.73) [/1000]
Congenital anomalies	2019	7	34331	303	8.83 (7.86-9.87) [/1000]
Congenital anomalies	2019	8	34301	300	8.75 (7.79-9.79) [/1000]
Congenital anomalies	2019	9	31432	276	8.78 (7.78-9.87) [/1000]
Congenital anomalies	2019	10	32919	290	8.81 (7.83-9.88) [/1000]
Congenital anomalies	2019	11	31181	267	8.56 (7.57-9.65) [/1000]
Congenital anomalies	2019	12	32305	270	8.36 (7.39-9.41) [/1000]
Congenital anomalies	2020	1	32060	272	8.48 (7.51-9.55) [/1000]
Congenital anomalies	2020	2	29406	264	8.98 (7.93-10.12) [/1000]
Congenital anomalies	2020	3	29632	280	9.45 (8.38-10.62) [/1000]
Congenital anomalies	2020	4	28488	252	8.85 (7.79-10.00) [/1000]
Congenital anomalies	2020	5	30326	276	9.10 (8.06-10.23) [/1000]
Congenital anomalies	2020	6	30775	278	9.03 (8.01-10.15) [/1000]
Congenital anomalies	2020	7	32621	296	9.07 (8.07-10.16) [/1000]
Congenital anomalies	2020	8	32042	257	8.02 (7.07-9.06) [/1000]
Congenital anomalies	2020	9	29949	284	9.48 (8.42-10.65) [/1000]
Congenital anomalies	2020	10	30948	281	9.08 (8.05-10.20) [/1000]
Congenital anomalies	2020	11	28169	275	9.76 (8.65-10.98) [/1000]
Congenital anomalies	2020	12	29569	273	9.23 (8.17-10.39) [/1000]
Congenital anomalies	2021	1	30429	280	9.20 (8.16-10.34) [/1000]
Congenital anomalies	2021	2	27741	278	10.02 (8.88-11.26) [/1000]
Congenital anomalies	2021	3	30839	283	9.18 (8.14-10.30) [/1000]
Congenital anomalies	2021	4	30961	282	9.11 (8.08-10.23) [/1000]
Congenital anomalies	2021	5	31530	278	8.82 (7.81-9.91) [/1000]
Congenital anomalies	2021	6	32665	284	8.69 (7.72-9.76) [/1000]
Congenital anomalies	2021	7	33868	332	9.80 (8.78-10.91) [/1000]

Congenital anomalies	2021	8	34505	312	9.04 (8.07-10.10) [/1000]
Congenital anomalies	2021	9	32845	301	9.16 (8.16-10.25) [/1000]
Congenital anomalies	2021	10	32810	282	8.59 (7.62-9.65) [/1000]
Congenital anomalies	2021	11	31316	244	7.79 (6.85-8.83) [/1000]
Congenital anomalies	2021	12	33601	316	9.40 (8.40-10.49) [/1000]
Congenital anomalies	2022	1	32051	275	8.58 (7.60-9.65) [/1000]
Congenital anomalies	2022	2	28206	246	8.72 (7.67-9.88) [/1000]
Congenital anomalies	2022	3	31220	320	10.25 (9.16-11.43) [/1000]
Congenital anomalies	2022	4	29817	237	7.95 (6.97-9.02) [/1000]
Congenital anomalies	2022	5	31433	267	8.49 (7.51-9.57) [/1000]
Congenital anomalies	2022	6	32214	288	8.94 (7.94-10.03) [/1000]
Congenital anomalies	2022	7	33507	288	8.60 (7.63-9.64) [/1000]
Congenital anomalies	2022	8	32850	289	8.80 (7.82-9.87) [/1000]
Congenital anomalies	2022	9	30974	261	8.43 (7.44-9.51) [/1000]
Congenital anomalies	2022	10	32543	271	8.33 (7.37-9.38) [/1000]
Congenital anomalies	2022	11	31996	253	7.91 (6.97-8.94) [/1000]
Congenital anomalies	2022	12	33041	251	7.60 (6.69-8.59) [/1000]
Anencephaly	2016	1	31578	12	38.00 (19.64-66.37) [/100,000]
Anencephaly	2016	2	29832	9	30.17 (13.80-57.26) [/100,000]
Anencephaly	2016	3	32104	12	37.38 (19.32-65.28) [/100,000]
Anencephaly	2016	4	31273	14	44.77 (24.48-75.10) [/100,000]
Anencephaly	2016	5	32674	11	33.67 (16.81-60.23) [/100,000]
Anencephaly	2016	6	32921	12	36.45 (18.84-63.66) [/100,000]
Anencephaly	2016	7	34139	13	38.08 (20.28-65.11) [/100,000]
Anencephaly	2016	8	34122	13	38.10 (20.29-65.14) [/100,000]
Anencephaly	2016	9	32134	9	28.01 (12.81-53.16) [/100,000]
Anencephaly	2016	10	32377	11	33.97 (16.96-60.78) [/100,000]
Anencephaly	2016	11	31275	11	35.17 (17.56-62.92) [/100,000]
Anencephaly	2016	12	32940	14	42.50 (23.24-71.30) [/100,000]
Anencephaly	2017	1	31868	16	50.21 (28.70-81.52) [/100,000]
Anencephaly	2017	2	28411	9	31.68 (14.49-60.13) [/100,000]
Anencephaly	2017	3	31453	19	60.41 (36.37-94.32) [/100,000]
Anencephaly	2017	4	29890	16	53.53 (30.60-86.91) [/100,000]
Anencephaly	2017	5	32190	19	59.02 (35.54-92.16) [/100,000]
Anencephaly	2017	6	32693	13	39.76 (21.17-67.99) [/100,000]
Anencephaly	2017	7	33660	11	32.68 (16.31-58.47) [/100,000]
Anencephaly	2017	8	34410	16	46.50 (26.58-75.50) [/100,000]
Anencephaly	2017	9	31997	22	68.76 (43.09-104.08) [/100,000]
Anencephaly	2017	10	32373	13	40.16 (21.38-68.66) [/100,000]
Anencephaly	2017	11	31107	15	48.22 (26.99-79.52) [/100,000]
Anencephaly	2017	12	32153	15	46.65 (26.11-76.93) [/100,000]
Anencephaly	2018	1	31897	11	34.49 (17.22-61.70) [/100,000]
Anencephaly	2018	2	27999	12	42.86 (22.15-74.85) [/100,000]
Anencephaly	2018	3	31093	7	22.51 (9.05-46.38) [/100,000]
Anencephaly	2018	4	29726	12	40.37 (20.86-70.51) [/100,000]
Anencephaly	2018	5	32506	18	55.37 (32.82-87.50) [/100,000]
Anencephaly	2018	6	31835	11	34.55 (17.25-61.82) [/100,000]
Anencephaly	2018	7	33193	13	39.16 (20.86-66.96) [/100,000]
Anencephaly	2018	8	33603	7	20.83 (8.38-42.92) [/100,000]
Anencephaly	2018	9	31319	17	54.28 (31.62-86.89) [/100,000]
Anencephaly	2018	10	32726	9	27.50 (12.58-52.20) [/100,000]
Anencephaly	2018	11	31223	14	44.84 (24.52-75.22) [/100,000]
Anencephaly	2018	12	31948	4	12.52 (3.41-32.05) [/100,000]
Anencephaly	2019	1	31726	9	28.37 (12.97-53.84) [/100,000]
Anencephaly	2019	2	28476	9	31.61 (14.45-59.99) [/100,000]
Anencephaly	2019	3	31269	12	38.38 (19.83-67.03) [/100,000]
Anencephaly	2019	4	30256	6	19.83 (7.28-43.16) [/100,000]
Anencephaly	2019	5	32428	14	43.17 (23.60-72.43) [/100,000]
Anencephaly	2019	6	31895	19	59.57 (35.87-93.01) [/100,000]
Anencephaly	2019	7	34331	13	37.87 (20.16-64.74) [/100,000]
Anencephaly	2019	8	34301	11	32.07 (16.01-57.37) [/100,000]
Anencephaly	2019	9	31432	10	31.81 (15.26-58.50) [/100,000]
Anencephaly	2019	10	32919	8	24.30 (10.49-47.88) [/100,000]
Anencephaly	2019	11	31181	9	28.86 (13.20-54.79) [/100,000]
Anencephaly	2019	12	32305	11	34.05 (17.00-60.92) [/100,000]
Anencephaly	2020	1	32060	6	18.71 (6.87-40.73) [/100,000]
Anencephaly	2020	2	29406	13	44.21 (23.54-75.59) [/100,000]
Anencephaly	2020	3	29632	11	37.12 (18.53-66.41) [/100,000]
Anencephaly	2020	4	28488	12	42.12 (21.77-73.57) [/100,000]
Anencephaly	2020	5	30326	9	29.68 (13.57-56.33) [/100,000]
Anencephaly	2020	6	30775	11	35.74 (17.84-63.95) [/100,000]

Anencephaly	2020	7	32621	15	45.98 (25.74-75.83) [/100,000]
Anencephaly	2020	8	32042	9	28.09 (12.84-53.31) [/100,000]
Anencephaly	2020	9	29949	10	33.39 (16.01-61.40) [/100,000]
Anencephaly	2020	10	30948	10	32.31 (15.50-59.42) [/100,000]
Anencephaly	2020	11	28169	17	60.35 (35.16-96.61) [/100,000]
Anencephaly	2020	12	29569	10	33.82 (16.22-62.19) [/100,000]
Anencephaly	2021	1	30429	10	32.86 (15.76-60.43) [/100,000]
Anencephaly	2021	2	27741	8	28.84 (12.45-56.81) [/100,000]
Anencephaly	2021	3	30839	11	35.67 (17.81-63.81) [/100,000]
Anencephaly	2021	4	30961	10	32.30 (15.49-59.39) [/100,000]
Anencephaly	2021	5	31530	7	22.20 (8.93-45.74) [/100,000]
Anencephaly	2021	6	32665	9	27.55 (12.60-52.30) [/100,000]
Anencephaly	2021	7	33868	19	56.10 (33.78-87.59) [/100,000]
Anencephaly	2021	8	34505	8	23.19 (10.01-45.68) [/100,000]
Anencephaly	2021	9	32845	9	27.40 (12.53-52.01) [/100,000]
Anencephaly	2021	10	32810	6	18.29 (6.71-39.80) [/100,000]
Anencephaly	2021	11	31316	10	31.93 (15.31-58.72) [/100,000]
Anencephaly	2021	12	33601	14	41.67 (22.78-69.90) [/100,000]
Anencephaly	2022	1	32051	11	34.32 (17.13-61.40) [/100,000]
Anencephaly	2022	2	28206	8	28.36 (12.25-55.88) [/100,000]
Anencephaly	2022	3	31220	13	41.64 (22.17-71.20) [/100,000]
Anencephaly	2022	4	29817	8	26.83 (11.58-52.86) [/100,000]
Anencephaly	2022	5	31433	19	60.45 (36.40-94.38) [/100,000]
Anencephaly	2022	6	32214	14	43.46 (23.76-72.91) [/100,000]
Anencephaly	2022	7	33507	12	35.81 (18.51-62.55) [/100,000]
Anencephaly	2022	8	32850	16	48.71 (27.84-79.08) [/100,000]
Anencephaly	2022	9	30974	11	35.51 (17.73-63.53) [/100,000]
Anencephaly	2022	10	32543	12	36.87 (19.05-64.40) [/100,000]
Anencephaly	2022	11	31996	10	31.25 (14.99-57.47) [/100,000]
Anencephaly	2022	12	33041	12	36.32 (18.77-63.43) [/100,000]
Meningomyelocele/Spina bifida	2016	1	31578	10	31.67 (15.19-58.23) [/100,000]
Meningomyelocele/Spina bifida	2016	2	29832	15	50.28 (28.14-82.92) [/100,000]
Meningomyelocele/Spina bifida	2016	3	32104	6	18.69 (6.86-40.67) [/100,000]
Meningomyelocele/Spina bifida	2016	4	31273	15	47.96 (26.85-79.10) [/100,000]
Meningomyelocele/Spina bifida	2016	5	32674	19	58.15 (35.01-90.79) [/100,000]
Meningomyelocele/Spina bifida	2016	6	32921	14	42.53 (23.25-71.34) [/100,000]
Meningomyelocele/Spina bifida	2016	7	34139	10	29.29 (14.05-53.86) [/100,000]
Meningomyelocele/Spina bifida	2016	8	34122	14	41.03 (22.43-68.83) [/100,000]
Meningomyelocele/Spina bifida	2016	9	32134	7	21.78 (8.76-44.88) [/100,000]
Meningomyelocele/Spina bifida	2016	10	32377	13	40.15 (21.38-68.65) [/100,000]
Meningomyelocele/Spina bifida	2016	11	31275	14	44.76 (24.48-75.10) [/100,000]
Meningomyelocele/Spina bifida	2016	12	32940	15	45.54 (25.49-75.10) [/100,000]
Meningomyelocele/Spina bifida	2017	1	31868	16	50.21 (28.70-81.52) [/100,000]
Meningomyelocele/Spina bifida	2017	2	28411	16	56.32 (32.19-91.44) [/100,000]
Meningomyelocele/Spina bifida	2017	3	31453	19	60.41 (36.37-94.32) [/100,000]
Meningomyelocele/Spina bifida	2017	4	29890	16	53.53 (30.60-86.91) [/100,000]
Meningomyelocele/Spina bifida	2017	5	32190	17	52.81 (30.77-84.54) [/100,000]
Meningomyelocele/Spina bifida	2017	6	32693	14	42.82 (23.41-71.84) [/100,000]
Meningomyelocele/Spina bifida	2017	7	33660	19	56.45 (33.99-88.13) [/100,000]
Meningomyelocele/Spina bifida	2017	8	34410	20	58.12 (35.51-89.75) [/100,000]
Meningomyelocele/Spina bifida	2017	9	31997	13	40.63 (21.63-69.47) [/100,000]
Meningomyelocele/Spina bifida	2017	10	32373	9	27.80 (12.71-52.77) [/100,000]
Meningomyelocele/Spina bifida	2017	11	31107	12	38.58 (19.93-67.38) [/100,000]
Meningomyelocele/Spina bifida	2017	12	32153	18	55.98 (33.18-88.46) [/100,000]
Meningomyelocele/Spina bifida	2018	1	31897	21	65.84 (40.76-100.62) [/100,000]
Meningomyelocele/Spina bifida	2018	2	27999	13	46.43 (24.72-79.38) [/100,000]
Meningomyelocele/Spina bifida	2018	3	31093	11	35.38 (17.66-63.29) [/100,000]
Meningomyelocele/Spina bifida	2018	4	29726	19	63.92 (38.49-99.80) [/100,000]
Meningomyelocele/Spina bifida	2018	5	32506	8	24.61 (10.63-48.49) [/100,000]
Meningomyelocele/Spina bifida	2018	6	31835	19	59.68 (35.94-93.19) [/100,000]
Meningomyelocele/Spina bifida	2018	7	33193	17	51.22 (29.84-81.99) [/100,000]
Meningomyelocele/Spina bifida	2018	8	33603	16	47.61 (27.22-77.31) [/100,000]
Meningomyelocele/Spina bifida	2018	9	31319	10	31.93 (15.31-58.71) [/100,000]
Meningomyelocele/Spina bifida	2018	10	32726	12	36.67 (18.95-64.04) [/100,000]
Meningomyelocele/Spina bifida	2018	11	31223	22	70.46 (44.16-106.66) [/100,000]
Meningomyelocele/Spina bifida	2018	12	31948	12	37.56 (19.41-65.60) [/100,000]
Meningomyelocele/Spina bifida	2019	1	31726	12	37.82 (19.55-66.06) [/100,000]
Meningomyelocele/Spina bifida	2019	2	28476	10	35.12 (16.84-64.57) [/100,000]
Meningomyelocele/Spina bifida	2019	3	31269	9	28.78 (13.16-54.63) [/100,000]
Meningomyelocele/Spina bifida	2019	4	30256	20	66.10 (40.38-102.07) [/100,000]
Meningomyelocele/Spina bifida	2019	5	32428	14	43.17 (23.60-72.43) [/100,000]

Meningomyelocele/Spina bifida	2019	6	31895	22	68.98 (43.23-104.41) [/100,000]
Meningomyelocele/Spina bifida	2019	7	34331	22	64.08 (40.16-97.00) [/100,000]
Meningomyelocele/Spina bifida	2019	8	34301	19	55.39 (33.35-86.49) [/100,000]
Meningomyelocele/Spina bifida	2019	9	31432	12	38.18 (19.73-66.68) [/100,000]
Meningomyelocele/Spina bifida	2019	10	32919	15	45.57 (25.51-75.14) [/100,000]
Meningomyelocele/Spina bifida	2019	11	31181	7	22.45 (9.03-46.25) [/100,000]
Meningomyelocele/Spina bifida	2019	12	32305	13	40.24 (21.43-68.80) [/100,000]
Meningomyelocele/Spina bifida	2020	1	32060	20	62.38 (38.11-96.33) [/100,000]
Meningomyelocele/Spina bifida	2020	2	29406	10	34.01 (16.31-62.53) [/100,000]
Meningomyelocele/Spina bifida	2020	3	29632	15	50.62 (28.33-83.48) [/100,000]
Meningomyelocele/Spina bifida	2020	4	28488	12	42.12 (21.77-73.57) [/100,000]
Meningomyelocele/Spina bifida	2020	5	30326	15	49.46 (27.69-81.57) [/100,000]
Meningomyelocele/Spina bifida	2020	6	30775	11	35.74 (17.84-63.95) [/100,000]
Meningomyelocele/Spina bifida	2020	7	32621	22	67.44 (42.27-102.09) [/100,000]
Meningomyelocele/Spina bifida	2020	8	32042	15	46.81 (26.20-77.20) [/100,000]
Meningomyelocele/Spina bifida	2020	9	29949	10	33.39 (16.01-61.40) [/100,000]
Meningomyelocele/Spina bifida	2020	10	30948	14	45.24 (24.73-75.89) [/100,000]
Meningomyelocele/Spina bifida	2020	11	28169	10	35.50 (17.02-65.28) [/100,000]
Meningomyelocele/Spina bifida	2020	12	29569	14	47.35 (25.89-79.43) [/100,000]
Meningomyelocele/Spina bifida	2021	1	30429	13	42.72 (22.75-73.05) [/100,000]
Meningomyelocele/Spina bifida	2021	2	27741	14	50.47 (27.59-84.66) [/100,000]
Meningomyelocele/Spina bifida	2021	3	30839	13	42.15 (22.45-72.07) [/100,000]
Meningomyelocele/Spina bifida	2021	4	30961	12	38.76 (20.03-67.69) [/100,000]
Meningomyelocele/Spina bifida	2021	5	31530	19	60.26 (36.28-94.09) [/100,000]
Meningomyelocele/Spina bifida	2021	6	32665	11	33.68 (16.81-60.25) [/100,000]
Meningomyelocele/Spina bifida	2021	7	33868	13	38.38 (20.44-65.63) [/100,000]
Meningomyelocele/Spina bifida	2021	8	34505	16	46.37 (26.51-75.29) [/100,000]
Meningomyelocele/Spina bifida	2021	9	32845	12	36.54 (18.88-63.81) [/100,000]
Meningomyelocele/Spina bifida	2021	10	32810	10	30.48 (14.62-56.04) [/100,000]
Meningomyelocele/Spina bifida	2021	11	31316	10	31.93 (15.31-58.72) [/100,000]
Meningomyelocele/Spina bifida	2021	12	33601	17	50.59 (29.48-80.99) [/100,000]
Meningomyelocele/Spina bifida	2022	1	32051	17	53.04 (30.90-84.91) [/100,000]
Meningomyelocele/Spina bifida	2022	2	28206	20	70.91 (43.32-109.49) [/100,000]
Meningomyelocele/Spina bifida	2022	3	31220	18	57.66 (34.17-91.11) [/100,000]
Meningomyelocele/Spina bifida	2022	4	29817	19	63.72 (38.37-99.49) [/100,000]
Meningomyelocele/Spina bifida	2022	5	31433	25	79.53 (51.48-117.39) [/100,000]
Meningomyelocele/Spina bifida	2022	6	32214	17	52.77 (30.74-84.48) [/100,000]
Meningomyelocele/Spina bifida	2022	7	33507	17	50.74 (29.56-81.22) [/100,000]
Meningomyelocele/Spina bifida	2022	8	32850	7	21.31 (8.57-43.90) [/100,000]
Meningomyelocele/Spina bifida	2022	9	30974	20	64.57 (39.45-99.71) [/100,000]
Meningomyelocele/Spina bifida	2022	10	32543	10	30.73 (14.74-56.50) [/100,000]
Meningomyelocele/Spina bifida	2022	11	31996	13	40.63 (21.64-69.47) [/100,000]
Meningomyelocele/Spina bifida	2022	12	33041	19	57.50 (34.62-89.79) [/100,000]
Cyanotic congenital heart disease	2016	1	31578	39	123.50 (87.84-168.80) [/100,000]
Cyanotic congenital heart disease	2016	2	29832	47	157.55 (115.78-209.45) [/100,000]
Cyanotic congenital heart disease	2016	3	32104	52	161.97 (120.99-212.35) [/100,000]
Cyanotic congenital heart disease	2016	4	31273	54	172.67 (129.74-225.24) [/100,000]
Cyanotic congenital heart disease	2016	5	32674	49	149.97 (110.97-198.22) [/100,000]
Cyanotic congenital heart disease	2016	6	32921	57	173.14 (131.16-224.27) [/100,000]
Cyanotic congenital heart disease	2016	7	34139	45	131.81 (96.16-176.34) [/100,000]
Cyanotic congenital heart disease	2016	8	34122	59	172.91 (131.65-222.98) [/100,000]
Cyanotic congenital heart disease	2016	9	32134	41	127.59 (91.58-173.05) [/100,000]
Cyanotic congenital heart disease	2016	10	32377	55	169.87 (128.00-221.06) [/100,000]
Cyanotic congenital heart disease	2016	11	31275	43	137.49 (99.52-185.15) [/100,000]
Cyanotic congenital heart disease	2016	12	32940	44	133.58 (97.07-179.28) [/100,000]
Cyanotic congenital heart disease	2017	1	31868	38	119.24 (84.40-163.63) [/100,000]
Cyanotic congenital heart disease	2017	2	28411	47	165.43 (121.58-219.93) [/100,000]
Cyanotic congenital heart disease	2017	3	31453	57	181.22 (137.28-234.73) [/100,000]
Cyanotic congenital heart disease	2017	4	29890	39	130.48 (92.80-178.33) [/100,000]
Cyanotic congenital heart disease	2017	5	32190	44	136.69 (99.34-183.45) [/100,000]
Cyanotic congenital heart disease	2017	6	32693	48	146.82 (108.27-194.62) [/100,000]
Cyanotic congenital heart disease	2017	7	33660	55	163.40 (123.12-212.63) [/100,000]
Cyanotic congenital heart disease	2017	8	34410	49	142.40 (105.37-188.22) [/100,000]
Cyanotic congenital heart disease	2017	9	31997	41	128.14 (91.97-173.79) [/100,000]
Cyanotic congenital heart disease	2017	10	32373	47	145.18 (106.69-193.02) [/100,000]
Cyanotic congenital heart disease	2017	11	31107	53	170.38 (127.65-222.80) [/100,000]
Cyanotic congenital heart disease	2017	12	32153	35	108.85 (75.83-151.36) [/100,000]
Cyanotic congenital heart disease	2018	1	31897	43	134.81 (97.58-181.54) [/100,000]
Cyanotic congenital heart disease	2018	2	27999	46	164.29 (120.31-219.08) [/100,000]
Cyanotic congenital heart disease	2018	3	31093	49	157.59 (116.61-208.29) [/100,000]
Cyanotic congenital heart disease	2018	4	29726	43	144.65 (104.71-194.80) [/100,000]

Cyanotic congenital heart disease	2018	5	32506	45	138.44 (100.99-185.19) [/100,000]
Cyanotic congenital heart disease	2018	6	31835	52	163.34 (122.02-214.15) [/100,000]
Cyanotic congenital heart disease	2018	7	33193	42	126.53 (91.21-171.00) [/100,000]
Cyanotic congenital heart disease	2018	8	33603	45	133.92 (97.70-179.15) [/100,000]
Cyanotic congenital heart disease	2018	9	31319	43	137.30 (99.38-184.89) [/100,000]
Cyanotic congenital heart disease	2018	10	32726	51	155.84 (116.05-204.85) [/100,000]
Cyanotic congenital heart disease	2018	11	31223	48	153.73 (113.37-203.78) [/100,000]
Cyanotic congenital heart disease	2018	12	31948	48	150.24 (110.80-199.15) [/100,000]
Cyanotic congenital heart disease	2019	1	31726	34	107.17 (74.23-149.72) [/100,000]
Cyanotic congenital heart disease	2019	2	28476	39	136.96 (97.41-187.18) [/100,000]
Cyanotic congenital heart disease	2019	3	31269	56	179.09 (135.31-232.50) [/100,000]
Cyanotic congenital heart disease	2019	4	30256	48	158.65 (117.00-210.29) [/100,000]
Cyanotic congenital heart disease	2019	5	32428	42	129.52 (93.36-175.03) [/100,000]
Cyanotic congenital heart disease	2019	6	31895	44	137.95 (100.25-185.15) [/100,000]
Cyanotic congenital heart disease	2019	7	34331	46	133.99 (98.11-178.68) [/100,000]
Cyanotic congenital heart disease	2019	8	34301	48	139.94 (103.20-185.49) [/100,000]
Cyanotic congenital heart disease	2019	9	31432	45	143.17 (104.44-191.52) [/100,000]
Cyanotic congenital heart disease	2019	10	32919	59	179.23 (136.46-231.13) [/100,000]
Cyanotic congenital heart disease	2019	11	31181	58	186.01 (141.28-240.40) [/100,000]
Cyanotic congenital heart disease	2019	12	32305	52	160.97 (120.24-211.03) [/100,000]
Cyanotic congenital heart disease	2020	1	32060	35	109.17 (76.05-151.80) [/100,000]
Cyanotic congenital heart disease	2020	2	29406	40	136.03 (97.20-185.18) [/100,000]
Cyanotic congenital heart disease	2020	3	29632	53	178.86 (134.01-233.89) [/100,000]
Cyanotic congenital heart disease	2020	4	28488	39	136.90 (97.37-187.10) [/100,000]
Cyanotic congenital heart disease	2020	5	30326	56	184.66 (139.52-239.73) [/100,000]
Cyanotic congenital heart disease	2020	6	30775	53	172.22 (129.03-225.21) [/100,000]
Cyanotic congenital heart disease	2020	7	32621	44	134.88 (98.02-181.03) [/100,000]
Cyanotic congenital heart disease	2020	8	32042	43	134.20 (97.14-180.72) [/100,000]
Cyanotic congenital heart disease	2020	9	29949	49	163.61 (121.06-216.25) [/100,000]
Cyanotic congenital heart disease	2020	10	30948	47	151.87 (111.61-201.90) [/100,000]
Cyanotic congenital heart disease	2020	11	28169	48	170.40 (125.67-225.86) [/100,000]
Cyanotic congenital heart disease	2020	12	29569	56	189.39 (143.09-245.87) [/100,000]
Cyanotic congenital heart disease	2021	1	30429	59	193.89 (147.63-250.04) [/100,000]
Cyanotic congenital heart disease	2021	2	27741	57	205.47 (155.66-266.13) [/100,000]
Cyanotic congenital heart disease	2021	3	30839	65	210.77 (162.71-268.57) [/100,000]
Cyanotic congenital heart disease	2021	4	30961	54	174.41 (131.05-227.51) [/100,000]
Cyanotic congenital heart disease	2021	5	31530	42	133.21 (96.02-180.01) [/100,000]
Cyanotic congenital heart disease	2021	6	32665	51	156.13 (116.27-205.23) [/100,000]
Cyanotic congenital heart disease	2021	7	33868	65	191.92 (148.15-244.56) [/100,000]
Cyanotic congenital heart disease	2021	8	34505	66	191.28 (147.96-243.29) [/100,000]
Cyanotic congenital heart disease	2021	9	32845	66	200.94 (155.44-255.58) [/100,000]
Cyanotic congenital heart disease	2021	10	32810	66	201.16 (155.61-255.85) [/100,000]
Cyanotic congenital heart disease	2021	11	31316	51	162.86 (121.28-214.07) [/100,000]
Cyanotic congenital heart disease	2021	12	33601	61	181.54 (138.89-233.14) [/100,000]
Cyanotic congenital heart disease	2022	1	32051	53	165.36 (123.89-216.24) [/100,000]
Cyanotic congenital heart disease	2022	2	28206	55	194.99 (146.93-253.74) [/100,000]
Cyanotic congenital heart disease	2022	3	31220	52	166.56 (124.42-218.36) [/100,000]
Cyanotic congenital heart disease	2022	4	29817	50	167.69 (124.49-221.02) [/100,000]
Cyanotic congenital heart disease	2022	5	31433	61	194.06 (148.47-249.21) [/100,000]
Cyanotic congenital heart disease	2022	6	32214	61	189.36 (144.87-243.17) [/100,000]
Cyanotic congenital heart disease	2022	7	33507	46	137.28 (100.53-183.08) [/100,000]
Cyanotic congenital heart disease	2022	8	32850	63	191.78 (147.40-245.31) [/100,000]
Cyanotic congenital heart disease	2022	9	30974	51	164.65 (122.62-216.43) [/100,000]
Cyanotic congenital heart disease	2022	10	32543	52	159.79 (119.36-209.49) [/100,000]
Cyanotic congenital heart disease	2022	11	31996	45	140.64 (102.60-188.15) [/100,000]
Cyanotic congenital heart disease	2022	12	33041	39	118.04 (83.95-161.32) [/100,000]
Congenital diaphragmatic hernia	2016	1	31578	5	15.83 (5.14-36.95) [/100,000]
Congenital diaphragmatic hernia	2016	2	29832	13	43.58 (23.21-74.51) [/100,000]
Congenital diaphragmatic hernia	2016	3	32104	14	43.61 (23.84-73.16) [/100,000]
Congenital diaphragmatic hernia	2016	4	31273	12	38.37 (19.83-67.02) [/100,000]
Congenital diaphragmatic hernia	2016	5	32674	10	30.61 (14.68-56.28) [/100,000]
Congenital diaphragmatic hernia	2016	6	32921	11	33.41 (16.68-59.78) [/100,000]
Congenital diaphragmatic hernia	2016	7	34139	17	49.80 (29.01-79.72) [/100,000]
Congenital diaphragmatic hernia	2016	8	34122	12	35.17 (18.17-61.42) [/100,000]
Congenital diaphragmatic hernia	2016	9	32134	12	37.34 (19.30-65.22) [/100,000]
Congenital diaphragmatic hernia	2016	10	32377	10	30.89 (14.81-56.79) [/100,000]
Congenital diaphragmatic hernia	2016	11	31275	10	31.97 (15.33-58.79) [/100,000]
Congenital diaphragmatic hernia	2016	12	32940	10	30.36 (14.56-55.82) [/100,000]
Congenital diaphragmatic hernia	2017	1	31868	8	25.10 (10.84-49.46) [/100,000]
Congenital diaphragmatic hernia	2017	2	28411	16	56.32 (32.19-91.44) [/100,000]
Congenital diaphragmatic hernia	2017	3	31453	8	25.43 (10.98-50.11) [/100,000]

Congenital diaphragmatic hernia	2017	4	29890	11	36.80 (18.37-65.84) [/100,000]
Congenital diaphragmatic hernia	2017	5	32190	12	37.28 (19.26-65.11) [/100,000]
Congenital diaphragmatic hernia	2017	6	32693	11	33.65 (16.80-60.19) [/100,000]
Congenital diaphragmatic hernia	2017	7	33660	15	44.56 (24.94-73.49) [/100,000]
Congenital diaphragmatic hernia	2017	8	34410	6	17.44 (6.40-37.95) [/100,000]
Congenital diaphragmatic hernia	2017	9	31997	10	31.25 (14.99-57.47) [/100,000]
Congenital diaphragmatic hernia	2017	10	32373	13	40.16 (21.38-68.66) [/100,000]
Congenital diaphragmatic hernia	2017	11	31107	8	25.72 (11.10-50.67) [/100,000]
Congenital diaphragmatic hernia	2017	12	32153	8	24.88 (10.74-49.02) [/100,000]
Congenital diaphragmatic hernia	2018	1	31897	13	40.76 (21.70-69.68) [/100,000]
Congenital diaphragmatic hernia	2018	2	27999	9	32.14 (14.70-61.01) [/100,000]
Congenital diaphragmatic hernia	2018	3	31093	16	51.46 (29.42-83.55) [/100,000]
Congenital diaphragmatic hernia	2018	4	29726	8	26.91 (11.62-53.02) [/100,000]
Congenital diaphragmatic hernia	2018	5	32506	10	30.76 (14.75-56.57) [/100,000]
Congenital diaphragmatic hernia	2018	6	31835	11	34.55 (17.25-61.82) [/100,000]
Congenital diaphragmatic hernia	2018	7	33193	11	33.14 (16.54-59.29) [/100,000]
Congenital diaphragmatic hernia	2018	8	33603	9	26.78 (12.25-50.84) [/100,000]
Congenital diaphragmatic hernia	2018	9	31319	9	28.74 (13.14-54.54) [/100,000]
Congenital diaphragmatic hernia	2018	10	32726	13	39.72 (21.15-67.92) [/100,000]
Congenital diaphragmatic hernia	2018	11	31223	15	48.04 (26.89-79.22) [/100,000]
Congenital diaphragmatic hernia	2018	12	31948	5	15.65 (5.08-36.52) [/100,000]
Congenital diaphragmatic hernia	2019	1	31726	16	50.43 (28.83-81.89) [/100,000]
Congenital diaphragmatic hernia	2019	2	28476	12	42.14 (21.78-73.60) [/100,000]
Congenital diaphragmatic hernia	2019	3	31269	7	22.39 (9.00-46.12) [/100,000]
Congenital diaphragmatic hernia	2019	4	30256	9	29.75 (13.60-56.46) [/100,000]
Congenital diaphragmatic hernia	2019	5	32428	6	18.50 (6.79-40.27) [/100,000]
Congenital diaphragmatic hernia	2019	6	31895	12	37.62 (19.44-65.71) [/100,000]
Congenital diaphragmatic hernia	2019	7	34331	19	55.34 (33.32-86.41) [/100,000]
Congenital diaphragmatic hernia	2019	8	34301	8	23.32 (10.07-45.95) [/100,000]
Congenital diaphragmatic hernia	2019	9	31432	14	44.54 (24.35-74.72) [/100,000]
Congenital diaphragmatic hernia	2019	10	32919	10	30.38 (14.57-55.86) [/100,000]
Congenital diaphragmatic hernia	2019	11	31181	10	32.07 (15.38-58.97) [/100,000]
Congenital diaphragmatic hernia	2019	12	32305	7	21.67 (8.71-44.64) [/100,000]
Congenital diaphragmatic hernia	2020	1	32060	9	28.07 (12.84-53.28) [/100,000]
Congenital diaphragmatic hernia	2020	2	29406	8	27.21 (11.75-53.60) [/100,000]
Congenital diaphragmatic hernia	2020	3	29632	15	50.62 (28.33-83.48) [/100,000]
Congenital diaphragmatic hernia	2020	4	28488	13	45.63 (24.30-78.02) [/100,000]
Congenital diaphragmatic hernia	2020	5	30326	9	29.68 (13.57-56.33) [/100,000]
Congenital diaphragmatic hernia	2020	6	30775	9	29.24 (13.37-55.51) [/100,000]
Congenital diaphragmatic hernia	2020	7	32621	16	49.05 (28.04-79.64) [/100,000]
Congenital diaphragmatic hernia	2020	8	32042	6	18.73 (6.87-40.75) [/100,000]
Congenital diaphragmatic hernia	2020	9	29949	11	36.73 (18.34-65.71) [/100,000]
Congenital diaphragmatic hernia	2020	10	30948	15	48.47 (27.13-79.93) [/100,000]
Congenital diaphragmatic hernia	2020	11	28169	12	42.60 (22.01-74.40) [/100,000]
Congenital diaphragmatic hernia	2020	12	29569	6	20.29 (7.45-44.16) [/100,000]
Congenital diaphragmatic hernia	2021	1	30429	11	36.15 (18.05-64.67) [/100,000]
Congenital diaphragmatic hernia	2021	2	27741	16	57.68 (32.97-93.65) [/100,000]
Congenital diaphragmatic hernia	2021	3	30839	11	35.67 (17.81-63.81) [/100,000]
Congenital diaphragmatic hernia	2021	4	30961	13	41.99 (22.36-71.79) [/100,000]
Congenital diaphragmatic hernia	2021	5	31530	7	22.20 (8.93-45.74) [/100,000]
Congenital diaphragmatic hernia	2021	6	32665	6	18.37 (6.74-39.98) [/100,000]
Congenital diaphragmatic hernia	2021	7	33868	11	32.48 (16.21-58.11) [/100,000]
Congenital diaphragmatic hernia	2021	8	34505	11	31.88 (15.92-57.03) [/100,000]
Congenital diaphragmatic hernia	2021	9	32845	8	24.36 (10.52-47.99) [/100,000]
Congenital diaphragmatic hernia	2021	10	32810	11	33.53 (16.74-59.98) [/100,000]
Congenital diaphragmatic hernia	2021	11	31316	14	44.71 (24.44-75.00) [/100,000]
Congenital diaphragmatic hernia	2021	12	33601	11	32.74 (16.34-58.57) [/100,000]
Congenital diaphragmatic hernia	2022	1	32051	12	37.44 (19.35-65.39) [/100,000]
Congenital diaphragmatic hernia	2022	2	28206	7	24.82 (9.98-51.13) [/100,000]
Congenital diaphragmatic hernia	2022	3	31220	8	25.62 (11.06-50.48) [/100,000]
Congenital diaphragmatic hernia	2022	4	29817	14	46.95 (25.67-78.77) [/100,000]
Congenital diaphragmatic hernia	2022	5	31433	11	35.00 (17.47-62.61) [/100,000]
Congenital diaphragmatic hernia	2022	6	32214	14	43.46 (23.76-72.91) [/100,000]
Congenital diaphragmatic hernia	2022	7	33507	11	32.83 (16.39-58.73) [/100,000]
Congenital diaphragmatic hernia	2022	8	32850	11	33.49 (16.72-59.91) [/100,000]
Congenital diaphragmatic hernia	2022	9	30974	10	32.29 (15.48-59.37) [/100,000]
Congenital diaphragmatic hernia	2022	10	32543	8	24.58 (10.61-48.43) [/100,000]
Congenital diaphragmatic hernia	2022	11	31996	9	28.13 (12.86-53.39) [/100,000]
Congenital diaphragmatic hernia	2022	12	33041	11	33.29 (16.62-59.56) [/100,000]
Omphalocele	2016	1	31578	12	38.00 (19.64-66.37) [/100,000]
Omphalocele	2016	2	29832	14	46.93 (25.66-78.73) [/100,000]

Omphalocele	2016	3	32104	17	52.95 (30.85-84.77) [/100,000]
Omphalocele	2016	4	31273	12	38.37 (19.83-67.02) [/100,000]
Omphalocele	2016	5	32674	20	61.21 (37.39-94.52) [/100,000]
Omphalocele	2016	6	32921	13	39.49 (21.03-67.52) [/100,000]
Omphalocele	2016	7	34139	11	32.22 (16.09-57.65) [/100,000]
Omphalocele	2016	8	34122	13	38.10 (20.29-65.14) [/100,000]
Omphalocele	2016	9	32134	9	28.01 (12.81-53.16) [/100,000]
Omphalocele	2016	10	32377	9	27.80 (12.71-52.76) [/100,000]
Omphalocele	2016	11	31275	7	22.38 (9.00-46.11) [/100,000]
Omphalocele	2016	12	32940	19	57.68 (34.73-90.06) [/100,000]
Omphalocele	2017	1	31868	13	40.79 (21.72-69.75) [/100,000]
Omphalocele	2017	2	28411	15	52.80 (29.55-87.06) [/100,000]
Omphalocele	2017	3	31453	17	54.05 (31.49-86.52) [/100,000]
Omphalocele	2017	4	29890	15	50.18 (28.09-82.76) [/100,000]
Omphalocele	2017	5	32190	14	43.49 (23.78-72.96) [/100,000]
Omphalocele	2017	6	32693	15	45.88 (25.68-75.66) [/100,000]
Omphalocele	2017	7	33660	13	38.62 (20.57-66.03) [/100,000]
Omphalocele	2017	8	34410	19	55.22 (33.25-86.21) [/100,000]
Omphalocele	2017	9	31997	12	37.50 (19.38-65.50) [/100,000]
Omphalocele	2017	10	32373	14	43.25 (23.64-72.55) [/100,000]
Omphalocele	2017	11	31107	11	35.36 (17.65-63.26) [/100,000]
Omphalocele	2017	12	32153	19	59.09 (35.58-92.26) [/100,000]
Omphalocele	2018	1	31897	22	68.97 (43.23-104.41) [/100,000]
Omphalocele	2018	2	27999	13	46.43 (24.72-79.38) [/100,000]
Omphalocele	2018	3	31093	18	57.89 (34.31-91.48) [/100,000]
Omphalocele	2018	4	29726	13	43.73 (23.29-74.77) [/100,000]
Omphalocele	2018	5	32506	12	36.92 (19.08-64.48) [/100,000]
Omphalocele	2018	6	31835	17	53.40 (31.11-85.49) [/100,000]
Omphalocele	2018	7	33193	9	27.11 (12.40-51.46) [/100,000]
Omphalocele	2018	8	33603	18	53.57 (31.75-84.65) [/100,000]
Omphalocele	2018	9	31319	17	54.28 (31.62-86.89) [/100,000]
Omphalocele	2018	10	32726	15	45.84 (25.66-75.59) [/100,000]
Omphalocele	2018	11	31223	19	60.85 (36.64-95.01) [/100,000]
Omphalocele	2018	12	31948	7	21.91 (8.81-45.14) [/100,000]
Omphalocele	2019	1	31726	15	47.28 (26.46-77.97) [/100,000]
Omphalocele	2019	2	28476	15	52.68 (29.49-86.87) [/100,000]
Omphalocele	2019	3	31269	13	41.57 (22.14-71.08) [/100,000]
Omphalocele	2019	4	30256	15	49.58 (27.75-81.76) [/100,000]
Omphalocele	2019	5	32428	12	37.01 (19.12-64.63) [/100,000]
Omphalocele	2019	6	31895	14	43.89 (24.00-73.64) [/100,000]
Omphalocele	2019	7	34331	15	43.69 (24.46-72.05) [/100,000]
Omphalocele	2019	8	34301	17	49.56 (28.87-79.34) [/100,000]
Omphalocele	2019	9	31432	14	44.54 (24.35-74.72) [/100,000]
Omphalocele	2019	10	32919	17	51.64 (30.09-82.67) [/100,000]
Omphalocele	2019	11	31181	16	51.31 (29.33-83.32) [/100,000]
Omphalocele	2019	12	32305	14	43.34 (23.69-72.70) [/100,000]
Omphalocele	2020	1	32060	12	37.43 (19.34-65.37) [/100,000]
Omphalocele	2020	2	29406	9	30.61 (14.00-58.09) [/100,000]
Omphalocele	2020	3	29632	15	50.62 (28.33-83.48) [/100,000]
Omphalocele	2020	4	28488	8	28.08 (12.12-55.33) [/100,000]
Omphalocele	2020	5	30326	12	39.57 (20.45-69.11) [/100,000]
Omphalocele	2020	6	30775	8	26.00 (11.22-51.21) [/100,000]
Omphalocele	2020	7	32621	12	36.79 (19.01-64.25) [/100,000]
Omphalocele	2020	8	32042	11	34.33 (17.14-61.42) [/100,000]
Omphalocele	2020	9	29949	18	60.10 (35.62-94.97) [/100,000]
Omphalocele	2020	10	30948	12	38.77 (20.04-67.72) [/100,000]
Omphalocele	2020	11	28169	9	31.95 (14.61-60.64) [/100,000]
Omphalocele	2020	12	29569	10	33.82 (16.22-62.19) [/100,000]
Omphalocele	2021	1	30429	21	69.01 (42.73-105.47) [/100,000]
Omphalocele	2021	2	27741	12	43.26 (22.35-75.55) [/100,000]
Omphalocele	2021	3	30839	5	16.21 (5.26-37.83) [/100,000]
Omphalocele	2021	4	30961	15	48.45 (27.12-79.90) [/100,000]
Omphalocele	2021	5	31530	12	38.06 (19.67-66.47) [/100,000]
Omphalocele	2021	6	32665	17	52.04 (30.32-83.31) [/100,000]
Omphalocele	2021	7	33868	12	35.43 (18.31-61.88) [/100,000]
Omphalocele	2021	8	34505	14	40.57 (22.18-68.07) [/100,000]
Omphalocele	2021	9	32845	20	60.89 (37.20-94.03) [/100,000]
Omphalocele	2021	10	32810	15	45.72 (25.59-75.39) [/100,000]
Omphalocele	2021	11	31316	3	9.58 (1.98-27.99) [/100,000]
Omphalocele	2021	12	33601	18	53.57 (31.75-84.65) [/100,000]
Omphalocele	2022	1	32051	13	40.56 (21.60-69.35) [/100,000]

Omphalocele	2022	2	28206	8	28.36 (12.25-55.88) [/100,000]
Omphalocele	2022	3	31220	14	44.84 (24.52-75.23) [/100,000]
Omphalocele	2022	4	29817	12	40.25 (20.80-70.29) [/100,000]
Omphalocele	2022	5	31433	11	35.00 (17.47-62.61) [/100,000]
Omphalocele	2022	6	32214	10	31.04 (14.89-57.08) [/100,000]
Omphalocele	2022	7	33507	14	41.78 (22.84-70.09) [/100,000]
Omphalocele	2022	8	32850	16	48.71 (27.84-79.08) [/100,000]
Omphalocele	2022	9	30974	17	54.88 (31.98-87.86) [/100,000]
Omphalocele	2022	10	32543	13	39.95 (21.27-68.30) [/100,000]
Omphalocele	2022	11	31996	16	50.01 (28.59-81.19) [/100,000]
Omphalocele	2022	12	33041	6	18.16 (6.66-39.52) [/100,000]
Gastroschisis	2016	1	31578	55	174.17 (131.24-226.65) [/100,000]
Gastroschisis	2016	2	29832	47	157.55 (115.78-209.45) [/100,000]
Gastroschisis	2016	3	32104	56	174.43 (131.79-226.46) [/100,000]
Gastroschisis	2016	4	31273	42	134.30 (96.81-181.49) [/100,000]
Gastroschisis	2016	5	32674	58	177.51 (134.82-229.41) [/100,000]
Gastroschisis	2016	6	32921	40	121.50 (86.82-165.42) [/100,000]
Gastroschisis	2016	7	34139	53	155.25 (116.31-203.02) [/100,000]
Gastroschisis	2016	8	34122	55	161.19 (121.45-209.76) [/100,000]
Gastroschisis	2016	9	32134	57	177.38 (134.37-229.76) [/100,000]
Gastroschisis	2016	10	32377	36	111.19 (77.89-153.90) [/100,000]
Gastroschisis	2016	11	31275	51	163.07 (121.44-214.35) [/100,000]
Gastroschisis	2016	12	32940	48	145.72 (107.46-193.16) [/100,000]
Gastroschisis	2017	1	31868	59	185.14 (140.97-238.75) [/100,000]
Gastroschisis	2017	2	28411	39	137.27 (97.63-187.61) [/100,000]
Gastroschisis	2017	3	31453	42	133.53 (96.25-180.45) [/100,000]
Gastroschisis	2017	4	29890	41	137.17 (98.45-186.04) [/100,000]
Gastroschisis	2017	5	32190	49	152.22 (112.63-201.20) [/100,000]
Gastroschisis	2017	6	32693	56	171.29 (129.42-222.38) [/100,000]
Gastroschisis	2017	7	33660	54	160.43 (120.54-209.27) [/100,000]
Gastroschisis	2017	8	34410	49	142.40 (105.37-188.22) [/100,000]
Gastroschisis	2017	9	31997	40	125.01 (89.32-170.19) [/100,000]
Gastroschisis	2017	10	32373	44	135.92 (98.77-182.42) [/100,000]
Gastroschisis	2017	11	31107	53	170.38 (127.65-222.80) [/100,000]
Gastroschisis	2017	12	32153	40	124.41 (88.89-169.37) [/100,000]
Gastroschisis	2018	1	31897	44	137.94 (100.25-185.14) [/100,000]
Gastroschisis	2018	2	27999	32	114.29 (78.19-161.31) [/100,000]
Gastroschisis	2018	3	31093	39	125.43 (89.21-171.43) [/100,000]
Gastroschisis	2018	4	29726	55	185.02 (139.41-240.77) [/100,000]
Gastroschisis	2018	5	32506	55	169.20 (127.49-220.18) [/100,000]
Gastroschisis	2018	6	31835	40	125.65 (89.78-171.06) [/100,000]
Gastroschisis	2018	7	33193	42	126.53 (91.21-171.00) [/100,000]
Gastroschisis	2018	8	33603	44	130.94 (95.16-175.74) [/100,000]
Gastroschisis	2018	9	31319	42	134.10 (96.67-181.23) [/100,000]
Gastroschisis	2018	10	32726	38	116.12 (82.18-159.34) [/100,000]
Gastroschisis	2018	11	31223	34	108.89 (75.42-152.14) [/100,000]
Gastroschisis	2018	12	31948	48	150.24 (110.80-199.15) [/100,000]
Gastroschisis	2019	1	31726	41	129.23 (92.75-175.28) [/100,000]
Gastroschisis	2019	2	28476	48	168.56 (124.31-223.43) [/100,000]
Gastroschisis	2019	3	31269	51	163.10 (121.46-214.39) [/100,000]
Gastroschisis	2019	4	30256	31	102.46 (69.63-145.40) [/100,000]
Gastroschisis	2019	5	32428	45	138.77 (101.24-185.64) [/100,000]
Gastroschisis	2019	6	31895	38	119.14 (84.32-163.49) [/100,000]
Gastroschisis	2019	7	34331	38	110.69 (78.34-151.90) [/100,000]
Gastroschisis	2019	8	34301	47	137.02 (100.70-182.17) [/100,000]
Gastroschisis	2019	9	31432	51	162.26 (120.83-213.28) [/100,000]
Gastroschisis	2019	10	32919	43	130.62 (94.55-175.91) [/100,000]
Gastroschisis	2019	11	31181	38	121.87 (86.26-167.24) [/100,000]
Gastroschisis	2019	12	32305	45	139.30 (101.62-186.35) [/100,000]
Gastroschisis	2020	1	32060	33	102.93 (70.86-144.52) [/100,000]
Gastroschisis	2020	2	29406	38	129.23 (91.46-177.33) [/100,000]
Gastroschisis	2020	3	29632	36	121.49 (85.10-168.15) [/100,000]
Gastroschisis	2020	4	28488	42	147.43 (106.27-199.23) [/100,000]
Gastroschisis	2020	5	30326	46	151.69 (111.07-202.28) [/100,000]
Gastroschisis	2020	6	30775	41	133.23 (95.62-180.69) [/100,000]
Gastroschisis	2020	7	32621	37	113.42 (79.87-156.31) [/100,000]
Gastroschisis	2020	8	32042	36	112.35 (78.70-155.51) [/100,000]
Gastroschisis	2020	9	29949	37	123.54 (87.00-170.25) [/100,000]
Gastroschisis	2020	10	30948	46	148.64 (108.84-198.21) [/100,000]
Gastroschisis	2020	11	28169	41	145.55 (104.47-197.40) [/100,000]
Gastroschisis	2020	12	29569	41	138.66 (99.52-188.06) [/100,000]

Gastroschisis	2021	1	30429	47	154.46 (113.51-205.34) [/100,000]
Gastroschisis	2021	2	27741	35	126.17 (87.90-175.42) [/100,000]
Gastroschisis	2021	3	30839	37	119.98 (84.49-165.34) [/100,000]
Gastroschisis	2021	4	30961	28	90.44 (60.10-130.68) [/100,000]
Gastroschisis	2021	5	31530	46	145.89 (106.83-194.55) [/100,000]
Gastroschisis	2021	6	32665	44	134.70 (97.89-180.79) [/100,000]
Gastroschisis	2021	7	33868	41	121.06 (86.89-164.19) [/100,000]
Gastroschisis	2021	8	34505	37	107.23 (75.51-147.77) [/100,000]
Gastroschisis	2021	9	32845	39	118.74 (84.45-162.29) [/100,000]
Gastroschisis	2021	10	32810	42	128.01 (92.27-172.99) [/100,000]
Gastroschisis	2021	11	31316	25	79.83 (51.67-117.82) [/100,000]
Gastroschisis	2021	12	33601	49	145.83 (107.90-192.75) [/100,000]
Gastroschisis	2022	1	32051	41	127.92 (91.81-173.50) [/100,000]
Gastroschisis	2022	2	28206	43	152.45 (110.35-205.29) [/100,000]
Gastroschisis	2022	3	31220	31	99.30 (67.48-140.91) [/100,000]
Gastroschisis	2022	4	29817	27	90.55 (59.68-131.72) [/100,000]
Gastroschisis	2022	5	31433	35	111.35 (77.57-154.82) [/100,000]
Gastroschisis	2022	6	32214	35	108.65 (75.69-151.07) [/100,000]
Gastroschisis	2022	7	33507	29	86.55 (57.97-124.28) [/100,000]
Gastroschisis	2022	8	32850	29	88.28 (59.13-126.76) [/100,000]
Gastroschisis	2022	9	30974	29	93.63 (62.71-134.44) [/100,000]
Gastroschisis	2022	10	32543	36	110.62 (77.49-153.12) [/100,000]
Gastroschisis	2022	11	31996	32	100.01 (68.42-141.16) [/100,000]
Gastroschisis	2022	12	33041	24	72.64 (46.55-108.06) [/100,000]
Limb reduction defect	2016	1	31578	8	25.33 (10.94-49.91) [/100,000]
Limb reduction defect	2016	2	29832	12	40.23 (20.79-70.25) [/100,000]
Limb reduction defect	2016	3	32104	11	34.26 (17.11-61.30) [/100,000]
Limb reduction defect	2016	4	31273	7	22.38 (9.00-46.11) [/100,000]
Limb reduction defect	2016	5	32674	10	30.61 (14.68-56.28) [/100,000]
Limb reduction defect	2016	6	32921	16	48.60 (27.78-78.91) [/100,000]
Limb reduction defect	2016	7	34139	15	43.94 (24.59-72.46) [/100,000]
Limb reduction defect	2016	8	34122	18	52.75 (31.27-83.36) [/100,000]
Limb reduction defect	2016	9	32134	11	34.23 (17.09-61.24) [/100,000]
Limb reduction defect	2016	10	32377	7	21.62 (8.69-44.54) [/100,000]
Limb reduction defect	2016	11	31275	11	35.17 (17.56-62.92) [/100,000]
Limb reduction defect	2016	12	32940	12	36.43 (18.83-63.63) [/100,000]
Limb reduction defect	2017	1	31868	10	31.38 (15.05-57.70) [/100,000]
Limb reduction defect	2017	2	28411	9	31.68 (14.49-60.13) [/100,000]
Limb reduction defect	2017	3	31453	15	47.69 (26.69-78.65) [/100,000]
Limb reduction defect	2017	4	29890	10	33.46 (16.04-61.52) [/100,000]
Limb reduction defect	2017	5	32190	14	43.49 (23.78-72.96) [/100,000]
Limb reduction defect	2017	6	32693	21	64.23 (39.77-98.17) [/100,000]
Limb reduction defect	2017	7	33660	11	32.68 (16.31-58.47) [/100,000]
Limb reduction defect	2017	8	34410	9	26.16 (11.96-49.64) [/100,000]
Limb reduction defect	2017	9	31997	7	21.88 (8.80-45.07) [/100,000]
Limb reduction defect	2017	10	32373	11	33.98 (16.96-60.79) [/100,000]
Limb reduction defect	2017	11	31107	13	41.79 (22.25-71.45) [/100,000]
Limb reduction defect	2017	12	32153	11	34.21 (17.08-61.21) [/100,000]
Limb reduction defect	2018	1	31897	15	47.03 (26.32-77.55) [/100,000]
Limb reduction defect	2018	2	27999	6	21.43 (7.86-46.64) [/100,000]
Limb reduction defect	2018	3	31093	11	35.38 (17.66-63.29) [/100,000]
Limb reduction defect	2018	4	29726	9	30.28 (13.85-57.47) [/100,000]
Limb reduction defect	2018	5	32506	9	27.69 (12.66-52.55) [/100,000]
Limb reduction defect	2018	6	31835	13	40.84 (21.74-69.82) [/100,000]
Limb reduction defect	2018	7	33193	9	27.11 (12.40-51.46) [/100,000]
Limb reduction defect	2018	8	33603	12	35.71 (18.45-62.37) [/100,000]
Limb reduction defect	2018	9	31319	8	25.54 (11.03-50.32) [/100,000]
Limb reduction defect	2018	10	32726	15	45.84 (25.66-75.59) [/100,000]
Limb reduction defect	2018	11	31223	9	28.82 (13.18-54.71) [/100,000]
Limb reduction defect	2018	12	31948	9	28.17 (12.88-53.47) [/100,000]
Limb reduction defect	2019	1	31726	9	28.37 (12.97-53.84) [/100,000]
Limb reduction defect	2019	2	28476	21	73.75 (45.66-112.71) [/100,000]
Limb reduction defect	2019	3	31269	7	22.39 (9.00-46.12) [/100,000]
Limb reduction defect	2019	4	30256	4	13.22 (3.60-33.85) [/100,000]
Limb reduction defect	2019	5	32428	7	21.59 (8.68-44.47) [/100,000]
Limb reduction defect	2019	6	31895	9	28.22 (12.90-53.56) [/100,000]
Limb reduction defect	2019	7	34331	12	34.95 (18.06-61.05) [/100,000]
Limb reduction defect	2019	8	34301	10	29.15 (13.98-53.61) [/100,000]
Limb reduction defect	2019	9	31432	8	25.45 (10.99-50.14) [/100,000]
Limb reduction defect	2019	10	32919	12	36.45 (18.84-63.67) [/100,000]
Limb reduction defect	2019	11	31181	11	35.28 (17.61-63.11) [/100,000]

Limb reduction defect	2019	12	32305	11	34.05 (17.00-60.92) [/100,000]
Limb reduction defect	2020	1	32060	13	40.55 (21.59-69.33) [/100,000]
Limb reduction defect	2020	2	29406	19	64.61 (38.91-100.88) [/100,000]
Limb reduction defect	2020	3	29632	10	33.75 (16.18-62.05) [/100,000]
Limb reduction defect	2020	4	28488	8	28.08 (12.12-55.33) [/100,000]
Limb reduction defect	2020	5	30326	5	16.49 (5.35-38.47) [/100,000]
Limb reduction defect	2020	6	30775	7	22.75 (9.15-46.86) [/100,000]
Limb reduction defect	2020	7	32621	10	30.66 (14.70-56.37) [/100,000]
Limb reduction defect	2020	8	32042	12	37.45 (19.35-65.41) [/100,000]
Limb reduction defect	2020	9	29949	12	40.07 (20.71-69.98) [/100,000]
Limb reduction defect	2020	10	30948	12	38.77 (20.04-67.72) [/100,000]
Limb reduction defect	2020	11	28169	5	17.75 (5.76-41.42) [/100,000]
Limb reduction defect	2020	12	29569	7	23.67 (9.52-48.77) [/100,000]
Limb reduction defect	2021	1	30429	3	9.86 (2.03-28.81) [/100,000]
Limb reduction defect	2021	2	27741	9	32.44 (14.84-61.58) [/100,000]
Limb reduction defect	2021	3	30839	9	29.18 (13.35-55.39) [/100,000]
Limb reduction defect	2021	4	30961	7	22.61 (9.09-46.58) [/100,000]
Limb reduction defect	2021	5	31530	7	22.20 (8.93-45.74) [/100,000]
Limb reduction defect	2021	6	32665	14	42.86 (23.43-71.90) [/100,000]
Limb reduction defect	2021	7	33868	14	41.34 (22.60-69.35) [/100,000]
Limb reduction defect	2021	8	34505	8	23.19 (10.01-45.68) [/100,000]
Limb reduction defect	2021	9	32845	8	24.36 (10.52-47.99) [/100,000]
Limb reduction defect	2021	10	32810	4	12.19 (3.32-31.21) [/100,000]
Limb reduction defect	2021	11	31316	12	38.32 (19.80-66.93) [/100,000]
Limb reduction defect	2021	12	33601	10	29.76 (14.27-54.72) [/100,000]
Limb reduction defect	2022	1	32051	7	21.84 (8.78-44.99) [/100,000]
Limb reduction defect	2022	2	28206	7	24.82 (9.98-51.13) [/100,000]
Limb reduction defect	2022	3	31220	9	28.83 (13.18-54.72) [/100,000]
Limb reduction defect	2022	4	29817	6	20.12 (7.39-43.79) [/100,000]
Limb reduction defect	2022	5	31433	6	19.09 (7.01-41.54) [/100,000]
Limb reduction defect	2022	6	32214	6	18.63 (6.84-40.54) [/100,000]
Limb reduction defect	2022	7	33507	9	26.86 (12.28-50.98) [/100,000]
Limb reduction defect	2022	8	32850	10	30.44 (14.60-55.98) [/100,000]
Limb reduction defect	2022	9	30974	8	25.83 (11.15-50.89) [/100,000]
Limb reduction defect	2022	10	32543	11	33.80 (16.87-60.47) [/100,000]
Limb reduction defect	2022	11	31996	11	34.38 (17.16-61.51) [/100,000]
Limb reduction defect	2022	12	33041	7	21.19 (8.52-43.65) [/100,000]
Cleft Lip with or without Cleft Palate	2016	1	31578	31	98.17 (66.71-139.32) [/100,000]
Cleft Lip with or without Cleft Palate	2016	2	29832	23	77.10 (48.88-115.66) [/100,000]
Cleft Lip with or without Cleft Palate	2016	3	32104	27	84.10 (55.43-122.34) [/100,000]
Cleft Lip with or without Cleft Palate	2016	4	31273	34	108.72 (75.30-151.89) [/100,000]
Cleft Lip with or without Cleft Palate	2016	5	32674	35	107.12 (74.62-148.95) [/100,000]
Cleft Lip with or without Cleft Palate	2016	6	32921	29	88.09 (59.00-126.49) [/100,000]
Cleft Lip with or without Cleft Palate	2016	7	34139	32	93.73 (64.12-132.30) [/100,000]
Cleft Lip with or without Cleft Palate	2016	8	34122	30	87.92 (59.33-125.49) [/100,000]
Cleft Lip with or without Cleft Palate	2016	9	32134	38	118.25 (83.70-162.28) [/100,000]
Cleft Lip with or without Cleft Palate	2016	10	32377	27	83.39 (54.96-121.31) [/100,000]
Cleft Lip with or without Cleft Palate	2016	11	31275	24	76.74 (49.17-114.16) [/100,000]
Cleft Lip with or without Cleft Palate	2016	12	32940	35	106.25 (74.02-147.74) [/100,000]
Cleft Lip with or without Cleft Palate	2017	1	31868	38	119.24 (84.40-163.63) [/100,000]
Cleft Lip with or without Cleft Palate	2017	2	28411	23	80.95 (51.32-121.45) [/100,000]
Cleft Lip with or without Cleft Palate	2017	3	31453	30	95.38 (64.36-136.13) [/100,000]
Cleft Lip with or without Cleft Palate	2017	4	29890	26	86.99 (56.83-127.43) [/100,000]
Cleft Lip with or without Cleft Palate	2017	5	32190	28	86.98 (57.81-125.69) [/100,000]
Cleft Lip with or without Cleft Palate	2017	6	32693	33	100.94 (69.49-141.73) [/100,000]
Cleft Lip with or without Cleft Palate	2017	7	33660	40	118.84 (84.91-161.79) [/100,000]
Cleft Lip with or without Cleft Palate	2017	8	34410	29	84.28 (56.45-121.01) [/100,000]
Cleft Lip with or without Cleft Palate	2017	9	31997	29	90.63 (60.71-130.14) [/100,000]
Cleft Lip with or without Cleft Palate	2017	10	32373	38	117.38 (83.08-161.08) [/100,000]
Cleft Lip with or without Cleft Palate	2017	11	31107	22	70.72 (44.33-107.06) [/100,000]
Cleft Lip with or without Cleft Palate	2017	12	32153	27	83.97 (55.35-122.15) [/100,000]
Cleft Lip with or without Cleft Palate	2018	1	31897	34	106.59 (73.83-148.92) [/100,000]
Cleft Lip with or without Cleft Palate	2018	2	27999	23	82.15 (52.08-123.23) [/100,000]
Cleft Lip with or without Cleft Palate	2018	3	31093	33	106.13 (73.07-149.02) [/100,000]
Cleft Lip with or without Cleft Palate	2018	4	29726	34	114.38 (79.22-159.80) [/100,000]
Cleft Lip with or without Cleft Palate	2018	5	32506	29	89.21 (59.76-128.10) [/100,000]
Cleft Lip with or without Cleft Palate	2018	6	31835	28	87.95 (58.45-127.09) [/100,000]
Cleft Lip with or without Cleft Palate	2018	7	33193	36	108.46 (75.97-150.12) [/100,000]
Cleft Lip with or without Cleft Palate	2018	8	33603	24	71.42 (45.77-106.25) [/100,000]
Cleft Lip with or without Cleft Palate	2018	9	31319	28	89.40 (59.42-129.19) [/100,000]
Cleft Lip with or without Cleft Palate	2018	10	32726	37	113.06 (79.62-155.80) [/100,000]

Cleft Lip with or without Cleft Palate	2018	11	31223	20	64.06 (39.13-98.91) [/100,000]
Cleft Lip with or without Cleft Palate	2018	12	31948	27	84.51 (55.70-122.94) [/100,000]
Cleft Lip with or without Cleft Palate	2019	1	31726	29	91.41 (61.23-131.25) [/100,000]
Cleft Lip with or without Cleft Palate	2019	2	28476	25	87.79 (56.82-129.57) [/100,000]
Cleft Lip with or without Cleft Palate	2019	3	31269	18	57.57 (34.12-90.96) [/100,000]
Cleft Lip with or without Cleft Palate	2019	4	30256	25	82.63 (53.48-121.95) [/100,000]
Cleft Lip with or without Cleft Palate	2019	5	32428	26	80.18 (52.38-117.46) [/100,000]
Cleft Lip with or without Cleft Palate	2019	6	31895	41	128.55 (92.26-174.35) [/100,000]
Cleft Lip with or without Cleft Palate	2019	7	34331	26	75.73 (49.48-110.95) [/100,000]
Cleft Lip with or without Cleft Palate	2019	8	34301	39	113.70 (80.86-155.40) [/100,000]
Cleft Lip with or without Cleft Palate	2019	9	31432	26	82.72 (54.04-121.18) [/100,000]
Cleft Lip with or without Cleft Palate	2019	10	32919	29	88.10 (59.01-126.49) [/100,000]
Cleft Lip with or without Cleft Palate	2019	11	31181	26	83.38 (54.48-122.15) [/100,000]
Cleft Lip with or without Cleft Palate	2019	12	32305	31	95.96 (65.21-136.18) [/100,000]
Cleft Lip with or without Cleft Palate	2020	1	32060	30	93.57 (63.14-133.56) [/100,000]
Cleft Lip with or without Cleft Palate	2020	2	29406	24	81.62 (52.30-121.41) [/100,000]
Cleft Lip with or without Cleft Palate	2020	3	29632	24	80.99 (51.90-120.49) [/100,000]
Cleft Lip with or without Cleft Palate	2020	4	28488	21	73.72 (45.64-112.66) [/100,000]
Cleft Lip with or without Cleft Palate	2020	5	30326	27	89.03 (58.68-129.51) [/100,000]
Cleft Lip with or without Cleft Palate	2020	6	30775	33	107.23 (73.82-150.56) [/100,000]
Cleft Lip with or without Cleft Palate	2020	7	32621	26	79.70 (52.07-116.76) [/100,000]
Cleft Lip with or without Cleft Palate	2020	8	32042	26	81.14 (53.01-118.87) [/100,000]
Cleft Lip with or without Cleft Palate	2020	9	29949	25	83.48 (54.03-123.20) [/100,000]
Cleft Lip with or without Cleft Palate	2020	10	30948	28	90.47 (60.13-130.73) [/100,000]
Cleft Lip with or without Cleft Palate	2020	11	28169	26	92.30 (60.30-135.21) [/100,000]
Cleft Lip with or without Cleft Palate	2020	12	29569	26	87.93 (57.45-128.81) [/100,000]
Cleft Lip with or without Cleft Palate	2021	1	30429	25	82.16 (53.18-121.26) [/100,000]
Cleft Lip with or without Cleft Palate	2021	2	27741	31	111.75 (75.94-158.58) [/100,000]
Cleft Lip with or without Cleft Palate	2021	3	30839	39	126.46 (89.94-172.84) [/100,000]
Cleft Lip with or without Cleft Palate	2021	4	30961	32	103.36 (70.71-145.88) [/100,000]
Cleft Lip with or without Cleft Palate	2021	5	31530	29	91.98 (61.61-132.07) [/100,000]
Cleft Lip with or without Cleft Palate	2021	6	32665	34	104.09 (72.09-145.42) [/100,000]
Cleft Lip with or without Cleft Palate	2021	7	33868	46	135.82 (99.45-181.13) [/100,000]
Cleft Lip with or without Cleft Palate	2021	8	34505	27	78.25 (51.57-113.83) [/100,000]
Cleft Lip with or without Cleft Palate	2021	9	32845	28	85.25 (56.65-123.19) [/100,000]
Cleft Lip with or without Cleft Palate	2021	10	32810	21	64.00 (39.62-97.82) [/100,000]
Cleft Lip with or without Cleft Palate	2021	11	31316	21	67.06 (41.51-102.49) [/100,000]
Cleft Lip with or without Cleft Palate	2021	12	33601	22	65.47 (41.04-99.11) [/100,000]
Cleft Lip with or without Cleft Palate	2022	1	32051	31	96.72 (65.73-137.26) [/100,000]
Cleft Lip with or without Cleft Palate	2022	2	28206	22	78.00 (48.89-118.07) [/100,000]
Cleft Lip with or without Cleft Palate	2022	3	31220	40	128.12 (91.55-174.43) [/100,000]
Cleft Lip with or without Cleft Palate	2022	4	29817	24	80.49 (51.58-119.74) [/100,000]
Cleft Lip with or without Cleft Palate	2022	5	31433	25	79.53 (51.48-117.39) [/100,000]
Cleft Lip with or without Cleft Palate	2022	6	32214	22	68.29 (42.80-103.38) [/100,000]
Cleft Lip with or without Cleft Palate	2022	7	33507	26	77.60 (50.69-113.68) [/100,000]
Cleft Lip with or without Cleft Palate	2022	8	32850	31	94.37 (64.13-133.92) [/100,000]
Cleft Lip with or without Cleft Palate	2022	9	30974	24	77.48 (49.65-115.27) [/100,000]
Cleft Lip with or without Cleft Palate	2022	10	32543	24	73.75 (47.26-109.71) [/100,000]
Cleft Lip with or without Cleft Palate	2022	11	31996	29	90.64 (60.71-130.14) [/100,000]
Cleft Lip with or without Cleft Palate	2022	12	33041	24	72.64 (46.55-108.06) [/100,000]
Cleft Palate alone	2016	1	31578	9	28.50 (13.03-54.10) [/100,000]
Cleft Palate alone	2016	2	29832	8	26.82 (11.58-52.83) [/100,000]
Cleft Palate alone	2016	3	32104	10	31.15 (14.94-57.28) [/100,000]
Cleft Palate alone	2016	4	31273	18	57.56 (34.12-90.95) [/100,000]
Cleft Palate alone	2016	5	32674	15	45.91 (25.70-75.71) [/100,000]
Cleft Palate alone	2016	6	32921	13	39.49 (21.03-67.52) [/100,000]
Cleft Palate alone	2016	7	34139	8	23.43 (10.12-46.17) [/100,000]
Cleft Palate alone	2016	8	34122	12	35.17 (18.17-61.42) [/100,000]
Cleft Palate alone	2016	9	32134	11	34.23 (17.09-61.24) [/100,000]
Cleft Palate alone	2016	10	32377	10	30.89 (14.81-56.79) [/100,000]
Cleft Palate alone	2016	11	31275	13	41.57 (22.13-71.07) [/100,000]
Cleft Palate alone	2016	12	32940	16	48.57 (27.77-78.87) [/100,000]
Cleft Palate alone	2017	1	31868	12	37.66 (19.46-65.77) [/100,000]
Cleft Palate alone	2017	2	28411	10	35.20 (16.88-64.72) [/100,000]
Cleft Palate alone	2017	3	31453	21	66.77 (41.33-102.04) [/100,000]
Cleft Palate alone	2017	4	29890	13	43.49 (23.16-74.36) [/100,000]
Cleft Palate alone	2017	5	32190	9	27.96 (12.79-53.07) [/100,000]
Cleft Palate alone	2017	6	32693	9	27.53 (12.59-52.25) [/100,000]
Cleft Palate alone	2017	7	33660	19	56.45 (33.99-88.13) [/100,000]
Cleft Palate alone	2017	8	34410	17	49.40 (28.78-79.09) [/100,000]
Cleft Palate alone	2017	9	31997	23	71.88 (45.57-107.84) [/100,000]

Cleft Palate alone	2017	10	32373	12	37.07 (19.15-64.74) [/100,000]
Cleft Palate alone	2017	11	31107	16	51.44 (29.40-83.51) [/100,000]
Cleft Palate alone	2017	12	32153	13	40.43 (21.53-69.13) [/100,000]
Cleft Palate alone	2018	1	31897	15	47.03 (26.32-77.55) [/100,000]
Cleft Palate alone	2018	2	27999	20	71.43 (43.64-110.30) [/100,000]
Cleft Palate alone	2018	3	31093	13	41.81 (22.26-71.49) [/100,000]
Cleft Palate alone	2018	4	29726	9	30.28 (13.85-57.47) [/100,000]
Cleft Palate alone	2018	5	32506	16	49.22 (28.14-79.92) [/100,000]
Cleft Palate alone	2018	6	31835	15	47.12 (26.37-77.70) [/100,000]
Cleft Palate alone	2018	7	33193	12	36.15 (18.68-63.14) [/100,000]
Cleft Palate alone	2018	8	33603	16	47.61 (27.22-77.31) [/100,000]
Cleft Palate alone	2018	9	31319	11	35.12 (17.53-62.84) [/100,000]
Cleft Palate alone	2018	10	32726	19	58.06 (34.96-90.65) [/100,000]
Cleft Palate alone	2018	11	31223	11	35.23 (17.59-63.03) [/100,000]
Cleft Palate alone	2018	12	31948	16	50.08 (28.63-81.32) [/100,000]
Cleft Palate alone	2019	1	31726	18	56.74 (33.63-89.65) [/100,000]
Cleft Palate alone	2019	2	28476	12	42.14 (21.78-73.60) [/100,000]
Cleft Palate alone	2019	3	31269	17	54.37 (31.67-87.03) [/100,000]
Cleft Palate alone	2019	4	30256	13	42.97 (22.88-73.46) [/100,000]
Cleft Palate alone	2019	5	32428	5	15.42 (5.01-35.98) [/100,000]
Cleft Palate alone	2019	6	31895	13	40.76 (21.70-69.69) [/100,000]
Cleft Palate alone	2019	7	34331	13	37.87 (20.16-64.74) [/100,000]
Cleft Palate alone	2019	8	34301	10	29.15 (13.98-53.61) [/100,000]
Cleft Palate alone	2019	9	31432	7	22.27 (8.95-45.88) [/100,000]
Cleft Palate alone	2019	10	32919	12	36.45 (18.84-63.67) [/100,000]
Cleft Palate alone	2019	11	31181	11	35.28 (17.61-63.11) [/100,000]
Cleft Palate alone	2019	12	32305	15	46.43 (25.99-76.57) [/100,000]
Cleft Palate alone	2020	1	32060	8	24.95 (10.77-49.16) [/100,000]
Cleft Palate alone	2020	2	29406	9	30.61 (14.00-58.09) [/100,000]
Cleft Palate alone	2020	3	29632	11	37.12 (18.53-66.41) [/100,000]
Cleft Palate alone	2020	4	28488	8	28.08 (12.12-55.33) [/100,000]
Cleft Palate alone	2020	5	30326	13	42.87 (22.83-73.29) [/100,000]
Cleft Palate alone	2020	6	30775	18	58.49 (34.67-92.42) [/100,000]
Cleft Palate alone	2020	7	32621	14	42.92 (23.47-72.00) [/100,000]
Cleft Palate alone	2020	8	32042	13	40.57 (21.60-69.37) [/100,000]
Cleft Palate alone	2020	9	29949	12	40.07 (20.71-69.98) [/100,000]
Cleft Palate alone	2020	10	30948	13	42.01 (22.37-71.82) [/100,000]
Cleft Palate alone	2020	11	28169	9	31.95 (14.61-60.64) [/100,000]
Cleft Palate alone	2020	12	29569	9	30.44 (13.92-57.77) [/100,000]
Cleft Palate alone	2021	1	30429	5	16.43 (5.34-38.34) [/100,000]
Cleft Palate alone	2021	2	27741	15	54.07 (30.27-89.17) [/100,000]
Cleft Palate alone	2021	3	30839	13	42.15 (22.45-72.07) [/100,000]
Cleft Palate alone	2021	4	30961	10	32.30 (15.49-59.39) [/100,000]
Cleft Palate alone	2021	5	31530	11	34.89 (17.42-62.41) [/100,000]
Cleft Palate alone	2021	6	32665	17	52.04 (30.32-83.31) [/100,000]
Cleft Palate alone	2021	7	33868	14	41.34 (22.60-69.35) [/100,000]
Cleft Palate alone	2021	8	34505	13	37.68 (20.06-64.42) [/100,000]
Cleft Palate alone	2021	9	32845	15	45.67 (25.56-75.31) [/100,000]
Cleft Palate alone	2021	10	32810	8	24.38 (10.53-48.04) [/100,000]
Cleft Palate alone	2021	11	31316	13	41.51 (22.11-70.98) [/100,000]
Cleft Palate alone	2021	12	33601	15	44.64 (24.99-73.62) [/100,000]
Cleft Palate alone	2022	1	32051	7	21.84 (8.78-44.99) [/100,000]
Cleft Palate alone	2022	2	28206	7	24.82 (9.98-51.13) [/100,000]
Cleft Palate alone	2022	3	31220	19	60.86 (36.64-95.02) [/100,000]
Cleft Palate alone	2022	4	29817	10	33.54 (16.08-61.67) [/100,000]
Cleft Palate alone	2022	5	31433	9	28.63 (13.09-54.35) [/100,000]
Cleft Palate alone	2022	6	32214	10	31.04 (14.89-57.08) [/100,000]
Cleft Palate alone	2022	7	33507	20	59.69 (36.46-92.17) [/100,000]
Cleft Palate alone	2022	8	32850	14	42.62 (23.30-71.50) [/100,000]
Cleft Palate alone	2022	9	30974	10	32.29 (15.48-59.37) [/100,000]
Cleft Palate alone	2022	10	32543	10	30.73 (14.74-56.50) [/100,000]
Cleft Palate alone	2022	11	31996	12	37.50 (19.38-65.50) [/100,000]
Cleft Palate alone	2022	12	33041	15	45.40 (25.41-74.87) [/100,000]
Down syndrome	2016	1	31578	57	180.51 (136.74-233.80) [/100,000]
Down syndrome	2016	2	29832	44	147.49 (107.19-197.95) [/100,000]
Down syndrome	2016	3	32104	37	115.25 (81.16-158.82) [/100,000]
Down syndrome	2016	4	31273	48	153.49 (113.19-203.45) [/100,000]
Down syndrome	2016	5	32674	43	131.60 (95.26-177.23) [/100,000]
Down syndrome	2016	6	32921	52	157.95 (117.99-207.08) [/100,000]
Down syndrome	2016	7	34139	58	169.89 (129.03-219.57) [/100,000]
Down syndrome	2016	8	34122	56	164.12 (124.00-213.07) [/100,000]

Down syndrome	2016	9	32134	51	158.71 (118.19-208.62) [/100,000]
Down syndrome	2016	10	32377	49	151.34 (111.98-200.03) [/100,000]
Down syndrome	2016	11	31275	56	179.06 (135.29-232.46) [/100,000]
Down syndrome	2016	12	32940	56	170.01 (128.45-220.71) [/100,000]
Down syndrome	2017	1	31868	52	163.17 (121.89-213.93) [/100,000]
Down syndrome	2017	2	28411	35	123.19 (85.82-171.29) [/100,000]
Down syndrome	2017	3	31453	39	123.99 (88.19-169.47) [/100,000]
Down syndrome	2017	4	29890	41	137.17 (98.45-186.04) [/100,000]
Down syndrome	2017	5	32190	57	177.07 (134.14-229.36) [/100,000]
Down syndrome	2017	6	32693	52	159.06 (118.81-208.53) [/100,000]
Down syndrome	2017	7	33660	55	163.40 (123.12-212.63) [/100,000]
Down syndrome	2017	8	34410	57	165.65 (125.48-214.57) [/100,000]
Down syndrome	2017	9	31997	51	159.39 (118.70-209.52) [/100,000]
Down syndrome	2017	10	32373	39	120.47 (85.68-164.65) [/100,000]
Down syndrome	2017	11	31107	52	167.16 (124.87-219.16) [/100,000]
Down syndrome	2017	12	32153	50	155.51 (115.44-204.97) [/100,000]
Down syndrome	2018	1	31897	51	159.89 (119.07-210.17) [/100,000]
Down syndrome	2018	2	27999	47	167.86 (123.36-223.16) [/100,000]
Down syndrome	2018	3	31093	46	147.94 (108.33-197.29) [/100,000]
Down syndrome	2018	4	29726	45	151.38 (110.44-202.51) [/100,000]
Down syndrome	2018	5	32506	54	166.12 (124.82-216.70) [/100,000]
Down syndrome	2018	6	31835	47	147.64 (108.50-196.28) [/100,000]
Down syndrome	2018	7	33193	46	138.58 (101.48-184.81) [/100,000]
Down syndrome	2018	8	33603	47	139.87 (102.79-185.95) [/100,000]
Down syndrome	2018	9	31319	49	156.45 (115.77-206.79) [/100,000]
Down syndrome	2018	10	32726	55	168.06 (126.63-218.70) [/100,000]
Down syndrome	2018	11	31223	56	179.35 (135.51-232.84) [/100,000]
Down syndrome	2018	12	31948	36	112.68 (78.93-155.97) [/100,000]
Down syndrome	2019	1	31726	48	151.30 (111.57-200.55) [/100,000]
Down syndrome	2019	2	28476	45	158.03 (115.29-211.40) [/100,000]
Down syndrome	2019	3	31269	51	163.10 (121.46-214.39) [/100,000]
Down syndrome	2019	4	30256	46	152.04 (111.33-202.74) [/100,000]
Down syndrome	2019	5	32428	51	157.27 (117.12-206.73) [/100,000]
Down syndrome	2019	6	31895	36	112.87 (79.07-156.23) [/100,000]
Down syndrome	2019	7	34331	47	136.90 (100.61-182.01) [/100,000]
Down syndrome	2019	8	34301	45	131.19 (95.71-175.51) [/100,000]
Down syndrome	2019	9	31432	51	162.26 (120.83-213.28) [/100,000]
Down syndrome	2019	10	32919	49	148.85 (110.14-196.74) [/100,000]
Down syndrome	2019	11	31181	44	141.11 (102.55-189.39) [/100,000]
Down syndrome	2019	12	32305	43	133.11 (96.35-179.25) [/100,000]
Down syndrome	2020	1	32060	51	159.08 (118.47-209.10) [/100,000]
Down syndrome	2020	2	29406	43	146.23 (105.85-196.92) [/100,000]
Down syndrome	2020	3	29632	52	175.49 (131.09-230.06) [/100,000]
Down syndrome	2020	4	28488	50	175.51 (130.30-231.33) [/100,000]
Down syndrome	2020	5	30326	45	148.39 (108.25-198.50) [/100,000]
Down syndrome	2020	6	30775	42	136.47 (98.38-184.43) [/100,000]
Down syndrome	2020	7	32621	50	153.28 (113.78-202.03) [/100,000]
Down syndrome	2020	8	32042	44	137.32 (99.79-184.30) [/100,000]
Down syndrome	2020	9	29949	61	203.68 (155.83-261.56) [/100,000]
Down syndrome	2020	10	30948	42	135.71 (97.83-183.40) [/100,000]
Down syndrome	2020	11	28169	56	198.80 (150.21-258.08) [/100,000]
Down syndrome	2020	12	29569	47	158.95 (116.81-211.31) [/100,000]
Down syndrome	2021	1	30429	50	164.32 (121.98-216.57) [/100,000]
Down syndrome	2021	2	27741	54	194.66 (146.27-253.91) [/100,000]
Down syndrome	2021	3	30839	43	139.43 (100.93-187.77) [/100,000]
Down syndrome	2021	4	30961	41	132.42 (95.05-179.61) [/100,000]
Down syndrome	2021	5	31530	45	142.72 (104.12-190.93) [/100,000]
Down syndrome	2021	6	32665	52	159.19 (118.91-208.71) [/100,000]
Down syndrome	2021	7	33868	56	165.35 (124.93-214.66) [/100,000]
Down syndrome	2021	8	34505	54	156.50 (117.59-204.15) [/100,000]
Down syndrome	2021	9	32845	47	143.10 (105.16-190.24) [/100,000]
Down syndrome	2021	10	32810	50	152.39 (113.13-200.86) [/100,000]
Down syndrome	2021	11	31316	46	146.89 (107.56-195.88) [/100,000]
Down syndrome	2021	12	33601	43	127.97 (92.63-172.34) [/100,000]
Down syndrome	2022	1	32051	46	143.52 (105.09-191.39) [/100,000]
Down syndrome	2022	2	28206	40	141.81 (101.33-193.06) [/100,000]
Down syndrome	2022	3	31220	54	172.97 (129.96-225.62) [/100,000]
Down syndrome	2022	4	29817	39	130.80 (93.03-178.76) [/100,000]
Down syndrome	2022	5	31433	41	130.44 (93.62-176.91) [/100,000]
Down syndrome	2022	6	32214	40	124.17 (88.72-169.05) [/100,000]
Down syndrome	2022	7	33507	54	161.16 (121.09-210.23) [/100,000]

Down syndrome	2022	8	32850	45	136.99 (99.94-183.26) [/100,000]
Down syndrome	2022	9	30974	47	151.74 (111.51-201.73) [/100,000]
Down syndrome	2022	10	32543	41	125.99 (90.43-170.88) [/100,000]
Down syndrome	2022	11	31996	36	112.51 (78.82-155.73) [/100,000]
Down syndrome	2022	12	33041	51	154.35 (114.95-202.90) [/100,000]
Suspected chromosomal disorder	2016	1	31578	38	120.34 (85.17-165.13) [/100,000]
Suspected chromosomal disorder	2016	2	29832	49	164.25 (121.54-217.09) [/100,000]
Suspected chromosomal disorder	2016	3	32104	63	196.24 (150.83-251.00) [/100,000]
Suspected chromosomal disorder	2016	4	31273	58	185.46 (140.86-239.69) [/100,000]
Suspected chromosomal disorder	2016	5	32674	52	159.15 (118.88-208.65) [/100,000]
Suspected chromosomal disorder	2016	6	32921	52	157.95 (117.99-207.08) [/100,000]
Suspected chromosomal disorder	2016	7	34139	58	169.89 (129.03-219.57) [/100,000]
Suspected chromosomal disorder	2016	8	34122	48	140.67 (103.74-186.47) [/100,000]
Suspected chromosomal disorder	2016	9	32134	37	115.14 (81.08-158.67) [/100,000]
Suspected chromosomal disorder	2016	10	32377	43	132.81 (96.13-178.85) [/100,000]
Suspected chromosomal disorder	2016	11	31275	41	131.10 (94.09-177.80) [/100,000]
Suspected chromosomal disorder	2016	12	32940	45	136.61 (99.66-182.76) [/100,000]
Suspected chromosomal disorder	2017	1	31868	42	131.79 (95.00-178.11) [/100,000]
Suspected chromosomal disorder	2017	2	28411	36	126.71 (88.76-175.38) [/100,000]
Suspected chromosomal disorder	2017	3	31453	47	149.43 (109.82-198.66) [/100,000]
Suspected chromosomal disorder	2017	4	29890	40	133.82 (95.62-182.19) [/100,000]
Suspected chromosomal disorder	2017	5	32190	52	161.54 (120.67-211.79) [/100,000]
Suspected chromosomal disorder	2017	6	32693	47	143.76 (105.65-191.13) [/100,000]
Suspected chromosomal disorder	2017	7	33660	62	184.19 (141.25-236.07) [/100,000]
Suspected chromosomal disorder	2017	8	34410	43	124.96 (90.45-168.29) [/100,000]
Suspected chromosomal disorder	2017	9	31997	51	159.39 (118.70-209.52) [/100,000]
Suspected chromosomal disorder	2017	10	32373	48	148.27 (109.34-196.54) [/100,000]
Suspected chromosomal disorder	2017	11	31107	39	125.37 (89.17-171.35) [/100,000]
Suspected chromosomal disorder	2017	12	32153	38	118.18 (83.65-162.18) [/100,000]
Suspected chromosomal disorder	2018	1	31897	52	163.02 (121.78-213.73) [/100,000]
Suspected chromosomal disorder	2018	2	27999	53	189.29 (141.82-247.53) [/100,000]
Suspected chromosomal disorder	2018	3	31093	46	147.94 (108.33-197.29) [/100,000]
Suspected chromosomal disorder	2018	4	29726	53	178.30 (133.58-233.15) [/100,000]
Suspected chromosomal disorder	2018	5	32506	52	159.97 (119.50-209.73) [/100,000]
Suspected chromosomal disorder	2018	6	31835	45	141.35 (103.12-189.10) [/100,000]
Suspected chromosomal disorder	2018	7	33193	40	120.51 (86.11-164.06) [/100,000]
Suspected chromosomal disorder	2018	8	33603	51	151.77 (113.02-199.50) [/100,000]
Suspected chromosomal disorder	2018	9	31319	48	153.26 (113.02-203.15) [/100,000]
Suspected chromosomal disorder	2018	10	32726	58	177.23 (134.60-229.05) [/100,000]
Suspected chromosomal disorder	2018	11	31223	37	118.50 (83.45-163.30) [/100,000]
Suspected chromosomal disorder	2018	12	31948	32	100.16 (68.52-141.37) [/100,000]
Suspected chromosomal disorder	2019	1	31726	54	170.21 (127.89-222.03) [/100,000]
Suspected chromosomal disorder	2019	2	28476	48	168.56 (124.31-223.43) [/100,000]
Suspected chromosomal disorder	2019	3	31269	46	147.11 (107.72-196.18) [/100,000]
Suspected chromosomal disorder	2019	4	30256	49	161.95 (119.84-214.05) [/100,000]
Suspected chromosomal disorder	2019	5	32428	47	144.94 (106.51-192.69) [/100,000]
Suspected chromosomal disorder	2019	6	31895	49	153.63 (113.68-203.06) [/100,000]
Suspected chromosomal disorder	2019	7	34331	42	122.34 (88.18-165.33) [/100,000]
Suspected chromosomal disorder	2019	8	34301	39	113.70 (80.86-155.40) [/100,000]
Suspected chromosomal disorder	2019	9	31432	49	155.89 (115.35-206.05) [/100,000]
Suspected chromosomal disorder	2019	10	32919	38	115.43 (81.70-158.41) [/100,000]
Suspected chromosomal disorder	2019	11	31181	36	115.45 (80.88-159.80) [/100,000]
Suspected chromosomal disorder	2019	12	32305	39	120.72 (85.86-165.00) [/100,000]
Suspected chromosomal disorder	2020	1	32060	41	127.89 (91.79-173.45) [/100,000]
Suspected chromosomal disorder	2020	2	29406	44	149.63 (108.74-200.82) [/100,000]
Suspected chromosomal disorder	2020	3	29632	47	158.61 (116.56-210.87) [/100,000]
Suspected chromosomal disorder	2020	4	28488	40	140.41 (100.33-191.15) [/100,000]
Suspected chromosomal disorder	2020	5	30326	41	135.20 (97.04-183.37) [/100,000]
Suspected chromosomal disorder	2020	6	30775	38	123.48 (87.39-169.44) [/100,000]
Suspected chromosomal disorder	2020	7	32621	41	125.69 (90.21-170.47) [/100,000]
Suspected chromosomal disorder	2020	8	32042	44	137.32 (99.79-184.30) [/100,000]
Suspected chromosomal disorder	2020	9	29949	54	180.31 (135.48-235.20) [/100,000]
Suspected chromosomal disorder	2020	10	30948	42	135.71 (97.83-183.40) [/100,000]
Suspected chromosomal disorder	2020	11	28169	36	127.80 (89.53-176.89) [/100,000]
Suspected chromosomal disorder	2020	12	29569	49	165.71 (122.62-219.02) [/100,000]
Suspected chromosomal disorder	2021	1	30429	39	128.17 (91.15-175.17) [/100,000]
Suspected chromosomal disorder	2021	2	27741	42	151.40 (109.14-204.59) [/100,000]
Suspected chromosomal disorder	2021	3	30839	33	107.01 (73.67-150.25) [/100,000]
Suspected chromosomal disorder	2021	4	30961	49	158.26 (117.11-209.18) [/100,000]
Suspected chromosomal disorder	2021	5	31530	46	145.89 (106.83-194.55) [/100,000]
Suspected chromosomal disorder	2021	6	32665	35	107.15 (74.64-148.99) [/100,000]

Suspected chromosomal disorder	2021	7	33868	54	159.44 (119.80-207.99) [/100,000]
Suspected chromosomal disorder	2021	8	34505	55	159.40 (120.10-207.43) [/100,000]
Suspected chromosomal disorder	2021	9	32845	50	152.23 (113.01-200.65) [/100,000]
Suspected chromosomal disorder	2021	10	32810	46	140.20 (102.66-186.96) [/100,000]
Suspected chromosomal disorder	2021	11	31316	43	137.31 (99.39-184.91) [/100,000]
Suspected chromosomal disorder	2021	12	33601	56	166.66 (125.92-216.37) [/100,000]
Suspected chromosomal disorder	2022	1	32051	38	118.56 (83.91-162.70) [/100,000]
Suspected chromosomal disorder	2022	2	28206	36	127.63 (89.41-176.65) [/100,000]
Suspected chromosomal disorder	2022	3	31220	50	160.15 (118.89-211.09) [/100,000]
Suspected chromosomal disorder	2022	4	29817	34	114.03 (78.98-159.31) [/100,000]
Suspected chromosomal disorder	2022	5	31433	27	85.90 (56.61-124.95) [/100,000]
Suspected chromosomal disorder	2022	6	32214	46	142.80 (104.56-190.42) [/100,000]
Suspected chromosomal disorder	2022	7	33507	47	140.27 (103.08-186.49) [/100,000]
Suspected chromosomal disorder	2022	8	32850	38	115.68 (81.87-158.74) [/100,000]
Suspected chromosomal disorder	2022	9	30974	33	106.54 (73.35-149.59) [/100,000]
Suspected chromosomal disorder	2022	10	32543	45	138.28 (100.88-184.98) [/100,000]
Suspected chromosomal disorder	2022	11	31996	36	112.51 (78.82-155.73) [/100,000]
Suspected chromosomal disorder	2022	12	33041	52	157.38 (117.56-206.33) [/100,000]
Hypospadias	2016	1	31578	43	136.17 (98.56-183.38) [/100,000]
Hypospadias	2016	2	29832	37	124.03 (87.34-170.92) [/100,000]
Hypospadias	2016	3	32104	45	140.17 (102.26-187.51) [/100,000]
Hypospadias	2016	4	31273	35	111.92 (77.97-155.62) [/100,000]
Hypospadias	2016	5	32674	37	113.24 (79.74-156.05) [/100,000]
Hypospadias	2016	6	32921	38	115.43 (81.70-158.40) [/100,000]
Hypospadias	2016	7	34139	35	102.52 (71.42-142.55) [/100,000]
Hypospadias	2016	8	34122	35	102.57 (71.46-142.63) [/100,000]
Hypospadias	2016	9	32134	43	133.81 (96.86-180.21) [/100,000]
Hypospadias	2016	10	32377	28	86.48 (57.47-124.97) [/100,000]
Hypospadias	2016	11	31275	40	127.90 (91.39-174.12) [/100,000]
Hypospadias	2016	12	32940	48	145.72 (107.46-193.16) [/100,000]
Hypospadias	2017	1	31868	36	112.97 (79.13-156.36) [/100,000]
Hypospadias	2017	2	28411	33	116.15 (79.97-163.08) [/100,000]
Hypospadias	2017	3	31453	31	98.56 (66.98-139.87) [/100,000]
Hypospadias	2017	4	29890	33	110.40 (76.01-155.01) [/100,000]
Hypospadias	2017	5	32190	34	105.62 (73.16-147.57) [/100,000]
Hypospadias	2017	6	32693	34	104.00 (72.03-145.30) [/100,000]
Hypospadias	2017	7	33660	44	130.72 (95.00-175.44) [/100,000]
Hypospadias	2017	8	34410	41	119.15 (85.52-161.61) [/100,000]
Hypospadias	2017	9	31997	30	93.76 (63.27-133.82) [/100,000]
Hypospadias	2017	10	32373	37	114.29 (80.49-157.50) [/100,000]
Hypospadias	2017	11	31107	24	77.15 (49.44-114.78) [/100,000]
Hypospadias	2017	12	32153	34	105.74 (73.24-147.74) [/100,000]
Hypospadias	2018	1	31897	37	116.00 (81.69-159.85) [/100,000]
Hypospadias	2018	2	27999	32	114.29 (78.19-161.31) [/100,000]
Hypospadias	2018	3	31093	36	115.78 (81.10-160.25) [/100,000]
Hypospadias	2018	4	29726	39	131.20 (93.31-179.31) [/100,000]
Hypospadias	2018	5	32506	45	138.44 (100.99-185.19) [/100,000]
Hypospadias	2018	6	31835	39	122.51 (87.13-167.43) [/100,000]
Hypospadias	2018	7	33193	27	81.34 (53.61-118.33) [/100,000]
Hypospadias	2018	8	33603	36	107.13 (75.05-148.29) [/100,000]
Hypospadias	2018	9	31319	24	76.63 (49.10-114.00) [/100,000]
Hypospadias	2018	10	32726	42	128.34 (92.51-173.44) [/100,000]
Hypospadias	2018	11	31223	30	96.08 (64.84-137.14) [/100,000]
Hypospadias	2018	12	31948	38	118.94 (84.18-163.22) [/100,000]
Hypospadias	2019	1	31726	48	151.30 (111.57-200.55) [/100,000]
Hypospadias	2019	2	28476	25	87.79 (56.82-129.57) [/100,000]
Hypospadias	2019	3	31269	29	92.74 (62.12-133.17) [/100,000]
Hypospadias	2019	4	30256	36	118.98 (83.35-164.69) [/100,000]
Hypospadias	2019	5	32428	33	101.76 (70.06-142.88) [/100,000]
Hypospadias	2019	6	31895	40	125.41 (89.61-170.74) [/100,000]
Hypospadias	2019	7	34331	32	93.21 (63.76-131.56) [/100,000]
Hypospadias	2019	8	34301	30	87.46 (59.02-124.83) [/100,000]
Hypospadias	2019	9	31432	30	95.44 (64.40-136.22) [/100,000]
Hypospadias	2019	10	32919	29	88.10 (59.01-126.49) [/100,000]
Hypospadias	2019	11	31181	35	112.25 (78.20-156.08) [/100,000]
Hypospadias	2019	12	32305	22	68.10 (42.68-103.09) [/100,000]
Hypospadias	2020	1	32060	43	134.12 (97.08-180.62) [/100,000]
Hypospadias	2020	2	29406	41	139.43 (100.07-189.10) [/100,000]
Hypospadias	2020	3	29632	26	87.74 (57.32-128.54) [/100,000]
Hypospadias	2020	4	28488	28	98.29 (65.32-142.02) [/100,000]
Hypospadias	2020	5	30326	30	98.93 (66.75-141.19) [/100,000]

Hypospadias	2020	6	30775	38	123.48 (87.39-169.44) [/100,000]
Hypospadias	2020	7	32621	40	122.62 (87.62-166.94) [/100,000]
Hypospadias	2020	8	32042	36	112.35 (78.70-155.51) [/100,000]
Hypospadias	2020	9	29949	27	90.15 (59.42-131.14) [/100,000]
Hypospadias	2020	10	30948	28	90.47 (60.13-130.73) [/100,000]
Hypospadias	2020	11	28169	32	113.60 (77.71-160.33) [/100,000]
Hypospadias	2020	12	29569	23	77.78 (49.31-116.69) [/100,000]
Hypospadias	2021	1	30429	28	92.02 (61.15-132.96) [/100,000]
Hypospadias	2021	2	27741	32	115.35 (78.91-162.80) [/100,000]
Hypospadias	2021	3	30839	31	100.52 (68.31-142.65) [/100,000]
Hypospadias	2021	4	30961	37	119.51 (84.16-164.68) [/100,000]
Hypospadias	2021	5	31530	41	130.03 (93.33-176.37) [/100,000]
Hypospadias	2021	6	32665	24	73.47 (47.08-109.30) [/100,000]
Hypospadias	2021	7	33868	33	97.44 (67.08-136.81) [/100,000]
Hypospadias	2021	8	34505	37	107.23 (75.51-147.77) [/100,000]
Hypospadias	2021	9	32845	35	106.56 (74.23-148.17) [/100,000]
Hypospadias	2021	10	32810	27	82.29 (54.24-119.71) [/100,000]
Hypospadias	2021	11	31316	22	70.25 (44.03-106.34) [/100,000]
Hypospadias	2021	12	33601	36	107.14 (75.05-148.30) [/100,000]
Hypospadias	2022	1	32051	28	87.36 (58.06-126.24) [/100,000]
Hypospadias	2022	2	28206	28	99.27 (65.97-143.44) [/100,000]
Hypospadias	2022	3	31220	42	134.53 (96.97-181.80) [/100,000]
Hypospadias	2022	4	29817	25	83.84 (54.27-123.75) [/100,000]
Hypospadias	2022	5	31433	26	82.72 (54.04-121.17) [/100,000]
Hypospadias	2022	6	32214	36	111.75 (78.28-154.68) [/100,000]
Hypospadias	2022	7	33507	30	89.53 (60.42-127.79) [/100,000]
Hypospadias	2022	8	32850	41	124.81 (89.58-169.28) [/100,000]
Hypospadias	2022	9	30974	27	87.17 (57.45-126.80) [/100,000]
Hypospadias	2022	10	32543	36	110.62 (77.49-153.12) [/100,000]
Hypospadias	2022	11	31996	26	81.26 (53.09-119.04) [/100,000]
Hypospadias	2022	12	33041	24	72.64 (46.55-108.06) [/100,000]
Transferred within 24 hours of delivery	2016	1	31633	1813	5.73 (5.48-5.99) [/100]
Transferred within 24 hours of delivery	2016	2	29921	1640	5.48 (5.23-5.75) [/100]
Transferred within 24 hours of delivery	2016	3	32259	1700	5.27 (5.03-5.52) [/100]
Transferred within 24 hours of delivery	2016	4	31378	1717	5.47 (5.22-5.73) [/100]
Transferred within 24 hours of delivery	2016	5	32778	1770	5.40 (5.16-5.65) [/100]
Transferred within 24 hours of delivery	2016	6	33059	1765	5.34 (5.10-5.59) [/100]
Transferred within 24 hours of delivery	2016	7	34258	1932	5.64 (5.40-5.89) [/100]
Transferred within 24 hours of delivery	2016	8	34225	1759	5.14 (4.91-5.38) [/100]
Transferred within 24 hours of delivery	2016	9	32217	1726	5.36 (5.11-5.61) [/100]
Transferred within 24 hours of delivery	2016	10	32468	1756	5.41 (5.16-5.66) [/100]
Transferred within 24 hours of delivery	2016	11	31383	1618	5.16 (4.91-5.41) [/100]
Transferred within 24 hours of delivery	2016	12	33056	1748	5.29 (5.05-5.53) [/100]
Transferred within 24 hours of delivery	2017	1	31962	1784	5.58 (5.33-5.84) [/100]
Transferred within 24 hours of delivery	2017	2	28481	1530	5.37 (5.11-5.64) [/100]
Transferred within 24 hours of delivery	2017	3	31546	1688	5.35 (5.11-5.60) [/100]
Transferred within 24 hours of delivery	2017	4	29949	1572	5.25 (5.00-5.51) [/100]
Transferred within 24 hours of delivery	2017	5	32255	1717	5.32 (5.08-5.57) [/100]
Transferred within 24 hours of delivery	2017	6	32775	1754	5.35 (5.11-5.60) [/100]
Transferred within 24 hours of delivery	2017	7	33739	1851	5.49 (5.25-5.73) [/100]
Transferred within 24 hours of delivery	2017	8	34492	1889	5.48 (5.24-5.72) [/100]
Transferred within 24 hours of delivery	2017	9	32066	1707	5.32 (5.08-5.57) [/100]
Transferred within 24 hours of delivery	2017	10	32438	1681	5.18 (4.94-5.43) [/100]
Transferred within 24 hours of delivery	2017	11	31181	1622	5.20 (4.96-5.45) [/100]
Transferred within 24 hours of delivery	2017	12	32221	1713	5.32 (5.07-5.57) [/100]
Transferred within 24 hours of delivery	2018	1	32003	1698	5.31 (5.06-5.56) [/100]
Transferred within 24 hours of delivery	2018	2	28092	1483	5.28 (5.02-5.55) [/100]
Transferred within 24 hours of delivery	2018	3	31180	1655	5.31 (5.06-5.56) [/100]
Transferred within 24 hours of delivery	2018	4	29832	1591	5.33 (5.08-5.59) [/100]
Transferred within 24 hours of delivery	2018	5	32615	1793	5.50 (5.25-5.75) [/100]
Transferred within 24 hours of delivery	2018	6	31958	1774	5.55 (5.30-5.81) [/100]
Transferred within 24 hours of delivery	2018	7	33328	1805	5.42 (5.18-5.66) [/100]
Transferred within 24 hours of delivery	2018	8	33713	1719	5.10 (4.87-5.34) [/100]
Transferred within 24 hours of delivery	2018	9	31395	1771	5.64 (5.39-5.90) [/100]
Transferred within 24 hours of delivery	2018	10	32796	1702	5.19 (4.95-5.44) [/100]
Transferred within 24 hours of delivery	2018	11	31306	1615	5.16 (4.92-5.41) [/100]
Transferred within 24 hours of delivery	2018	12	31996	1649	5.15 (4.91-5.40) [/100]
Transferred within 24 hours of delivery	2019	1	31855	1604	5.04 (4.80-5.28) [/100]
Transferred within 24 hours of delivery	2019	2	28555	1507	5.28 (5.02-5.54) [/100]
Transferred within 24 hours of delivery	2019	3	31356	1687	5.38 (5.13-5.64) [/100]
Transferred within 24 hours of delivery	2019	4	30323	1614	5.32 (5.07-5.58) [/100]

Transferred within 24 hours of delivery	2019	5	32496	1740	5.35 (5.11-5.60) [/100]
Transferred within 24 hours of delivery	2019	6	31985	1736	5.43 (5.18-5.68) [/100]
Transferred within 24 hours of delivery	2019	7	34414	1829	5.31 (5.08-5.56) [/100]
Transferred within 24 hours of delivery	2019	8	34375	1845	5.37 (5.13-5.61) [/100]
Transferred within 24 hours of delivery	2019	9	31486	1647	5.23 (4.99-5.48) [/100]
Transferred within 24 hours of delivery	2019	10	32982	1764	5.35 (5.11-5.60) [/100]
Transferred within 24 hours of delivery	2019	11	31251	1652	5.29 (5.04-5.54) [/100]
Transferred within 24 hours of delivery	2019	12	32351	1644	5.08 (4.84-5.33) [/100]
Transferred within 24 hours of delivery	2020	1	32107	1658	5.16 (4.92-5.41) [/100]
Transferred within 24 hours of delivery	2020	2	29465	1532	5.20 (4.95-5.46) [/100]
Transferred within 24 hours of delivery	2020	3	29662	1484	5.00 (4.76-5.26) [/100]
Transferred within 24 hours of delivery	2020	4	28528	1491	5.23 (4.97-5.49) [/100]
Transferred within 24 hours of delivery	2020	5	30384	1549	5.10 (4.85-5.35) [/100]
Transferred within 24 hours of delivery	2020	6	30822	1644	5.33 (5.09-5.59) [/100]
Transferred within 24 hours of delivery	2020	7	32675	1695	5.19 (4.95-5.43) [/100]
Transferred within 24 hours of delivery	2020	8	32093	1550	4.83 (4.60-5.07) [/100]
Transferred within 24 hours of delivery	2020	9	29970	1478	4.93 (4.69-5.18) [/100]
Transferred within 24 hours of delivery	2020	10	30999	1580	5.10 (4.85-5.35) [/100]
Transferred within 24 hours of delivery	2020	11	28196	1424	5.05 (4.80-5.31) [/100]
Transferred within 24 hours of delivery	2020	12	29594	1485	5.02 (4.77-5.27) [/100]
Transferred within 24 hours of delivery	2021	1	30492	1518	4.98 (4.74-5.23) [/100]
Transferred within 24 hours of delivery	2021	2	27800	1429	5.14 (4.88-5.41) [/100]
Transferred within 24 hours of delivery	2021	3	30923	1585	5.13 (4.88-5.38) [/100]
Transferred within 24 hours of delivery	2021	4	31043	1528	4.92 (4.68-5.17) [/100]
Transferred within 24 hours of delivery	2021	5	31571	1633	5.17 (4.93-5.42) [/100]
Transferred within 24 hours of delivery	2021	6	32719	1745	5.33 (5.09-5.58) [/100]
Transferred within 24 hours of delivery	2021	7	33955	1750	5.15 (4.92-5.39) [/100]
Transferred within 24 hours of delivery	2021	8	34590	1915	5.54 (5.30-5.78) [/100]
Transferred within 24 hours of delivery	2021	9	32926	1670	5.07 (4.84-5.31) [/100]
Transferred within 24 hours of delivery	2021	10	32883	1680	5.11 (4.87-5.35) [/100]
Transferred within 24 hours of delivery	2021	11	31361	1559	4.97 (4.73-5.22) [/100]
Transferred within 24 hours of delivery	2021	12	33634	1707	5.08 (4.84-5.32) [/100]
Transferred within 24 hours of delivery	2022	1	32105	1609	5.01 (4.78-5.26) [/100]
Transferred within 24 hours of delivery	2022	2	28254	1406	4.98 (4.73-5.24) [/100]
Transferred within 24 hours of delivery	2022	3	31231	1556	4.98 (4.74-5.23) [/100]
Transferred within 24 hours of delivery	2022	4	29830	1500	5.03 (4.78-5.28) [/100]
Transferred within 24 hours of delivery	2022	5	31447	1581	5.03 (4.79-5.27) [/100]
Transferred within 24 hours of delivery	2022	6	32236	1638	5.08 (4.84-5.33) [/100]
Transferred within 24 hours of delivery	2022	7	33551	1788	5.33 (5.09-5.57) [/100]
Transferred within 24 hours of delivery	2022	8	32865	1702	5.18 (4.94-5.42) [/100]
Transferred within 24 hours of delivery	2022	9	31058	1540	4.96 (4.72-5.21) [/100]
Transferred within 24 hours of delivery	2022	10	32657	1665	5.10 (4.86-5.34) [/100]
Transferred within 24 hours of delivery	2022	11	32085	1558	4.86 (4.62-5.10) [/100]
Transferred within 24 hours of delivery	2022	12	33131	1581	4.77 (4.54-5.01) [/100]
Death before birth-certificate registration	2016	1	31426	773	2.46 (2.29-2.64) [/100]
Death before birth-certificate registration	2016	2	29763	739	2.48 (2.31-2.67) [/100]
Death before birth-certificate registration	2016	3	32077	833	2.60 (2.43-2.78) [/100]
Death before birth-certificate registration	2016	4	31187	778	2.49 (2.32-2.67) [/100]
Death before birth-certificate registration	2016	5	32595	781	2.40 (2.23-2.57) [/100]
Death before birth-certificate registration	2016	6	32857	808	2.46 (2.29-2.63) [/100]
Death before birth-certificate registration	2016	7	34035	806	2.37 (2.21-2.54) [/100]
Death before birth-certificate registration	2016	8	34039	828	2.43 (2.27-2.60) [/100]
Death before birth-certificate registration	2016	9	32038	768	2.40 (2.23-2.57) [/100]
Death before birth-certificate registration	2016	10	32244	776	2.41 (2.24-2.58) [/100]
Death before birth-certificate registration	2016	11	31204	728	2.33 (2.17-2.51) [/100]
Death before birth-certificate registration	2016	12	32853	741	2.26 (2.10-2.42) [/100]
Death before birth-certificate registration	2017	1	31753	793	2.50 (2.33-2.68) [/100]
Death before birth-certificate registration	2017	2	28303	679	2.40 (2.22-2.58) [/100]
Death before birth-certificate registration	2017	3	31354	732	2.33 (2.17-2.51) [/100]
Death before birth-certificate registration	2017	4	29776	807	2.71 (2.53-2.90) [/100]
Death before birth-certificate registration	2017	5	32067	771	2.40 (2.24-2.58) [/100]
Death before birth-certificate registration	2017	6	32594	799	2.45 (2.29-2.63) [/100]
Death before birth-certificate registration	2017	7	33527	753	2.25 (2.09-2.41) [/100]
Death before birth-certificate registration	2017	8	34268	791	2.31 (2.15-2.47) [/100]
Death before birth-certificate registration	2017	9	31856	721	2.26 (2.10-2.43) [/100]
Death before birth-certificate registration	2017	10	32241	720	2.23 (2.07-2.40) [/100]
Death before birth-certificate registration	2017	11	31026	682	2.20 (2.04-2.37) [/100]
Death before birth-certificate registration	2017	12	32055	723	2.26 (2.10-2.42) [/100]
Death before birth-certificate registration	2018	1	31809	748	2.35 (2.19-2.52) [/100]
Death before birth-certificate registration	2018	2	27918	672	2.41 (2.23-2.59) [/100]
Death before birth-certificate registration	2018	3	30986	742	2.39 (2.23-2.57) [/100]

Death before birth-certificate registration	2018	4	29671	687	2.32 (2.15-2.49) [/100]
Death before birth-certificate registration	2018	5	32417	697	2.15 (1.99-2.31) [/100]
Death before birth-certificate registration	2018	6	31733	672	2.12 (1.96-2.28) [/100]
Death before birth-certificate registration	2018	7	33089	751	2.27 (2.11-2.44) [/100]
Death before birth-certificate registration	2018	8	33485	765	2.28 (2.13-2.45) [/100]
Death before birth-certificate registration	2018	9	31181	730	2.34 (2.18-2.52) [/100]
Death before birth-certificate registration	2018	10	32582	741	2.27 (2.12-2.44) [/100]
Death before birth-certificate registration	2018	11	31102	647	2.08 (1.92-2.25) [/100]
Death before birth-certificate registration	2018	12	31830	617	1.94 (1.79-2.10) [/100]
Death before birth-certificate registration	2019	1	31666	634	2.00 (1.85-2.16) [/100]
Death before birth-certificate registration	2019	2	28421	632	2.22 (2.06-2.40) [/100]
Death before birth-certificate registration	2019	3	31187	732	2.35 (2.18-2.52) [/100]
Death before birth-certificate registration	2019	4	30164	710	2.35 (2.19-2.53) [/100]
Death before birth-certificate registration	2019	5	32342	677	2.09 (1.94-2.26) [/100]
Death before birth-certificate registration	2019	6	31786	674	2.12 (1.96-2.28) [/100]
Death before birth-certificate registration	2019	7	34207	666	1.95 (1.80-2.10) [/100]
Death before birth-certificate registration	2019	8	34151	670	1.96 (1.82-2.11) [/100]
Death before birth-certificate registration	2019	9	31315	634	2.02 (1.87-2.19) [/100]
Death before birth-certificate registration	2019	10	32804	658	2.01 (1.86-2.16) [/100]
Death before birth-certificate registration	2019	11	31077	599	1.93 (1.78-2.09) [/100]
Death before birth-certificate registration	2019	12	32183	645	2.00 (1.85-2.16) [/100]
Death before birth-certificate registration	2020	1	31939	646	2.02 (1.87-2.18) [/100]
Death before birth-certificate registration	2020	2	29297	616	2.10 (1.94-2.27) [/100]
Death before birth-certificate registration	2020	3	29507	661	2.24 (2.07-2.42) [/100]
Death before birth-certificate registration	2020	4	28370	630	2.22 (2.05-2.40) [/100]
Death before birth-certificate registration	2020	5	30263	659	2.18 (2.02-2.35) [/100]
Death before birth-certificate registration	2020	6	30670	635	2.07 (1.91-2.24) [/100]
Death before birth-certificate registration	2020	7	32492	622	1.91 (1.77-2.07) [/100]
Death before birth-certificate registration	2020	8	31939	553	1.73 (1.59-1.88) [/100]
Death before birth-certificate registration	2020	9	29838	543	1.82 (1.67-1.98) [/100]
Death before birth-certificate registration	2020	10	30850	606	1.96 (1.81-2.13) [/100]
Death before birth-certificate registration	2020	11	28052	552	1.97 (1.81-2.14) [/100]
Death before birth-certificate registration	2020	12	29444	558	1.90 (1.74-2.06) [/100]
Death before birth-certificate registration	2021	1	30365	589	1.94 (1.79-2.10) [/100]
Death before birth-certificate registration	2021	2	27686	542	1.96 (1.80-2.13) [/100]
Death before birth-certificate registration	2021	3	30766	540	1.76 (1.61-1.91) [/100]
Death before birth-certificate registration	2021	4	30909	639	2.07 (1.91-2.23) [/100]
Death before birth-certificate registration	2021	5	31455	604	1.92 (1.77-2.08) [/100]
Death before birth-certificate registration	2021	6	32585	545	1.67 (1.54-1.82) [/100]
Death before birth-certificate registration	2021	7	33808	603	1.78 (1.65-1.93) [/100]
Death before birth-certificate registration	2021	8	34454	579	1.68 (1.55-1.82) [/100]
Death before birth-certificate registration	2021	9	32811	560	1.71 (1.57-1.85) [/100]
Death before birth-certificate registration	2021	10	32742	578	1.77 (1.63-1.91) [/100]
Death before birth-certificate registration	2021	11	31263	534	1.71 (1.57-1.86) [/100]
Death before birth-certificate registration	2021	12	33543	581	1.73 (1.60-1.88) [/100]
Death before birth-certificate registration	2022	1	31976	561	1.75 (1.61-1.90) [/100]
Death before birth-certificate registration	2022	2	28133	511	1.82 (1.66-1.98) [/100]
Death before birth-certificate registration	2022	3	31150	604	1.94 (1.79-2.10) [/100]
Death before birth-certificate registration	2022	4	29750	582	1.96 (1.80-2.12) [/100]
Death before birth-certificate registration	2022	5	31387	623	1.98 (1.83-2.15) [/100]
Death before birth-certificate registration	2022	6	32139	648	2.02 (1.87-2.18) [/100]
Death before birth-certificate registration	2022	7	33419	617	1.85 (1.70-2.00) [/100]
Death before birth-certificate registration	2022	8	32780	615	1.88 (1.73-2.03) [/100]
Death before birth-certificate registration	2022	9	30935	557	1.80 (1.66-1.95) [/100]
Death before birth-certificate registration	2022	10	32490	580	1.79 (1.64-1.94) [/100]
Death before birth-certificate registration	2022	11	31938	570	1.78 (1.64-1.94) [/100]
Death before birth-certificate registration	2022	12	32972	625	1.90 (1.75-2.05) [/100]

Note: Details about each variable are presented in Supplementary methods. Infants with missing obstetric estimate of gestation, or missing outcomes were excluded from the analyses of the specific outcomes.

Supplemental Table S15 Segmented regression (interrupted time-series) of the monthly series, 2016-2022

Subgroup	Outcome	Onset point	Pre-onset trend, RR per month (95% CI)	p	Level change, RR (95% CI)	p	Slope change, RR per month (95% CI)	p	Dispersion	Durbin-Watson
Overall	Congenital anomalies	March 2020	0.9993 (0.9987-0.9999)	0.026	1.0227 (0.9892-1.0574)	0.186	0.9994 (0.9980-1.0009)	0.456	1.13	2.13
Overall	Congenital anomalies	December 2020	0.9995 (0.9990-0.9999)	0.024	1.0360 (1.0085-1.0642)	0.010	0.9977 (0.9960-0.9993)	0.005	1.07	2.19
Overall	Anencephaly	March 2020	0.9977 (0.9948-1.0006)	0.123	0.9200 (0.8176-1.0352)	0.166	1.0092 (1.0041-1.0144)	<0.001	1.17	2.01
Overall	Anencephaly	December 2020	0.9981 (0.9956-1.0007)	0.147	0.8635 (0.7771-0.9594)	0.006	1.0173 (1.0111-1.0235)	<0.001	1.13	2.07
Overall	Meningomyelocele/Spina bifida	March 2020	0.9999 (0.9970-1.0027)	0.936	0.9592 (0.8415-1.0934)	0.533	0.9991 (0.9932-1.0050)	0.755	0.97	2.12
Overall	Meningomyelocele/Spina bifida	December 2020	0.9989 (0.9967-1.0011)	0.326	1.0628 (0.9515-1.1872)	0.281	0.9952 (0.9893-1.0012)	0.114	0.97	2.07
Overall	Cyanotic congenital heart disease	March 2020	1.0018 (1.0004-1.0032)	0.014	1.1149 (1.0636-1.1686)	<0.001	0.9982 (0.9958-1.0007)	0.168	1.05	1.84
Overall	Cyanotic congenital heart disease	December 2020	1.0027 (1.0014-1.0040)	<0.001	1.1075 (1.0330-1.1874)	0.004	0.9942 (0.9903-0.9980)	0.003	1.05	1.89
Overall	Congenital diaphragmatic hernia	March 2020	0.9995 (0.9970-1.0021)	0.722	1.0530 (0.9523-1.1644)	0.314	1.0008 (0.9966-1.0050)	0.696	1.05	2.12
Overall	Congenital diaphragmatic hernia	December 2020	1.0008 (0.9988-1.0029)	0.431	0.9397 (0.8221-1.0742)	0.362	1.0041 (0.9957-1.0125)	0.341	1.06	2.13
Overall	Omphalocele	March 2020	1.0002 (0.9975-1.0030)	0.860	0.9322 (0.8511-1.0210)	0.131	0.9997 (0.9947-1.0047)	0.901	0.96	2.22
Overall	Omphalocele	December 2020	0.9988 (0.9964-1.0012)	0.343	1.0610 (0.9309-1.2094)	0.375	0.9961 (0.9891-1.0031)	0.274	0.96	2.18
Overall	Gastroschisis	March 2020	0.9963 (0.9946-0.9981)	<0.001	1.0698 (0.9797-1.1681)	0.133	0.9917 (0.9877-0.9957)	<0.001	0.94	2.35
Overall	Gastroschisis	December 2020	0.9966 (0.9954-0.9978)	<0.001	1.0274 (0.9704-1.0877)	0.353	0.9882 (0.9840-0.9924)	<0.001	0.92	2.37
Overall	Limb reduction defect	March 2020	0.9964 (0.9931-0.9997)	0.031	0.9933 (0.8795-1.1219)	0.914	1.0029 (0.9987-1.0072)	0.180	1.00	2.07
Overall	Limb reduction defect	December 2020	0.9966 (0.9945-0.9987)	0.002	0.9878 (0.8628-1.1308)	0.858	1.0044 (0.9983-1.0104)	0.156	1.00	2.08
Overall	Cleft Lip with or without Cleft Palate	March 2020	0.9985 (0.9974-0.9996)	0.007	0.9805 (0.9017-1.0662)	0.645	1.0036 (1.0005-1.0066)	0.022	1.03	2.44
Overall	Cleft Lip with or without Cleft Palate	December 2020	0.9982 (0.9972-0.9991)	<0.001	1.0805 (1.0187-1.1461)	0.010	1.0002 (0.9968-1.0037)	0.892	1.01	2.42
Overall	Cleft Palate alone	March 2020	1.0000 (0.9986-1.0013)	0.959	1.0118 (0.9211-1.1114)	0.807	0.9979 (0.9941-1.0017)	0.277	0.93	1.94
Overall	Cleft Palate alone	December 2020	1.0003 (0.9992-1.0014)	0.573	0.9253 (0.8475-1.0103)	0.083	1.0014 (0.9958-1.0070)	0.624	0.91	1.96
Overall	Down syndrome	March 2020	1.0002 (0.9992-1.0012)	0.700	0.9991 (0.9476-1.0533)	0.973	0.9968 (0.9941-0.9995)	0.022	0.98	2.03
Overall	Down syndrome	December 2020	1.0001 (0.9994-1.0008)	0.732	0.9746 (0.9293-1.0222)	0.290	0.9967 (0.9925-1.0010)	0.129	0.97	2.00
Overall	Suspected chromosomal disorder	March 2020	1.0012 (0.9998-1.0026)	0.086	1.0196 (0.9365-1.1100)	0.655	0.9974 (0.9942-1.0007)	0.118	1.13	1.99
Overall	Suspected chromosomal disorder	December 2020	1.0010 (1.0001-1.0019)	0.028	1.0674 (1.0159-1.1215)	0.010	0.9934 (0.9900-0.9969)	<0.001	1.07	2.05
Overall	Hypospadias	March 2020	0.9973 (0.9961-0.9985)	<0.001	1.0081 (0.9384-1.0829)	0.826	1.0001 (0.9974-1.0029)	0.923	1.20	2.55
Overall	Hypospadias	December 2020	0.9975 (0.9965-0.9984)	<0.001	1.0109 (0.9632-1.0610)	0.660	0.9993 (0.9961-1.0026)	0.687	1.20	2.56
Overall	Transferred within 24 hours of delivery	March 2020	1.0010 (1.0004-1.0016)	0.001	0.9467 (0.9245-0.9694)	<0.001	1.0003 (0.9991-1.0015)	0.639	2.10	2.35
Overall	Transferred within 24 hours of delivery	December 2020	1.0001 (0.9993-1.0010)	0.725	1.0105 (0.9779-1.0441)	0.534	0.9993 (0.9981-1.0006)	0.287	2.78	1.79
Overall	Death before birth-certificate registration	March 2020	0.9964 (0.9958-0.9970)	<0.001	0.9622 (0.9226-1.0035)	0.072	1.0024 (1.0000-1.0048)	0.046	1.63	1.98
Overall	Death before birth-certificate registration	December 2020	0.9962 (0.9956-0.9968)	<0.001	0.9561 (0.9047-1.0105)	0.112	1.0050 (1.0019-1.0081)	0.002	1.54	2.12
≥37 weeks	Congenital anomalies	March 2020	0.9995 (0.9987-1.0002)	0.162	1.0155 (0.9782-1.0543)	0.420	0.9996 (0.9981-1.0010)	0.540	1.17	1.93
≥37 weeks	Congenital anomalies	December 2020	0.9996 (0.9990-1.0001)	0.118	1.0217 (0.9853-1.0594)	0.247	0.9984 (0.9964-1.0005)	0.128	1.15	1.93
≥37 weeks	Anencephaly	March 2020	0.9984 (0.9946-1.0023)	0.433	0.8904 (0.7494-1.0578)	0.187	1.0103 (1.0038-1.0169)	0.002	1.18	2.08
≥37 weeks	Anencephaly	December 2020	0.9984 (0.9955-1.0012)	0.267	0.8610 (0.7625-0.9722)	0.016	1.0190 (1.0122-1.0258)	<0.001	1.16	2.12
≥37 weeks	Meningomyelocele/Spina bifida	March 2020	0.9979 (0.9952-1.0007)	0.147	1.0187 (0.8581-1.2093)	0.832	0.9966 (0.9892-1.0041)	0.372	0.78	1.92
≥37 weeks	Meningomyelocele/Spina bifida	December 2020	0.9971 (0.9949-0.9993)	0.010	1.1814 (1.0555-1.3224)	0.004	0.9875 (0.9819-0.9932)	<0.001	0.72	2.03
≥37 weeks	Cyanotic congenital heart disease	March 2020	1.0023 (1.0006-1.0041)	0.009	1.0981 (1.0397-1.1597)	<0.001	0.9974 (0.9948-1.0001)	0.062	1.17	1.78
≥37 weeks	Cyanotic congenital heart disease	December 2020	1.0033 (1.0017-1.0049)	<0.001	1.0366 (0.9465-1.1352)	0.438	0.9961 (0.9911-1.0010)	0.118	1.23	1.77
≥37 weeks	Congenital diaphragmatic hernia	March 2020	0.9999 (0.9972-1.0026)	0.939	1.0111 (0.9087-1.1249)	0.840	1.0024 (0.9968-1.0080)	0.404	1.23	2.22
≥37 weeks	Congenital diaphragmatic hernia	December 2020	1.0007 (0.9986-1.0028)	0.513	0.9366 (0.8148-1.0765)	0.356	1.0059 (0.9960-1.0158)	0.245	1.23	2.23
≥37 weeks	Omphalocele	March 2020	0.9984 (0.9957-1.0011)	0.243	1.0015 (0.8853-1.1329)	0.981	1.0005 (0.9946-1.0064)	0.866	0.91	2.11
≥37 weeks	Omphalocele	December 2020	0.9980 (0.9957-1.0003)	0.082	1.0956 (0.9392-1.2781)	0.245	0.9964 (0.9883-1.0046)	0.389	0.90	2.13
≥37 weeks	Gastroschisis	March 2020	0.9967 (0.9933-1.0000)	0.050	0.9750 (0.8482-1.1207)	0.722	0.9886 (0.9837-0.9936)	<0.001	0.90	2.27

≥37 weeks	Gastroschisis	December 2020	0.9961 (0.9938-0.9984)	<0.001	0.8795 (0.7737-0.9997)	0.049	0.9907 (0.9843-0.9972)	0.005	0.90	2.26
≥37 weeks	Limb reduction defect	March 2020	0.9952 (0.9926-0.9978)	<0.001	1.0513 (0.9489-1.1647)	0.339	1.0046 (1.0004-1.0089)	0.032	0.79	2.50
≥37 weeks	Limb reduction defect	December 2020	0.9960 (0.9942-0.9978)	<0.001	1.0514 (0.9471-1.1671)	0.347	1.0045 (0.9989-1.0101)	0.119	0.80	2.50
≥37 weeks	Cleft Lip with or without Cleft Palate	March 2020	0.9983 (0.9968-0.9997)	0.018	0.9714 (0.8907-1.0595)	0.513	1.0047 (1.0014-1.0079)	0.005	1.06	2.39
≥37 weeks	Cleft Lip with or without Cleft Palate	December 2020	0.9980 (0.9969-0.9992)	<0.001	1.0596 (0.9896-1.1346)	0.097	1.0025 (0.9983-1.0066)	0.241	1.07	2.34
≥37 weeks	Cleft Palate alone	March 2020	1.0000 (0.9991-1.0010)	0.968	1.0199 (0.9355-1.1120)	0.654	0.9973 (0.9933-1.0013)	0.183	0.85	2.24
≥37 weeks	Cleft Palate alone	December 2020	1.0004 (0.9994-1.0014)	0.422	0.9288 (0.8481-1.0172)	0.111	1.0007 (0.9949-1.0064)	0.822	0.84	2.26
≥37 weeks	Down syndrome	March 2020	1.0006 (0.9992-1.0019)	0.413	0.9462 (0.8790-1.0185)	0.141	0.9976 (0.9940-1.0013)	0.208	1.10	2.01
≥37 weeks	Down syndrome	December 2020	1.0000 (0.9991-1.0009)	0.937	0.9381 (0.8802-0.9999)	0.049	0.9989 (0.9931-1.0048)	0.720	1.11	1.95
≥37 weeks	Suspected chromosomal disorder	March 2020	1.0025 (1.0011-1.0040)	<0.001	1.0130 (0.9127-1.1243)	0.808	0.9962 (0.9929-0.9995)	0.024	1.22	1.84
≥37 weeks	Suspected chromosomal disorder	December 2020	1.0022 (1.0012-1.0031)	<0.001	1.0594 (0.9670-1.1607)	0.216	0.9921 (0.9868-0.9975)	0.004	1.18	1.88
≥37 weeks	Hypospadias	March 2020	0.9971 (0.9960-0.9983)	<0.001	1.0128 (0.9507-1.0789)	0.694	1.0002 (0.9974-1.0029)	0.911	1.21	2.51
≥37 weeks	Hypospadias	December 2020	0.9973 (0.9963-0.9982)	<0.001	1.0233 (0.9745-1.0746)	0.356	0.9989 (0.9958-1.0021)	0.503	1.20	2.52
≥37 weeks	Transferred within 24 hours of delivery	March 2020	1.0016 (1.0007-1.0025)	<0.001	0.9361 (0.9087-0.9643)	<0.001	1.0001 (0.9987-1.0016)	0.882	1.74	2.20
≥37 weeks	Transferred within 24 hours of delivery	December 2020	1.0006 (0.9996-1.0017)	0.222	0.9986 (0.9580-1.0409)	0.946	0.9995 (0.9982-1.0008)	0.460	2.31	1.65
≥37 weeks	Death before birth-certificate registration	March 2020	0.9927 (0.9903-0.9951)	<0.001	1.0365 (0.9331-1.1513)	0.504	1.0083 (1.0038-1.0128)	<0.001	1.59	1.83
≥37 weeks	Death before birth-certificate registration	December 2020	0.9937 (0.9917-0.9958)	<0.001	1.0298 (0.9107-1.1644)	0.640	1.0098 (1.0034-1.0163)	0.003	1.60	1.82
<37 weeks	Congenital anomalies	March 2020	0.9981 (0.9975-0.9988)	<0.001	1.0572 (1.0084-1.1084)	0.021	0.9990 (0.9968-1.0012)	0.388	0.88	2.32
<37 weeks	Congenital anomalies	December 2020	0.9986 (0.9979-0.9993)	<0.001	1.0604 (1.0267-1.0952)	<0.001	0.9963 (0.9943-0.9982)	<0.001	0.86	2.50
<37 weeks	Anencephaly	March 2020	0.9957 (0.9906-1.0009)	0.107	0.9885 (0.8170-1.1960)	0.905	1.0072 (0.9989-1.0155)	0.090	1.01	2.13
<37 weeks	Anencephaly	December 2020	0.9972 (0.9930-1.0014)	0.196	0.8599 (0.7228-1.0231)	0.089	1.0148 (1.0033-1.0265)	0.011	0.99	2.14
<37 weeks	Meningomyelocele/Spina bifida	March 2020	1.0033 (0.9983-1.0083)	0.199	0.8609 (0.7171-1.0334)	0.108	1.0028 (0.9946-1.0110)	0.505	1.06	2.21
<37 weeks	Meningomyelocele/Spina bifida	December 2020	1.0022 (0.9986-1.0059)	0.231	0.8430 (0.7089-1.0023)	0.053	1.0097 (0.9992-1.0203)	0.070	1.05	2.18
<37 weeks	Cyanotic congenital heart disease	March 2020	0.9994 (0.9972-1.0016)	0.598	1.1827 (1.0366-1.3495)	0.013	1.0004 (0.9936-1.0072)	0.908	0.95	1.66
<37 weeks	Cyanotic congenital heart disease	December 2020	1.0004 (0.9989-1.0019)	0.603	1.3146 (1.1788-1.4661)	<0.001	0.9897 (0.9823-0.9972)	0.007	0.83	2.04
<37 weeks	Congenital diaphragmatic hernia	March 2020	0.9980 (0.9939-1.0023)	0.363	1.1808 (0.9676-1.4410)	0.102	0.9964 (0.9901-1.0029)	0.277	0.91	2.35
<37 weeks	Congenital diaphragmatic hernia	December 2020	1.0008 (0.9975-1.0041)	0.638	0.9334 (0.7533-1.1566)	0.529	0.9997 (0.9886-1.0110)	0.961	0.92	2.34
<37 weeks	Omphalocele	March 2020	1.0013 (0.9966-1.0060)	0.598	0.8682 (0.7431-1.0142)	0.075	0.9993 (0.9917-1.0071)	0.867	1.01	2.32
<37 weeks	Omphalocele	December 2020	0.9990 (0.9947-1.0034)	0.669	1.0099 (0.8220-1.2409)	0.925	0.9971 (0.9860-1.0083)	0.612	1.02	2.22
<37 weeks	Gastroschisis	March 2020	0.9951 (0.9934-0.9968)	<0.001	1.1510 (1.0487-1.2633)	0.003	0.9931 (0.9885-0.9976)	0.003	0.96	2.20
<37 weeks	Gastroschisis	December 2020	0.9962 (0.9949-0.9975)	<0.001	1.0957 (1.0081-1.1911)	0.032	0.9877 (0.9823-0.9932)	<0.001	0.95	2.24
<37 weeks	Limb reduction defect	March 2020	0.9988 (0.9922-1.0054)	0.718	0.8538 (0.6468-1.1270)	0.264	0.9979 (0.9875-1.0084)	0.694	1.07	1.82
<37 weeks	Limb reduction defect	December 2020	0.9976 (0.9932-1.0020)	0.279	0.7989 (0.5882-1.0849)	0.150	1.0055 (0.9894-1.0219)	0.504	1.07	1.85
<37 weeks	Cleft Lip with or without Cleft Palate	March 2020	0.9986 (0.9962-1.0009)	0.228	1.0371 (0.8988-1.1967)	0.618	0.9985 (0.9930-1.0040)	0.586	0.94	2.21
<37 weeks	Cleft Lip with or without Cleft Palate	December 2020	0.9982 (0.9967-0.9997)	0.018	1.1704 (1.0152-1.3493)	0.030	0.9905 (0.9835-0.9976)	0.008	0.89	2.34
<37 weeks	Cleft Palate alone	March 2020	0.9992 (0.9926-1.0058)	0.809	0.9731 (0.7644-1.2387)	0.825	1.0003 (0.9913-1.0094)	0.953	1.13	1.89
<37 weeks	Cleft Palate alone	December 2020	0.9994 (0.9950-1.0039)	0.808	0.8934 (0.7127-1.1198)	0.328	1.0052 (0.9945-1.0160)	0.345	1.13	1.89
<37 weeks	Down syndrome	March 2020	0.9983 (0.9967-1.0000)	0.049	1.1599 (1.0719-1.2550)	<0.001	0.9949 (0.9916-0.9982)	0.002	0.68	2.14
<37 weeks	Down syndrome	December 2020	0.9999 (0.9982-1.0016)	0.933	1.0517 (0.9809-1.1275)	0.156	0.9923 (0.9889-0.9958)	<0.001	0.73	2.17
<37 weeks	Suspected chromosomal disorder	March 2020	0.9978 (0.9951-1.0005)	0.108	1.0443 (0.9372-1.1637)	0.433	0.9996 (0.9942-1.0052)	0.900	1.03	1.85
<37 weeks	Suspected chromosomal disorder	December 2020	0.9981 (0.9961-1.0002)	0.077	1.0612 (0.9410-1.1968)	0.333	0.9969 (0.9885-1.0053)	0.467	1.03	1.85
<37 weeks	Hypospadias	March 2020	0.9975 (0.9951-1.0000)	0.050	0.9963 (0.8702-1.1407)	0.957	1.0001 (0.9953-1.0048)	0.979	0.99	2.33
<37 weeks	Hypospadias	December 2020	0.9979 (0.9963-0.9995)	0.012	0.9533 (0.8417-1.0796)	0.451	1.0015 (0.9938-1.0093)	0.698	0.99	2.33
<37 weeks	Transferred within 24 hours of delivery	March 2020	0.9994 (0.9989-0.9998)	0.006	0.9742 (0.9558-0.9929)	0.007	1.0002 (0.9994-1.0011)	0.576	1.11	2.26
<37 weeks	Transferred within 24 hours of delivery	December 2020	0.9989 (0.9985-0.9994)	<0.001	1.0053 (0.9798-1.0315)	0.687	0.9999 (0.9984-1.0013)	0.853	1.18	1.99
<37 weeks	Death before birth-certificate registration	March 2020	0.9958 (0.9951-0.9966)	<0.001	0.9675 (0.9125-1.0259)	0.269	1.0015 (0.9985-1.0045)	0.319	1.64	1.96

<37 weeks	Death before birth-certificate registration	December 2020	0.9958 (0.9952-0.9965)	<0.001	0.9241 (0.8718-0.9796)	0.008	1.0056 (1.0022-1.0090)	0.001	1.40	2.02

Note: Each row is one quasi-Poisson log-linear model of the monthly event count, with the monthly number of live births as offset, fitted to the 84 monthly observations from January 2016 to December 2022. Each model includes a linear pre-onset time trend, an immediate level change at the onset point, a subsequent slope change, and the first and second Fourier harmonics of the annual cycle; Newey-West standard errors (lag 4) were used to accommodate residual autocorrelation. Outcomes with fewer than 100 events in a subgroup were not modelled. Two onset points were pre-specified: March 2020, the first infants born after the pandemic was declared, and December 2020, the first infants whose first trimester fell entirely within the pandemic; the two onset points are alternative specifications of the same series, not a joint model. Dispersion is the estimated quasi-Poisson dispersion parameter and Durbin-Watson is the test statistic for residual autocorrelation, both reported as fit diagnostics. A level change dated to March 2020 births should not be read as a teratogenic effect, because those infants completed organogenesis before the pandemic began. CI, confidence interval; RR, rate ratio.

Supplementary Figures

Supplemental Figure S1 Adjusted odds ratios of the study outcomes by birth year (subgroup analysis, only infants delivered at ≥37 weeks' gestation)

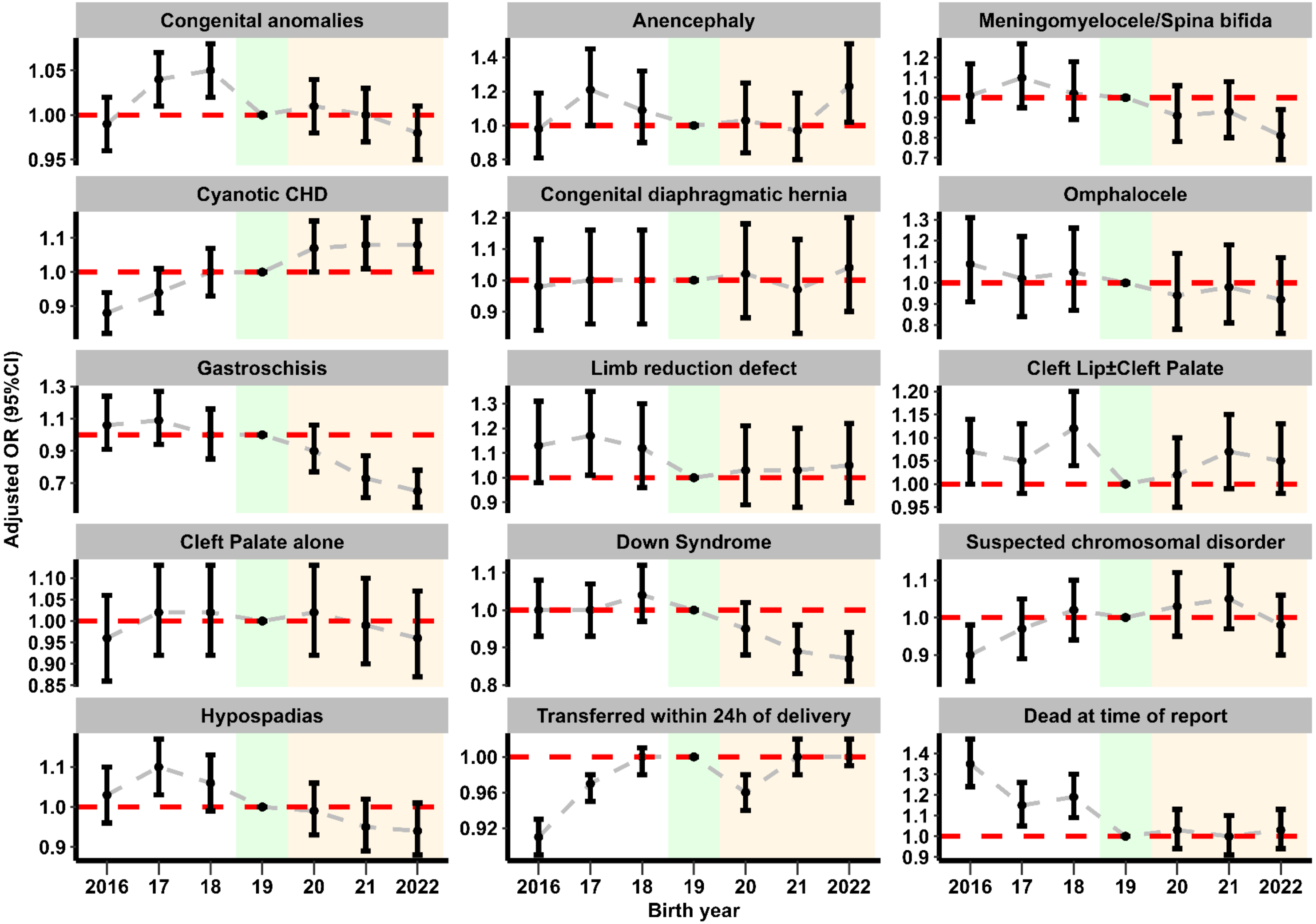

Note: Details about each variable are presented in Supplementary methods. Infants with missing obstetric estimate of gestation, or missing outcomes were excluded from the analyses of the specific outcomes. The presented odds ratios were adjusted for maternal age, maternal residence, maternal ethnicity/race, maternal education attainment, principal source of payment for current delivery, and marital status.
Abbreviations: OR, odds ratio; CI, confidence interval; CHD, congenital heart disease.

Supplemental Figure S2 Adjusted odds ratios of the study outcomes by birth year (subgroup analysis, only infants delivered at <37 weeks' gestation)

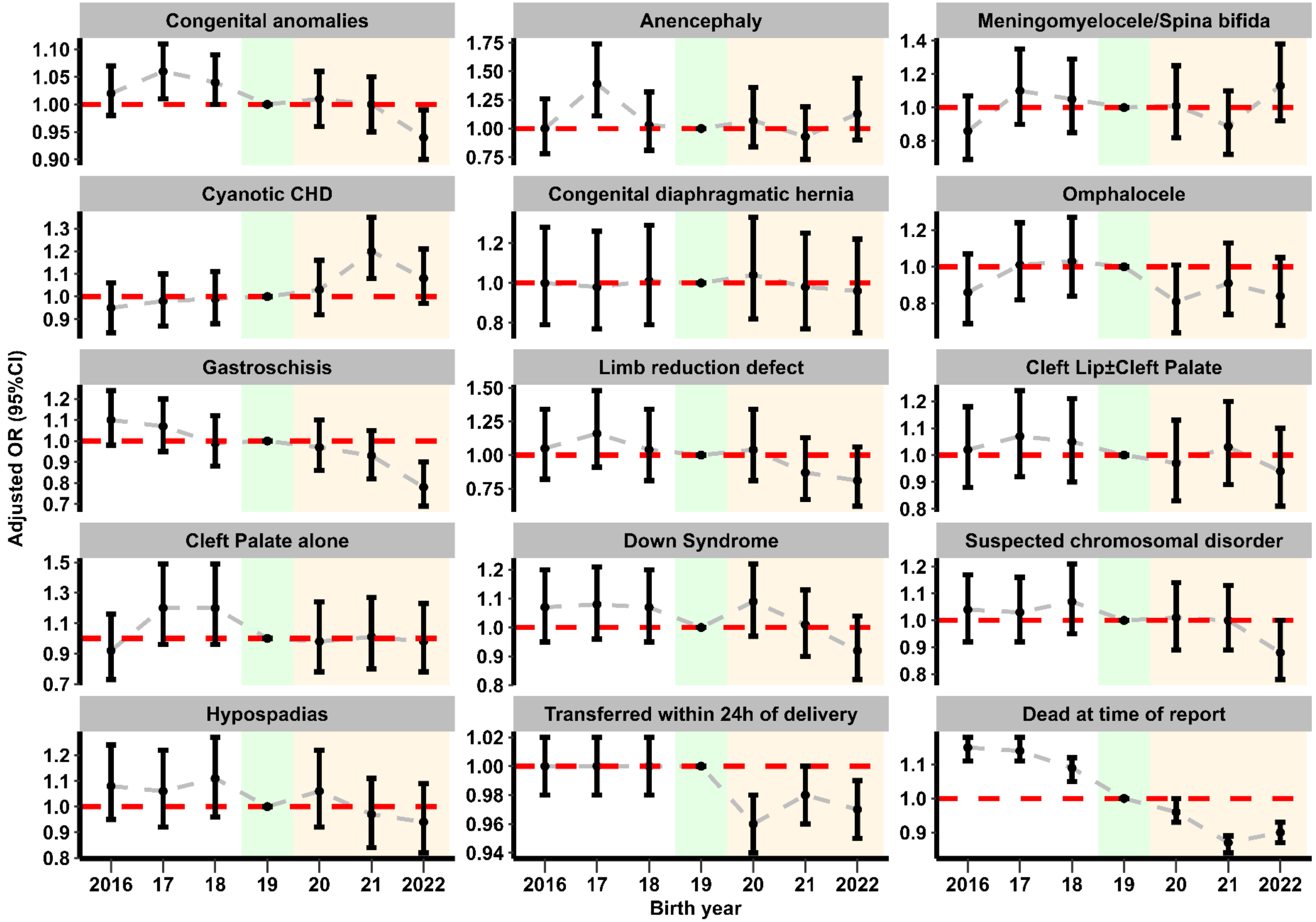

Note: Details about each variable are presented in Supplementary methods. Infants with missing obstetric estimate of gestation, or missing outcomes were excluded from the analyses of the specific outcomes. The presented odds ratios were adjusted for maternal age, maternal residence, maternal ethnicity/race, maternal education attainment, principal source of payment for current delivery, and marital status.
Abbreviations: OR, odds ratio; CI, confidence interval; CHD, congenital heart disease.

Supplemental Figure S3 Reported prevalence of anencephaly by birth month

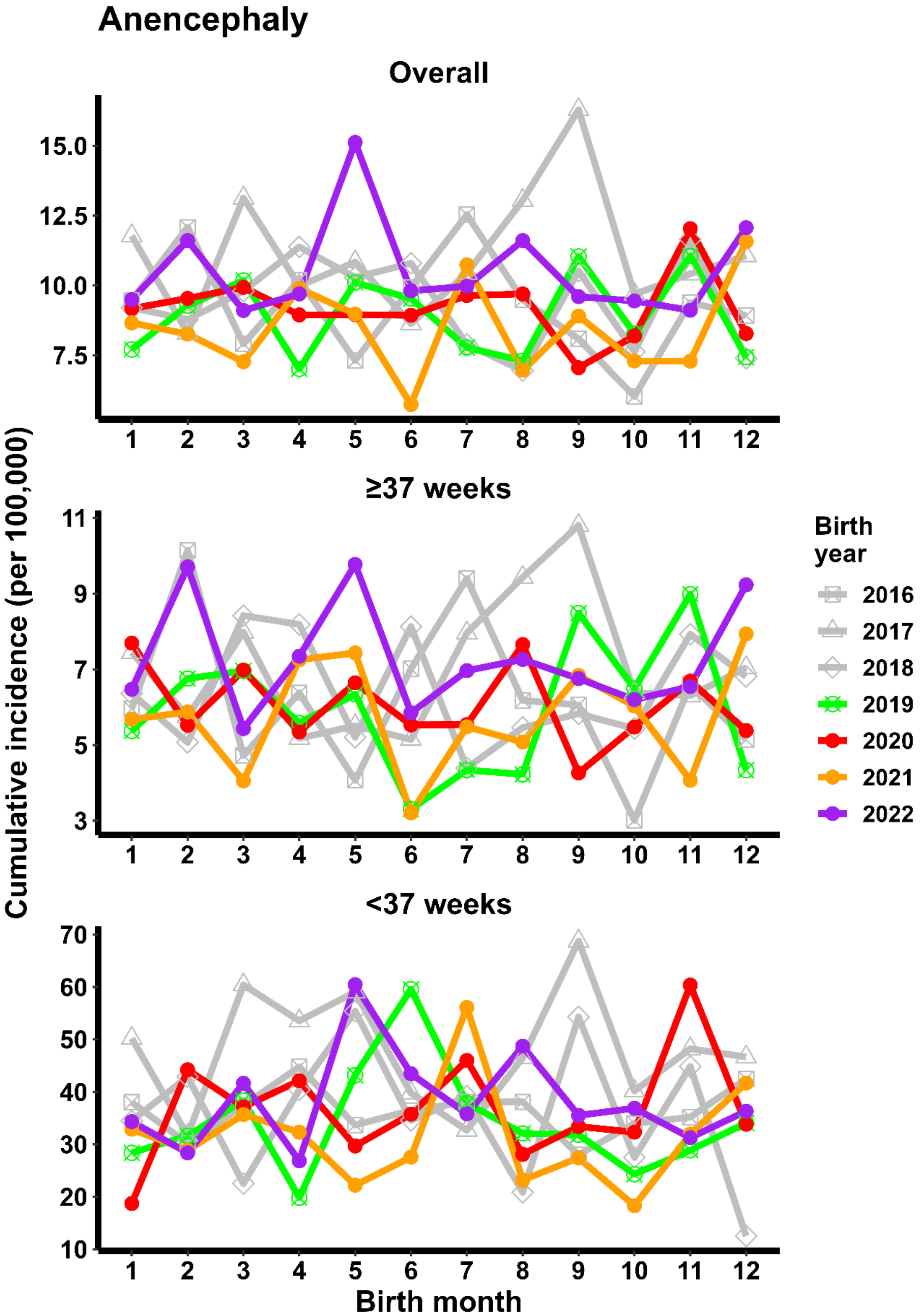

Note: Details about each variable are presented in Supplementary methods. Infants with missing obstetric estimate of gestation (in the subgroups by obstetric estimate of gestation), or missing outcome were excluded from analyses of the specific outcome.

Supplemental Figure S4 Reported prevalence of meningomyelocele/spina bifida by birth month

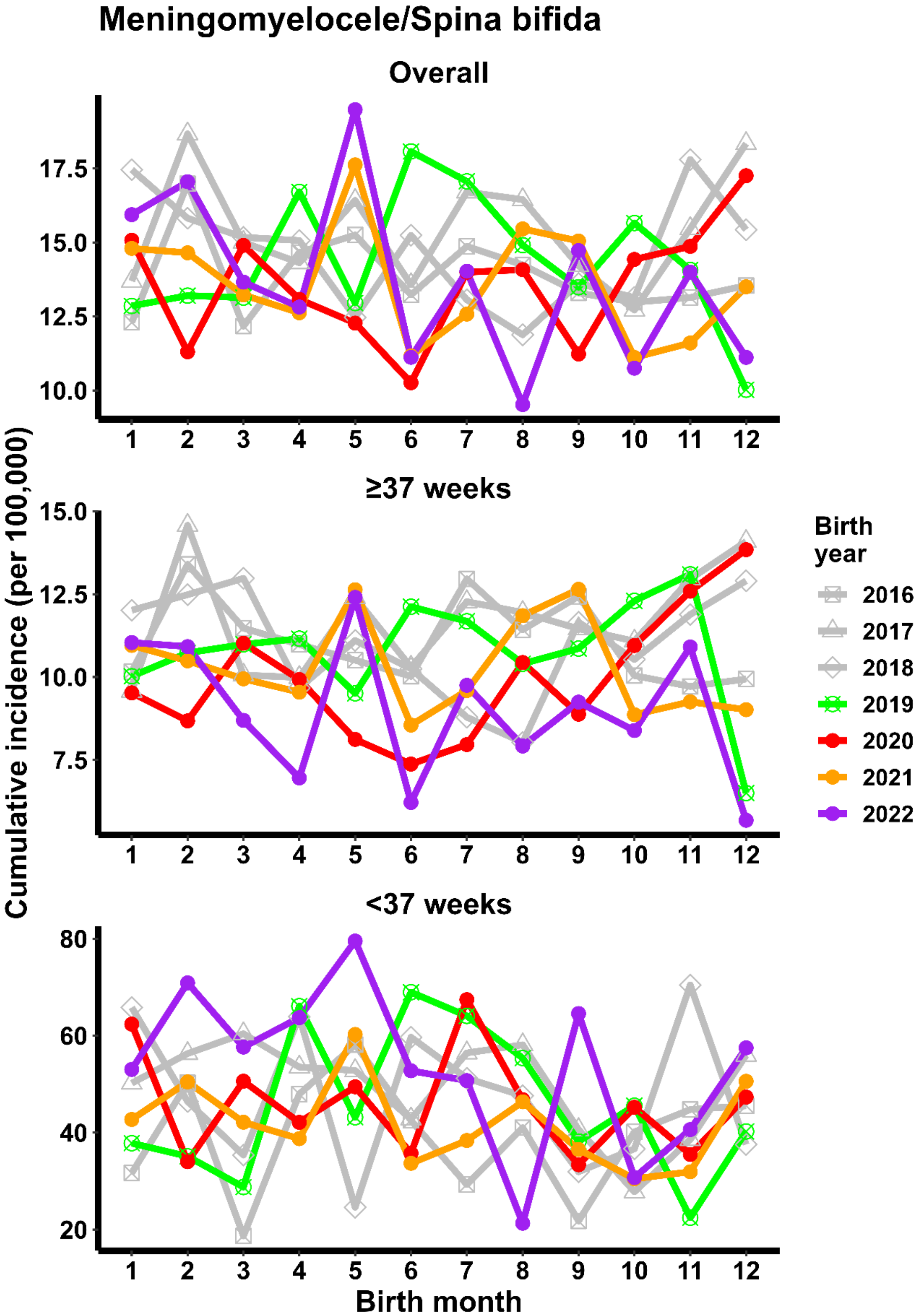

Note: Details about each variable are presented in Supplementary methods. Infants with missing obstetric estimate of gestation (in the subgroups by obstetric estimate of gestation), or missing outcome were excluded from analyses of the specific outcome.

Supplemental Figure S5 Reported prevalence of congenital diaphragmatic hernia by birth month

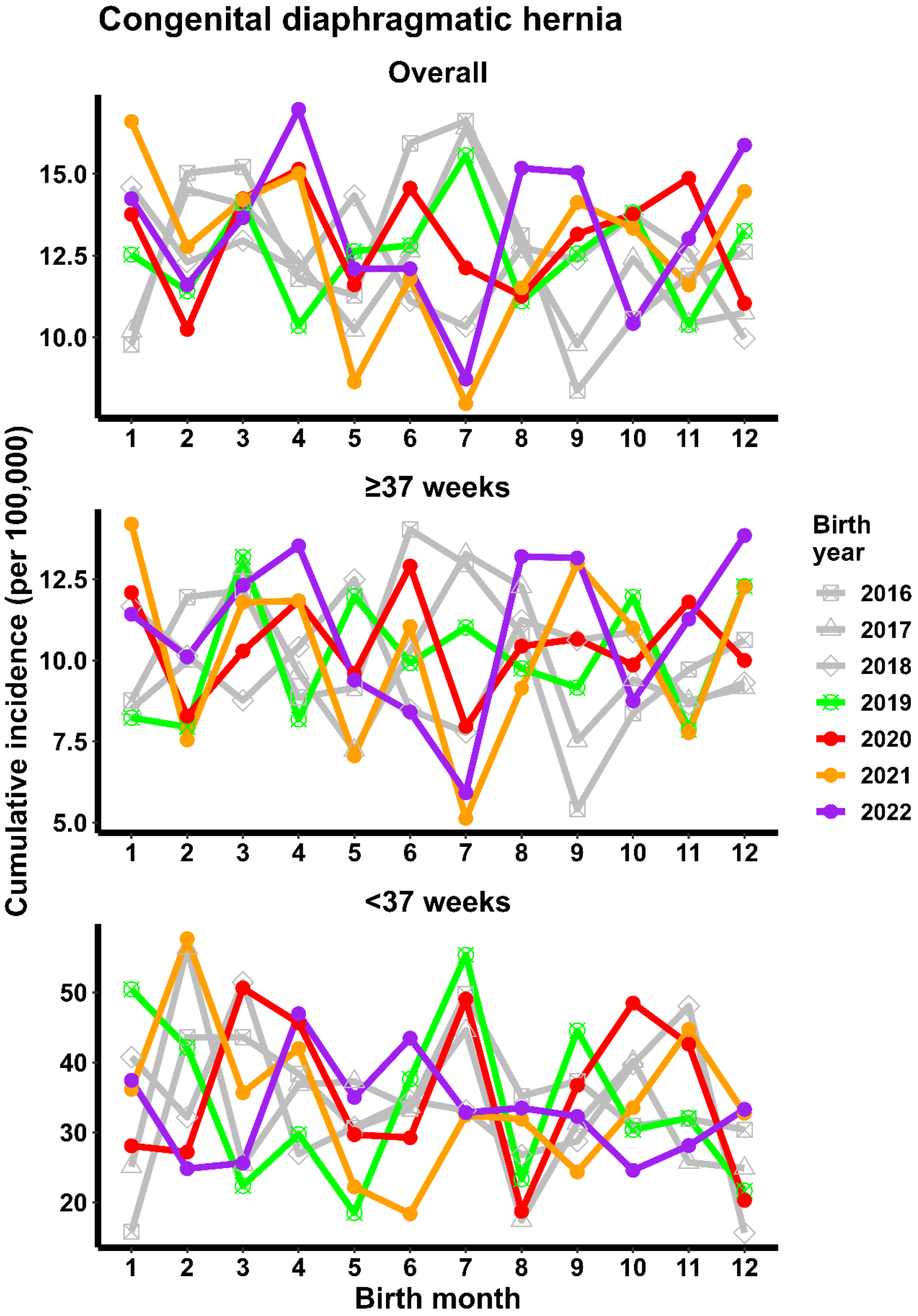

Note: Details about each variable are presented in Supplementary methods. Infants with missing obstetric estimate of gestation (in the subgroups by obstetric estimate of gestation), or missing outcome were excluded from analyses of the specific outcome.

Supplemental Figure S6 Reported prevalence of omphalocele by birth month

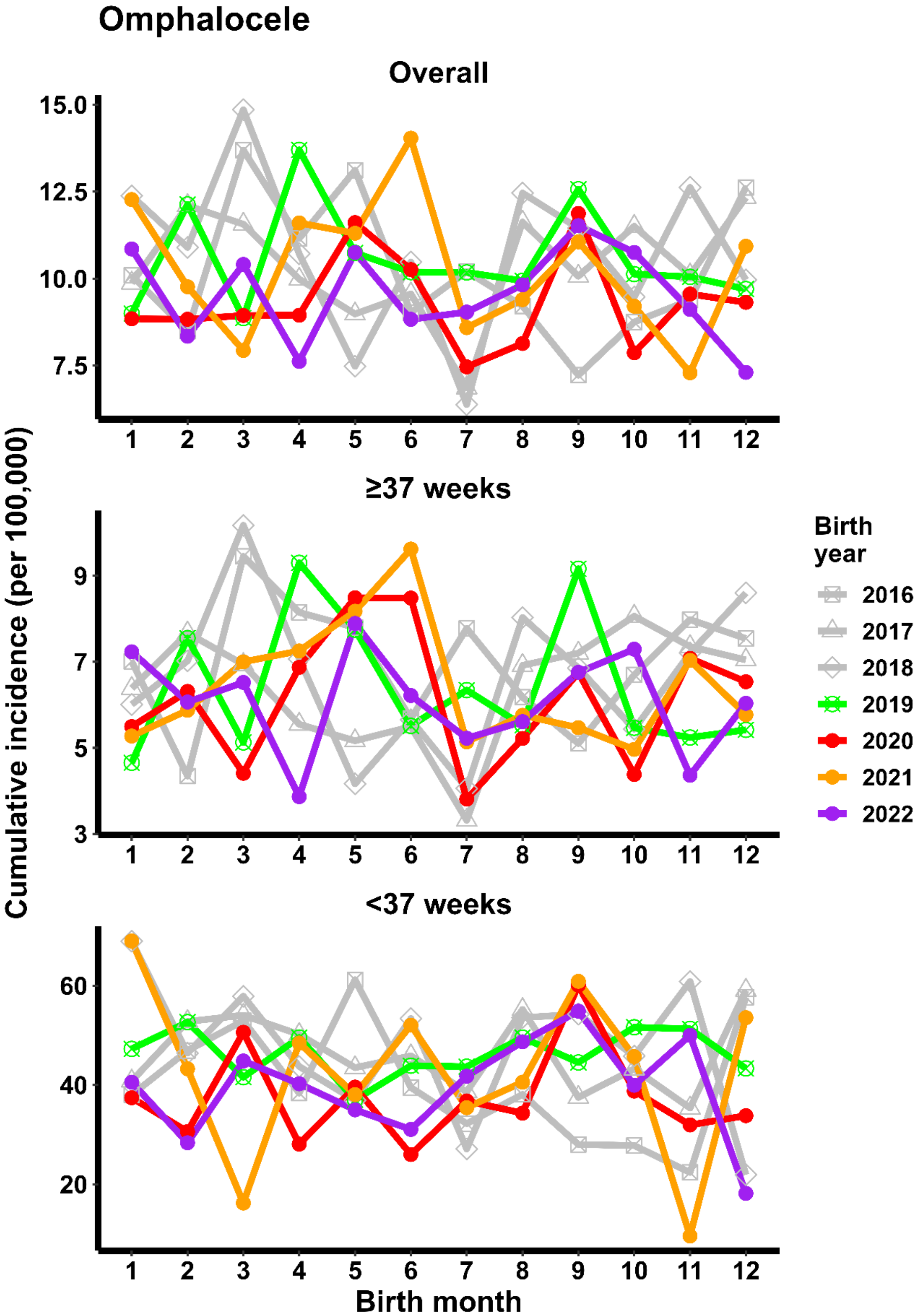

Note: Details about each variable are presented in Supplementary methods. Infants with missing obstetric estimate of gestation (in the subgroups by obstetric estimate of gestation), or missing outcome were excluded from analyses of the specific outcome.

Supplemental Figure S7 Reported prevalence of gastroschisis by birth month

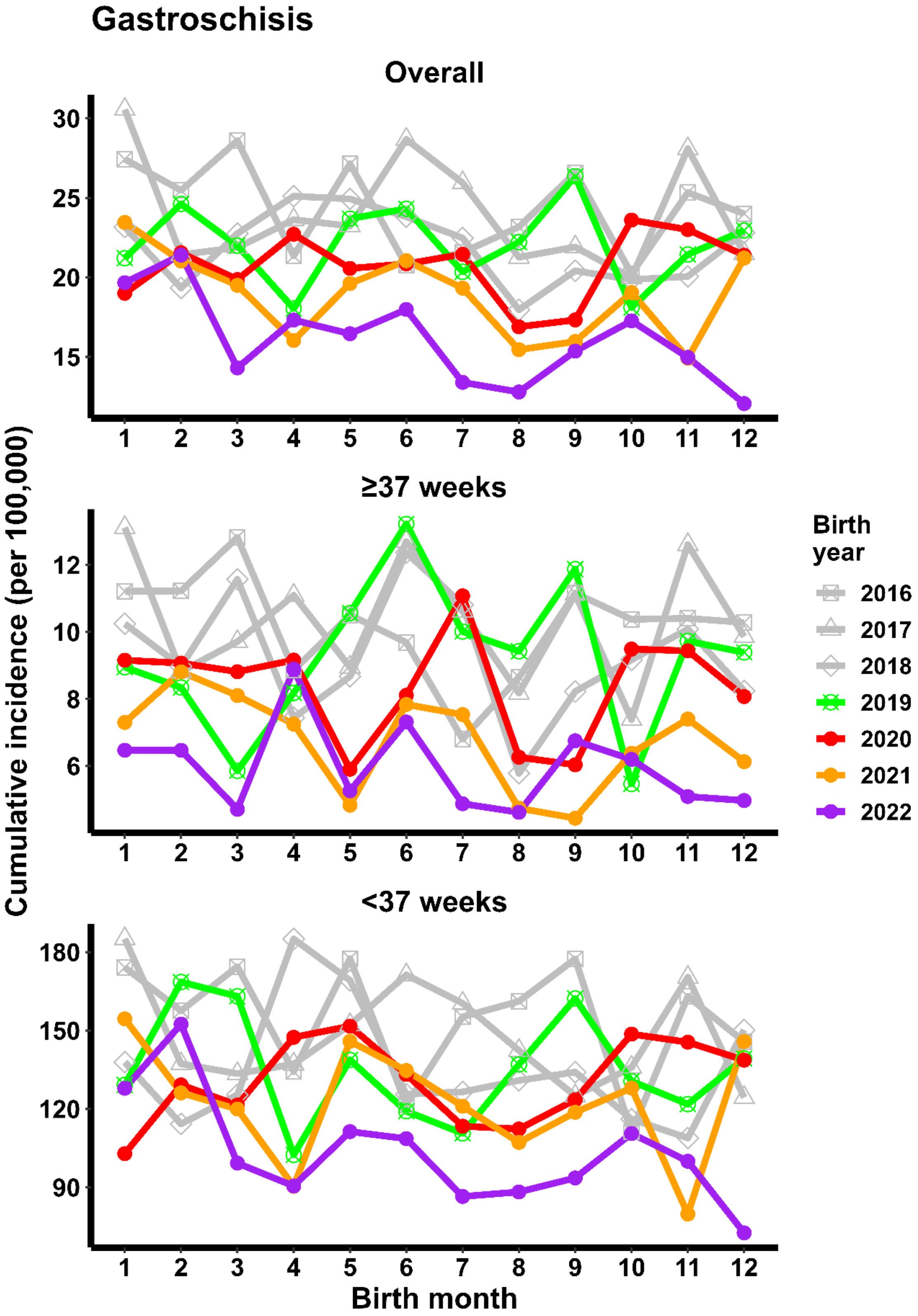

Note: Details about each variable are presented in Supplementary methods. Infants with missing obstetric estimate of gestation (in the subgroups by obstetric estimate of gestation), or missing outcome were excluded from analyses of the specific outcome.

Supplemental Figure S8 Reported prevalence of limb reduction defect by birth month

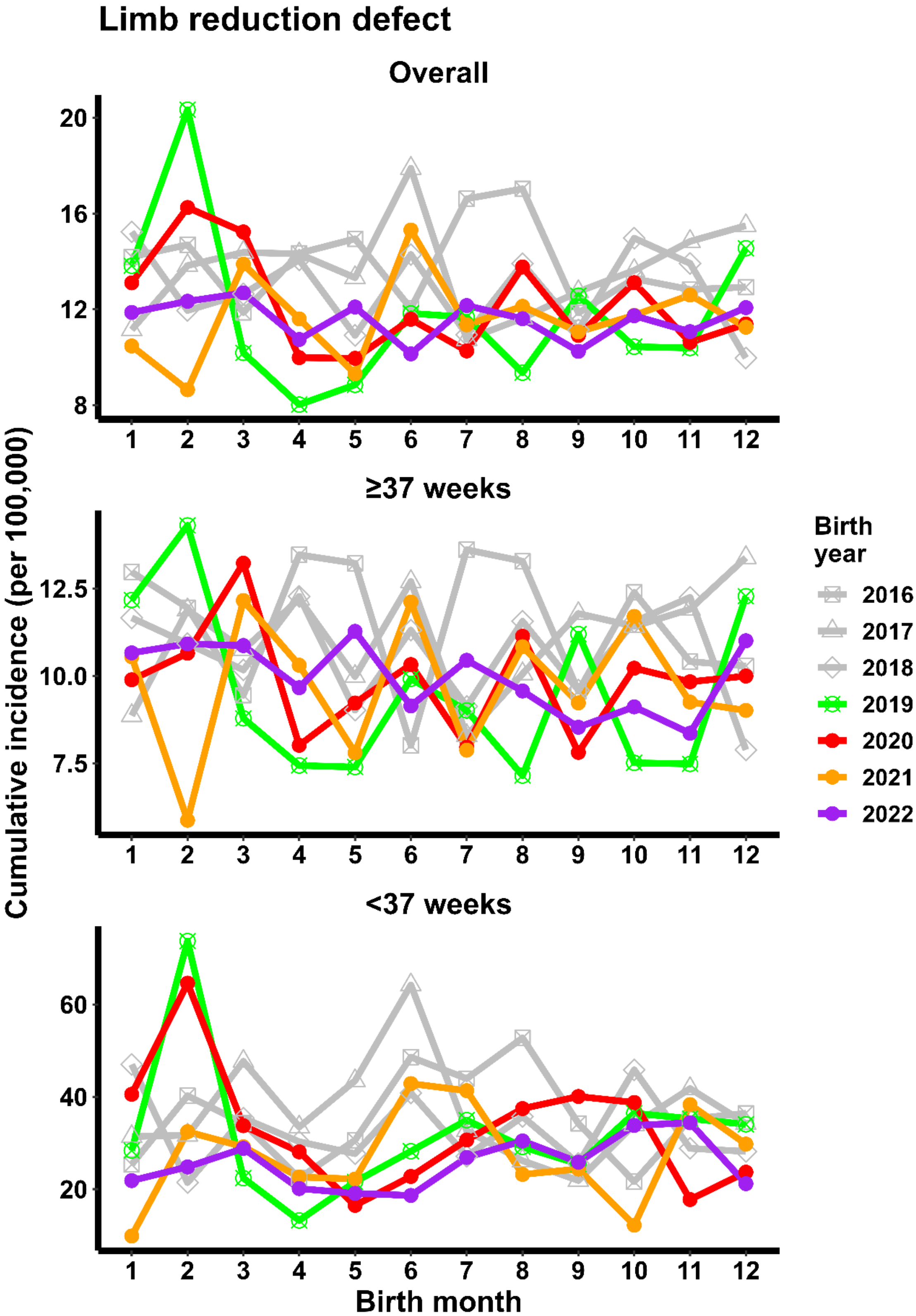

Note: Details about each variable are presented in Supplementary methods. Infants with missing obstetric estimate of gestation (in the subgroups by obstetric estimate of gestation), or missing outcome were excluded from analyses of the specific outcome.

Supplemental Figure S9 Reported prevalence of cleft lip with or without cleft palate by birth month

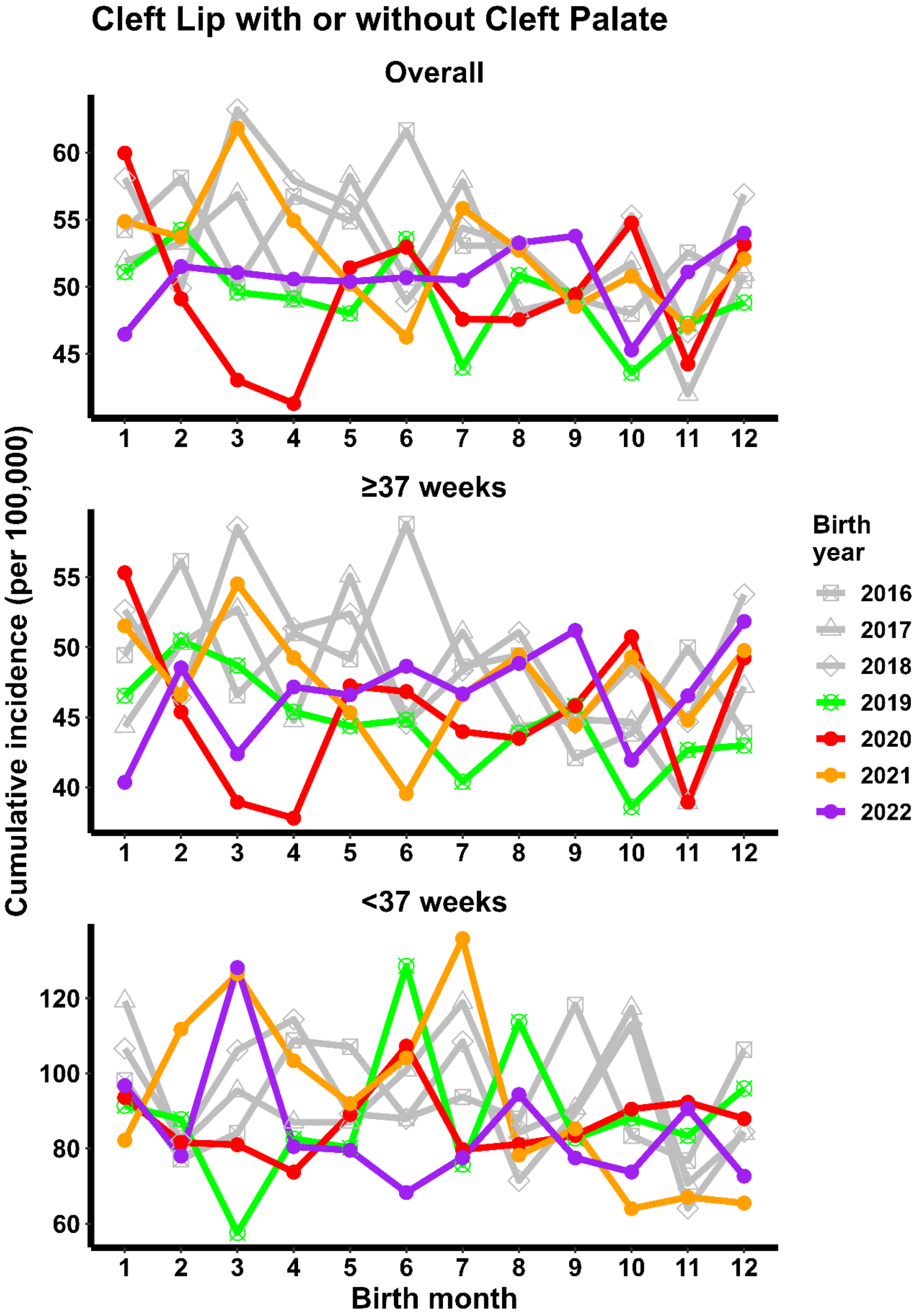

Note: Details about each variable are presented in Supplementary methods. Infants with missing obstetric estimate of gestation (in the subgroups by obstetric estimate of gestation), or missing outcome were excluded from analyses of the specific outcome.

Supplemental Figure S10 Reported prevalence of cleft palate alone by birth month

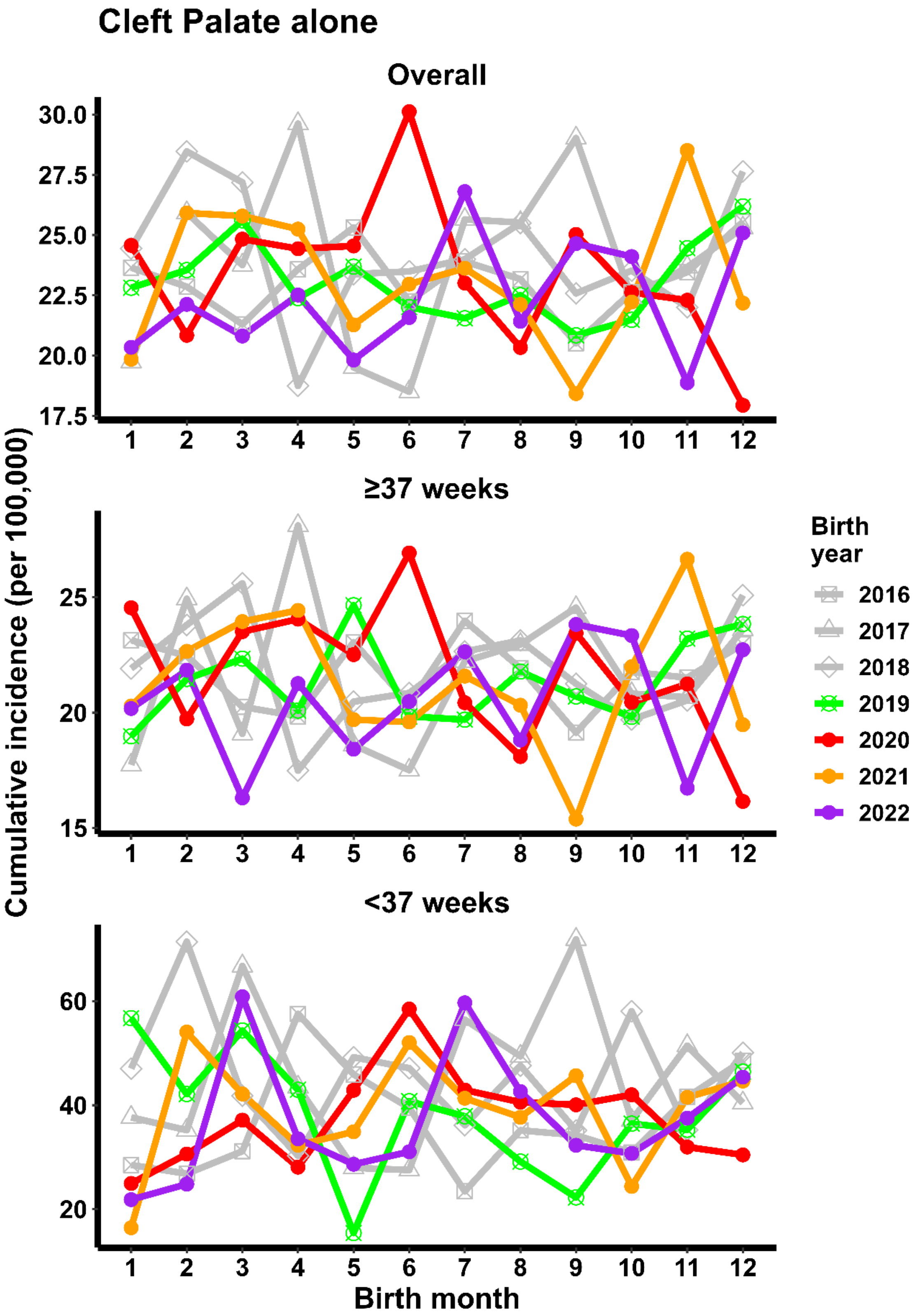

Note: Details about each variable are presented in Supplementary methods. Infants with missing obstetric estimate of gestation (in the subgroups by obstetric estimate of gestation), or missing outcome were excluded from analyses of the specific outcome.

Supplemental Figure S11 Reported prevalence of Down syndrome by birth month

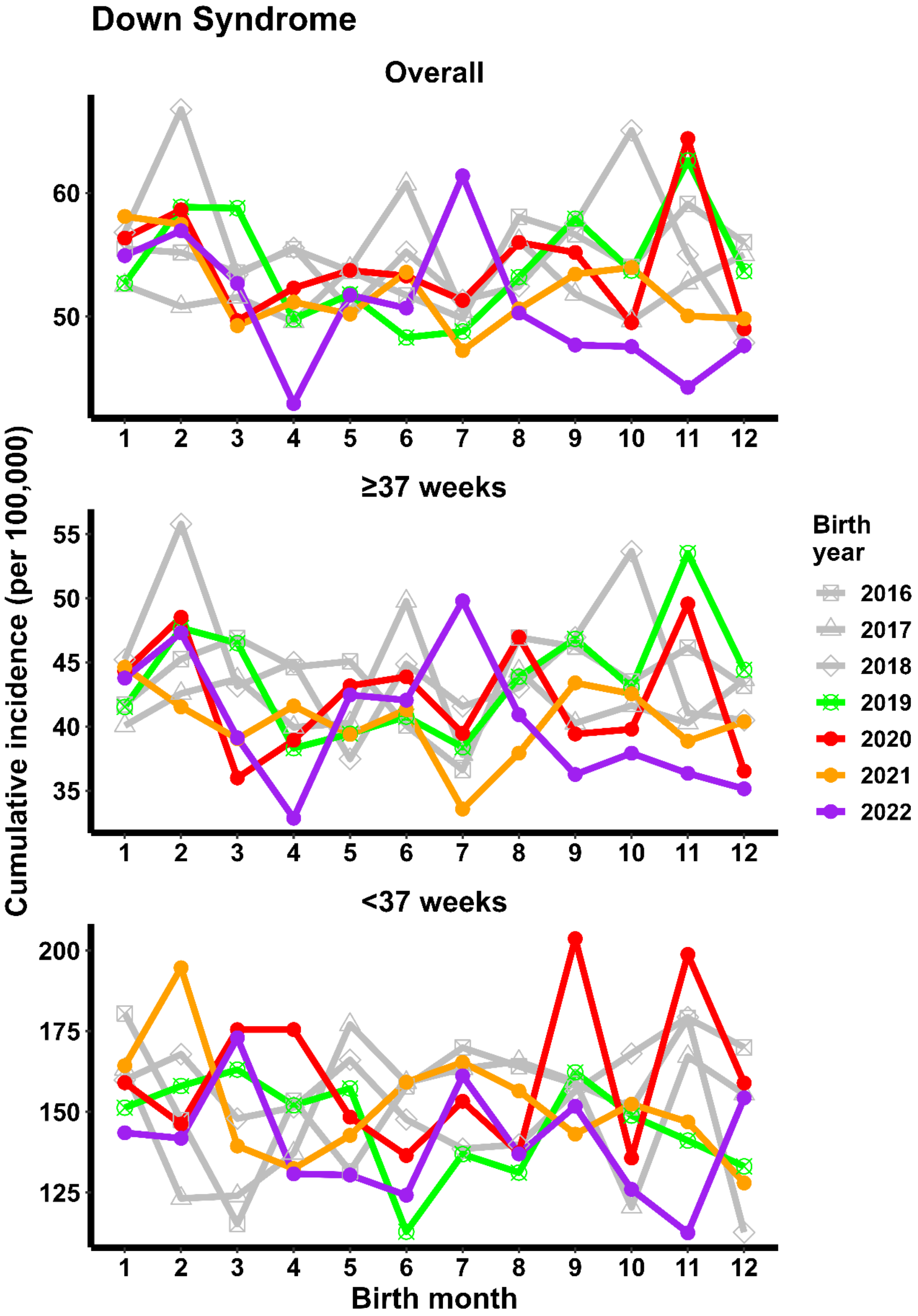

Note: Details about each variable are presented in Supplementary methods. Infants with missing obstetric estimate of gestation (in the subgroups by obstetric estimate of gestation), or missing outcome were excluded from analyses of the specific outcome.

Supplemental Figure S12 Reported prevalence of suspected chromosomal disorder by birth month

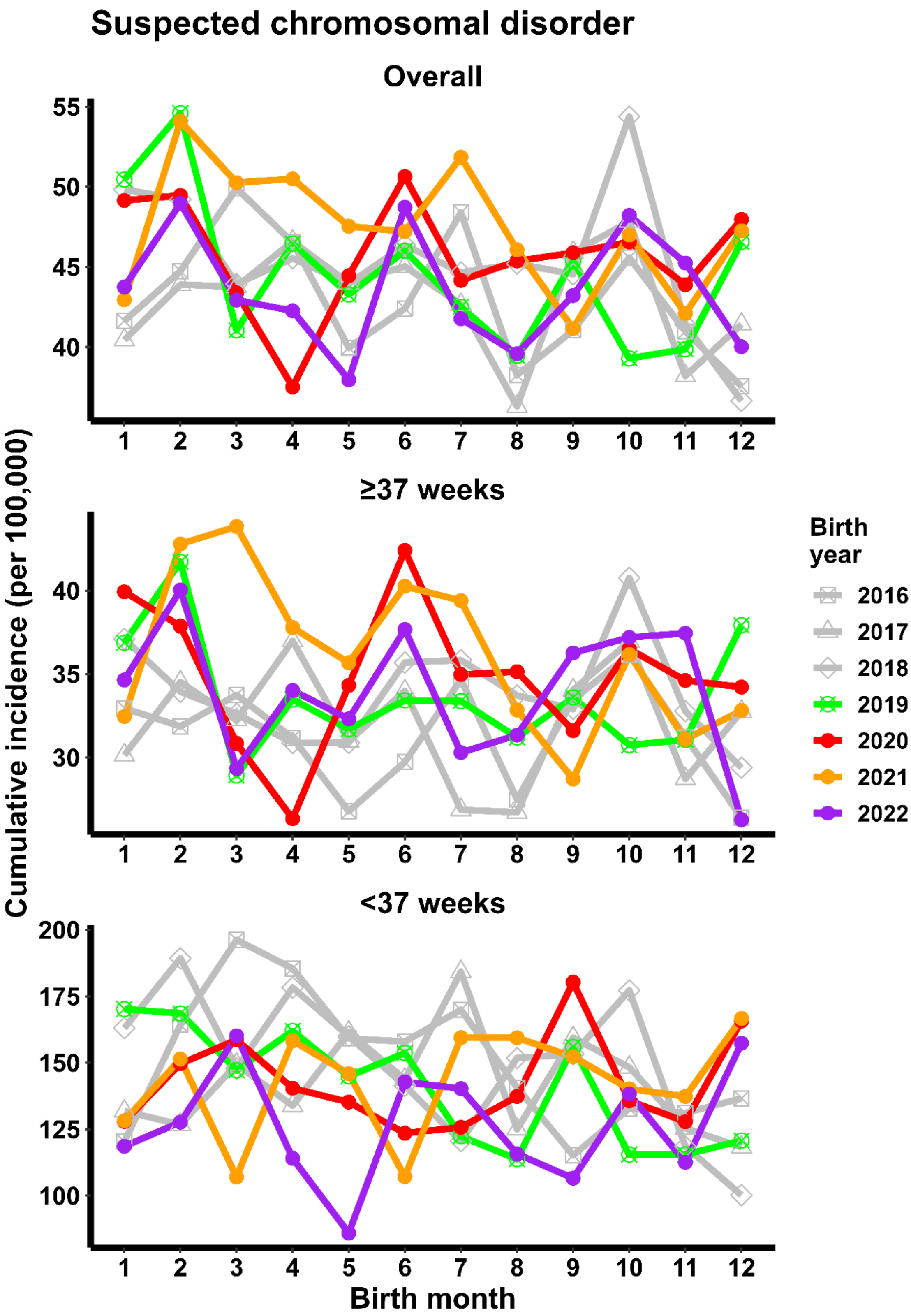

Note: Details about each variable are presented in Supplementary methods. Infants with missing obstetric estimate of gestation (in the subgroups by obstetric estimate of gestation), or missing outcome were excluded from analyses of the specific outcome.

Supplemental Figure S13 Reported prevalence of hypospadias by birth month

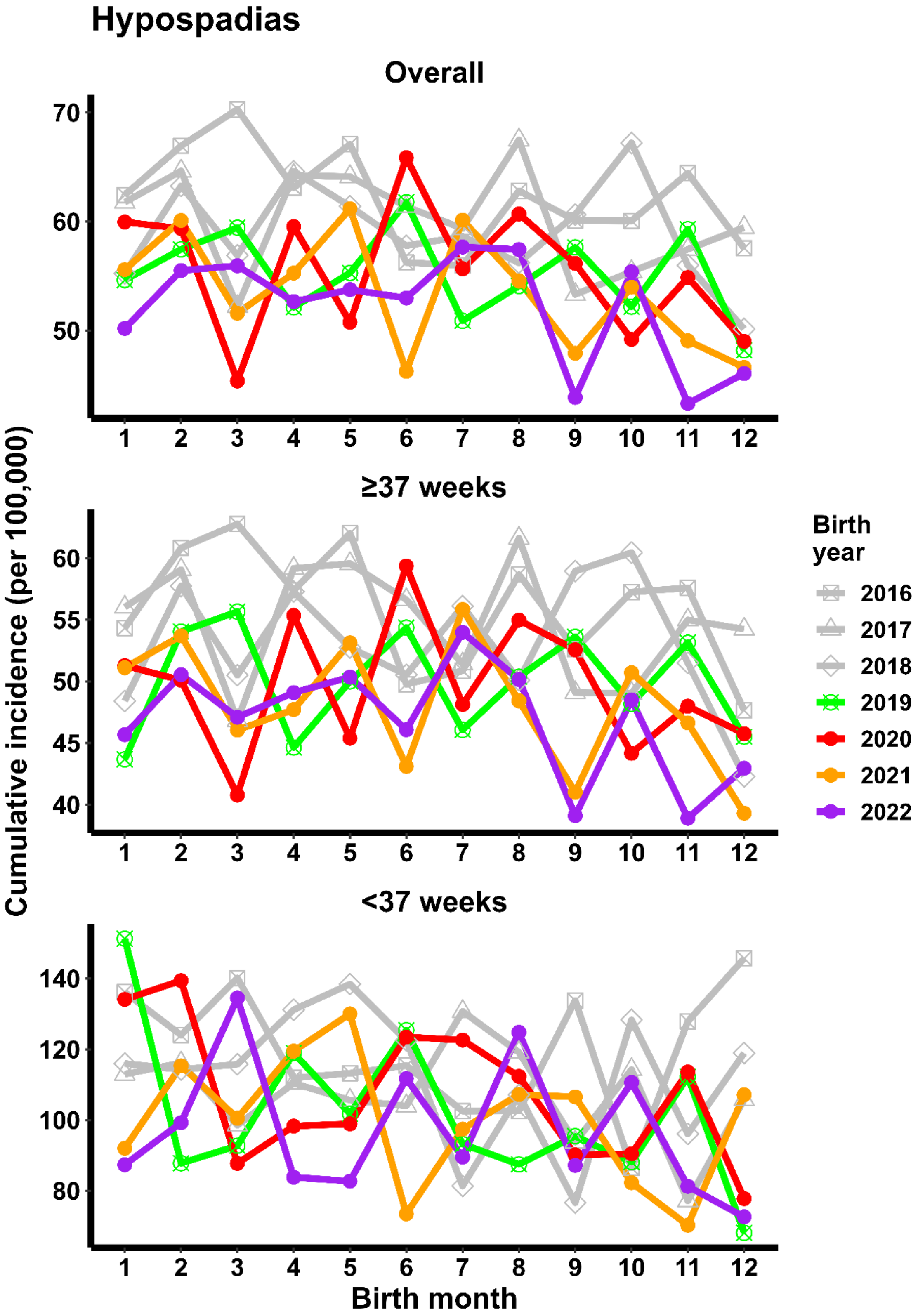

Note: Details about each variable are presented in Supplementary methods. Infants with missing obstetric estimate of gestation (in the subgroups by obstetric estimate of gestation), or missing outcome were excluded from analyses of the specific outcome.

Supplemental Figure S14 Reported prevalence of transfer within 24 hours of delivery by birth month

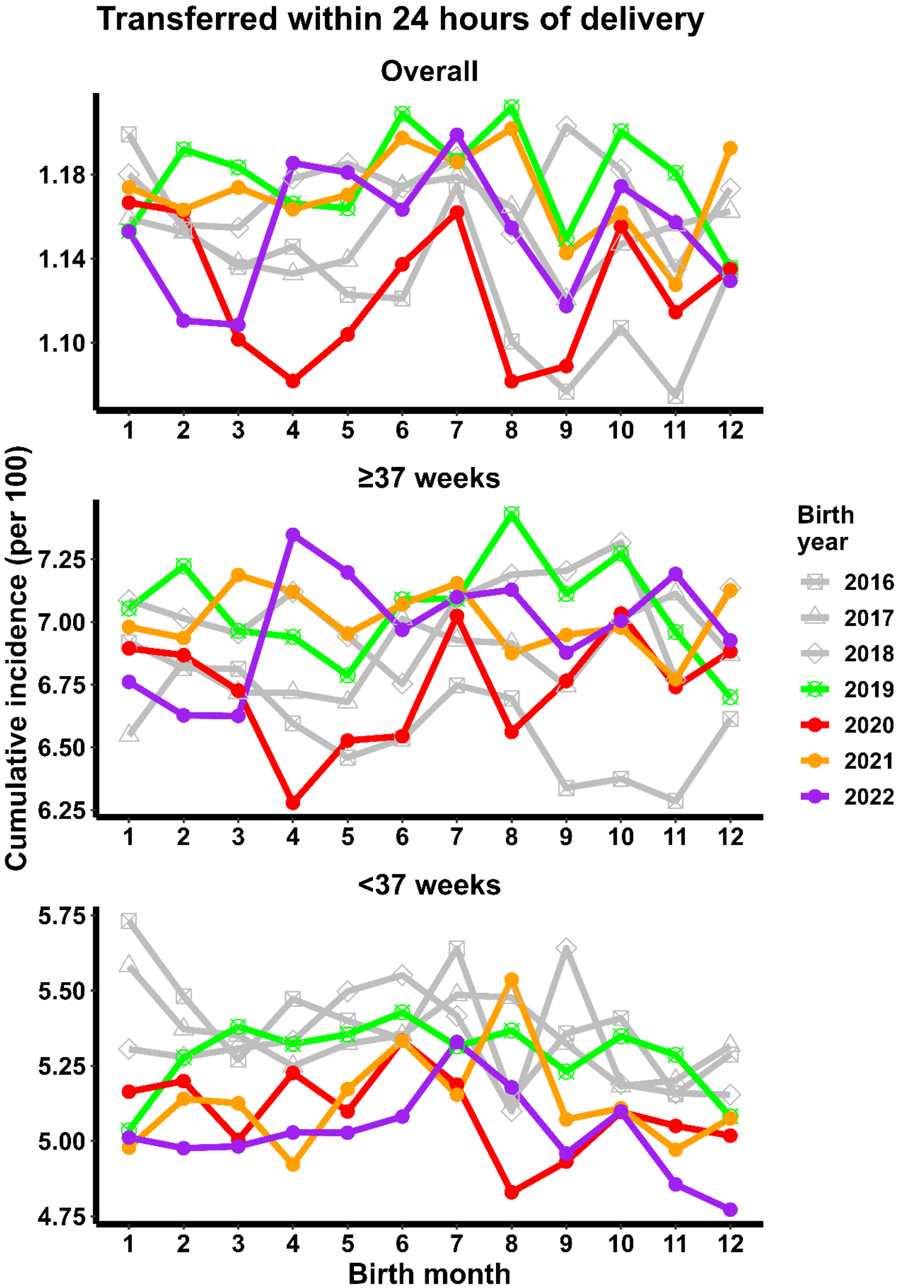

Note: Details about each variable are presented in Supplementary methods. Infants with missing obstetric estimate of gestation (in the subgroups by obstetric estimate of gestation), or missing outcome were excluded from analyses of the specific outcome.

Supplemental Figure S15 Reported prevalence of death before birth-certificate registration by birth month

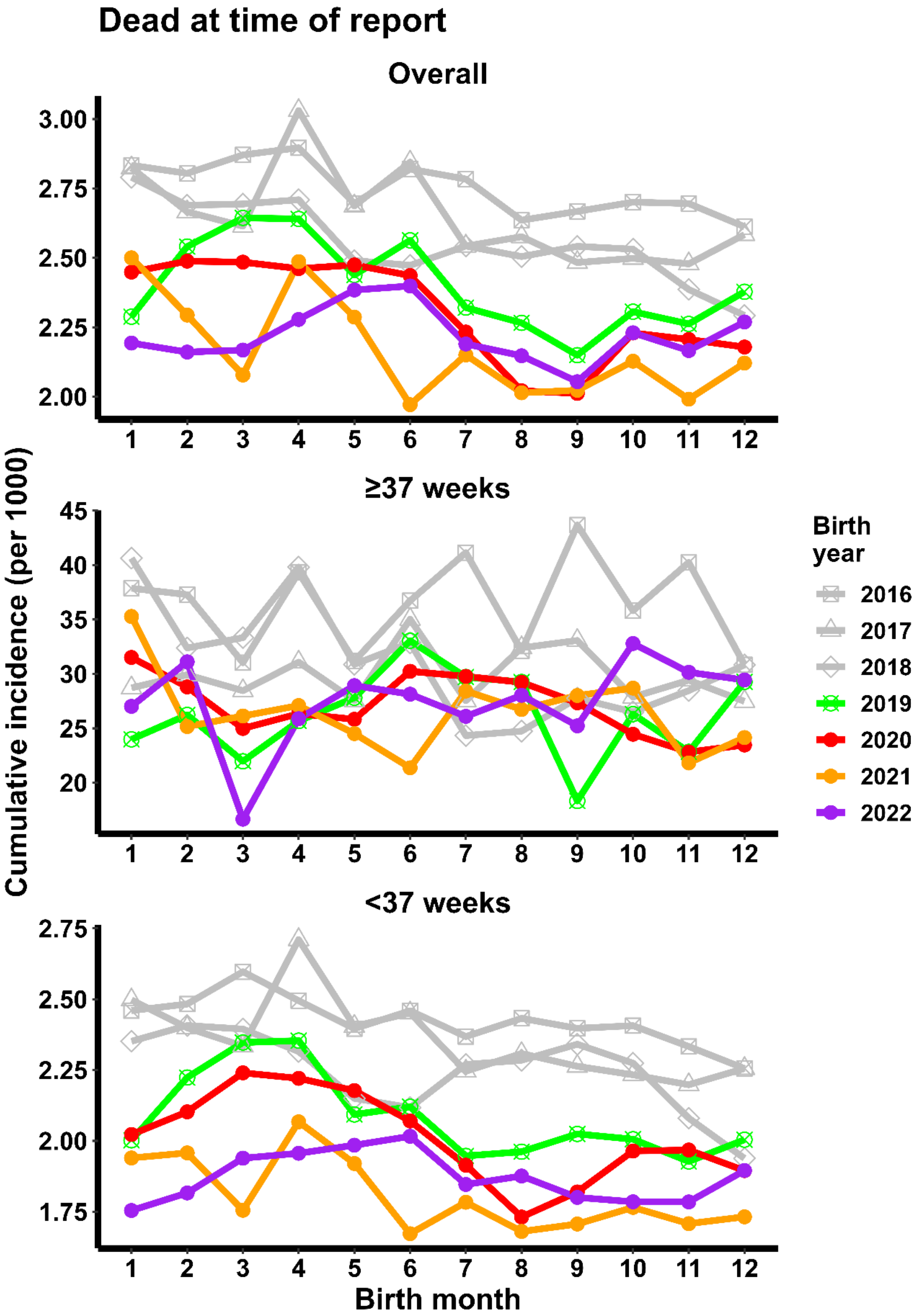

Note: Details about each variable are presented in Supplementary methods. Infants with missing obstetric estimate of gestation (in the subgroups by obstetric estimate of gestation), or missing outcome were excluded from analyses of the specific outcome.

Supplemental Figure S16 Segmented regression of the monthly reported prevalence of cyanotic congenital heart disease, 2016-2022

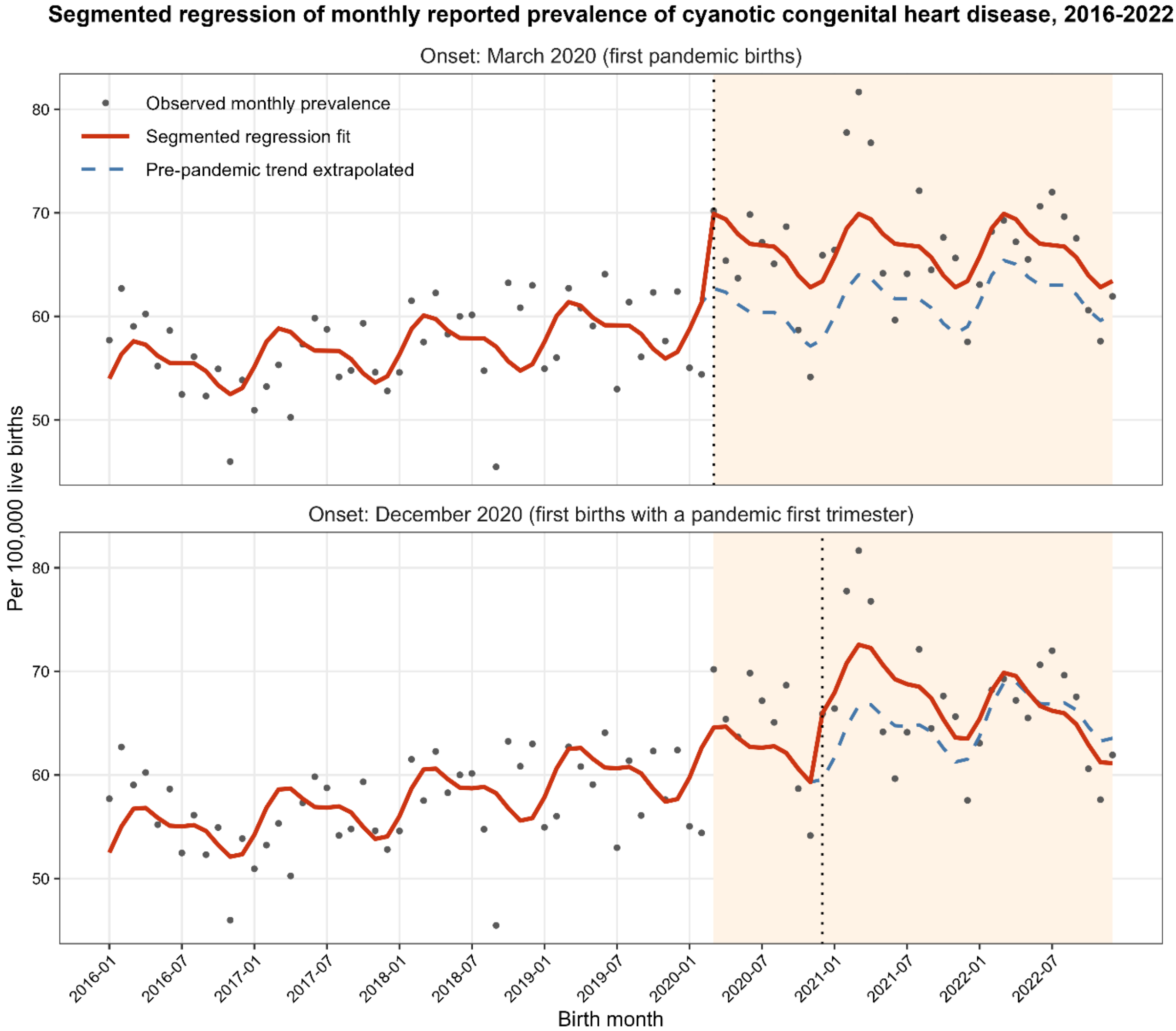

Note: Points are the observed monthly birth-certificate–reported prevalence of cyanotic congenital heart disease at live birth, per 100,000 live births. The solid line is the fitted quasi-Poisson segmented regression, including the seasonal harmonics, and therefore oscillates within each year; the dashed line is the pre-onset segment extrapolated forward, that is the trajectory expected had the pre-pandemic level and slope continued. The two panels are alternative specifications of the same series. Full model output, including the pre-onset trend, the level change and the slope change with 95% confidence intervals, is given in Supplemental Table S15.
